# Long, Synthetic *Staphylococcus aureus* Type 8 Capsular Oligosaccharides Reveal Structural Epitopes for Effective Immune Recognition

**DOI:** 10.1021/jacs.4c16118

**Published:** 2025-01-10

**Authors:** Kitt Emilie Østerlid, Charlotte Sorieul, Luca Unione, Sizhe Li, Cristian García-Sepúlveda, Filippo Carboni, Linda Del Bino, Francesca Berni, Ana Arda, Herman S. Overkleeft, Gijsbert A. van der Marel, Maria Rosaria Romano, Jesús Jiménez-Barbero, Roberto Adamo, Jeroen D. C. Codée

**Affiliations:** † Leiden Institute of Chemistry, Leiden University, Einsteinweg 55, 2333 CC Leiden, The Netherlands; ‡ GSK Siena, Via Fiorentina, 1, 53100 Siena SI, Italy; § Center for Cooperative Research in Biosciences (CIC bioGUNE), Basque Research and Technology Alliance (BRTA), 48160 Derio, Bizkaia, Spain; ∥ Ikerbasque, Basque Foundation for Science, Bilbao 48009, Spain; ⊥ Department of Organic & Inorganic Chemistry, Faculty of Science and Technology, University of the Basque Country, EHU-UPV, 48940 Leioa, Bizkaia, Spain; # Centro de Investigacion Biomedica En Red de Enfermedades Respiratorias, 28029 Madrid, Spain

## Abstract

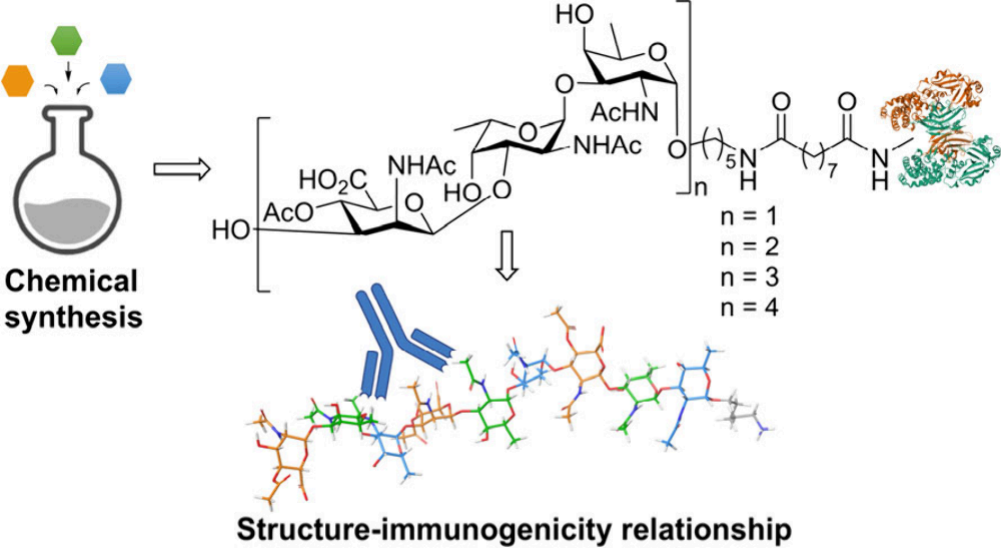

*Staphylococcus aureus* is a Gram-positive bacterium that is responsible for severe nosocomial infections. The rise of multidrug-resistant strains, which can pose significant health threats, prompts the development of new treatment interventions, and much attention has been directed at the development of prophylactic and therapeutic vaccination strategies. Capsular polysaccharides (CPs) are key protective elements of the *S. aureus* cell wall and have been proposed as promising candidate antigens. Thirteen different CP serotypes have been identified to date, of which types 5 and 8 are the most prominent. CP8 is composed of trisaccharide repeating units that are built up from an *N*-acetyl-4-*O*-acetyl*-β*-d-mannosaminuronic acid, that carries a C-4-*O*-acetyl, an *N*-acetyl-α-d-fucosamine, and an *N*-acetyl-α-l-fucosamine. Synthetic oligosaccharides are valuable tools to unravel the immunogenicity of bacterial oligosaccharides at the molecular level. However, the rare monosaccharides, *cis*-glycosidic linkages, and *O*-acetylation represent significant challenges for the synthesis of CP8 fragments. Here the stereoselective assembly of well-defined CP8 fragments, comprising a trimer, hexamer, nonamer, and dodecamer, is presented. This is the first time that fragments larger than a single repeating trisaccharide, which has been proven to be insufficient for antigenic activity, have been assembled. Structural studies have revealed a linear conformation for the oligosaccharides, with each trisaccharide repeat tilted ∼90° with respect to the flanking repeats, which is stabilized by the acetyl groups that prevent rotation around the glycosidic linkages. The *N*-acetyl groups in each repeating unit point in the same direction, generating a hydrophobic flank in the trisaccharide repeats. We applied the oligomers to generate model glycoconjugate vaccine modalities, which we then used to raise anti-CP8 antibodies. The antibody interaction and immunization studies have revealed a clear length dependent structure–activity relationship for the oligosaccharides, with an oligosaccharide of at least three repeating units required for an adequate immune response.

## Introduction

*Staphylococcus aureus* (*S. aureus*), a Gram-positive bacterium that is part of our microbiome, is one of the most common opportunistic pathogens. It is found in human mucous membranes and skin, and when these barriers are breached, it can cause various diseases, ranging from minor skin abscesses to deadly bloodstream infections (bacteremia), heart valve infections (endocarditis), bone infections (osteomyelitis), lung infections (pneumonia), meningitis, and septic shock.^1,2^ It especially poses a threat to newborns and immunocompromised patients, such as elderly, postsurgical, and dialysis patients. *S. aureus* is one of the ESKAPE bacteria (the collection of multidrug resistant bacteria that include *Enterococcus faecium*, *Staphylococcus aureus*, *Klebsiella pneumoniae*, *Acinetobacter baumannii*, *Pseudomonas aeruginosa*, and *Enterobacter* spp.) and a WHO high priority pathogen with the rise of antibiotic-resistant strains,^3^ like methicillin-resistant *S. aureus* (MRSA)^4^ and vancomycin-resistant *S. aureus* (VRSA).^5^ This urges the development of new therapeutic strategies, such as active or passive vaccination strategies.^6,7^ The complex cell wall of *S. aureus* features several characteristic glycopolymers,^8−10^ including capsular polysaccharides (CPs), wall teichoic acids (WTA), and lipoteichoic acids (LTA) that may be used as targets for agents eliciting a protective immune response.^11,12^ Various bacterial CPs have been used to develop antibacterial vaccines, and glycoconjugate vaccines have become one of the most effective and safe preventive treatments to combat bacterial infections. To date 13 different *S. aureus* CP serotypes have been identified from clinical isolates with CP type 5 (CP5) and type 8 (CP8) being the most abundant, comprising more than 80% of the clinical isolates.^13−16^ Conjugate vaccines, generated using isolated CP5 and CP8 *S. aureus* polysaccharides, have been explored up to phase III trials, where they unfortunately and surprisingly showed limited efficacy.^17−20^ Suboptimal epitope presentation may hinder eliciting a sufficient immune response against conjugated heterogeneous polysaccharides, and therefore synthetic oligosaccharides have attracted attention.^17,21^

**Figure 1:**
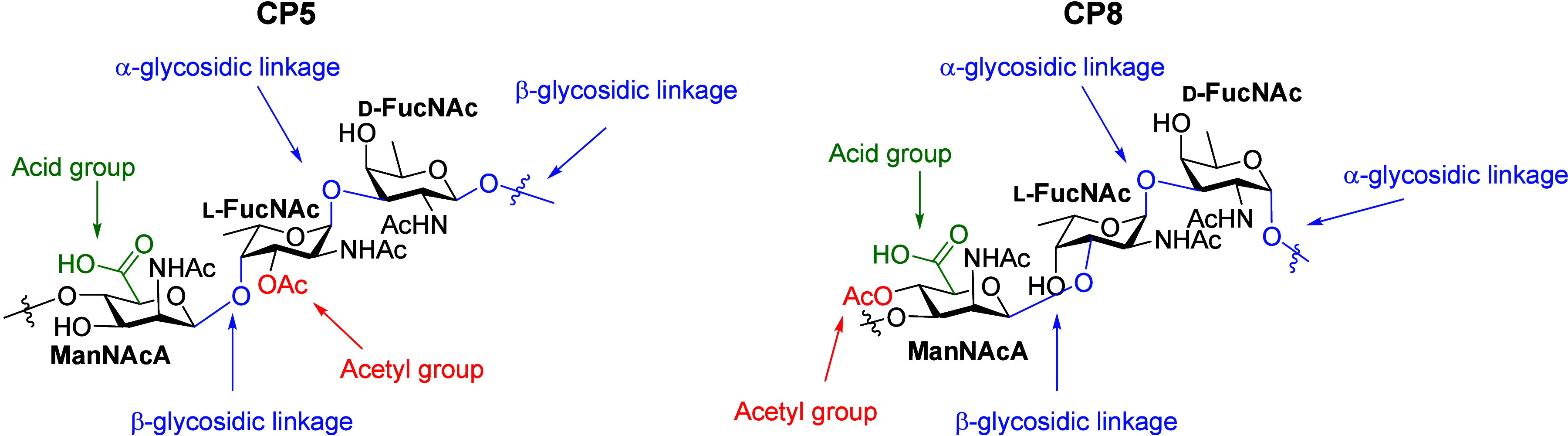
Representation of the common RUs of CP5 and CP8.

The structures of *S. aureus* CP5 and CP8 share the same three constituting rare monosaccharides: *N*-acetyl d-mannosaminuronic acid (ManNAcA), *N*-acetyl l-fucosamine (l-FucNAc), and *N*-acetyl d-fucosamine (d-FucNAc), as depicted in Figure 1.^22−24^ They differ in glycosidic linkages and acetylation patterns. CP8 was first isolated in 1984 by Fournier^25^ and originally thought to consist of *N*-acetyl fucosamine and *N*-acetyl galactosaminuronic acid. This was revised in 1988 when the chemical structure was found to be similar to CP5,^22^ and in 2005, Jones established this structure to have the repeating unit (RU) →3-β-d-ManNAcA(4-*O*Ac)-(1→3)-α-l-FucNAc-(1→3)-α-d-FucNAc-(1→.^24^ CP8 is acetylated at the C-4 position of the ManNAcA residue, and this acetylation has been found to be important for the induction of protective anti-CP antibody responses upon vaccination.^26^

**Figure 2:**
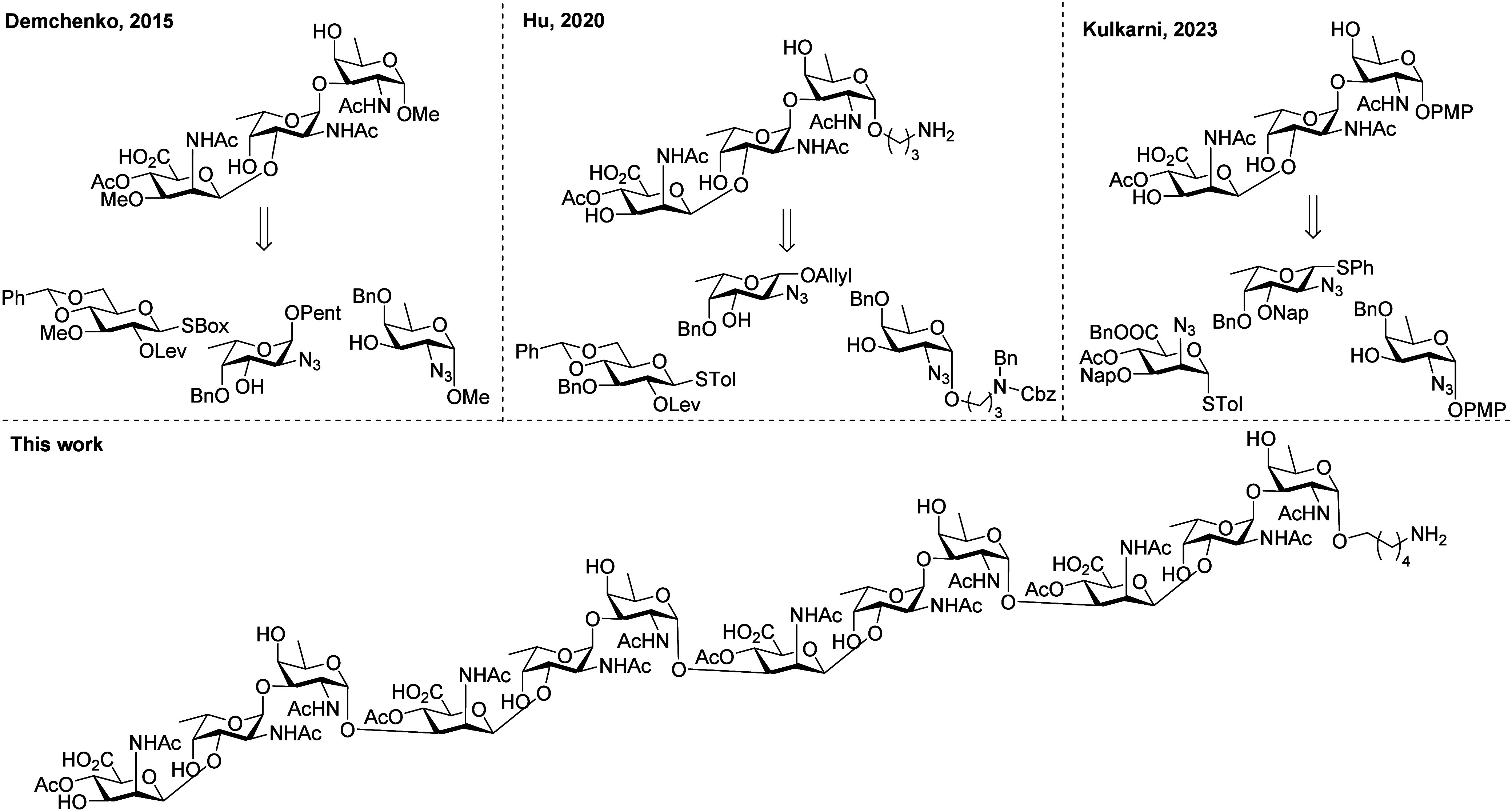
Previously synthesized trisaccharides and a dodecamer were synthesized in this work.

Due to their biological importance, several attempts to synthesize CP8 fragments have been reported over the past years, as summarized in Figure 2. The synthesis of CP8 oligosaccharides is challenging because of the 1,2-*cis* glycosylic linkages, the rare monosaccharides, and many types of functional groups (carboxylates, acetamides, and acetyl esters). The first synthesis of a CP8 oligosaccharide was reported by Demchenko and co-workers in 2015,^27^ who prepared a trisaccharide with methyl groups on both capping ends, which made conjugation and elongation impossible. They synthesized the trisaccharide starting from the nonreducing end, and their approach involved a post-glycosylation-oxidation of the mannose residue at the trisaccharide level, a post-glycosylation inversion of C-2 to generate the mannosamine stereochemistry on a disaccharide, and installation of the *O*-acetyl on the trisaccharide. The glucose donor building block, used as a precursor for the ManNAcA, carried an orthogonal C-2-levulinoyl participating group to guarantee the formation of the desired β-linkage. Later, Demchenko and co-workers presented the synthesis of a protected hexasaccharide, that was assembled in a [2 + 4] glycosylation strategy, because attempts at a [3 + 3] strategy failed.^28^ The final protecting group manipulations however proved ineffective, and the target deprotected hexasaccharide could not be obtained, highlighting the difficulty in synthesizing these complex bacterial glycans. In 2020 Hu and co-workers reported a similar route toward the trisaccharide repeating unit, using similar post-glycosylation modulations to create the β-mannosamine linkage, but they chose to perform the oxidation, inversion at C-2, and acetylation on the trisaccharide stage. They installed a linker on the reducing end of the trisaccharide and showed conjugation to a carrier protein was possible.^29^ In 2023 Kulkarni and co-workers reported the first synthesis of CP8 trisaccharide RU that relied on the use of a mannosaminuronic acid building block. They built the CP8 trissaccharide from the reducing to the nonreducing end and installed orthogonal protecting groups on the capping ends of the trisaccharide to allow for elongation in either direction in the future.^30^

We describe here the synthesis of CP8 structures carrying an *O*-acetyl on C-4 of the ManNAcA residue and their use in structural and epitope mapping studies. Specifically, we report on a set of CP8 oligosaccharides ranging in length from a trisaccharide (1 RU) to a dodecasaccharide (4 RUs). The developed synthetic strategy is designed such that manipulations on large oligosaccharides are minimized. For this purpose, we employed a pre-glycosylation oxidation strategy in which the mannosaminuronic acid carboxylic acid and C-4 acetyl ester were installed on the monosaccharide level. We have equipped the fragments with an orthogonal amine linker which has enabled the conjugation to a carrier protein, to generate glycoconjugate vaccine modalities for immunological studies. Structural studies on the well-defined oligomers provided insight into the 3D-structure of the CP8 fragments and revealed that ManNAcA C-4 acetyl groups play a crucial role in constraining the conformational freedom of the longer fragments, leading to extended structures in which the acetyl esters and acetamide groups form hydrophobic patches along the axis of the oligosaccharides. Our epitope mapping studies have revealed monoclonal antibodies to bind to an extended epitope, and the longer fragments not only bound better to antibodies raised against native CP8 but also raised higher IgG titers following immunization of mice with the glycoconjugates.

## Results and Discussion

### Synthesis

**Scheme 1:**
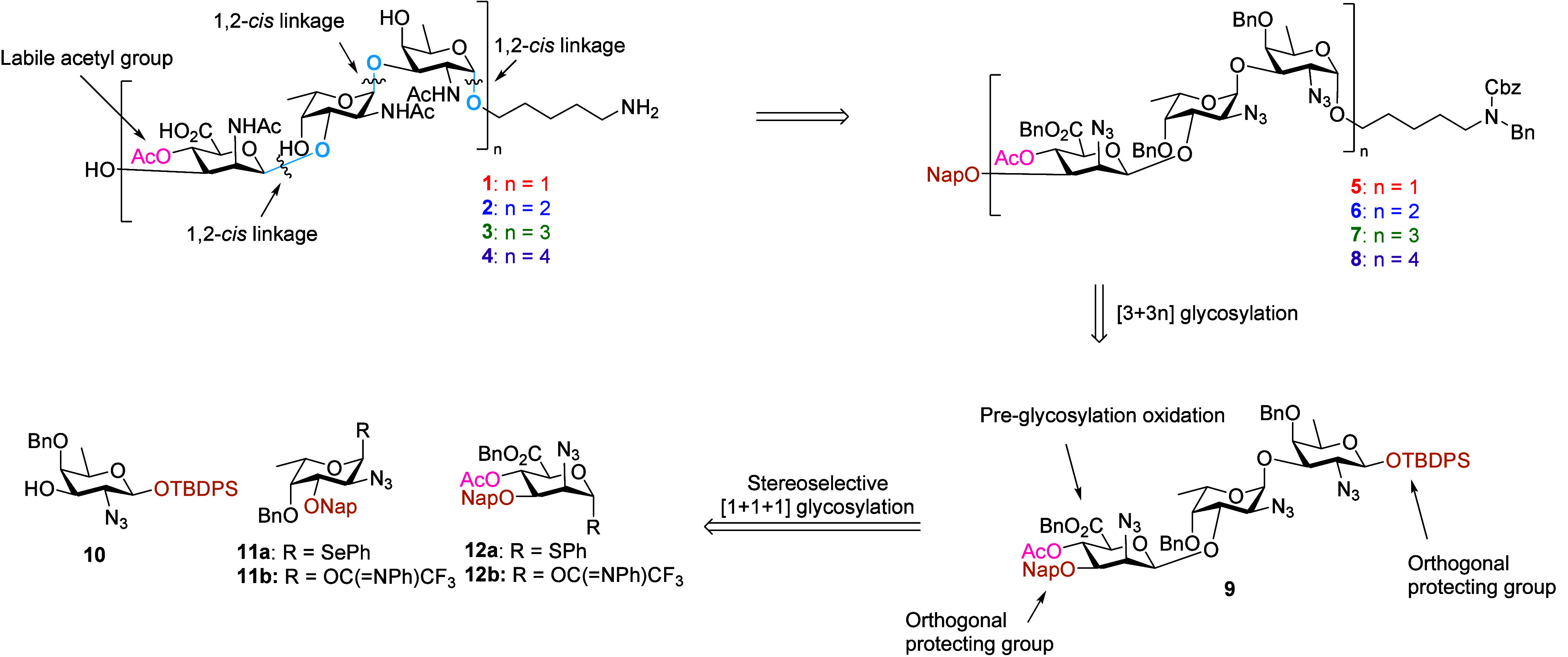
Retrosynthetic Analysis of the Set of Target CP8 Oligosaccharides

Our retrosynthetic analysis of the CP8 fragments is shown in Scheme 1 and comprises the assembly of the oligosaccharides, each equipped with an amino linker for site-selective conjugation purposes, using the central trisaccharide building block **9**. This key synthon incorporates the mannuronic acid’s carboxylic acid functionality and the C-4-*O*-acetyl ester, which precludes challenging modifications later in the synthesis scheme. Especially the execution of multiple oxidations on a complex, partially protected glycan can be arduous.^28,31,32^ The protecting group strategy was designed such that only two steps are required post-glycosylation to unmask all functional groups: transformation of the azides—required as nonparticipating groups in the *cis*-glycosylation reactions—into the corresponding acetamides and hydrogenation of all benzyl-type ethers. The trisaccharide building block **9** further carries a *tert*-butyldiphenylsilyl (TBDPS) on the anomeric position and a 2-methylnaphthyl (Nap) group on the mannosaminuronic acid C-3-OH to allow for orthogonal deprotection and selective elongation at either the reducing or nonreducing end. We choose to build the larger glycans exploiting the reliable stereoselectivity of the 2-azidofucose donor, as we have previously established that 2-azidofucose donors, carrying ether type protecting groups, in combination with weakly nucleophilic alcohol acceptors, such as the 2-azidomannuronic acid C-3-OH, can provide the desired 1,2-*cis*-2-azidofucose linkages with excellent stereoselectivity.^33^ We aimed to prepare trisaccharide **9** from three monosaccharide building blocks: the d-2-azidofucose (FucN_3_) building block **10**, the l-FucN_3_ building block **11**, and the d-2-azidomannuronic acid (ManN_3_A) building block **12**, in a [1 + 1+1] glycosylation approach. Mannuronic acid building blocks are among the most effective donors to install the challenging 1,2-*cis*-mannose type glycosidic linkages, and the use of the ManN_3_A building block thus not only obviates the need for late-stage oxidation reactions but should also streamline the stereoselective assembly of the central trisaccharide **9**.

All required building blocks **10**, **11**, and **12** were synthesized from commercially available starting materials building on routes we previously established,^33^ as detailed in the Supporting Information (SI). The synthesis of the d-FucN_3_ building block was achieved from d-galactose in 14 steps with an overall yield of 12%, while for the l-FucN_3_ donors a route starting from l-fucose was implemented to give **11b** in 20% yield over 11 steps (**11a** over 9 steps and 23%). The d-ManN_3_A building blocks **12a** and **12b** were synthesized from d-mannosamine in 11 (28% overall yield) or 13 steps (37% overall yield), respectively.

We started the construction of the central trisaccharide **9** with the synthesis of the l-FucN_3_-d-FucN_3_ disaccharide **13**. First, the l-FucN_3_ selenophenyl donor **11a** was investigated using *N*-iodosuccinimide (NIS) and trimethylsilyl trifluoromethanesulfonate (TMSOTf) as a promoter in dichloromethane (DCM). Surprisingly, a moderately β-selective glycosylation was found (Table 1, entry 1). While lowering the temperature gave even more of the β-product (entry 2), increasing the temperature led to the formation of more of the desired α-product (entry 3). We next explored the use of imidate donor **11b** with TMSOTf as a promoter in DCM at room temperature. Using these conditions, a highly α-selective glycosylation was achieved, but the disaccharide **13** was formed in low yield (entry 4). Increasing the reaction time (entries 5 and 6) improved the yield only moderately. We then switched the promoter from TMSOTf to *tert*-butyldimethylsilyl trifluoromethanesulfonate (TBSOTf) (entry 7), which led to an improved yield of 98%, while the excellent α-selectivity was maintained.^34^ The markedly different outcome of these latter glycosylations may be explained by the fact that TMSOTf can lead to silylation of the acceptor alcohol, hampering the glycosylation.^35^ The α-linkage was confirmed by ^1^H NMR, with the anomeric proton of the newly formed acetal appearing as a doublet at 5.24 ppm with a coupling constant *J*_H1–H2_ of 2.4 Hz.

**Table 1:**
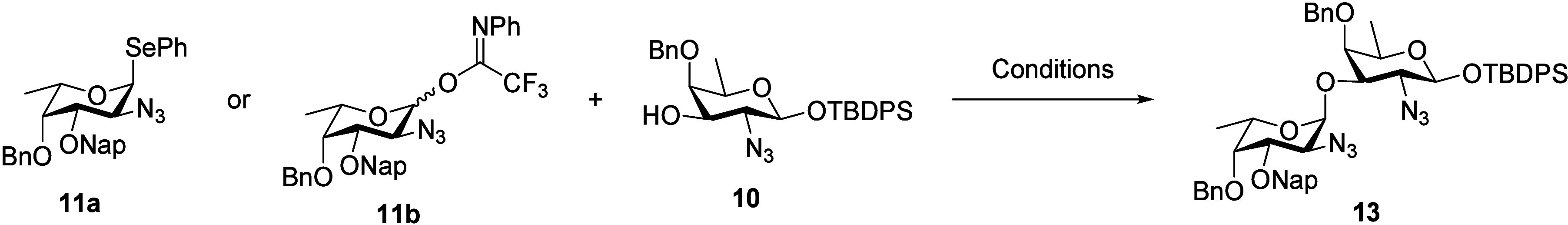
Optimization of Disaccharide Glycosylation^a^

Entry	Donor	Conditions	Temperature (°C)	Time (h)	Yield (%)	α:β^b^
1	**11a**	NIS, TMSOTf, DCM	–78 to −50	1	71	35:65
2	**11a**	NIS, TMSOTf, DCM	–80 to −60	2	51	20:80
3	**11a**	NIS, TMSOTf, DCM	rt	0.5	62	52:48
4	**11b**	TMSOTf, DCM	rt	0.5	11	99:1^c^
5	**11b**	TMSOTf, DCM	rt	1.5	25	99:1^c^
6	**11b**	TMSOTf, DCM	rt	2	36	99:1^c^
7	**11b**	TBSOTf, DCM	rt	0.5	98	95:5

a General conditions: 3 Å mol. sieves, 0.1 M DCM, 0.2 equiv of promoter, 1.5 equiv of NIS, 1.2 equiv of **11a** or 1.3 equiv of **11b**.

b The α:β ratio was determined by NMR of the purified products.

c No β-product was isolated.

**Scheme 2:**
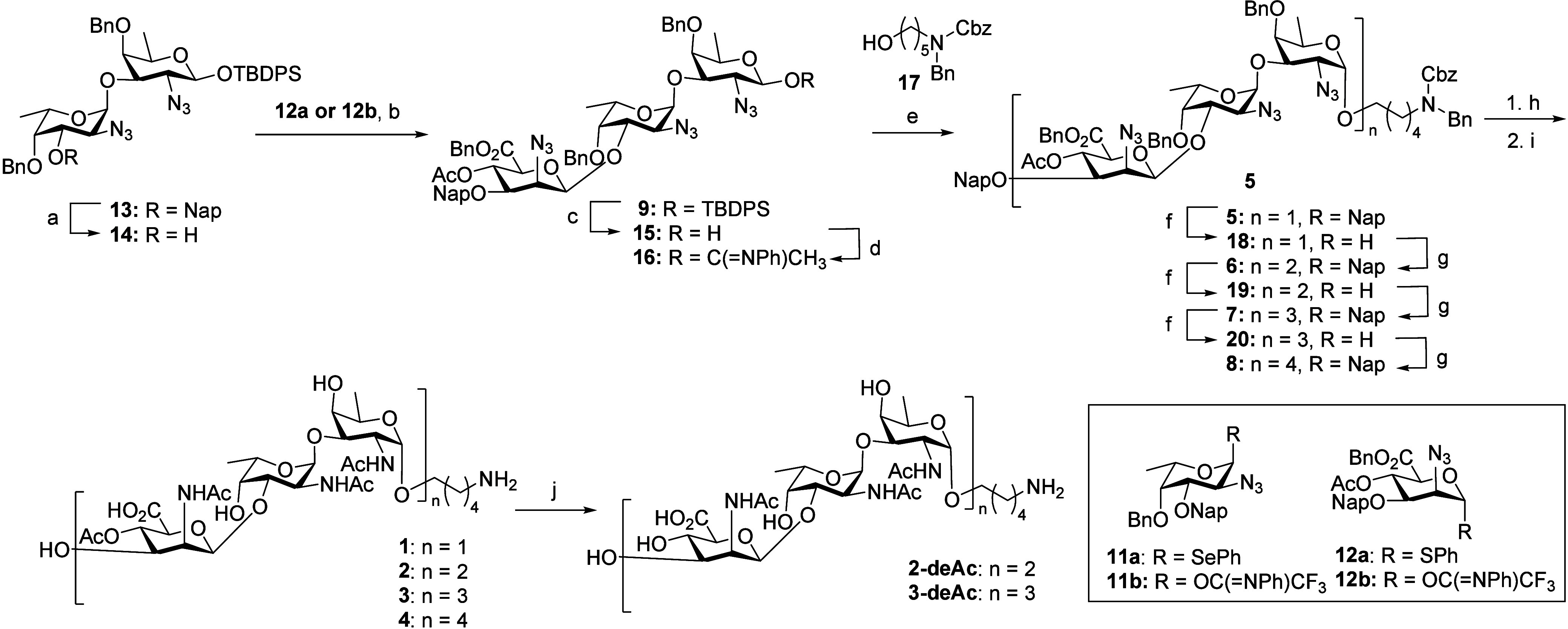
Synthesis of Target Oligosaccharides Reaction conditions: a) DDQ, DCM/H_2_O, 86%, b) **12a**, NIS, TfOH, DCM, −78 °C → −10 °C, 44%, α:β = 25:75 or **12b**, TfOH, DCM, −78 °C → −10 °C, 66%, α:β = 14:86, c) TBAF, AcOH, THF, 0 °C → rt °C, 84%, d) ClC(=NPh)CF_3_, K_2_CO_3_, acetone, 93%, e) TMSI, Ph_3_P=O, DCM/Et_2_O, 83%, α:β = 75:25, f) DDQ, DCM/H_2_O, **18** = 80%, **19** = 54%, **20** = 57%, g) **16**, TBSOTf, DCM, **6** = 87%, **7** = 77%, **8** = 68%, h) Zn, AcOH, Ac_2_O, THF, 50 °C, (i) Pd(OH)_2_/C, AcOH, H_2_, t-BuOH/H_2_O, yield over two steps **1** = 45%, **2** = 37%, **3** = 57%, **4** = 33%, j) 1 M NaOH in H_2_O, **2-deAc** = 41%, **3-deAc** = 46%.

Next the Nap-group in disaccharide **13** was oxidatively cleaved by 2,3-dichloro-5,6-dicyano-1,4-benzoquinone (DDQ) in DCM/H_2_O yielding the disaccharide acceptor **14** in 86% yield as shown in Scheme 2, setting the stage for the [1 + 2] glycosylation to form the central trisaccharide **9**. Using the ManN_3_A thioglycoside **12a** in combination with NIS and triflic acid (TfOH) as a promoter delivered the target compound **9** in relatively poor yield but decent selectivity (44%, α:β = 25:75). Switching to the corresponding imidate donor **12b** the yield was improved to 66% and the α:β ratio increased to 14:86, as shown in Scheme 2. The β-linked trisaccharide **9** could easily be purified by column chromatography, and the β-linkage was confirmed by ^1^H NMR and ^13^C NMR with the β-ManN_3_A anomeric proton and carbon having a CH-coupling constant of *J*_C1–H1_ = 158.8 Hz. The TBDPS group on the anomeric position of the d-FucN_3_ in **9** was removed using *tetra*-butylammonium fluoride (TBAF) buffered by acetic acid (AcOH) in tetrahydrofuran (THF) to give hemiacetal **15** in 84% yield, and this was followed by installation of the *N*-phenyl trifluoroacetimidate^36^ to give key trisaccharide donor **16** in 93% yield. The stereoselective installation of the linker proved challenging because of the relatively high reactivity of the primary alcohol of the alkane linker **17**.^37^ Gratifyingly, it was found that activation of the imidate donor **16** using trimethylsilyl iodide (TMSI) and triphenylphospine oxide (Ph_3_P=O)^38^ led to the desired α-linked product **5** in 93% yield and a α:β ratio of 75:25 (see the SI for optimization of these reaction conditions).

Now the stage was set for the assembly of the larger fragments using the projected [3 + 3*n*] glycosylation strategy. Thus, hexasaccharide **6** was synthesized by unmasking the ManN_3_A C-3-OH by oxidative denaphthylation of **5** with DDQ giving acceptor **18** in 80% yield. The [3 + 3] glycosylation of acceptor **18** and donor **16** using TBSOTf as a promoter at room temperature yielded the target hexamer **6** as a single anomer in 87% yield. The nonasaccharide **7** was synthesized using similar steps, and the nonasaccharide **7** was obtained in the [3 + 6] glycosylation in 78% yield in a highly stereoselective manner. Finally, the dodecasaccharide was synthesized by first transforming nonamer **7** into the corresponding acceptor **20** in 57% yield. The [3 + 9] glycosylation solely afforded the α-anomer of the dodecasaccharide **8** in 68% yield. All of the newly formed α-linkages were confirmed by ^1^H NMR and ^13^C NMR as shown in the SI. Overall, the assembly strategy proved to be very effective, providing the protected CP8 oligomers of unprecedented length, in a highly stereoselective manner.

We then turned to deprotection of the synthetic CP8 oligosaccharides. First, one-pot azide reduction and acetylation using zinc, acetic acid (AcOH), and acetic anhydride (Ac_2_O) afforded the acetamides in yields ranging from 77% to 98%. Previously, we observed lactamization of the mannosaminuronic acid residue upon reduction of the azide,^33^ but by reducing the azide in the presence of AcOH, lactam formation was effectively prevented. Final and global hydrogenation with Pd(OH)_2_/C in *t*-BuOH/H_2_O with AcOH gave target compounds **1**–**4** in yields ranging from 34 to 53% after gel filtration purification, completing the assembly of the set of CP8 oligosaccharides.

### Conjugates and Antibody Binding

**Figure 3:**
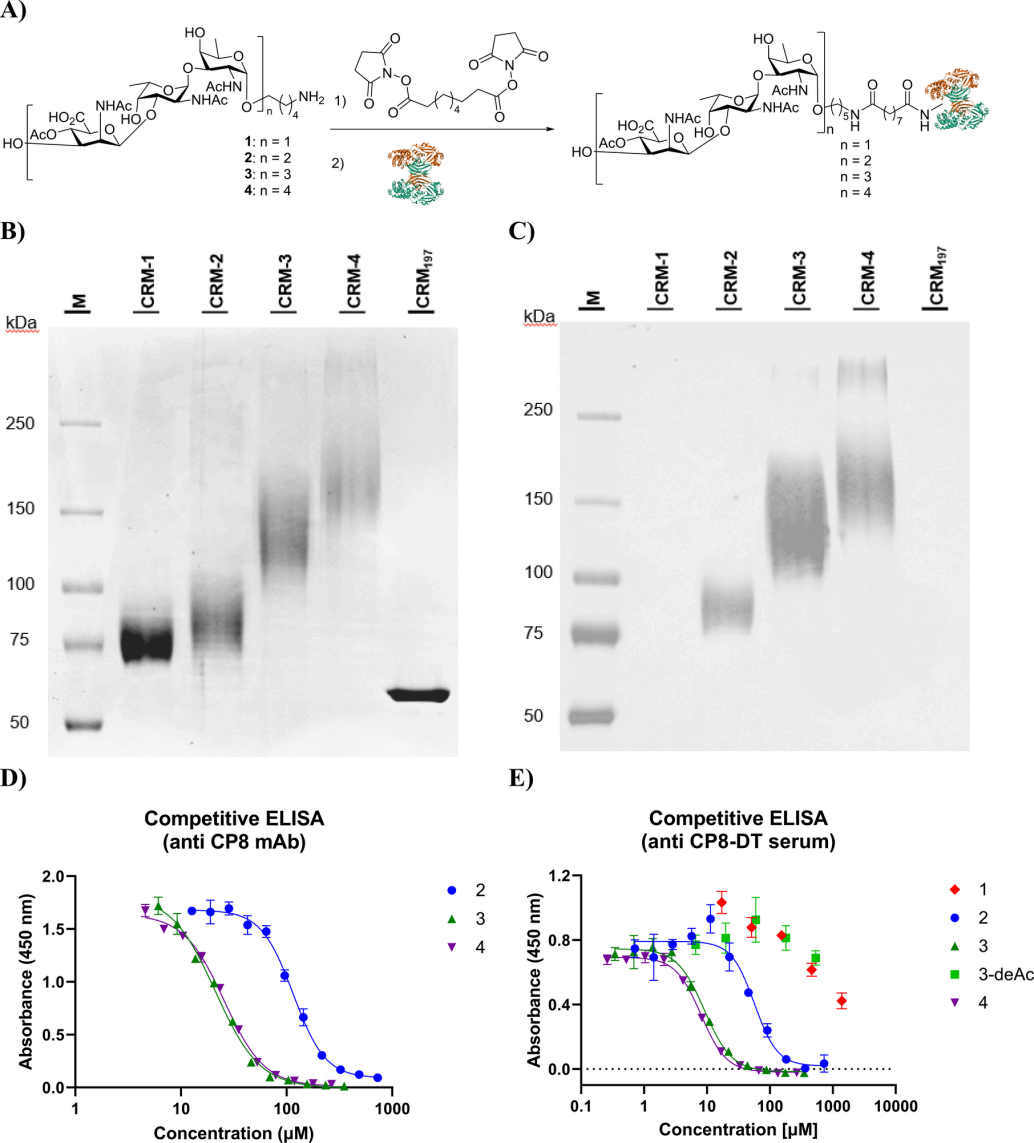
A) Conjugation of the synthetic fragments. 1) Suberic acid bis(*N*-hydroxysuccinimide ester) 30 equiv for **1** and 15 equiv for **2–4** in DMSO/H_2_O 9:1, 2) CRM_197_ in PBS or HEPES 25 nM. B) SDS-page with conjugates **CRM1–4**. C) Western Blot performed with an anti-CP8 mAb showed recognition of **CRM2–4**. BSA-Pel-CRM was included as a negative control. D) Competitive ELISA with anti mAb-CP8. E) Competitive ELISA with polyclonal anti-CP8 serum.

Having the synthetic fragments in hand, we set out to map their binding to monoclonal antibodies, as well as polyclonal serum raised against native CP8, and to generate semisynthetic model vaccines to explore their immunogenic properties. To do so, we first generated a set of conjugates in which the synthetic oligomers were conjugated to Cross-Reactive Material 197 (CRM_197_), which is an oft-used, nontoxic carrier protein, that has been found to adequately raise a T-cell-based immune response and to be safe and efficient in children.^39^ It can be readily modified, exploiting the surface exposed lysine residues, and we therefore first functionalized the synthetic CP8 fragments with a suberic acid cross-linker on the reducing end aminopentyl group (Figure 3A). To optimize loading on the protein we varied the amount of the oligomers (using 10, 20, and 30 equiv) as well as the constitution of the buffer. The generated conjugates were analyzed by SDS-PAGE and MALDI-TOF, to reveal that the amount of oligosaccharide used had a large impact on the loading of the carrier protein and that a HEPES buffer (25 mM) gave superior results with respect to PBS (see the SI for details). Using 30 equiv of the synthetic fragments carrying the activated succinimide suberic acid esters, we assembled **CRM-1**, **CRM-2**, **CRM-3**, and **CRM-4**, having an average of 11 trisaccharide, 8 hexasaccharide, 13 nonasaccharide, and 14 dodecasaccharide moieties per protein, respectively (see Figure 3B).

With the **CRM1–4** conjugates, we first investigated the recognition by monoclonal anti-CP8 antibodies (mAb-CP8) using a Western Blot experiment. As the Western Blot in Figure 3C shows, the trisaccharide conjugate **CRM-1** was poorly recognized, while conjugates **CRM2–4** all bound well to the mAb-CP8, providing a first indication that larger fragments are required to present an effective epitope. To provide more quantitative insight into the binding affinity, a competitive ELISA was performed, using ELISA plates precoated with isolated, natural CP8 polysaccharide (CP8-PS). These showed a clear concentration-dependent competition for nonconjugated hexasaccharide **2**, nonasaccharide **3**, and dodecasaccharide **4**, with no binding being detected for trisaccharide **1** (Figure 3D). Also, the nonasaccharide lacking the ManNAcA-C4-*O*-acetyl esters, **3-deAc**, generated by saponification of nonamer **3** (Scheme 2), could not compete for binding. Binding to the nonasaccharide was significantly stronger than binding to the hexasaccharide and was on par with binding to dodecasaccharide **4**. Apparently, nonamer **9** is large enough to harbor the epitope for mAb-CP8, while hexamer **2** is too short. A competitive ELISA with polyclonal anti-CP8 serum (pAb-CP8) provided a similar picture, with stronger competition being observed in comparison to the competition for binding with the monoclonal antibody for all fragments (Figure 3E). Also in this experiment, trisaccharide **1** and deacetylated **3-deAc** showed relatively poor binding, and nonamer **3** and dodecasaccharide **4** surfaced as the best binders.

### Structural, Conformational, and Interaction Studies

**Figure 4:**
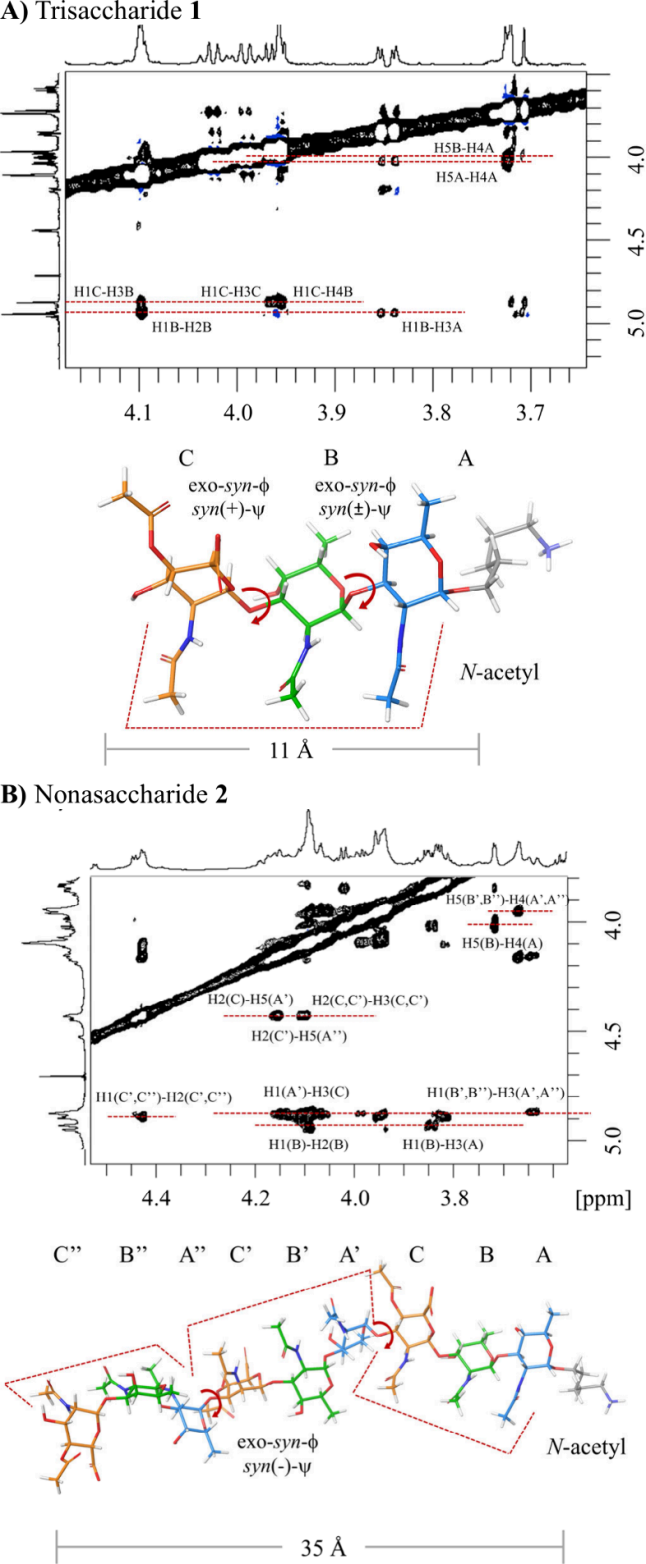
Conformational analysis of trisaccharide **1** and of nonasaccharide **3** as established by NMR and MM calculations. A) Zoom area of the 2D NOESY spectrum of trisaccharide **1** (top) and its main conformation as defined by NOE analysis and MM calculations (bottom). B) Zoom area of the 2D NOESY spectrum of the nonasaccharide **3** (top) and its main conformation as defined by NOE analysis and MM calculations (bottom). Monosaccharidic residues are labeled with a letter code. The main conformation at each glycosidic linkage, the spatial orientation of the acetyl groups, and the average length are reported.

To account for the established structure–activity relationships in the above ELISA experiments, we next set out to probe the structure of the synthetic fragments and map the epitopes present in the oligosaccharides using saturation transfer difference (STD) NMR spectroscopy (STD-NMR). The conformation and dynamics of the synthetic oligomers in solution were investigated using a combination of NMR methodologies (using *J*-couplings and NOE interactions), assisted by computational protocols (MM).^40^ First, trisaccharide **1** was investigated. The resulting intra- and inter-residue NOE cross-peaks allowed us to unequivocally define its major conformation in solution (Figure 4A). Specifically, the analysis of the intraresidual NOE and *J*-couplings established that the three pyranoside residues (A: α-d-FucNAc; B: α-l-FucNAc; C: β-d-ManNAcA) adopt the expected chair conformations (^4^*C*_1_ for residues A and C and ^1^*C*_4_ for residue B) (see the SI for details). Next, the conformation around the glycosidic linkages was analyzed. The simultaneous observation of strong NOEs for the H1(B)–H3(A) and H5(B)–H4(A) proton pairs is indicative of the presence of a major, well-defined exo-*syn*-ϕ/*syn*(±)-ψ conformation around the B–A glycosidic linkage (Figure 4A, left). Fittingly, MM calculations also predicted the predominance of the exo-*syn*-ϕ/*syn*(±)-ψ conformation for this glycosidic linkage. Integration of the observed NOE cross-peaks was used to estimate the ensemble average proton–proton distances (Å), which resulted in the definition of the ψ angle value of ca. ψ = +20 ± 10. In contrast, for the B–C glycosidic linkage, the MM calculations predicted the existence of an equilibrium between a major exo-*syn*-ϕ/*syn*(+)-ψ and a minor exo-*syn*-ϕ/*anti*-ψ geometry. Fittingly, the strong NOEs observed for the H1(C)–H4(B) and H1(C)–H3(B) proton pairs, together with the very low intensity for H1(C) and H2(B) NOE, assessed that the exo-*syn*-ϕ/*syn*(+)-ψ conformation around the C–B linkage is the most populated one in solution. Overall, this leads to the major conformation for trisaccharide **1** shown in Figure 4A (Figure 4A, bottom). Inspection of this 3D structure revealed that alternative conformations around the two glycosidic linkages are prevented because of steric clashes of the acetamide groups of residues A and B with the methyl and carboxylate moieties of residues B and C, respectively. For this major conformation, the average length of the trisaccharide is ∼11 Å, while the three acetamide groups are oriented in the same spatial direction with respect to the plane defined by the sugar rings.

A similar analysis was performed on the nonasaccharide **3**. NOE cross-peaks could be identified that were in full agreement with those observed for trimer **1** (see Figure 4B, top). Nonetheless, the severe NMR signal overlap precluded quantitative integration of the cross-peaks. Thus, a qualitative characterization in terms of weak, medium, and strong intensity signals was used to define the conformations (see SI Figures S17 and S18). In line with the structure for trimer **1**, the exo-*syn*-ϕ/*syn*(±)-ψ conformation around the B–A linkage was found to be the most populated. Similarly, the exo-*syn*-ϕ/*syn*(+)-ψ conformation dominates the C–B glycosidic linkage. The additional A–C glycosidic linkages populate exclusively the exo-*syn*-ϕ/*syn*(−)-ψ conformation, as deduced from the exclusive presence of strong H1(A′)–H3(C) and H5(A′)–H2(C) NOEs. Interestingly, the spatial orientation of the ManNAcA C-4-acetyl and C-2-acetamide groups provides an energy barrier for rotation around the A′–C and A″–C′ glycosidic linkages. As a result, nonasaccharide **3** adopts an extended conformation of ∼35 Å average length, which roughly corresponds to three times the length of the trisaccharide. The negative charges of the carboxylate moieties are at a distance of 15–16 Å from each other. Furthermore, in this structure, the three acetamide groups of each repeating unit (RU) and the ManNAcA acetyl ester of the RU at the reducing end are presented in the same spatial direction, with the trisaccharide RUs being tilted by a dihedral angle close to 90° between two consecutive RUs (Figure 4B, bottom). The proximity of the *N*- and *O*-acetyl methyl groups creates hydrophobic patches that may be important for binding to antibodies.

**Figure 5:**
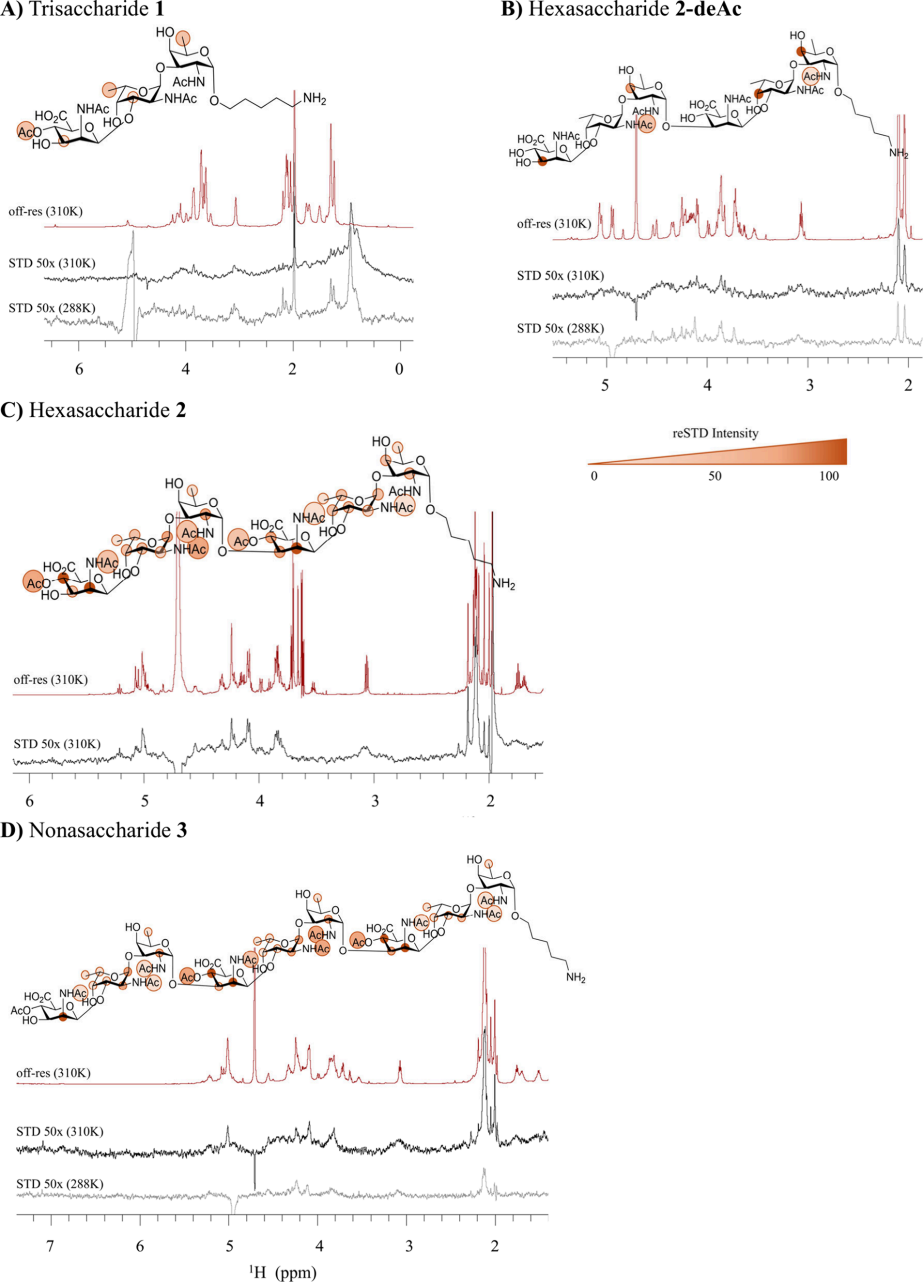
^1^H STD-NMR spectra performed for the complexes of mAb-CP8 and the trisaccharide **1** (A), the deacetylated hexasaccharide **2-deAc** (B), the hexasaccharide **2** (C), and the nonasaccharide **3** (D). Off-resonance spectra (in red) and the corresponding STD-NMR spectra at 310 K (in black) and at 288 K (in gray). The representation of the epitope map disclosed by the analysis of the relative STD-NMR signal intensities for each oligosaccharide is reported as a color legend associated with the STD% values.

To reveal the structural elements that define the optimal binding epitope, the interaction of the saccharides with mAb-CP8 was explored by saturation transfer difference NMR (^1^H STD-NMR) experiments. In particular, the tri-, hexa-, and nonasaccharide **1**, **2**, and **3** as well as the deacetylated hexasaccharide (**2-deAc**, generated by saponification of hexamer **2**, Scheme 2) were tested. For trisaccharide **1**, the resulting NMR spectrum acquired at physiological temperature (310 K) showed no significant STD-NMR signals. At lower temperature (288 K) the STD-NMR signals slightly increased, suggesting the existence of a very weak interaction (Figure 5A). In contrast, the STD-NMR spectrum of hexasaccharide **2** at 310 K revealed clear STD signals (Figure 5C). The deacetylated hexamer **2-deAc** showed only marginal STD-NMR signals, the intensity of which again enhanced upon lowering the temperature (Figure 5B). This result indicates a weak interaction between the mAb and the deacetylated hexasaccharide and thus suggests a key role of the *O*-acetyl group of the mannuronic residue for mAb binding. Consistently, in the absence of the ManNAcA *O*-acetyl, the ManNAcA residues did not significantly contribute to the binding, as deduced from the negligible intensities found for the signals of this residue (compare Figures 5B and 5C). Interestingly, the STD-NMR spectrum for nonasaccharide **3** showed less intense STD signals than the spectrum of hexasaccharide **2** (compare Figures 5C and 5D). Since the success of a STD NMR experiment depends on a fast dissociation rate of the ligand–mAb complex on the NMR relaxation time scale, the observed low intensities of the STD signals found for the nonamer may be explained by the binding that is too strong for this molecule, as revealed in the ELISA assays. Consistent with this hypothesis, the STD-NMR signals became clearer at higher temperature, where dissociation of the ligand from the antibody becomes faster (Figure 5D). Next, the relative STD-NMR signal intensities were used to define the corresponding binding epitopes. In general, a similar STD profile was observed for hexasaccharide **2** and nonasaccharide **3**. The strongest STD-NMR signals were observed for mannuronic acid, the α-L-FucNAc residues, and the corresponding methyl groups of the acetyl esters and acetamide moieties. In particular, the H2 and H4 protons of the ManNAcA residues displayed the strongest STD effects, ranging between 75 and 100% of the maximum STD relative intensity. The *O*-acetyl at the mannuronic residue, the *N*-acetyl and the H1–H2 of the l-FucNAc, together with d-FucNAc H2 displayed relative STD intensities ranging between 50 and 74%. Weaker STD signals were recorded for the H3 and the *N*-acetyl moiety of the ManNAcA, the H3–H5 of the l-FucNAc, and the *N*-acetyl group of the l-FucNAc. Interestingly, marginal STD signals (below 25%) were measured for the methyl groups of the l- and d-FucNAc residues, all along the saccharide chain, as well as for the *N*-acetyl moieties of the reducing end terminal saccharides. However, a comparison of the STD results of the hexa- and nonasaccharide reveals a shift in the main epitope (compare Figures 5B and 5D). For the longer oligosaccharide, the strongest STD signals arose from the central RU, while for the hexasaccharide, the main epitope is formed by the terminal repeating unit at the nonreducing end.

Overall, these data clearly indicate that the mAb recognizes the CP8 oligosaccharides through an extended binding epitope that spans over 2 RUs, and that is mainly defined by the interaction of the acetylated ManNAcA and L-Fuc residues. For the longer nonasaccharide (3 RUs) the central region of the oligosaccharidic chain is in close contact with the antibody binding site, while in the shorter hexasaccharide it engages mostly in binding with the nonreducing end terminal part.

### *In Vivo* Studies

**Figure 6:**
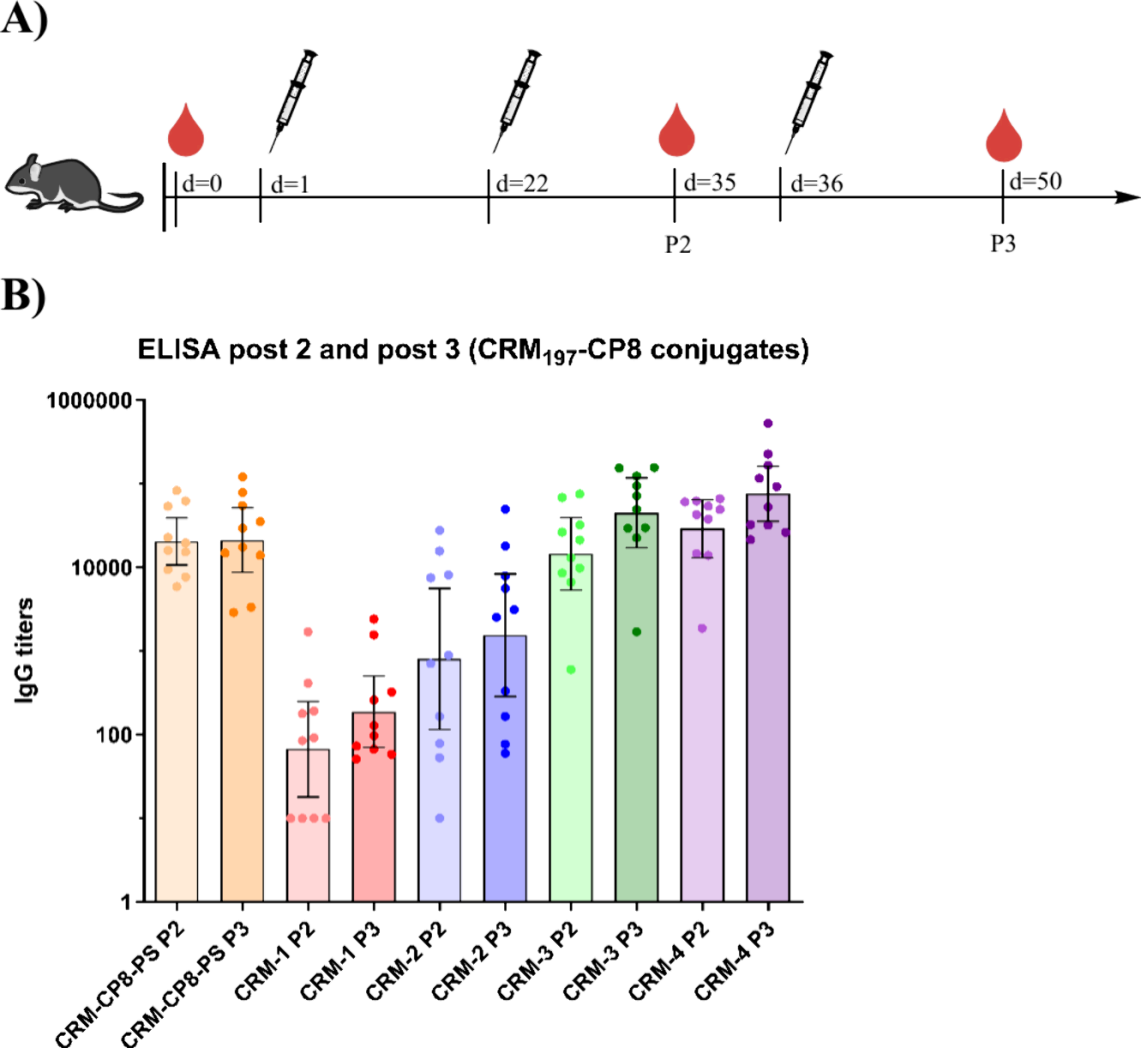
A) Schematic illustration of the *in vivo* study. Injections were performed at day 1, day 22, and day 36, and a bleed was performed at day 0, day 35 (post 2), and day 50 (post 3). B) ELISA post 2 (P2) and post 3 (P3) IgG titers.

Finally, we investigated the immunological properties of the CRM-CP8 conjugates in a mouse immunization study, in which the conjugates (with a dose of 1 μg of carbohydrate per immunization) were injected together with aluminum hydroxide (AlOH, 3 mg/mL) as an adjuvant. Besides the four synthetic CP8 conjugates also a CP8-PS-CRM conjugate was used for comparison. Five groups of 10 mice (5 weeks old) were injected three times, at day 1, 22, and 36, taking a bleed at day 35 (post 2) and day 50 (post 3, the final bleed). The anti-CP8 IgG titers in the collected sera were measured by using ELISAs. As shown in Figure 6, a clear oligosaccharide-length-dependent immune response was observed for the conjugates of the synthetic oligosaccharides. The immunization with the trisaccharide conjugate **CRM-1** led to the lowest anti-CP8 titers, while slightly higher titers were found for the hexasaccharide **CRM-2**. Antibody levels elicited by the conjugate of the shortest oligosaccharides appeared more scattered, as opposed to the longest structures. For the nona- and dodecasaccharide high titers were found with only a small difference between the two fragments in favor of the dodecasaccharide **CRM-4**. The titers from the nona- and dodecasaccharide conjugates compared well with the titers found in the immunization with the natural CP8-PS conjugate. After injections two and three, a small boost was observed for the synthetic conjugates, with the boosting effect being strongest for the shortest, weakest antigens (trisaccharide **1** and hexasaccharide **2**). No boost effect was observed for the CP8-PS conjugate. Overall, these results show that the synthetic oligosaccharides mimic the antigenicity of the full polysaccharide well, if sufficiently long (i.e., three RUs or more) saccharides are used.

## Conclusion

In this work, a convergent strategy for the assembly of synthetic, conjugation-ready *S. aureus* CP8 oligosaccharides comprising multiple repeating units has been developed. By using a pre-glycosylation oxidation strategy to introduce the mannosaminuronic acids in combination with two 2-azidofucose synthons, an effective route to generate the required trisaccharide building block is disclosed. Using an orthogonally protected trisaccharide, a set of CP8 oligosaccharides has been assembled, ranging in length from a tri- to a dodecasaccharide, carrying *O*-acetyl esters at the ManNAcA C4-OH. The developed protecting group strategy has enabled high yielding and stereoselective glycosylation reactions to construct all required 1,2-*cis* linkages. It also allowed for a highly efficient global deprotection scheme, requiring only two transformations and leaving the *O*-acetyl ester unscathed. An aminopentyl linker was installed, which allowed for conjugation to CRM_197_ to construct a set of model conjugate vaccines. The glycoconjugates were evaluated for their binding to mono- and polyclonal antibodies and used in immunization experiments. These revealed a clear length-dependent immune response. While the trisaccharide was found to be too short to bind the antibodies or raise an immune response capable of adequately recognizing the natural polysaccharide, the hexasaccharide bound the antibodies better and the nona- and dodecasaccharide provided optimal epitopes for recognition. The conjugates of the latter oligomers raised a high titer of antibodies recognizing the natural polysaccharide well. Detailed structural studies revealed that the oligosaccharides adopt an extended, almost linear structure, in which all acetyl groups of each trisaccharide repeating unit point in the same direction, generating hydrophobic patches along the periphery of the oligosaccharide chain. These formed important recognition elements in the epitope for the monoclonal antibody. The interaction and immunization studies have revealed the requirements for at least three repeating units to deliver a strong binding epitope.

This study highlights the advantages of larger synthetic oligosaccharides for immunological studies at the molecular level. Because of the challenges associated with the assembly of bacterial oligosaccharides, often oligosaccharides comprising only a single repeating unit are reported. This obviously simplifies the synthesis campaign, but it does bring about the risk of synthesizing a suboptimal frameshift of the repeating unit, and it fails to capture epitopes spanning multiple repeating units. Our work illustrates how progressing insight into glycosylation chemistry, which enables the effective stereoselective construction of difficult glycosidic linkages, alongside the development of ever more effective protecting and functional group manipulations, required to install all of the different functionalities present in bacterial glycans, opens the way to construct longer, fully functional oligosaccharides. These not only enable the conception of synthetic vaccines but also can be used as high value tool compounds to probe bacterial biosynthesis enzymes and investigate (multivalent) interactions with host (immune cell) receptors.

## Supporting Information Available

Elaborated synthetic optimizations and building block synthesis, experimental procedures, characterization data, NMR spectra, antibody binding, and *in vivo* study data and structural characterization data (PDF)
