# Supplementary material for: Long, Synthetic Staphylococcus aureus Type 8 Capsular Oligosaccharides Reveal Structural Epitopes for Effective Immune Recognition
Source: J Am Chem Soc. 2025 Jan 10;147(3):2829–40. doi: 10.1021/jacs.4c16118 (PMC11760181; doi:10.1021/jacs.4c16118)

Supporting information

Long, synthetic *Staphylococcus aureus* type 8 capsular oligosaccharides reveal structural epitopes for effective immune recognition

Kitt Emilie Østerlid[1], Charlotte Sorieul[1], Luca Unione[3,4], Sizhe Li[1], Cristian García-Sepúlveda[3], Filippo Carboni[2], Linda Del Bino[2], Francesca Berni[1], Ana Arda[3,4], Herman S. Overkleeft[1], Gijsbert A. van der Marel[1], Maria Rosaria Romano[2], Jesús Jiménez-Barbero[3,4,5,6], Roberto Adamo[2], Jeroen D. C. Codée[1,*]

[1] Leiden Institute of Chemistry, Leiden University, Einsteinweg 55, 2333 CC Leiden, The Netherlands

[2] GSK Siena, Via Fiorentina, 1, 53100 Siena SI, Italy

[3] Center for Cooperative Research in Biosciences (CIC bioGUNE), Basque Research and Technology Alliance (BRTA), 48160 Derio, Bizkaia, Spain.

[4] Ikerbasque, Basque Foundation for Science, Bilbao, Spain.

[5] Department of Organic & Inorganic Chemistry, Faculty of Science and Technology, University of the Basque Country, EHU-UPV, 48940 Leioa, Bizkaia, Spain.

[6] Centro de Investigacion Biomedica En Red de Enfermedades Respiratorias, 28029 Madrid, Spain.

Table of Contents

Description of the synthesis of the building blocks 3
Optimization of the linker installation 5
General experimental procedures 7
Synthesis of the D-fucose building block 8
Synthesis of the L-fucose building block 11
Synthesis of the D-mannose building block 14
Synthesis of the trisaccharide 18
Synthesis of Longer fragments 22
Preparation of S. aureus type 8 conjugates 28
MALDI-TOF MS 29
Protocol for Western Blot (using mAb and pAb) 33
Protocol for competitive ELISA with mAb 34
Protocol for competitive ELISA with pAb 35
Structural conformation 35
Ligands-Antibody interaction studies 41

Characterization of CP8-conjugated used for *in vivo* studies 43
Immunizations 44
ELISA protocol for *in vivo* studies 44
References 45
Spectra 46

Description of the synthesis of the building blocks

All the building blocks were synthesized from commercially available starting materials. The synthesis of the D-FucN$_3$ building block (Scheme S1A) commenced with D-galactose following a reported procedure.[1] In a 5-step reaction sequence in which the required galactose-to-fucose deoxygenation was achieved by iodination of the C-6 position and radical reduction of the primary iodide, the acetylated D-fucose **S1** was obtained in 54% yield from D-galactose on large scale. Next, anomeric bromination followed by elimination using zinc and NH_4Cl gave fucal **S2** in 48% yield. A regio- and stereoselective azidophenylselenation using the more soluble azidotrimethylsilane (TMSN$_3$) instead of NaN$_3$ together with (bisacetoxyiodo)benzene (BAIB) and diphenyldiselenide ((SePh)$_2$) by a procedure develop by Nifantiev and co-workers[2] followed by saponification afforded **S3** in 67% yield. Now, the C-3-OH was selectively naphthylated via the intermediate tin-acetal,[3] allowing for benzylation of the free C-4-OH giving **S5**. The anomeric phenylselenyl group was hydrolyzed using *N*-iodosuccinimide (NIS) in acetone/water and the lactol, was then silylated using tert-butyldiphenylsilyl, chloride (TBDPS-Cl) providing **S7** in 96% yield. Lastly, the Nap-group was oxidative cleaved with 2,3-dichloro-5,6-dicyano-1,4-benzoquinone (DDQ) in 90% yield to give acceptor **10**.

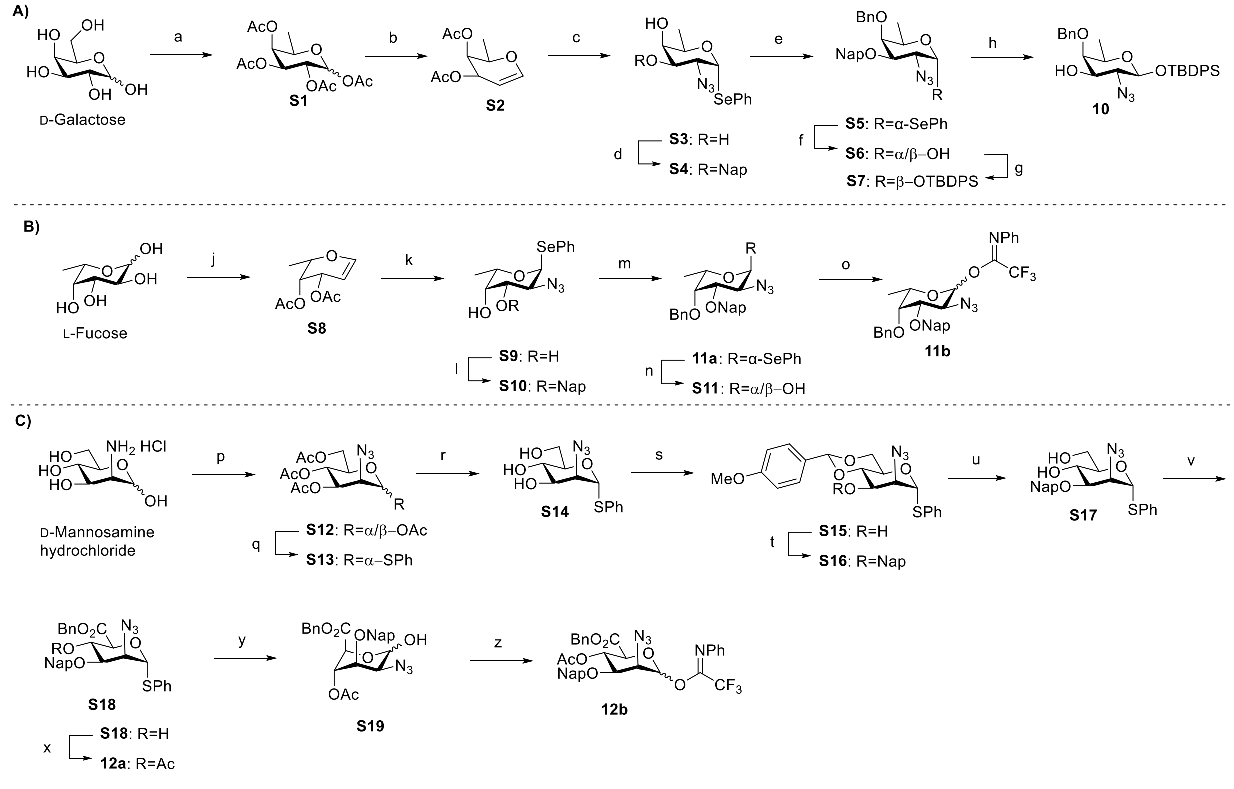

Scheme S1: Synthesis of the building blocks. *Reaction conditions:* A) a) i) conc. H_2SO_4, acetone, ii) PPh_3, I_2, imidazole, toluene/MeCN, 90 °C, iii) VA-044, aq. H_3PO_2, Et_3N, i-PrOH, 60 °C, iv) 80% aq. AcOH, 90 °C, v)Ac_2O, pyridine, 0 °C → rt, 5 steps 54%, b) i) HBr in AcOH 33%, DMC, 0 °C → rt, ii) Zn, NH_4Cl, EtOAc, 60 °C, 2 steps 48%, c) i) $(PhSe)_2$, BAIB, TMSN$_3$, DCM, -20 °C → -30 °C, ii) NaOMe, MeOH, 2 steps 67%, d) Bu_2SnO, toluene, 140 °C then Bu_4NBr, CsF, NapBr, 120 °C, 93%, e) BnBr, NaH, DMF, 0 °C → rt, 88%, f) NIS, acetone/H_2O, 0 °C, 99%, g) TBDPSCl, imidazole, DMAP, DCM, 0 °C → rt, 96%, h) DDQ, DCM/H_2O, 90%, B) j i) Ac_2O, pyridine, 0 °C → rt, ii) HBr in AcOH 33%, DMC, 0 °C, iii) Zn, NH_4Cl, EtOAc, 60 °C, 3 steps 47%, k) i) $(PhSe)_2$, BAIB, TMSN$_3$, DCM, -20 °C → -30 °C, ii) NaOMe, MeOH, 2 steps 64%, l) Bu_2SnO, toluene, 140 °C then Bu_4NBr, CsF, NapBr, 120 °C, 89%, m) BnBr, NaH, DMF, 0 °C → rt, 86%, n) NIS, acetone/H_2O, 0 °C, 93%, o) ClC(=NPh)CF_3, K_2CO_3, acetone, 95%, C) p) i) Tf_2O, NaN_3, $CuSO_4{\cdot}5\ H_2O$, pyridine, 0 °C ii) Ac_2O, 0 °C → rt, 2 steps 98%, q) SHPh, $BF_3{\cdot}Et_2O$, DCM, 0 °C → rt, 88%, r) NaOMe, MeOH, 90%, s) p-MeO-PhCH(OMe)$_2$, CSA, MeCN, 300 mbar, 50 °C, 88%, t) NapBr, NaH, DMF, 0 °C → rt, 93%, u) CSA, MeOH, 87%, v) i) TEMPO, BAIB, AcOH, DCM/t-BuOH/H_2O, 0 °C → 4 °C, ii) BnBr, K_2CO_3, DMF, 2 steps 74%, x) Ac_2O, DMAP, pyridine, 0 °C, 90%, y) NIS, TFA, DCM, 0 °C then Et_3N, 80%, z) ClC(=NPh)CF_3, K_2CO_3, acetone, 95%.

The same approach was implemented for the L-FucN$_3$ building block (Scheme S1B), however now starting from commercially available L-fucose. Per-acetylation followed by bromination and elimination gave fucal **S8** in 47% yield. Azidoselenation followed by saponification gave **S9** in 64%. Selective naphthylation of the C-3-OH followed by benzylation of C-4-OH gave **11a** in 86% yield. Hydrolysis of the anomeric phenylselenyl and installation of the *N*-phenyl trifluoroacetimidate[4] functionality delivered donor **11b** in excellent yield.

The D-ManAN$_3$ was obtained from D-mannosamine hydrochloride (Scheme S1C) by an azidotranfer with freshly prepared triflic azide (TfN$_3$) followed by an one-pot acetylation[5] giving **S12** in 98% yield. Pyridine was chosen as solvent, thus enabling an in-situ acetylation to avoid formation of the glucose epimer side-product, which have been reported previously.[6] Next, synthesis to compound **S14** followed a literature procudre,[7] by first installation of a thiophenyl group to provide to **S13** in 88% yield. Saponification of the remaining three acetyl esters was followed by the installation of a *p*-methoxybenzylidene to mask the C-4 and C6-hydroxyl groups. Protection of the remaining C-3-OH as the Nap-ether delivered **S15**. The *p*-methoxybenzylidene was removed with CSA to enable the regio- and chemoselective oxidation of the primary alcohol using 2,2,6,6-tetramethyl-1-piperidinyloxy (TEMPO) and BAIB.[8,9] Alkylation of the newly formed carboxylic acid as the corresponding benzyl ester delivered **S18** in 74% overall yield. The remaining C-4-OH was acetylated in 90% yield giving **12a**. NMR analysis of this building block revealed a ring flip from a $^4C_1$ to a $^1C_4$ conformation.

The removal of thiophenyl from **12b** proved to be much more difficult than first anticipated and different attempts were investigated to optimize this transformation. First, hydrolysis using *N*-bromosuccinimide (NBS) in acetone/water resulted in low yields due to oxidation of the thiophenyl group to give **12c** (Table S1, Entry 1-2). A procedure using NIS and trifluoroacetic acid (TFA) in dichloromethane (DMC) and water did not lead to any reaction (Entry 3), even when an excess of the reagents was used (Entry 4). By preforming this reaction under anhydrous conditions the desired product was obtained after quenching with sat. aq. $Na_2S_2O_3$ (Entry 5), but an unknown impurity was formed which could not be removed during purification. Unfortunately, the nature of the impurity, revealed in the NMR spectrum could not be identified. Neither prolonging the reaction time (Entry 6) or quenching with piperidine before adding sat. aq. $Na_2S_2O_3$ (Entry 7) improved the outcome. Using NBS and trimethylsilyl trifluoromethanesulfonate (TMSOTf) in DCM/water (Entry 8) also provided an impure product. Finally, it was found that using 1.5 equiv. NIS and 1 equiv. TFA in DCM (Entry 9) under anhydrous conditions and quenching with Et_3N before adding sat. aq. $Na_2S_2O_3$ yielded the desired product in 80% yield, however several column chromatography purifications were needed. After obtaining the hemiacetal **S19** the *N*-phenyl trifluoroacetimidate donor was installed yielding **12b** in 95% yield and the ring was found to flip back to a $^4C_1$ conformation as judged by NMR.

Table S1: Optimization of the thiophenyl removal.

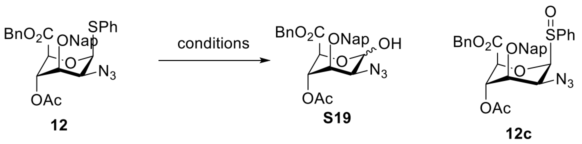

Entry	Conditions	Temperature (°C)	Time (h)	Yield (%)	Notes
1	NBS, acetone/H_2O 10:1	rt	1	48	33 % of **12c**
2	NBS, acetone/H_2O 10:1	0 → rt	0.75	43	42 % of **12c**
3	NIS (1.1 equiv.), TFA (1.1 equiv.) DCM/H_2O 10:1	0	--	--	No reaction
4	NIS (2.5 equiv.), TFA (1.1 equiv.) DCM/H_2O 10:1	0	--	--	No reaction
5	NIS (2 equiv.), TFA (2 equiv.) DCM	0	2	77 [(a)]	Unknown impurity
6	NIS (2 equiv.), TFA (2 equiv.) DCM	0	3	90 [(a)]	Unknown impurity
7	NIS (1.1 equiv.), TFA (1.1 equiv.) DCM, then piperidine	0	4	59 [(a)]	Unknown impurity
8	NBS, TMSOTf, DCM/H_2O 20:1	0	0.75	87 [(a)]	Unknown impurity
9	NIS (1.1 equiv.), TFA (1.1 equiv.) DCM, then Et_3N	0	1	80	

[(a)] Impure product.

Optimization of the linker installation

The stereoselective installation of the linker proved challenging because of the relatively high reactivity of the primary alcohol of the alkane linker **17**. First, installation of the linker was investigated using monosaccharide donor **S20**. It was found that use of the phenylselenyl donor in combination with NIS and TMSOTf mainly gave the *β*-product (Table S2, Entry 1-3). Gratifyingly, activation of the corresponding imidate donor using trimethylsilyl iodide (TMSI) and triphenylphospine oxide (Ph_3PO) did lead to the desired *α*-linked product (entry 4) and by changing the solvent to a 1:1 mixture of DCM/Et_2O the selectivity improved (Entry 5).[10]

Table S2: Investigation of the linker installation.

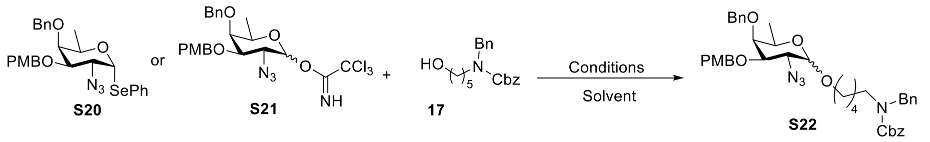

Entry	Donor	Conditions	Solvent	Temperature (°C)	Time (h)	Yield (%)	*α*:*β* [(a)]
1	**S20**	TMSOTf, NIS	DCM/Et_2O 1:1	-40 to -20	1.5	78	10:90
2	**S20**	TMSOTf, NIS	DCM/Et_2O 1:1	-40 to rt	19	64	6:94
3	**S20**	TMSOTf, NIS	DCM	rt	2	64	22:68
4	**S21**	Ph_3PO, TMSI	DCM	rt	23	96	55:45
5	**21**	Ph_3PO, TMSI	DCM/Et_2O 1:1	rt	20	98	81:19

General conditions: 3 A mole sieves, 0.1 M solvent, either 0.2 equiv. promoter and 1.5 equiv. NIS or 1 eq TMSI and 6 equiv. PhP_3O. [(a)] The *α*:*β* ratio was determined by NMR of the purified products.

In another attempt to obtain better α-selectivity the reactivity of the linker alcohol was modified by use of difluorinated alcohol **S24** as seen in Table S3. Placing two fluorine atoms close to the hydroxy group of the linker precursor lowers the nucleophilicity of the alcohol group, further improving the stereoselectivity. The linker was synthesized following a procedure of Seeberger and co-workers.[11] Using **S23** as a nucleophile, high α-selectivity was found (Table S3, Entry 1), especially with the TMSI/Ph_3PO system (Entry 2-3), although the yields of these glycosylations diminished. Overall the use of the non-fluorinated linker appeared to be more effective, cheaper and easier to prepare and therefore the synthesis was continued with non-fluorinated linker **17**.

Table S3: Investigation of the linker installation.

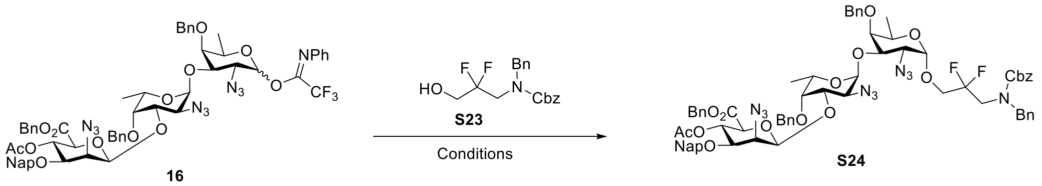

Entry	Conditions	Solvent	Time (h)	Yield (%)	α:β [(a)]
1	TMSOTf	DCM	0.5	86	64:34
2	TMSI, Ph_3P=O	DCM	22	51	83:17
3	TMSI, Ph_3P=O	DCM/Et_2O 1:1	22	46	87:13

General conditions: 3 A mole sieves, 0.1 M solvent, either 0.2 equiv. TMSOTf or 1 equiv. TMSI and 6 equiv. PhP_3O. [(a)] The α:β ratio was determined by NMR of the purified products.

General experimental procedures

All reagents were of commercial grade and used as received unless otherwise noted. All moisture sensitive reactions were performed under an argon or nitrogen (N_2) atmosphere. Dried solvents (DCM, DMF, THF, toluene, Et_2O) were stored over flame-dried 3 or 4Å molecular sieves. Reactions were monitored by thin layer chromatography (TLC) analysis conducted with Merck aluminum sheets with 0.20 mm of silica gel 60. The plates were detected by UV (254 nm) and were applicable by spraying with 20% sulfuric acid in EtOH or with a solution of $(NH_4)_6Mo_7O_{24}\cdot 4H_2O$ (25 g/L) and $(NH_4)_4Ce(SO_4)_4\cdot 2H_2O$ (10 g/L) in 10% sulfuric acid (aq.) followed by charring at ~150 °C. Flash column chromatography was performed with silica gel (40-63μm). Size-exclusion chromatography was carried out using SephadexTM (LH-20, GE Healthcare Life Sciences) by isocratic elution with DCM/MeOH (1:1, v:v). High-resolution mass spectra were recorded on a Thermo Finigan LTQ Orbitrap mass spectrometer equipped with an electrospray ion source in positive mode (source voltage 3.5 kV, sheath gas flow 10, capillary temperature 275 ºC) with resolution R=60.000 at m/z=400 (mass range 150-4000). ^1H and ^{13}C spectra were recorded on a Bruker AV-400 (400 and 101 MHz respectively), Bruker AV-500 (500 and 126 MHz respectively), Bruker AV-600 (600 and 151 MHz respectively), Bruker AV-850 (800 and 214 MHz respectively) or a Bruker AV-1200 (1200 and 302 MHz respectively). Chemical shifts (δ) are given in ppm relative to the residual signal of the deuterated solvent (^1H-NMR: 7.26 ppm for $CDCl_3$, 3.31 ppm for MeOD, 1.94 for $CNCD_3$ or 4.79 for D_2O. ^{13}C-NMR: 77.16 ppm for $CDCl_3$, 49.00 ppm for MeOD, 1.32 for $CNCD_3$). Coupling constants (J) are given in Hz. All ^{13}C spectra are proton decoupled. NMR peak assignments were made using COSY and HSQC experiments, where applicable, HMBC and GATED experiments were used to further elucidate the structure. The anomeric product ratios were analyzed through integration of proton NMR signals.

General experimental for deprotection of the 2-methylnaphthyl group

The fully protected CP8-OS (1 equiv.) was dissolved in DCM/H_2O (0.1 M) and added DDQ (2 equiv.). The reaction was stirred under N_2 at rt until TLC showed full conversion (~4-6 h). The reaction was quenched with $Na_2S_2O_3$ (aq., sat.) and diluted in EtOAc and extracted (x3). The combined organic layers were washed with $NaHCO_3$ (sat. aq.; x4) and brine (x1), dried over Na_2SO_4, filtered and concentrated *in vacuo*. Column chromatography gave the wanted product.

General glycosylation of [3+3], [3+6] and [3+9]

The trisaccharide donor **16** (1.3 equiv.) and the acceptor (1 equiv.) was co-evaporated with toluene (3x), dissolved in dry DCM (0.1 M), added 3Å molecular sieves at rt and stirred for 30 min at rt. TBSOTf (0.2 equiv.) was added at rt and the reaction was stirred at rt under argon until TLC showed full conversion (~30 min). The reaction was quenched with Et_3N, dissolved in EtOAc, washed with $NaHCO_3$ (sat. aq.; x1) and brine (x1), dried over Na_2SO_4 and concentrated. Purification by column chromatography and/or size exclusion gave the wanted product.

General deprotection of 5, 6, 7 and 8

Protected CP8-OS was dissolved in dry, distilled THF (3 mL) and added Zn (300 equiv.), AcOH (1 mL) and Ac_2O (0.5 mL). The reaction was stirred at 50 ºC overnight until TLC showed full conversion. The solution was filtered, concentrated *in vacuo* and co-evaporated with toluene (x3). Column chromatography (DCM/MeOH 98:2 → 95:5) and/or size exclusion gave the wanted product. The acetamide-OS was dissolved in *t*-BuOH (1.5 mL) and added AcOH (1 mL, 0.1 mL in 100 mL MiliQ). Another 1 mL *t*-BuOH was added to dissolve the compound. The solution was birched with argon for 20 min and then added $Pd(OH)_2/C$ (catalytic amount). The reaction was again birched with argon for 5 minutes before the atmosphere was changed for H_2. The mixture was stirred for under H_2 atmosphere

for three days or until completion by NMR was detected. The mixture was filtered over a Whatman filter and lyophilized. Purification by a HW40 column with NH_4OAc followed by lyophilization gave the wanted product.

Synthesis of the D-fucose building block

1,2,3,4-tetra-*O*-acetyl-D-fucopyranose (S1)

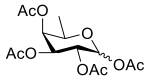

Acetone (1200 mL) was cooled to 0 ºC and slowly dropwise added conc. H_2SO_4 (40 mL). D-Galactose (50 g, 277.5 mmol) was added portion wise and the reaction was allowed to warm to rt and stirred for 7 h until TLC (Pent/EtOAc, 1:1) showed full conversion. The now yellow solution was cooled to 0 ºC and neutralized with $NaHCO_3$ (sat. aq.) until pH~8-9. The acetone was evaporated and the aqueous phase was extracted with EtOAc (x3). The combined organic phases were washed with brine (x1), dried over Na_2SO_4, filtered and concentrated *in vacuo*. The crude product (62.04 g, 238.3 mmol) was dissolved in toluene/MeCN (2:1, 700 mL). First PPh_3 (118.66 g, 524.4 mmol, 2.2 equiv.) and imidazole (71.39 g, 1050 mmol, 4.4 equiv.) were added followed by portion wise addition of I_2 (90.74 g, 357.5 mmol, 1.5 equiv.). The reaction was heated to 90 ºC and stirred for 24 h until TLC (Pent/EtOAc 1:1) showed full conversion. After cooling to rt, the solvents were evaporated and the residue was dissolved in EtOAc, washed with $Na_2S_2O_3$ (aq., sat., x2), H_2O (x2) and brine (x1), dried over Na_2SO_4, filtered and concentrated *in vacuo*. To the crude product in *i*-PrOH (700 mL) were added Et_3N (199 mL, 1450 mmol, 6 equiv.) and aq. H_3PO_2 (50%, 84 mL, 953.2 mmol, 4 equiv.) and the mixture was stirred under N_2 for 30 min. 2,2'-azobis[2-(2-imidazolin-2-yl)propane] dihydrochloride (VA-044, 23.11 g, 71.49 mmol, 0.3 equiv.) was added at rt and the reaction was heated to 80 ºC and stirred under N_2 for 1 h until TLC (Pent/EtOAc 4:1) showed full conversion. The solvent was evaporated and residue was dissolved in EtOAc, washed with NH_4Cl (sat. aq.; x1) and brine (x1), dried over Na_2SO_4, filtered and concentrated *in vacuo*. The crude residue was dissolved in 80% aq. AcOH (600 mL) and stirred at 90 ºC for 18 h until TLC (Pent/EtOAc 4:1) showed full conversion. The solvents were evaporated and the residue was co-evaporated with toluene (x4). The residue was dissolved in pyridine (700 mL) and cooled to 0 ºC. Ac_2O (400 mL) was added and the reaction was slowly allowed to warm to rt and stirred for 18 h until TLC (Pent/EtOAc 4:1) showed full conversion. The solvents were evaporated and the residue wash dissolved in EtOAc, washed with 1 M HCl (x2), sat. aq. $NaHCO_3$ (sat. aq.; x3) and brine (x1), dried over Na_2SO_4, filtered and concentrated *in vacuo*. Column chromatography (Pentane/EtOAc 85:15 → 70:30) gave **S1** in 54% yield (49.42 g, 149 mmol) in a *α*/*β* ratio = 0.8:1. **^{1}H NMR (400 MHz, $CDCl_3$)** δ 6.34 (s, 1H, *α*-H-1), 5.68 (d, *J* = 8.3 Hz, 1H, *β-H-1*), 5.33 (t, *J* = 1.4 Hz, 2H, *α*-H-4, *α*-H-3), 5.33 – 5.32 (m, 1H, *α*-H-2), 5.32 – 5.29 (m, 1H, *β*-H-4), 5.27 (dd, *J* = 3.5, 1.1 Hz, 1H, *β*-H-2), 5.07 (dd, *J* = 10.4, 3.4 Hz, 1H, *β*-H-3), 4.30 – 4.23 (q, *J* = 6,5 Hz, 1H, *α*-H-5), 3.95 (*J* = 6.5 Hz, 1H, *β*-H-5), 2.19 (s, 3H, COC*H₃*), 2.18 (s, 3H, COC*H₃*), 2.14 (s, 3H, COC*H₃*), 2.11 (s, 3H, COC*H₃*), 2.04 (s, 3H, COC*H₃*), 2.01 (s, 3H, COC*H₃*), 2.00 (s, 3H, COC*H₃*), 1.99 (s, 3H, COC*H₃*), 1.22 (d, *J* = 6.4 Hz, 3H, *β-H-6*), 1.15 (d, *J* = 6.5 Hz, 3H, α-*H-6*). **^{13}C NMR (101 MHz, $CDCl_3$)** δ 170.70 (C=O), 170.68 (C=O), 170.36 (C=O), 170.20 (C=O), 170.11 (C=O), 169.63 (C=O), 169.33 (C=O), 169.32 (C=O), 92.31 (*β*-C-1), 90.09 (*α*-C-1), 71.39 (*β*-C-3), 70.71 (*α*-C-3/ *β*-C-4/ *α*-C-4), 70.39 (*β*-C-5), 70.05 (*β*-C-2), 68.03 (*α*-C-3/ *β*-C-4/ *α*-C-4), 67.96 (*α*-C-2), 67.42 (*α*-C-5), 66.59 (*α*-C-3/ *β*-C-4/ *α*-C-4), 21.08 (CO*C*H₃), 21.00 (CO*C*H₃), 20.83 (CO*C*H₃), 20.80 (CO*C*H₃), 20.77 (CO*C*H₃), 20.73 (CO*C*H₃), 16.07 (C-6), 16.06 (C-6). **HRMS**: $[M+Na]^+$ calculated for $C_{14}H_{20}O_9Na$: 355.10050; found 355.09974

3,4-di-*O*-acetyl-D-fucal (S2)

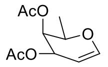

S1 (17.83 g, 53.7 mmol) was dissolved in DCM (215 mL, 0.25 M), cooled to 0 ºC and added HBr in AcOH (33 wt%, 14.6 mL, 80.55 mmol, 1.5 equiv.) using a dropping funnel. The reaction was stirred at 0 ºC under N_2 for 2 h until TLC (Pent/EtOAc 4:1) showed full conversion. The solution was poured over ice and stirred until the ice was molten. The aqueous phase was extracted with DCM (x3) and the combined organic phases were washed with H_2O (x1) and brine (x1), dried over Na_2SO_4, filtered and concentrated *in vacuo*. The residue was co-evaporated with toluene (x3) and used immediately without any further purification. The crude product (17.51 g, 49.74 mmol) was dissolved in EtOAc (166 mL, 0.3 M) and Zn (22.77 g, 348.2 mmol, 7 equiv.) and NH_4Cl (18.62 g, 348.2 mmol, 7 equiv.) were added portion wise. The reaction was stirred at 60 ºC under N_2 for 1 h until TLC (Pent/EtOAc 4:1) showed full conversion. The mixture was cooled to rt, filtered and concentrated *in vacuo*. Column chromatography (Pentane/EtOAc + 1% Et_3N 9:1 → 7:3) gave **S2** in 44% yield (5.11 g, 23.9 mmol) (49% brsm). **^{1}H NMR (400 MHz, $CDCl_3$)** δ 6.46 (dd, *J* = 6.4, 2.0 Hz, 1H, H-1), 5.60 – 5.52 (m, 1H, H-3), 5.28 (dq, *J* = 4.7, 1.8 Hz, 1H, H-4), 4.63 (dt, *J* = 6.3, 1.9 Hz, 1H, H-2), 4.21 (q, *J* = 6.5 Hz, 1H, H-5), 2.15 (s, 3H, COC*H*$_3$), 2.01 (s, 3H, COC*H*$_3$), 1.27 (d, *J* = 6.6 Hz, 3H, H-6). **^{13}C NMR (101 MHz, $CDCl_3$)** δ 170.86 (C=O), 170.56 (C=O), 146.25 (C-1), 98.39 (C-2), 71.65 (C-3), 66.37 (C-4), 65.17 (C-5), 21.00 (CO*C*H$_3$), 20.85 (CO*C*H$_3$), 16.66 (C-6). **HRMS**: $[M+Na]^+$ calculated for $C_{10}H_{14}O_5Na$: 237.07389; found 237.07422

Phenyl 2-azido-2-deoxy-1-seleno-*α*-D-fucopyranoside (S3)

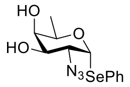

S2 (6.562 g, 30.66 mmol) and $(PhSe)_2$ (9.57 g, 30.66 mmol, 1 equiv.) was dissolved in DCM (155 mL, 0.2 M) and degassed under argon at rt for 30 min. The reaction was cooled to -30 ºC, added BAIB (9.87 g, 30.66 mmol, 1 equiv.) and $TMSN_3$ (8.1 mL, 61.31 mmol, 2 equiv.) and stirred at -20 ºC overnight until TLC (Tol/EtOAc 9:1) showed full conversion. Cyclohexene (12 mL) was added, and the reaction was stirred at rt for 30 min before being concentrated *in vacuo*. The lipophilic byproducts were removed by column chromatography (Pentane/EtOAc 10:0 → 7:3) were all the carbohydrate positive fractions were collected. The crude residue (14.46 g, 35 mmol, impure) was dissolved in MeOH (120 mL, 0.3 M) and added NaOMe (1.6 mL, 7 mmol, 0.2 equiv.). The reaction was stirred at rt for 2 h until TLC (Pent/EtOAc 1:1) showed full conversion and then neutralized with Amberlite IR-120 H^+ resins, filtered and concentrated *in vacuo*. The crude product was recrystallized in hot toluene to give **S3** in 67% yield (6.78 g, 20.7 mmol). **^{1}H NMR (400 MHz, MeOD)** δ 7.60 – 7.55 (m, 2H, Ar-*H*), 7.34 – 7.22 (m, 3H, Ar-*H*), 5.91 (dd, *J* = 5.2, 0.6 Hz, 1H, H-1), 4.29 (q, *J* = 6.5 Hz, 1H, H-5), 4.01 (dd, *J* = 9.9, 5.3 Hz, 1H, H-2), 3.76 – 3.68 (m, 2H, H-4, H-3), 1.15 (d, *J* = 6.6 Hz, 3H, H-6). **^{13}C NMR (101 MHz, MeOD)** δ 135.91 (Ar-*C*), 130.04 (Ar-*C*), 128.72 (Ar-C_q), 86.89 (C-1), 72.91 (C-3), 72.68 (C-4), 70.61 (C-5), 62.91 (C-2), 16.42 (C-6). **HRMS**: $[M+Na]^+$ calculated for $C_{12}H_{15}N_3O_3SeNa$: 352.01763; found 352.01709

Phenyl 2-azido-2-deoxy-3-*O*-(2-naphthylmethyl)-1-seleno-*α*-D-fucopyranoside (S4)

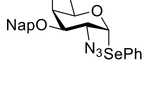

S3 (3.60 g, 10.94 mmol) was co-evaporated with toluene (x3) and dissolved in dry toluene (55 ml, 0.2 M). Bu_2SnO (2.778 g, 11.16 mmol, 1.02 equiv.) was added and the flask was equipped with a Dean-Stark. The reaction was heated to 140 ºC for 3 h. The now clear solution was cooled to 60 ºC before adding Bu_4NBr (3.704 g, 11.49 mmol, 1.05 equiv.), CsF (1.965 g, 11.16 mmol, 1.02 equiv.) and NapBr (2.540 g, 11.49 mmol, 1.05 equiv.). The reaction was heated to 120 ºC for 1 h until TLC (Pent/EtOAc 3:2) showed full conversion. The reaction was allowed to cool to rt before a 10% KF solution was added and the reaction was stirred for 30 min. The aqueous phase was extracted with EtOAc (x3) and the combined organic phases were washed with brine (x1), dried over Na_2SO_4, filtered and concentrated *in vacuo*. Column chromatography (Pentane/EtOAc 9:1 → 7:3) gave **S4** in 93% yield (4.768 g, 10.18 mmol). **^{1}H NMR (400 MHz, $CDCl_3$)** δ 7.92 – 7.82 (m, 4H, Ar-*H*), 7.63

– 7.45 (m, 5H, Ar-*H*), 7.33 – 7.27 (m, 3H, Ar-*H*), 5.91 (d, *J* = 5.4 Hz, 1H, H-1), 4.88 (dd, *J* = 13.6, 11.5 Hz, 2H, Ar-C*H*$_2$), 4.30 (qt, *J* = 6.6, 1.5 Hz, 1H, H-5), 4.21 (dd, *J* = 10.2, 5.3 Hz, 1H, H-2), 3.91 (dt, *J* = 3.2, 1.6 Hz, 1H, H-4), 3.76 (dd, *J* = 10.2, 3.1 Hz, 1H, H-3), 2.39 (t, *J* = 1.6 Hz, 1H, OH), 1.26 (d, *J* = 6.5 Hz, 3H, H-6). **^{13}C NMR (101 MHz, CDCl$_3$)** δ 133.35 (Ar-*C*), 133.33 (Ar-*C$_q$*), 129.24 (Ar-*C$_q$*), 128.79 (Ar-*C*), 128.63 (Ar-*C$_q$*) 128.13 (Ar-*C*), 127.94 (Ar-*C*), 127.91 (Ar-*C*), 127.17 (Ar-*C*), 126.53 (Ar-*C*), 126.45 (Ar-*C*), 125.81 (Ar-*C*), 85.30 (C-1), 79.35 (C-3), 72.42 (Ar-CH$_2$), 68.71 (C-5, C-4), 60.40 (C-2), 16.83 (C-6). **HRMS**: [M+H]$^+$ calculated for $C_{23}H_{23}N_3O_3SeH$: 470.09829; found 470.09776

Phenyl 2-azido-4-*O*-benzyl-2-deoxy-3-*O*-(2-naphthylmethyl)-1-seleno-*α*-D-fucopyranoside (S5)

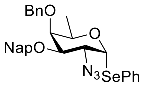

S4 (3.228 g, 6.89 mmol) was dissolved in DMF (67 mL, 0.1 M) and cooled to 0 ºC. BnBr (1.06 mL, 8.96 mmol, 1.3 equiv.) and NaH (60% suspension in mineral oil, 358 mg, 8.96 mmol, 1.3 equiv.) was added and the solution was slowly allowed to warm to rt and stirred under N$_2$ for 18 h until TLC (Pent/EtOAc 9:1) showed full conversion. The reaction was quenched with H$_2$O and extracted with Et$_2$O (x3). The combined organic phases were washed with brine (x1), dried over Na$_2$SO$_4$, filtered and concentrated *in vacuo*. Column chromatography (Pentane/EtOAc 95:5 → 85:15) gave **S5** in 88% yield (3.627 g. 6.49 mmol). **^{1}H NMR (400 MHz, CDCl$_3$)** δ 7.92 – 7.81 (m, 4H, Ar-*H*), 7.60 – 7.53 (m, 3H, Ar-*H*), 7.53 – 7.47 (m, 2H, Ar-*H*), 7.37 – 7.19 (m, 8H, Ar-*H*), 5.95 (d, *J* = 5.3 Hz, 1H, H-1), 5.01 – 4.87 (m, 3H, Ar-C*H*$_2$, Ar-C*H*$_2$), 4.65 (d, *J* = 11.4 Hz, 1H, Ar-C*H*$_2$), 4.40 (dd, *J* = 10.3, 5.3 Hz, 1H, H-2), 4.23 (q, *J* = 6.3 Hz, 1H, H-5), 3.79 (dd, *J* = 10.3, 2.7 Hz, 1H, H-3), 3.74 (dd, *J* = 2.8, 1.2 Hz, 1H, H-4), 1.14 (d, *J* = 6.5 Hz, 3H, H-6). **^{13}C NMR (101 MHz, CDCl$_3$)** δ 138.24 (Ar-*C$_q$*), 135.09 (Ar-*C$_q$*), 134.50 (Ar-*C*), 133.42 (Ar-*C$_q$*), 133.23 (Ar-*C$_q$*), 128.54 (Ar-*C*), 128.45 (Ar-*C*), 128.29 (Ar-*C*), 128.13 (Ar-*C*), 127.92 (Ar-*C*), 127.89 (Ar-*C*), 127.80 (Ar-*C*), 126.76 (Ar-*C*), 126.41 (Ar-*C*), 126.26 (Ar-*C*), 125.81 (Ar-*C*), 85.68 (C-1), 80.79 (C-3), 75.98 (C-4), 75.16 (Ar-*C*H$_2$-3), 72.77 (Ar-*C*H$_2$), 69.55 (C-5), 61.17 (C-2), 16.69 (C-6). **HRMS**: [M+Na]$^+$ calculated for $C_{30}H_{29}N_3O_3SeNa$: 582.12718; found 582.12685

2-azido-4-*O*-benzyl-2-deoxy-3-*O*-(2-naphthylmethyl)-*α*/*β*-D-fucopyranose (S6)

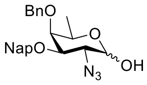

S5 (3.618 g, 6.47 mmol) was dissolved in acetone/H$_2$O (130 mL, 10:1, 0.05 M), cooled to 0 ºC and added NIS (2.912 g, 12.94 mmol, 2 equiv.). The reaction was stirred at 0 ºC for 15 min until TLC (Pent/EtOAc 3:2) showed full conversion. The solvents were evaporated and the residue was dissolved in EtOAc, washed with sat. aq. Na$_2$S$_2$O$_3$ (sat. aq.; x1), sat. aq. NaHCO$_3$ (sat. aq.; x1) and brine (x1), dried over Na$_2$SO$_4$, filtered and concentrated *in vacuo*. Column chromatography (Pentane/EtOAc 8:2 → 6:4) gave **S6** in 99% yield in a *α*/*β* ratio 1:0.9 (2.687 g, 6.405 mmol). **^{1}H NMR (400 MHz, CDCl$_3$)** δ 7.91 – 7.79 (m, 7H, Ar-*H*), 7.59 – 7.45 (m, 6H, Ar-*H*), 7.41 – 7.26 (m, 9H, Ar-*H*), 5.33 (t, *J* = 2.9 Hz, 1H, *α*-H-1), 4.97 (dd, *J* = 11.5, 4.5 Hz, 2H, Ar-C*H*$_2$), 4.89 (d, *J* = 10.1 Hz, 4H, Ar-C*H*$_2$), 4.68 (dd, *J* = 16.6, 11.5 Hz, 2H, Ar-C*H*$_2$), 4.47 (t, *J* = 7.5 Hz, 1H, *β*-H-1), 4.15 – 4.07 (m, 1H, *β*-H-5), 4.06 – 3.95 (m, 2H, *α*H-3, *α*-H-2), 3.80 (dd, *J* = 10.3, 7.9 Hz, 1H, *β*-H-2), 3.76 – 3.71 (m, 1H, *β*-H-4), 3.58 (dd, *J* = 2.8, 1.0 Hz, 1H, *α*-H-4), 3.48 (qd, *J* = 6.4, 1.1 Hz, 1H, *α*-H-5), 3.41 (dd, *J* = 10.3, 2.8 Hz, 1H, *β*-H-3), 3.29 (d, *J* = 7.1 Hz, 1H, *β*-OH), 2.77 (dd, *J* = 3.0, 0.9 Hz, 1H, *α*-OH), 1.21 (d, *J* = 6.4 Hz, 2H, *α*-H-6), 1.17 (d, *J* = 6.5 Hz, 3H, *β*-H-6). **^{13}C NMR (101 MHz, CDCl$_3$)** δ 138.29 (Ar-*C$_q$*), 138.16 (Ar-*C$_q$*), 135.21 (Ar-*C$_q$*), 135.14 (Ar-*C$_q$*), 133.23 (Ar-*C$_q$*), 128.55 (Ar-*C*), 128.52 (Ar-*C*), 128.46 (Ar-*C*), 128.11 (Ar-*C*), 128.07 (Ar-*C*), 127.98 (Ar-*C*), 127.94 (Ar-*C*), 127.90 (Ar-*C*), 127.88 (Ar-*C*), 96.58 (*β*-C-1), 92.58 (*α*-C-1), 81.05 (*β*-C-3), 77.88 (*α*-C-3), 76.22 (*β*-C-4), 75.03 (Ar-*C*H$_2$), 74.95 (Ar-*C*H$_2$), 72.86 (Ar-*C*H$_2$), 72.57 (Ar-*C*H$_2$), 71.20 (*α*-C-5), 67.01 (*β*-C-5), 64.96 (*β*-C-2), 60.46 (*α*-C-2), 17.05 (C-6), 16.97 (C-6). **HRMS**: [M+Na]$^+$ calculated for $C_{24}H_{25}N_3O_4Na$: 442.17428; found 442.17373

***Tert*-butyldiphenylsilyl 2-azido-4-*O*-benzyl-2-deoxy-3-*O*-(2-naphthylmethyl)-*β*-D-fucopyranoside (S7)**

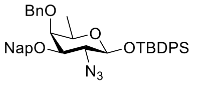

S6 (2.688 g, 6.408 mmol) was co-evaporated with toluene (x3), dissolved in dry DCM 32 mL, 0.2 M) and cooled to 0 ºC. TBDPS-Cl (1.97 mL, 7.69 mmol, 1.2 equiv.), imidazole (1.091 g, 16.02 mmol, 2.5 equiv.) and DMAP (157 mg, 1.282 mmol, 0.2 equiv.) were added and the reaction was stirred at rt under N_2 for 2 h until TLC (Pent/EtOAc 95:5) showed full conversion. The solution was dissolved in EtOAc, washed with 1 M HCl (x3) and brine (x1), dried over Na_2SO_4, filtered and concentrated *in vacuo*. Column chromatography (Pentane/EtOAc 100:0 → 90:10) gave **S7** in 96% (4.067 g, 6.18 mmol). **^{1}H NMR (400 MHz, $CDCl_3$)** δ 7.87 – 7.65 (m, 10H, Ar-*H*), 7.53 – 7.28 (m, 16H, Ar-*H*), 4.96 (d, *J* = 11.8 Hz, 1H, Ar-C*H*$_2$), 4.83 (d, *J* = 2.2 Hz, 2H, Ar-C*H*$_2$), 4.68 (d, *J* = 11.8 Hz, 1H, Ar-C*H*$_2$), 4.31 (d, *J* = 7.7 Hz, 1H, H-1), 3.89 (dd, *J* = 10.4, 7.7 Hz, 1H, H-2), 3.46 (dd, *J* = 3.1, 1.1 Hz, 1H, H-4), 3.25 (dd, *J* = 10.4, 2.9 Hz, 1H, H-3), 3.08 (qd, *J* = 6.4, 1.1 Hz, 1H, H-5), 1.11 (s, 9H, TBDPS-C*H*$_3$), 1.01 (d, *J* = 6.4 Hz, 3H, H-6). **^{13}C NMR (101 MHz, $CDCl_3$)** δ 138.59 (Ar-C_q), 136.25 (Ar-*C*), 136.11 (Ar-*C*), 135.44 (Ar-C_q), 134.94 (Ar-C_q), 133.59 (Ar-C_q), 133.36 (Ar-C_q), 133.28 (Ar-C_q), 133.17 (Ar-C_q), 129.81 (Ar-*C*), 129.64 (Ar-*C*), 128.43 (Ar-*C*), 128.38 (Ar-*C*), 128.36 (Ar-*C*), 128.07 (Ar-*C*), 127.87 (Ar-*C*), 127.79 (Ar-*C*), 127.56 (Ar-*C*), 127.32 (Ar-*C*), 126.64 (Ar-*C*), 126.34 (Ar-*C*), 126.16 (Ar-*C*), 125.83 (Ar-*C*), 97.44 (C-1), 81.18 (C-3), 75.35 (C-4), 74.79 (Ar-*C*H2), 73.58 (Ar-*C*H$_2$), 71.28 (C-5), 66.76 (C-2), 27.01 (TBDPS-*C*H$_3$), 16.71 (C-6). **HRMS**: $[M+Na]^+$ calculated for $C_{40}H_{43}N_3O_4SiNa$: 680.29205; found 680.29150

***Tert*-butyldiphenylsilyl 2-azido-4-*O*-benzyl-2-deoxy-*β*-D-fucopyranoside (10)**

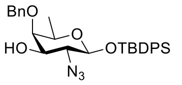

S7 (4.3923 g, 5.01 mmol) was dissolved in DCM/H_2O (50 mL, 20:1, 0.1 M) and added DDQ (1.705 g, 7.51 mmol, 1.5 equiv.). The reaction was stirred at rt under N_2 for 2 h until TLC (Pent/EtOAc 9:1) showed full conversion. The solution was quenched with $Na_2S_2O_3$ (aq. sat.), dissolved and extracted with EtOAc x3. The combined organic phases were washed with sat. aq. $NaHCO_3$ (sat. aq.; x4, until the yellow color disappeared) and brine (x1), dried over Na_2SO_4, filtered and concentrated *in vacuo*. Column chromatography (Pentane/EtOAc 95:5 → 80:20) gave **10** in 84% yield (2.89 g, 5.58 mmol). **^{1}H NMR (400 MHz, $CDCl_3$)** δ 7.82 – 7.69 (m, 4H, Ar-*H*), 7.51 – 7.33 (m, 11H, Ar-*H*), 4.82 (d, *J* = 11.6 Hz, 1H, Ar-C*H*$_2$), 4.71 (d, *J* = 11.6 Hz, 1H, Ar-C*H*$_2$), 4.36 (d, *J* = 7.7 Hz, 1H, H-1), 3.58 (dd, *J* = 10.3, 7.7 Hz, 1H, H-2), 3.47 (dd, *J* = 3.6, 1.2 Hz, 1H, H-4), 3.39-3.33 (m, 1H, H-3), 3.21 (qd, *J* = 6.5, 1.2 Hz, 1H, H-5), 2.24 (d, *J* = 7.4 Hz, 1H, OH), 1.15 (s, 9H, TBDPS-C*H*$_3$), 1.12 (d, *J* = 6.5 Hz, 3H, H-6). **^{13}C NMR (101 MHz, $CDCl_3$)** δ 138.10 (Ar-C_q), 136.21 (Ar-*C*), 136.05 (Ar-*C*), 133.56 (Ar-C_q), 133.17 (Ar-C_q), 129.88 (Ar-*C*), 129.71 (Ar-*C*), 128.76 (Ar-*C*), 128.25 (Ar-*C*), 128.22 (Ar-*C*), 127.60 (Ar-*C*), 127.35 (Ar-*C*), 96.99 (C-1), 78.84 (C-4), 76.04 (Ar-*C*H$_2$), 72.93 (C-3), 70.87 (C-5), 67.53 (C-2), 27.00 (TBDPS-*C*H$_3$), 16.69 (C-6). **HRMS**: $[M+Na]^+$ calculated for $C_{29}H_{35}N_3O_4SiNa$: 540.22945; found 540.22890

Synthesis of the L-fucose building block

3,4-di-*O*-acetyl-L-fucal (S8)

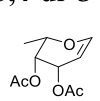

A solution of Ac_2O (80 mL, 14 equiv.) and pyridine (100 ml, 0.6 M) was cooled to 0 ºC. L-Fucose (10 g, 60.92 mmol) was added portion wise and the reaction was stirred at 4 C under N_2 for 18 h until TLC (Pent/EtOAc 3:2) showed full conversion. The solution was poured over ice and stirred until the ice was molten. The aqueous phase was extracted with DCM (x3) and the combined organic phases were washed with 1 M HCl (x3), H_2O (x2) and brine (x1), dried over Na_2SO_4, filtered and concentrated *in vacuo*. The residue was co-evaporated with toluene (x3) and used without any further purification. The crude product (18.9 g, 56.91 mmol) was dissolved in DCM (230 mL, 0.25

M) and cooled to 0 ºC. HBr in AcOH (33%, 15.5 mL, 85.36 mmol, 1.5 equiv.) was added using a dropping funnel and the reaction was stirred at 0 ºC under N_2 for 2 h until TLC (Pent/EtOAc 4:1) showed full conversion. The solution was poured over ice and stirred until the ice was molten. The aqueous phase was extracted with DCM (x3) and the combined organic phases were washed with aq. sat. $NaHCO_3$ (sat. aq.; x1), H_2O (x1) and brine (x1), dried over Na_2SO_4, filtered and concentrated *in vacuo*. The residue was co-evaporated with toluene (x3) and used immediately without any further purification. The crude product (18. g, 53.69 mmol) was dissolved in EtOAc (180 mL, 0.3 M) and Zn (24.58 g, 375.8 mmol, 7 equiv.) and NH_4Cl (20.10 g, 375.8 mmol, 7 equiv.) were added portion wise. The reaction was stirred at 60 ºC under N_2 for 1 h until TLC (Pent/EtOAc 4:1) showed full conversion, cooled to rt, filtered and concentrated *in vacuo*. Column chromatography (Pentane/EtOAc + 1% Et_3N 9:1 → 7:3) gave **S8** in 48% yield (6.35 g, 29.6 mmol) over 3 steps. **^{1}H NMR (400 MHz, $CDCl_3$)** δ 6.46 (dd, *J* = 6.3, 2.0 Hz, 1H, H-1), 5.57 (dtd, *J* = 4.9, 2.0, 1.1 Hz, 1H, H-3), 5.31 – 5.25 (m, 1H, H-4), 4.63 (dt, *J* = 6.4, 2.0 Hz, 1H, H-2), 4.20 (q, *J* = 6.6 Hz, 1H, H-5), 2.15 (s, 3H, COC*H*3), 2.01 (s, 3H, COC*H*3), 1.27 (d, *J* = 6.6 Hz, 3H, H-6). **^{13}C NMR (101 MHz, $CDCl_3$)** δ 170.85 (C=O), 170.55 (C=O), 146.24 (C-1), 98.39 (C-2), 72.16 (C-5), 66.37 (C-4), 65.17 (C-3), 21.00 (CO*C*H_3), 20.85 (CO*C*H_3), 16.66 (C-6). **HRMS**: $[M+Na]^+$ calculated for $C_{10}H_{14}O_5Na$: 237.07389; found 237.07334

Phenyl 2-azido-2-deoxy-1-seleno-*α*-L-fucopyranoside (S9)

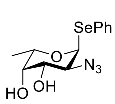

S8 (6.345 g, 29.64 mmol) and $(PhSe)_2$ (9.248 g, 29.64 mmol, 1 equiv.) was dissolved in DCM (150 mL, 0.2 M) and degassed under argon at rt for 30 min. The reaction was cooled to -30 ºC and added BAIB (9.548 g, 29.64 mmol, 1 equiv.) and $TMSN_3$ (7.7 mL, 59.28 mmol, 2 equiv.). The reaction was allowed to warm to -20 ºC and stirred overnight until TLC (Tol/EtOAc 10:1) showed full conversion. Cyclohexene (10 mL) was added and the reaction was stirred at rt for 30 min before concentration *in vacuo*. The lipophilic by products were removed by column chromatography (Pentane/EtOAc 10:0 → 7:3) were all the carbohydrate positive fraction were collected. The crude residue (12.399 g, 30.01 mmol) was dissolved in MeOH (100 mL, 0.3 M) and added NaOMe (1.4 mL, 6.004 mmol, 0.2 equiv.). The reaction was stirred at rt for 2 h until TLC (Pent/EtOAc 1:1) showed full conversion. The solution was neutralized with Amberlite IR-120 H^+ resins, filtered and concentrated *in vacuo*. The crude product was recrystallized in hot toluene to give **S9** in 64% yield (4.296 g. 13.09 mmol) over two steps. **^{1}H NMR (400 MHz, MeOD)** δ 7.63 – 7.53 (m, 2H, Ar-*H*), 7.31 – 7.27 (m, 3H, Ar-*H*), 5.91 (d, *J* = 5.4 Hz, 1H, H-1), 4.30 (q, *J* = 6.5 Hz, 1H, H-5), 4.01 (dd, *J* = 9.9, 5.3 Hz, 1H, H-2), 3.76 – 3.67 (m, 2H, H-3, H-4), 1.15 (d, *J* = 6.5 Hz, 3H, H-6). **^{13}C NMR (101 MHz, MeOD)** δ 135.91 (Ar-*C*), 130.04 (Ar-*C*), 128.73 (Ar-*C*), 86.90 (C-1), 72.92 (C-4), 72.68 (C-3), 70.62 (C-5), 62.91 (C-2), 16.42 (C-6). **HRMS**: $[M+H]^+$ calculated for $C_{12}H_{15}N_3O_3SeH$: 330.03569; found 330.03514

Phenyl 2-azido-2-deoxy-3-*O*-(2-naphthylmethyl)-1-seleno-*α*-L-fucopyranoside (S10)

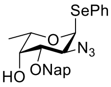

S9 (6.005 g, 18.25 mmol) was co-evaporated with toluene (x3) and dissolved in toluene (91 mL, 0.2 M). Bu_2SnO (4.634 g, 18.62 mmol, 1.02 equiv.) was added and the flask was equipped with a Dean Stark. The reaction was heated to 140 ºC for 3 h and the now clear solution was cooled to 60 ºC before adding Bu_4NBr (6.178 g, 19.16 mmol, 1,05 equiv.), CsF (2.828 g, 18.62 mmol, 1.02 equiv.) and NapBr (4.235 g, 19.16 mmol, 1.05 equiv.). The reaction was heated to 120 ºC for 1 h until TLC (Pent/EtOAc 3:2) showed full consumption. The reaction was allowed to cool to rt before a 10% KF solution was added and the reaction was stirred for 30 min. The aqueous phase was extracted with EtOAc (x3) and the combined organic phases were washed with brine (x1), dried over Na_2SO_4, filtered and concentrated *in vacuo*. Column chromatography (Pentane/EtOAc 9:1 → 7:3) gave **S10** in 91% yield (7.785 g, 26.62 mmol). **^{1}H NMR (400 MHz, $CDCl_3$)** δ 7.92 – 7.83 (m, 4H, Ar-*H*), 7.63 – 7.45 (m, 5H, Ar-*H*), 7.36 – 7.27 (m, 3H, Ar-*H*), 5.91 (d, *J* = 5.3 Hz, 1H, *H-1*), 4.92 (d, *J* = 11.5 Hz, 1H, Ar-C*H*$_2$), 4.86 (d, *J* = 11.5 Hz, 1H, Ar-C*H*$_2$), 4.35 – 4.26 (m, 1H, H-5), 4.21 (dd, *J* = 10.1, 5.3

Hz, 1H, H-2), 3.91 (dt, J = 3.1, 1.5 Hz, 1H, H-4), 3.76 (dd, J = 10.2, 3.1 Hz, 1H, H-3), 2.43 (t, J = 1.6 Hz, 1H, OH) , 1.26 (d, J = 6.6 Hz, 3H, H-6). **^{13}C NMR (101 MHz, $CDCl_3$)** δ 134.55 (Ar-C_q), 134.60 (Ar-*C*), 133.33 (Ar-C_q), 133.31 (Ar-C_q), 129.23 (Ar-C), 128.76 (Ar-*C*), 127.92 (Ar-*C*), 127.89 (Ar-*C*), 127.14 (Ar-*C*), 126.51 (Ar-*C*), 126.43 (Ar-*C*), 125.80 (Ar-*C*), 85.29 (C-1), 79.35 (C-3), 72.38 (Ar-CH_2), 68.70 (C-4, C-5), 68.68 (C-4, C-5), 60.37 (C-2), 16.17 (C-6). **HRMS**: $[M+H]^+$ calculated for $C_{23}H_{23}N_3O_3SeH$: 470.09829; found 470.09776

Phenyl 2-azido-4-*O*-benzyl-2-deoxy-3-*O*-(2-naphthylmethyl)-L-fucopyranoside (11a)

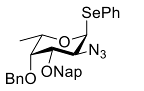

S10 (3.371 g, 7.20 mmol) was dissolved in DMF (72 mL, 0.1 M) and cooled to 0 ºC. BnBr (1.1 mL, 9.35 mmol, 1.3 equiv.) and NaH (374 mg, 0.95 mmol, 1.3 equiv.) was added and the solution was stirred under N_2 at rt for 16 h until TLC (Pent/EtOAc 9:1) showed full conversion. The reaction was quenched with H_2O and extracted with Et_2O (x3). The combined organic phases were washed with brine (x1), dried over Na_2SO_4, filtered and concentrated *in vacuo*. Column chromatography (Pentane/EtOAc 95:5 → 85:15) gave **11a** in 93% yield (3.743 g, 6.70 mmol). **^{1}H NMR (400 MHz, $CDCl_3$)** δ 7.94 – 7.83 (m, 4H, Ar-*H*), 7.64 – 7.53 (m, 4H, Ar-H), 7.41 – 7.21 (m, 9H, Ar-H), 5.98 (d, J = 5.3 Hz, 1H, H-1), 4.99 (d, J = 11.4 Hz, 1H, Ar-CH_2), 4.97 – 4.88 (m, 2H, Ar-CH_2), 4.67 (d, J = 11.4 Hz, 1H, Ar-CH_2), 4.43 (dd, J = 10.3, 5.3 Hz, 1H, H-2), 4.25 (q, J = 6.1 Hz, 1H, H-5), 3.81 (dd, J = 10.3, 2.7 Hz, 1H, H-3), 3.76 (m, 1H, H-4), 1.16 (d, J = 6.5 Hz, 3H, H-6). **^{13}C NMR (101 MHz, $CDCl_3$)** δ 138.21 (Ar-C_q), 135.06 (Ar-C_q), 133.39 (Ar-*C*), 133.19 (Ar-C_q), 129.14 (Ar-C_q), 128.80 (Ar-*C*), 128.50 (Ar-C_q), 128.42 (Ar-*C*), 128.25 (Ar-*C*), 128.10 (Ar-*C*), 127.89 (Ar-*C*), 127.86 (Ar-*C*), 127.77 (Ar-*C*), 126.72 (Ar-*C*), 126.38 (Ar-*C*), 126.22 (Ar-*C*), 125.77 (Ar-*C*), 85.64 (C-1), 80.76 (C-3), 75.94 (C-4), 72.72 (Ar-CH_2), 69.52 (Ar-CH_2), 61.13 (C-5), 16.66 (C-6). **HRMS**: $[M+NH_4]^+$ calculated for $C_{30}H_{29}N_3O_3SeNH_4$: 577.17179; found 577.17128

2-azido-4-*O*-benzyl-2-deoxy-3-*O*-(2-naphthylmethyl)-*α*/*β*-L-fucopyranose (S11)

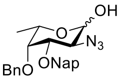

S10 (3.71 g, 6.673 mmol) was dissolved in acetone/H_2O (133 mL, 10:1, 0.05 M), cooled to 0 ºC and added NIS (3 g, 13.32 mmol, 2 equiv.). The reaction was stirred at 0 ºC for 15 min. The solvents were evaporated and the residue was dissolved in EtOAc, washed with sat. aq. $Na_2S_2O_3$ (sat. aq.; x1), sat. aq. $NaHCO_3$ (sat. aq.; x1) and brine (x1), dried over Na_2SO_4, filtered and concentrated *in vacuo*. Column chromatography (Pentane/EtOAc 8:2 → 6:4) gave **S11** in 89% yield (2.497 g, 5.95 mmol). **^{1}H NMR (400 MHz, $CDCl_3$)** δ 7.90 – 7.78 (m, 8H, Ar-*H*), 7.59 – 7.44 (m, 6H, Ar-*H*), 7.39 – 7.26 (m, 10H, Ar-*H*), 5.33 (t, J = 2.3 Hz, 1H, *α-H-1*), 4.97 (dd, J = 11.5, 4.4 Hz, 2H, Ar-CH_2), 4.89 (d, J = 9.5 Hz, 4H, Ar-CH_2), 4.68 (dd, J = 15.1, 11.5 Hz, 2H, Ar-CH_2), 4.47 (t, J = 6.9 Hz, 1H, *β-H-1*), 4.12 (q, J = 6.3 Hz, 1H, *α-H-5*), 4.01 (m, 2H, , *α-H-3*, *α-H-2*), 3.79 (dd, J = 10.3, 7.9 Hz, 1H, *β-H-2*), 3.73 (dd, J = 2.1, 1.2 Hz, 1H, *α-H-4*), 3.58 (dd, J = 2.9, 1.1 Hz, 1H, *β-H-3*), 3.48 (q, J = 6.4, 5.9 Hz, 1H, *β-H-5*), 3.41 (dd, J = 10.3, 2.8 Hz, 1H, *β-H-4*), 3.19 (d, J = 6.7 Hz, 1H, *β*-O*H*), 2.71 (d, J = 2.9 Hz, 1H, *α*-O*H*), 1.21 (d, J = 6.4 Hz, 2H, *β-H-6*), 1.17 (d, J = 6.5 Hz, 3H, *α-H-6*). **^{13}C NMR (101 MHz, $CDCl_3$)** δ 135.42 (Ar-C_q), 133.32 (Ar-C_q), 128.54 (Ar-*C*), 128.46 (Ar-*C*), 128.11 (Ar-*C*), 128.07 (Ar-*C*), 127.94 (Ar-*C*), 127.88 (Ar-*C*), 96.56 (*β*-C-1), 92.60 (*α*-C-1), 81.05 (*β*-C-4), 77.87 (*α*-C-3), 76.21 (*α*-C-4), 75.03 (Ar-CH_2), 74.97 (Ar-CH_2), 72.86 (Ar-CH_2), 72.56 (Ar-CH_2), 71.19 (*β*-C-5), 67.02 (*α*-C-5), 64.95 (*β*-C-2), 61.08 (*α*-C-2), 17.06 (C-6), 16.98 (C-6). **HRMS**: $[M+Na]^+$ calculated for $C_{24}H_{25}N_3O_4Na$: 442.17428; found 442.17373

2-azido-4-*O*-benzyl-2-deoxy-3-*O*-(2-naphthylmethyl)-1-*O*-(*N*-phenyl-2,2,2-trifluoroacetimidoyl)-*α*/*β*-L-fucopyranose (11b)

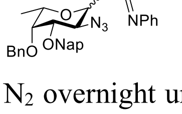

S11 (1.0951 g, 2.61 mmol) was co-evaporated with toluene (x3) and dissolved in dry acetone (19 mL, 0.2 M). K_2CO_3 (722 mg, 5.22 mmol, 2 equiv.) and ClC(=NPh)CF_3 (0.85 mL, 5.22 mmol, 2 equiv.) and was added and the reaction was stirred at rt under N_2 overnight until TLC (Pent/EtOAc 4:1) showed full conversion. The reaction was filtered on Celite

and concentrated *in vacuo*. Column chromatography (Pentane/EtOAc 95:5 → 85:15) gave **11b** in 97% yield (1.492 g, 2.526 mmol). **^{1}H NMR (400 MHz, $CDCl_3$)** δ 7.96 – 7.79 (m, 4H, Ar-*H*), 7.59 – 7.47 (m, 3H, Ar-*H*), 7.43 – 7.28 (m, 6H, Ar-*H*), 7.09 (td, *J* = 7.5, 1.1 Hz, 1H, Ar-*H*), 6.83 (d, *J* = 7.8 Hz, 2H, Ar-*H*), 5.47 (bs, 1H, *H-1*), 4.99 (d, *J* = 11.6 Hz, 1H, Ar-CH_2), 4.89 (s, 2H, Ar-CH_2), 4.71 (d, *J* = 11.6 Hz, 1H, Ar-CH_2), 4.09 (t, *J* = 9.3 Hz, 1H, *H-2*), 3.59 (s, 1H, *H-5*), 3.44 (s, 2H, *H-3*, *H-4*), 1.21 (d, *J* = 6.3 Hz, 3H, *H-6*). **^{13}C NMR (101 MHz, $CDCl_3$)** δ 138.06 (Ar- C_q), 134.95 (Ar- C_q), 133.35 (Ar- C_q), 126.49 (Ar-*C*), 126.34 (Ar-*C*), 125.84 (Ar-*C*), 124.41 (Ar-*C*), 119.44 (Ar-*C*), 80.97 (C-3/C-4), 75.04 (Ar-*C*H_2), 74.74 (C-5), 73.00 (Ar-*C*H_2), 72.01 (C-3/C-4), 62.26 (C-2), 16.82 (C-6). **HRMS** found for the hydrolyzed donor: $[M+Na]^+$ calculated for $C_{24}H_{25}N_3O_7Na$: 442.17428; found 442.17327

Synthesis of the D-mannose building block

1,3,4,6 Tetra-*O*-acetyl-*α*/*β*-D-mannopyranose (S12)

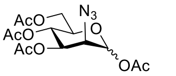

To an ice-cool solution of NaN_3 (4.522 g, 69.55 mmol, 1.5 equiv.) in pyridine (80 mL) was slowly added Tf_2O (9.3 mL, 55.65 mmol, 1.2 equiv.) and the resulting orange mixture was stirred at 0 ºC for 2 h. Mannosamine hydrochloride (10 g, 46.37 mmol) was dissolved in pyridine (47 mL) and added Et_3N (12.9 mL, 92.74 mmol, 2 equiv.) and $CuSO_4 \cdot 5\ H_2O$ (116 mg, 0.46 mmol, 0.01 equiv.) dissolved in as little H_2O as possible. The resulting blue mixture was cooled to 0 ºC and the freshly made TfN_3 solution was added dropwise via a dropping funnel. The resulting green mixture was stirred at 0 ºC for 4 h until TLC (DCM/MeOH/Et_3N 20:75:5) showed full conversion of the starting material. The solution turned yellow. Ac_2O (48.2 mL) was added and the reaction was stirred overnight after which TLC (Pent/EtOAc 3:2) showed full conversion. The reaction was dissolved in EtOAc and the organic phase was washed with 1 M HCl aq. (x3), sat. aq. $NaHCO_3$ (sat. aq.; x3), H_2O (x1) and brine (x1), dried over Na_2SO_4, filtered and concentrated *in vacuo* and co-evaporated with toluene x2 to remove pyridine and **S12** was obtained in an quant. yield and a *α*/*β* ratio on 5:2. Used without further purification. **^{1}H NMR (400 MHz, $CDCl_3$)** δ 6.12 (d, *J* = 1.9 Hz, 1H, *α*-H-1), 5.84 (d, *J* = 1.4 Hz, 1H, *β*-H-1), 5.43 – 5.34 (m, 2H, *α*-H-4, *α*-H-3), 5.30 (t, *J* = 9.9 Hz, 1H, *β*-H-4), 5.07 (dd, *J* = 9.8, 3.7 Hz, 1H, *β*-H-3), 4.27 (m, 2H, *α*/*β*-H-6), 4.18 – 4.12 (m, 1H, *β*-H-2), 4.09 (dd, *J* = 12.4, 2.4 Hz, 2H, *α*/*β*-H-6), 4.06 – 3.98 (m, 2H, *α*-H-2, *α*-H-5), 3.74 (ddd, *J* = 9.9, 4.8, 2.3 Hz, 1H, *β*-H-5), 2.19 (s, 3H, *β*-COCH_3), 2.17 (s, 3H, *α*-COCH_3), 2.12 (d, *J* = 1.1 Hz, 6H, *α*/*β*-COCH_3), 2.10 (s, 3H, *α*-COCH_3), 2.09 (s, 3H, *β*-COCH_3), 2.06 (s, 3H, *α*-COCH_3), 2.05 (s, 3H, *β*-COCH_3). **^{13}C NMR (101 MHz, $CDCl_3$)** δ 170.93 (C=O), 170.23 (C=O), 169.53 (C=O), 168.39 (C=O), 91.52 (*α* -C-1), 91.33 (*β*-C-1), 73.45 (*β*-C-5), 72.02 (*β*-C-3), 70.89 (*α* -C-3), 70.70 (*α*-C-5), 65.43 (*α*-C-4), 65.01 (*β*-C-4), 61.90 (*α*-C-6), 61.84 (*β*-C-6), 61.20 (*β*-C-2), 60.65 (*α*-C-2), 21.05 (CO*C*H_3), 20.88 (CO*C*H_3), 20.77 (CO*C*H_3), 20.68 (CO*C*H-$_3$). **HRMS**: $[M+Na]^+$ calculated for $C_{14}H_{19}N_3O_9Na$: 396.10190; found 396.10135

Phenyl 3,4,6 tri-*O*-acetyl-2-azido-2-deoxy-1-thio-*α*/*β*-D-mannopyranoside[12] (S13)

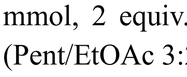

To an ice-cooled solution of **S12** (17.09 g, 45.78 mmol) in dry DCM (230 mL, 0.2 M) was slowly added PhSH (4.7 mL, 45.78 mmol, 1 equiv.) and BF_3OEt_2 (11.3 mL, 91.56 mmol, 2 equiv.) the resulting mixture was allowed to warm to rt and stirred under N_2 until TLC (Pent/EtOAc 3:2) showed full composition of the starting material (3 days). The reaction was quenched with Et_3N, diluted in DCM and the organic phase was washed with sat. aq. $NaHCO_3$ (sat. aq.; x1), 1 M NaOH (x3), H_2O (x1) and brine (x1), dried over Na_2SO_4, filtered and concentrated *in vacuo*. Column chromatography (Pentane/EtOAc 90:10 → 70:30) gave **S13** in 88% yield (16.99 g, 40.12 mmol) with a *α*/*β* ratio on 89:11. NMR reported for the *α*-anomer. **^{1}H NMR (400 MHz, $CDCl_3$)** δ 7.54 – 7.44 (m, 2H, Ar-*H*), 7.37 – 7.29 (m, 3H, Ar-*H*), 5.53 (d, *J* = 1.0 Hz, 1H, *H-1*), 5.39 – 5.33 (m, 2H, H-3, H-*4*), 4.52 – 4.46 (m, 1H*, H-5*), 4.31 – 4.24 (m, 2H, H-2, H-6), 4.08 (dd, *J* = 12.3, 2.4 Hz, 1H, H-6), 2.12 (s, 3H,

COCH_3), 2.08 (s, 3H, COCH_3), 2.06 (s, 3H, COCH_3). **^{13}C NMR (101 MHz, CDCl$_3$)** δ 170.84 (C=O), 170.11 (C=O), 169.63 (C=O), 132.08 (Ar-*C*), 129.45 (Ar-*C*), 128.40 (Ar-*C*), 85.96 (C-1), 71.30 (C-4), 69.68 (C-5), 66.17 (C-3), 62.82 (C-2), 62.29 (C-6), 20.86 (CO*C*H$_3$), 20.83 (CO*C*H$_3$), 20.72 (CO*C*H$_3$). **HRMS**: [M+Na]$^+$ calculated for $C_{18}H_{21}N_3O_7SNa$: 446.09979; found 446.09924

Phenyl 2-azido-2-deoxy-1-thio-*α*-D-mannopyranoside (S14)

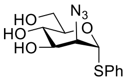

α-**S13** (14.95 g, 35.30 mmol) was dissolved in MeOH (117 mL, 0.3 M) and added NaOMe (25% wt in MeOH, 0.8 mL, 3.53 mmol, 0.1 equiv.). The reaction was stirred at rt for 3 h until TLC (Pent/EtoAc 4:6) showed full conversion and then neutralized with Amberlite IR-120 H$^+$ resins, filtered and concentrated. The crude **S14** was isolated in 90% (9.42 g, 31.69 mmol) and used without further purification. **^{1}H NMR (400 MHz, MeOD)** δ 7.57 – 7.50 (m, 2H, Ar-*H*), 7.36 – 7.26 (m, 3H, Ar-*H*), 5.48 (d, *J* = 1.4 Hz, 1H, H-1), 4.14 (dd, *J* = 3.8, 1.5 Hz, 1H, H-2), 4.06 – 4.01 (m, 1H, H-5), 3.94 (dd, *J* = 9.3, 3.8 Hz, 1H, H-3), 3.81 (dd, *J* = 12.1, 2.4 Hz, 1H, H-6), 3.72 (dd, *J* = 12.1, 5.7 Hz, 1H, H-6), 3.67 (t, *J* = 9.5 Hz, 1H, H-4). **^{13}C NMR (101 MHz, MeOD)** δ 135.99 (Ar- C_q), 133.73 (Ar-*C*), 130.16 (Ar-*C*), 128.31 (Ar-*C*), 87.29 (C-1), 75.82 (C-5), 73.09 (C-3), 69.30 (C-4), 67.10 (C-2), 60.72 (C-6). **HRMS**: [M+Na]$^+$ calculated for $C_{12}H_{15}N_3O_4SNa$: 320.06810; found 320.06755

Phenyl 2-azido-2-deoxy-4,6-*O*-(*p*-methoxybenzylidene)-1-thio-*α*-D-mannopyranoside (S15)

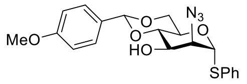

S14 (9.47 g, 31.85 mmol) was co-evaporated with toluene (x3) and dissolved in dry MeCN (160 mL, 0.2 M). Anisaldehyde dimethyl acetal (7 mL, 41.41 mmol, 2 equiv.) and camphorsulfonic acid (370 mg, 1.59 mmol, 5 mol%) was added sequentially and the reaction was stirred on the rotary evaporator (300 mbar at 50 ºC) until TLC (Pent/EtOAc 7:3) showed full conversion (~1 h). The reaction was quenched with Et_3N and concentrated *in vacuo*. Column chromatography (Pentane/EtOAc 95:5 → 75:25) gave **S15** in 90% (11.93 g, 28.72 mmol). **^{1}H NMR (500 MHz, CDCl$_3$)** δ 7.50 – 7.39 (m, 4H, Ar-*H*), 7.39 – 7.29 (m, 3H, Ar-*H*), 6.95 – 6.88 (m, 2H, Ar-*H*), 5.55 (s, 1H, PMP-C*H*), 5.47 (d, *J* = 1.2 Hz, 1H, H-1), 4.33-4.28 (td, *J* = 9.7, 4.9 Hz, 1H, H-5), 4.26-4.23 (dt, *J* = 9.7, 3.9 Hz, 1H, H-3), 4.22 – 4.20 (m, 1H, H-2), 4.19 (d, *J* = 5.0 Hz, 1H, H-6), 3.81 (s, 3H, OC*H*$_3$), 3.79 (d, *J* = 10.3 Hz, 1H, H-6), 2.82 (d, *J* = 3.9 Hz, 1H, OH). **^{13}C NMR (126 MHz, CDCl$_3$)** δ 160.48 (Ar-C_q), 133.11 (Ar-C_q), 132.06 (Ar-*C*), 130.4 (Ar-C_q), 129.42 (Ar-*C*), 128.25 (Ar-*C*), 127.77 (Ar-*C*) 113.92 (Ar-*C*), 102.43 (PMB-*C*H), 87.65 (C-1), 79.16 (C-4), 69.39 (C-3), 68.43 (C-6), 65.20 (C-2), 64.73 (C-5), 55.46 (O*C*H$_3$). **HRMS**: [M+H]$^+$ calculated for $C_{20}H_{21}N_3O_5SH$: 416.12802; found 416.12876

Phenyl 2-azido-2-deoxy-4,6-*O*-(*p*-methoxybenzylidene)-3-*O*-(2-naphthylmethyl)-1-thio-*α*-D-mannopyranoside (S16)

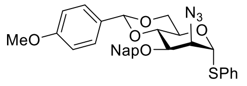

S15 (11.07 g, 26.65 mmol) was co-evaporated with toluene (x3), dissolved in DMF (266 mL, 0.1 M) and cooled to 0 ºC. NaH (60% in mineral oil, 1.386 g, 34.64 mmol, 1.3 equiv.) was added and the mixture was stirred for 20 min. Then NapBr (7.656 g, 34.64 mmol, 1.3 equiv.) was added and the reaction was slowly allowed to warm to rt and stirred for 22 h (overnight) under N_2 after which TLC (Pent/EtOAc 4:1) showed full conversion. The reaction was quenched with H_2O and extracted with Et_2O (x3). The combined organic phases was washed with brine (x1) and dried over Na_2SO_4, filtered and concentrated *in vacuo.* Column chromatography (Pentane/EtOAc 95:5 → 80:20) gave **S16** in 98% yield (14.46 g, 2602 mmol). **^{1}H NMR (400 MHz, CDCl$_3$)** δ 7.88 – 7.81 (m, 3H, Ar-*H*), 7.54 – 7.46 (m, 3H, Ar-*H*), 7.47 – 7.43 (m, 2H, Ar-*H*), 7.42 – 7.37 (m, 2H, Ar-*H*), 7.32 – 7.28 (m, 3H, Ar-*H*), 6.96 – 6.87 (m, 2H, Ar-*H*), 5.62 (s, 1H, PMP-C*H*), 5.43 (d, *J* = 1.1 Hz, 1H, H-1), 5.07 (d, *J* = 12.7 Hz, 1H, Ar-C*H*$_2$), 4.92 (d, *J* = 12.4 Hz, 1H, Ar-C*H*$_2$), 4.32 (m, 1H, H-5), 4.25 – 4.15 (m, 4H, H-2, H-3, H-4, H-6), 3.87 – 3.84 (m, 1H, H-6) 3.84 (s, 3H, OC*H*$_3$). **^{13}C NMR (101 MHz, CDCl$_3$)** δ 160.23 (Ar-C_q), 135.35 (Ar-C_q), 133.41 (Ar-C_q), 133.17 (Ar-C_q), 132.91 (Ar-C_q), 132.14 (Ar-*C*), 129.95 (Ar-C_q), 129.39 (Ar-*C*), 128.43 (Ar-*C*), 128.25 (Ar-*C*),

128.17 (Ar-*C*), 127.83 (Ar-*C*), 127.59 (Ar-*C*), 126.58 (Ar-*C*), 126.27 (Ar-*C*), 126.13 (Ar-*C*), 125.64 (Ar-*C*), 113.75 (Ar-*C*), 101.87 (PMP-*C*H), 87.34 (C-1), 79.19 (C-3/C-4), 75.95 (C-3/C-4), 73.55 (Ar-CH_2), 68.46 (C-6), 65.33 (C-5), 64.27 (C-2), 55.45 (OCH_3). **HRMS**: $[M+H]^+$ calculated for $C_{31}H_{29}N_3O_5SH$: 556.19062; found 556.19007

Phenyl 2-azido-2-deoxy-3-*O*-(2-naphthylmethyl)-1-thio-*α*-D-mannopyranoside (S17)

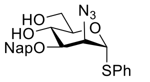

S16 (14.46 g, 26.05 mmol) was co-evaporated with toluene (x2) and dissolved in MeOH (0.1 M). Camphorsulfonic acid (605 mg, 2.61 mmol, 0.1 equiv.) was added and the reaction was stirred for 1 h at rt until TLC (Pent/EtOAc 4:1) showed full conversion. The reaction was quenched with Et_3N and concentrated *in vacuo*. Column chromatography (Pentane/EtOAc 80:20 → 50:50) gave **S17** in 88% yield (9.97 g, 22.79 mmol). **^{1}H NMR (400 MHz, $CDCl_3$)** δ 7.92 – 7.81 (m, 4H, Ar-*H*), 7.58 – 7.47 (m, 3H, Ar-*H*), 7.46 – 7.36 (m, 2H, Ar-*H*), 7.33 – 7.28 (m, 3H, Ar- *H*), 5.42 (d, *J* = 1.5 Hz, 1H, H-1), 4.92 (d, *J* = 11.7 Hz, 1H, Ar-CH_2), 4.84 (d, *J* = 11.7 Hz, 1H, Ar-CH_2), 4.18 – 4.10 (m, 2H, H-2, H-5), 4.05 (td, *J* = 9.3, 2.6 Hz, 1H, H-4), 3.94 (dd, *J* = 9.1, 3.5 Hz, 1H, H-3), 3.83 (dt, *J* = 4.9, 2.8 Hz, 2H, H-6), 2.80 (d, *J* = 3.0 Hz, 1H, C4-OH), 2.10 – 1.97 (m, 1H, C6-OH). **^{13}C NMR (101 MHz, $CDCl_3$)** δ 134.63 (Ar-C_q), 133.38 (Ar-C_q), 133.33 (Ar-*C*), 132.98 (Ar-*C*), 132.35 (Ar-*C*), 129.39 (Ar-*C*), 128.84 (Ar-*C*), 128.31 (Ar-*C*), 128.16 (Ar-*C*), 127.91 (Ar-*C*), 127.39 (Ar-*C*), 126.53 (Ar-*C*), 126.45 (Ar-*C*), 125.95 (Ar-*C*), 86.63 (C-1), 79.67 (C-3), 73.41 (C-5), 72.56 (CH_2-Ar), 67.22 (C-4), 62.36 (C-6), 62.09 (C-2). **HRMS**: $[M+Na]^+$ calculated for $C_{23}H_{23}N_3O_4SNa$: 460.13073; found 460.13015

Benzyl (phenyl 2-azido-2-deoxy-3-*O*-(2-naphthylmethyl)-1-thio-*α*-D-mannopyranosiduronate) (S18)

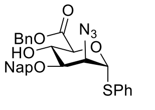

S17 (9.95 g, 22.76 mmol) was dissolved in DCM/H_2O/*t*-BuOH (8:4:1, 114 mL, 0.2 M) and under vigorous stirring added AcOH (0.26 mL, 4.55 mmol, 0.2 equiv.), TEMPO (711 mg, 4.55 mmol, 0.2 equiv.) and $PhI(AcO)_2$ (BAIB, 18.32 g, 56.89 mmol, 2.5 equiv.). The reaction was stirred at 4 ºC overnight until full consumption on TLC (Pent/EtOAc 1:1) was observed. The reaction was quenched with $Na_2S_2O_3$ (aq., sat.) and the aqueous phase was extracted with EtOAc (x3). The combined organic phases were washed with brine (x1), dried over Na_2SO_4, filtered and concentrated *in vacuo*. The residue was co-evaporated with toluene (x3) and used without any further purifications. The crude product (22.76 mmol) was dissolved in DMF (230 mL, 0.1 M) and cooled to 0 ºC. K_2CO_3 (6.291 g, 45.55 mmol, 1.5 equiv.) and BnBr (5.4 mL, 45.52 mmol, 1.5 equiv.) were added and the reaction was stirred overnight until TLC (Pent/EtOAc 7:3) showed full conversion. The reaction was quenched with H_2O and extracted with Et_2O (x3). The combined organic phase was washed with H_2O (x1) and brine (x1), dried over Na_2SO_4, filtered and concentrated *in vacuo*. Column chromatography (Pentane/EtOAc 90:10 → 70:30) gave **S18** in 75% yield (9.30 g, 17.17 mmol). **^{1}H NMR (400 MHz, $CDCl_3$)** δ 7.90 – 7.80 (m, 4H, Ar-*H*), 7.52 – 7.43 (m, 5H, Ar-*H*), 7.36 – 7.32 (m, 3H, Ar-*H*), 7.30 – 7.26 (m, 2H, Ar-*H*), 7.25 – 7.16 (m, 3H, Ar-*H*), 5.52 (d, *J* = 2.9 Hz, 1H, H-1), 5.16 (s, 2H, Ar-CH_2), 4.95 (d, *J* = 11.9 Hz, 1H, Ar-CH_2), 4.88 (d, *J* = 11.8 Hz, 1H, Ar-CH_2), 4.68 (d, *J* = 8.0, 1H, H-5), 4.38 (td, *J* = 7.9, 3.5 Hz, 1H, H-4), 4.00 – 3.91 (m, 2H, H-2, H-3), 2.88 (d, *J* = 3.5 Hz, 1H, C4-OH). **^{13}C NMR (101 MHz, $CDCl_3$)** δ 169.80 (C-6), 135.06 (Ar-*C*), 134.83 (Ar-*C*), 133.38 (Ar-*C*), 133.28 (Ar-*C*), 132.32 (Ar-*C*), 129.25 (Ar-*C*), 128.75 (Ar-*C*), 128.63 (Ar-*C*), 128.61 (Ar-*C*), 128.31 (Ar-*C*), 128.25 (Ar-*C*), 128.16 (Ar-*C*), 127.88 (Ar-*C*), 127.10 (Ar-*C*), 126.41 (Ar-*C*), 126.31 (Ar-*C*), 125.89 (Ar-*C*), 85.70 (C-1), 78.10 (C-2), 73.63 (Ar-CH_2), 72.95 (C-5), 68.64 (C-4), 67.53 (Ar-CH_2), 61.38 (C-3). **HRMS**: $[M+Na]^+$ calculated for $C_{30}H_{27}N_3O_5SNa$: 564.15691; found 564.15636

Benzyl (phenyl 4-*O*-acetyl-2-azido-2-deoxy-3-*O*-(2-naphthylmethyl)-1-thio-*α*-D-mannopyranosiduronate) (12a)

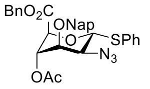

S18 (1.662 g, 3.05 mmol) was dissolved in pyridine (15 mL, 0.2 M) and cooled to 0 ºC. Ac_2O (0.57 mL, 6.10 mmol, 2 equiv.) and DMAP (74 mg, 0.61 mmol, 0.2 equiv.) was added and the reaction was stirred under N_2 for 30 min until TLC (Pent/EtOAc 3:1) showed full conversion. The reaction was quenched with MeOH, diluted in EtOAc and washed with 1 M HCl (x3), sat. $NaHCO_3$ (sat. aq.; x1) and brine (x1), dried over Na_2SO_4, filtered and concentrated *in vacuo*. Column chromatography (Pentane/EtOAc 9:1 → 6:4) gave **12a** in 95% yield (1.70 g, 2.91 mmol). **^{1}H NMR (400 MHz, $CDCl_3$)** δ 7.84 – 7.77 (m, 3H, Ar-*H*), 7.75 (d, *J* = 1.7 Hz, 1H, Ar-*H*), 7.65 – 7.55 (m, 2H, Ar-*H*), 7.52 – 7.39 (m, 3H, Ar-*H*), 7.24 (dt, *J* = 5.1, 2.5 Hz, 6H, Ar-*H)*, 7.15 – 7.07 (m, 2H, Ar-*H*), 5.78 (d, *J* = 9.3 Hz, 1H, H-1), 5.62 (dd, *J* = 4.8, 2.9 Hz, 1H, H-4), 5.01 (d, *J* = 12.1 Hz, 1H, Ar-CH_2), 4.82 (d, *J* = 12.2 Hz, 1H, Ar-CH_2), 4.67 (s, 2H, Ar-CH_2), 4.62 (d, *J* = 2.9 Hz, 1H, H-5), 3.98 (dd, *J* = 4.7, 3.0 Hz, 1H, H-3), 3.45 (dd, *J* = 9.5, 2.9 Hz, 1H, H-2), 2.02 (s, 3H, COC*H*$_3$). **^{13}C NMR (101 MHz, $CDCl_3$)** δ 169.74 (C-6), 167.86 (C=O), 134.84 (Ar-*C$_q$*), 133.96 (Ar-*C$_q$*), 133.25 (Ar-*C$_q$*), 133.21 (Ar-*C*), 132.45 (Ar-*C$_q$*), 131.91 (Ar-*C*), 128.99 (Ar-*C*), 128.65 (Ar-*C*), 128.58 (Ar-*C*), 128.48 (Ar-*C*), 128.11 (Ar-*C*), 128.02 (Ar-*C*), 127.82 (Ar-*C*), 127.36 (Ar-*C*), 126.38 (Ar-*C*), 126.34 (Ar-*C*), 125.93 (Ar-*C*), 81.09 (C-1), 74.62 (C-3), 73.66 (C-5), 73.02 (Ar-*C*H$_2$), 68.47 (C-4), 67.53 (Ar-*C*H$_2$), 57.90 (C-2), 21.00 (CO*C*H$_3$). **HRMS**: $[M+Na]^+$ calculated for $C_{32}H_{29}N_3O_6SNa$: 606.16743; found 606.16693

Benzyl (4-*O*-acetyl-2-azido-2-deoxy-3-*O*-(2-naphthylmethyl)-*α*-D-mannopyranosiduronate) (S19)

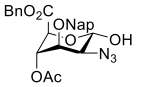

12a (1.327 g, 2.27 mmol) was co-evaporated with toluene (x3), dissolved in dry DCM (23 mL, 0.1 M) and cooled to 0 ºC. NIS (767 mg, 3.41 mmol, 1.5 equiv.) and TFA (0.17 mL, 2.27 mmol, 1 equiv.) was added and the reaction was stirred at 0 ºC under N_2 until TLC (Pent/EtOAc 7:3) showed full conversion (~4 h). The reaction was quenched with Et_3N (1 equiv.) and $NaHCO_3$ (sat. aq.) was added and the solution was stirred vigorously. The solution was diluted in EtOAc, washed with $Na_2S_2O_3$ (sat. aq.; x1), sat. $NaHCO_3$ (sat. aq.; x1) and brine (x1), dried over Na_2SO_4, filtered and concentrated. Column chromatography (Pentane/EtOAc 8:2 → 6:4) gave **S19** in 75% yield (833 mg, 1.69 mmol). **^{1}H NMR (400 MHz, $CDCl_3$)** δ 7.81 (dd, *J* = 8.3, 2.7 Hz, 3H, Ar-*H*), 7.73 (d, *J* = 1.6 Hz, 1H, Ar-*H*), 7.54 – 7.43 (m, 2H, Ar-*H*), 7.40 (dd, *J* = 8.5, 1.7 Hz, 1H, Ar-*H*), 7.35 – 7.21 (m, 4H, Ar-*H*), 7.18 (dd, *J* = 6.7, 3.0 Hz, 2H, Ar-*H*), 5.66 (dd, *J* = 6.6, 4.4 Hz, 1H, H-1), 5.55 (dd, *J* = 5.3, 3.9 Hz, 1H, H-4), 5.05 (d, *J* = 12.1 Hz, 1H, Ar-C*H*$_2$), 4.86 (d, *J* = 12.2 Hz, 1H, Ar-C*H*$_2$), 4.74 – 4.65 (dd, 2H, *J* = 11.5, 5.5 Hz, , Ar-C*H*$_2$), 4.57 (d, *J* = 3.9 Hz, 1H, H-5), 3.99 (dd, *J* = 5.4, 3.1 Hz, 1H, H-3), 3.94 (d, *J* = 4.8 Hz, 1H, OH), 3.62 (dd, *J* = 6.7, 3.1 Hz, 1H, H-2), 2.01 (s, 3H, COC*H*$_3$). **^{13}C NMR (101 MHz, $CDCl_3$)** δ 169.91 (C=O), 168.34 (C=O), 134.84 (Ar-*C$_q$*), 133.25 (Ar-*C$_q$*), 133.20 (Ar-*C$_q$*), 128.69 (Ar-*C*), 128.66 (Ar-*C*), 128.40 (Ar-*C*), 128.09 (Ar-*C*), 127.83 (Ar-*C*), 126.81 (Ar-*C*), 126.38 (Ar-*C*), 126.26 (Ar-*C*), 125.67 (Ar-*C*), 91.65 (C-1), 75.09 (C-3), 72.98 (C-5), 72.37 (Ar-*C*H$_2$), 68.88 (C-4), 67.73 (Ar-*C*H$_2$), 60.60 (C-2), 21.01 (CO*C*H$_3$). **HRMS**: $[M+Na]^+$ calculated for $C_{26}H_{25}N_3O_7Na$: 514.15902; found 514.15847

Benzyl (4-*O*-acetyl-2-azido-2-deoxy-3-*O*-(2-naphthylmethyl)-1-*O*-(*N*-phenyl-2,2,2-trifluoroacetimidoyl)-*α*/*β*-D-mannopyranosiduronate) (12b)

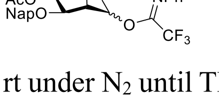

S19 (1.656 g, 2.37 mmol) was co-evaporated with toluene (x3) and dissolved in dry acetone (12 mL, 0.2 M). K_2CO_3 (656 mg, 4.74 mmol, 2 equiv.) and ClC(=NPh)CF_3 (0.77 mL, 4.74 mmol, 2 equiv.) were added and the reaction was stirred overnight at rt under N_2 until TLC (Pent/EtOAc 4:1) showed full conversion. The reaction was filtered on Celite and concentrated *in vacuo*. Column chromatography (Pentane/EtOAc 9:1 → 6:4) gave **12b** in 95% yield (1.49 g, 2.249 mmol). **^{1}H NMR (400 MHz, CD_3CN)** δ 7.93 – 7.77 (m, 6H, Ar-*H*), 7.56 – 7.41 (m, 4H, Ar-*H*), 7.43 – 7.19 (m, 10H, Ar-*H*), 7.19 – 7.07 (m, 2H, Ar-*H*), 6.79 (d, *J* = 8.1 Hz, 3H, Ar-*H*), 6.40 (bs,

1H, H-1), 5.41 (t, *J* = 6.8 Hz, 1H, H-4), 5.07 (d, *J* = 12.1 Hz, 1H, Ar-C*H*$_2$), 4.96 (d, *J* = 12.2 Hz, 1H, Ar-C*H*$_2$), 4.79 (d, *J* = 2.7 Hz, 3H, Ar-C*H*$_2$), 4.48 (d, *J* = 6.2 Hz, 1H, H-5), 4.19 – 4.10 (m, 2H, H-2/H-3), 2.12 (s, 3H, COC*H*$_3$). **^{13}C NMR (101 MHz, CD$_3$CN)** δ 135.88 (Ar-*C$_q$*), 134.08 (Ar-*C$_q$*), 129.81 (Ar-*C*), 129.45 (Ar-*C*), 129.43 (Ar-*C*), 129.36 (Ar-*C*), 129.10 (Ar-*C*), 128.74 (Ar-*C*), 128.54 (Ar-*C*), 127.96 (Ar-*C*), 127.29 (Ar-*C*), 127.20 (Ar-*C*), 127.03 (Ar-*C*), 75.77 (C-3), 73.61 (C-5/ Ar-*C*H$_2$), 68.35 (C-4), 68.28 (Ar-*C*H$_2$), 60.04 (C-2), 29.62 (C-OCH$_3$). **HRMS**: [M+Na]$^+$ calculated for $C_{34}H_{39}F_3N_4O_7Na$: 685.1886; found 685.18778

Synthesis of the trisaccharide

***Tert*-butyldiphenylsilyl 2-azido-4-*O*-benzyl-2-deoxy-3-*O*-(2-naphthylmethyl)-*α*-L-fucopyranosyl-(1→3)-2-azido-4-*O*-benzyl-2-deoxy-*β*-D-fucopyranoside (13)**

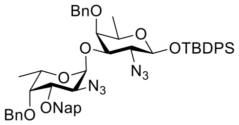

Donor **11b** (2.46 g, 4.171 mmol, 1.3 equiv.) and acceptor **10** (1.661 g, 3.21 mmol, 1 equiv.) was co-evaporated with toluene (3x), dissolved in dry DCM (32 mL, 0.1 M), added 3Å molecular sieves at rt and stirred for 30 min. TBSOTf (0.15 mL, 0.64 mmol, 0.2 equiv.) was added at rt and the reaction was stirred at rt until TLC (Pent/EtOAc 9:1) showed full conversion of the acceptor (~30 min). The reaction was quenched with Et$_3$N, dissolved in EtOAc, washed with NaHCO$_3$ (sat. aq.; x1), brine (x1), dried over Na$_2$SO$_4$ and concentrated. Column chromatography (Pentane/EtOAc 95:5 → 80:20) gave **13** in 86% yield (2.55 g, 2.77 mmol) and in a *α*/*β* ratio 95:5. **^{1}H NMR (400 MHz, CDCl$_3$)** δ 7.85 – 7.80 (m, 2H, Ar-*H*), 7.80 – 7.70 (m, 6H, Ar-*H*), 7.53 – 7.27 (m, 19H, Ar-*H*), 5.24 (d, *J* = 2.3 Hz, 1H, H-1'), 4.95 (d, *J* = 11.5 Hz, 1H, Ar-C*H*$_2$), 4.88 (d, *J* = 11.4 Hz, 1H, Ar-C*H*$_2$), 4.80 (d, *J* = 12.0 Hz, 1H, Ar-C*H*$_2$), 4.72 (d, *J* = 11.7 Hz, 1H, Ar-C*H*$_2$), 4.61 (dd, *J* = 11.6, 5.5 Hz, 2H, Ar-C*H*$_2$), 4.34 (d, *J* = 7.7 Hz, 1H, H-1), 3.93 – 3.89 (m, 1H, H-2), 3.87 (t, *J* = 1.3 Hz, 2H, H-3', H-2'), 3.78 (q, *J* = 6.4 Hz, 1H, H-5'), 3.55 (d, *J* = 1.5 Hz, 1H, H-4'), 3.37 (dd, *J* = 10.6, 2.9 Hz, 1H, H-4), 3.29 (dd, *J* = 3.0, 1.0 Hz, 1H, H-5), 3.17 (q, *J* = 6.9 Hz, 1H, H-5), 1.12 (s, 9H, TBDPS-C*H*$_3$), 1.06 (d, *J* = 6.5 Hz, 3H, H-6'), 1.04 (d, *J* = 6.4 Hz, 3H, H-6). **^{13}C NMR (101 MHz, CDCl$_3$)** δ 138.61 (Ar-*C$_q$*), 138.22 (Ar-*C$_q$*), 136.23 (Ar-*C*), 136.06 (Ar-*C*), 135.21 (Ar-*C$_q$*), 133.53 (Ar-*C$_q$*), 133.38 (Ar-*C$_q$*), 133.20 (Ar-*C$_q$*), 133.10 (Ar-*C$_q$*), 129.88 (Ar-*C*), 128.51 (Ar-*C*), 128.48 (Ar-*C*), 128.45 (Ar-*C*), 128.08 (Ar-*C*), 127.95 (Ar-*C*), 127.84 (Ar-*C*), 127.79 (Ar-*C*), 127.60 (Ar-*C*), 127.31 (Ar-*C*), 126.80 (Ar-*C*), 126.35 (Ar-*C*), 126.21 (Ar-*C*), 125.92 (Ar-*C*), 100.07 (C-1'), 97.35 (C-1), 79.30 (C-4), 78.90 (C-3), 77.16 (C-3'), 76.42 (C-4'), 75.41 (Ar-*C*H$_2$), 75.06 (Ar-*C*H$_2$), 72.75 (Ar-*C*H$_2$), 70.73 (C-5), 67.51 (C-5'), 66.54 (C-2'), 59.70 (C-2), 26.99 (TBDPS-*C*H$_3$), 16.86 (C-6), 16.67 (C-6). **HRMS**: [M+Na]$^+$ calculated for $C_{53}H_{58}N_6O_7SiNa$: 941.40339; found 941.40285

***Tert*-butyldiphenylsilyl 2-azido-4-*O*-benzyl-2-deoxy-*α*-L-fucopyranosyl-(1→3)-2-azido-4-*O*-benzyl-2-deoxy-*β*-D-fucopyranoside (14)**

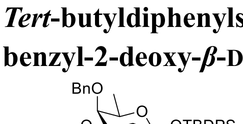

13 (1.34 g, 1.45 mmol) was dissolved in DCM/H$_2$O (14.5 mL, 20:1, 0.1 M), added DDQ (660 mg, 2.91 mmol, 2 equiv.) stirred at rt under N$_2$ for 1.5 h until TLC (Pent/EtOAc 9:1) showed full conversion. The solution was quenched with Na$_2$S$_2$O$_3$ (aq., sat.), dissolved in EtOAc and extracted (x3), and the combined organic phases were washed with sat. aq. NaHCO$_3$ (sat. aq.; x4, until the yellow color disappeared) and brine (x1), dried over Na$_2$SO$_4$, filtered and concentrated *in vacuo*. Column chromatography (Pentane/EtOAc 95:5 → 80:20) gave **14** in 86% yield (976 mg, 1.25 mmol). **^{1}H NMR (400 MHz, CDCl$_3$)** δ 7.83 – 7.69 (m, 4H, Ar-*H*), 7.48 – 7.29 (m, 16H, Ar-*H*), 5.23 (d, *J* = 3.7 Hz, 1H, H-1'), 4.77 (d, *J* = 11.6 Hz, 2H, Ar-C*H*$_2$), 4.69 (dd, *J* = 11.7, 8.5 Hz, 2H, Ar-C*H*$_2$), 4.36 (d, *J* = 7.7 Hz, 1H, H-1), 3.91 (dd, *J* = 10.5, 7.7 Hz, 1H, H-2), 3.86 (dd, *J* = 10.9, 3.4 Hz, 1H, H-4'), 3.83 – 3.77 (m, 1H, H-5'), 3.51 (dd, *J* = 3.4, 1.3 Hz, 1H, H-3'), 3.44 – 3.31 (m, 3H, H-2', H-3, H-4), 3.19 (q, *J* = 7.1, 6.5 Hz, 1H,

H-5), 1.17 (d, *J* = 6.6 Hz, 3H, H-6'), 1.13 (s, 9H, TBDPS-C*H*$_3$), 1.08 (d, *J* = 6.4 Hz, 3H, H-6). **^{13}C NMR (101 MHz, CDCl$_3$)** δ 138.55 (Ar-*C$_q$*), 137.82 (Ar-*C$_q$*), 136.22 (Ar-*C*), 136.03 (Ar-*C*), 133.52 (Ar-*C$_q$*), 133.10 (Ar-*C$_q$*), 129.87 (Ar-*C*), 129.66 (Ar-*C*), 128.80 (Ar-*C*), 128.50 (Ar-*C*), 128.36 (Ar-*C*), 128.32 (Ar-*C*), 127.85 (Ar-*C*), 127.58 (Ar-*C*), 127.30 (Ar-*C*), 100.01 (C-1'), 97.45 (C-1), 79.80 (C-3'), 79.38 (C-4), 78.66 (C-3), 76.20 (Ar-*C*H$_2$), 75.45 (Ar-*C*H$_2$), 70.81 (C-5), 68.45 (C-4'), 67.27 (C-5'), 66.49 (C-2), 60.74 (C-2'), 26.99 (TBDPS-*C*H$_2$), 16.86 (C-6), 16.70 (C-6').**HRMS**: [M+Na]$^+$ calculated for $C_{42}H_{50}N_6O_7SiNa$: 801.34079; found 801.34025

***Tert*-butyldiphenylsilyl (Benzyl (4-*O*-acetyl-2-azido-2-deoxy-3-*O*-(2-naphthylmethyl)-*β*-D-mannopyranosiduronsyl)-(1→3)-2-azido-4-*O*-benzyl-2-deoxy-*α*-L-fucopyranosyl-(1→3)-2-azido-4-*O*-benzyl-2-deoxy-*β*-D-fucopyranoside (9)**

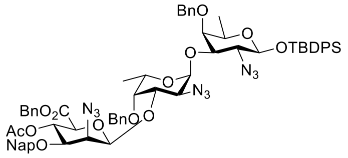

Donor **12b** (1.02 g, 1.54 mmol, 1.5 equiv.) and acceptor **14** (780 mg, 1.00 mmol, 1 equiv.) was co-evaporated with toluene (3x), dissolved in dry DCM (10 mL, 0.1 M), added 3Å molecular sieves and stirred for 30 min. The solution was cooled to -80 ºC and TfOH (18 µL, 0.20 mmol, 0.2 equiv.) was added. The reaction was allowed to warm to -10 ºC and stirred until TLC (Pent/EtOAc 8:2) showed full conversion of the acceptor (~5 h). The reaction was quenched with Et_3N, dissolved in EtOAc, washed with $NaHCO_3$ (sat. aq.; x1) and brine (x1), dried over Na_2SO_4, filtered and concentrated *in vacuo*. Column chromatography (Pentane/EtOAc 95:5 → 80:20) gave **9** in 68% (851 mg, 0.68 mmol) and in a *α*/*β* ratio 15:85. For the *β*-anomer: **^{1}H NMR (400 MHz, CDCl$_3$)** δ 7.83 – 7.68 (m, 8H, Ar-*H*), 7.52 – 7.27 (m, 24H, Ar-*H*), 5.46 (t, *J* = 9.2 Hz, 1H, H-4''), 5.19 (dd, *J* = 12.2, 3.7 Hz, 1H, H-1'), 5.05 (dd, *J* = 12.2, 1.6 Hz, 2H, Ar-C*H*$_2$), 4.83 – 4.71 (m, 3H, Ar-C*H*$_2$), 4.66 (d, *J* = 7.2 Hz, 1H, Ar-C*H*$_2$), 4.63 (d, *J* = 6.9 Hz, 1H, Ar-C*H*$_2$), 4.55 (d, *J* = 1.5 Hz, 1H, H-1''), 4.52 (d, *J* = 11.7 Hz, 1H, Ar-C*H*$_2$), 4.33 (d, *J* = 7.7 Hz, 1H, H-1), 4.16 (dd, *J* = 10.7, 2.9 Hz, 1H, H-3'), 3.91 – 3.86 (m, 1H, H-2), 3.84 (d, *J* = 9.3 Hz, 1H, H-5''), 3.75 – 3.65 (m, 2H, H-5', H-2'), 3.63 (dd, *J* = 3.6, 1.4 Hz, 1H, H-2''), 3.59 (dd, *J* = 9.1, 3.5 Hz, 1H, H-3''), 3.49 (dd, *J* = 3.0, 1.3 Hz, 1H, H-4'), 3.33 (dd, *J* = 10.5, 3.0 Hz, 1H, H-3), 3.15 (q, *J* = 6.3 Hz, 1H, H-4), 3.20 – 3.11 (q, *J* = 6.3 Hz, 1H, H-4), 1.86 (s, 3H, , COC*H*$_3$), 1.11 (s, 9H, TBDPS-C*H*$_3$), 1.07 (d, *J* = 6.6 Hz, 3H, H-6'), 1.05 (d, *J* = 6.3 Hz, 3H, H-6). **^{13}C NMR (101 MHz, CDCl$_3$)** δ 169.32 (*C*=O), 166.55 (*C*=O), 138.81 (Ar-*C$_q$*), 138.11 (Ar-*C$_q$*), 136.24 (Ar-*C*), 136.06 (Ar-*C*), 134.67 (Ar-*C$_q$*), 133.80 (Ar-*C$_q$*), 133.59 (Ar-*C$_q$*), 133.26 (Ar-*C$_q$*), 133.08 (Ar-*C$_q$*), 129.88 (Ar-*C*), 129.65 (Ar-*C*), 128.88 (Ar-*C*), 128.68 (Ar-*C*), 128.64 (Ar-*C*), 128.55 (Ar-*C*), 128.45 *α*(Ar-*C*), 128.32 (Ar-*C*), 128.07 (Ar-*C*), 127.95 (Ar-*C*), 127.92 (Ar-*C*), 127.77 (Ar-*C*), 127.60 (Ar-*C*), 127.30 (Ar-*C*), 126.91 (Ar-*C*), 126.58 (Ar-*C*), 126.42 (Ar-*C*), 125.71 (Ar-*C*), 100.14 (C-1'), 97.61 (C-1''), 97.38 (C-1), 79.42 (C-4), 78.99 (C-3), 77.48 (C-4'), 77.16 (C-4''), 75.57 (Ar-*C*H$_2$), 75.47 (C-3'), 75.05 (Ar-*C*H$_2$), 73.85 (C-5''), 72.45 (Ar-*C*H$_2$), 70.75 (C-5), 68.09 (C-4''), 67.81 (Ar-*C*H$_2$), 67.20 (C-5'), 66.30 (C-2), 61.47 (C-2''), 58.55 (C-2'), 27.00 (TBDPS-*C*H$_3$), 20.79 (CO*C*H$_3$), 16.77 (C-6', C-6). For the *α*-anomer: **^{1}H NMR** (400 MHz, CDCl$_3$) δ 7.87 – 7.69 (m, 13H, Ar-*H*), 7.53 – 7.27 (m, 32H, Ar-*H*), 7.25 – 7.13 (m, 7H, Ar-*H*), 7.07 – 6.98 (m, 2H, Ar-*H*), 5.70 (d, *J* = 7.5 Hz, 1H, H-1), 5.54 (dd, *J* = 4.6, 2.8 Hz, 1H, H-4''), 5.36 (d, *J* = 3.8 Hz, 1H, H-1'), 5.05 (d, *J* = 11.7 Hz, 2H, Ar-C*H*$_2$), 4.94 (d, *J* = 11.6 Hz, 1H, Ar-C*H*$_2$), 4.82 – 4.59 (m, 9H, Ar-C*H*$_2$), 4.59 – 4.49 (m, 2H, Ar-C*H*$_2$, H-5''), 4.40 – 4.33 (m, 2H, H-1'', H-3'), 4.02 – 3.95 (m, 2H, H-2, H-3'), 3.95 – 3.77 (m, 2H, H-2', H-5'), 3.67 (dd, *J* = 7.6, 2.9 Hz, 1H, H-2''), 3.45 – 3.31 (m, 3H, H-3, H-4, H-4'), 3.19 (q, *J* = 6.4 Hz, 2H, H-5), 2.06 (s, 3H, COC*H*$_3$), 1.14 (s, 9H, TBDPS-C*H*$_3$), 1.08 – 1.02 (m, 6H, H-6, H-6'). **^{13}C NMR** ^{13}C NMR (101 MHz, CDCl$_3$) δ 169.81 (*C*=O), 167.38 (*C*=O), 138.73 (Ar-*C$_q$*), 138.52 (Ar-*C$_q$*), 136.24 (Ar-*C*), 136.22 (Ar-*C*), 136.05 (Ar-*C$_q$*), 136.03 (Ar-*C$_q$*), 134.91 (Ar-*C$_q$*), 134.39 (Ar-*C$_q$*), 133.60 (Ar-*C$_q$*), 133.26 (Ar-*C$_q$*), 133.19 (Ar-*C$_q$*), 133.15 (Ar-*C$_q$*), 129.85 (Ar-*C*), 129.63 (Ar-*C*), 128.80 (Ar-*C*), 128.60 (Ar-*C*), 128.56 (Ar-*C*), 128.50 (Ar-*C*), 128.46 (Ar-*C*), 128.41 (Ar-*C*), 128.35 (Ar-*C*), 128.32 (Ar-*C*), 128.30 (Ar-*C*), 128.14 (Ar-*C*), 127.85 (Ar-*C*), 127.82 (Ar-*C*), 127.66 (Ar-*C*), 127.58 (Ar-*C*), 127.29 (Ar-*C*), 126.53 (Ar-*C*),

126.38 (Ar-*C*), 126.21 (Ar-*C*), 125.52 (Ar-*C*), 100.00 (C-1'), 98.91 (C-1"), 97.51 (C-1), 78.77 (C-3, C4, C-4', C-5'), 78.70 (C-3, C4, C-4', C-5'), 78.65 (C-3, C4, C-4', C-5'), 78.32 (C-3'), 75.19 (Ar-CH_2), 75.09 (C-3"), 75.00 (Ar-CH_2), 73.05 (Ar-CH_2), 70.81 (C-5), 68.73 (C-4"), 67.34 (Ar-CH_2), 67.30 (C-5"), 66.45 (C-2), 60.18 (C-2'), 59.40 (C-2"), 27.01 (TBDPS-CH_3), 21.06 ($COCH_3$), 16.74 (C-6', C-6), 16.69 (C-6', C-6). **HRMS**: $[M+Na]^+$ calculated for $C_{68}H_{73}N_9O_{13}SiNa$: 1274.49948; found 1274.49893.

(Benzyl (4-*O*-acetyl-2-azido-2-deoxy-3-*O*-(2-naphthylmethyl)-*β*-D-mannopyranosiduronsyl))-(1→3)-2-azido-4-*O*-benzyl-2-deoxy-*α*-L-fucopyranosyl-(1→3)-2-azido-4-*O*-benzyl-2-deoxy-*α*/*β*-D-fucopyranose (15)

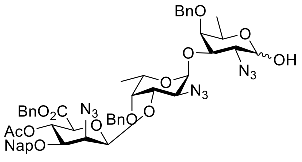

9 (906 mg, 0.72 mmol) was dissolved THF (7.2 mL, 0.1 M) and cooled to 0℃. AcOH (80 µL, 1.45 mmol, 2 equiv.) and TBAF (1 M in THF, 1.44 mL, 1.45 mmol, 2 equiv.) was added and the reaction was allowed to warm to rt under N_2 and stirred overnight (~18 h) until TLC (Pent/EtOAc 3:2) showed full conversion. The reaction was quenched with NH_4Cl (aq. sat.) and dissolved in EtOAc. The organic layer was washed with H_2O (x3) and brine (x1), dried over Na_2SO_4, filtered and concentrated *in vacuo*. Column chromatography (Pentane/EtOAc 7:3 → 5:5) gave **15** in 84% yield (613 mg, 0.60 mmol) as a *α*/*β* mixture. **^{1}H NMR (400 MHz, $CDCl_3$)** δ 7.81 (t, *J* = 8.0 Hz, 6H), 7.74 (d, *J* = 1.7 Hz, 2H), 7.52 – 7.42 (m, 6H), 7.39 – 7.27 (m, 27H), 5.46 (td, *J* = 9.3, 1.0 Hz, 2H), 5.34 (t, *J* = 2.8 Hz, 1H), 5.25 (d, *J* = 3.7 Hz, 1H), 5.21 (d, *J* = 3.6 Hz, 1H), 5.03 (dd, *J* = 3.4, 2.3 Hz, 4H), 4.84 – 4.74 (m, 5H), 4.73 – 4.60 (m, 5H), 4.60 – 4.53 (m, 4H), 4.49 (t, *J* = 6.9 Hz, 1H), 4.28 – 4.21 (m, 1H), 4.20 – 4.13 (m, 1H), 4.06 (dd, *J* = 10.6, 2.7 Hz, 1H), 3.94 (dd, *J* = 10.6, 3.4 Hz, 1H), 3.85 (ddt, *J* = 9.4, 6.2, 3.4 Hz, 4H), 3.77 (tt, *J* = 10.6, 3.4 Hz, 3H), 3.68 – 3.61 (m, 3H), 3.60 (dd, *J* = 3.6, 1.8 Hz, 1H), 3.57 (dq, *J* = 2.5, 1.5 Hz, 3H), 3.55 – 3.51 (m, 2H), 3.48 (dd, *J* = 10.5, 2.8 Hz, 1H), 3.41 (d, *J* = 2.8 Hz, 1H), 3.01 (d, *J* = 2.6 Hz, 1H), 1.84 (s, 6H), 1.27 – 1.20 (m, 6H), 1.14 (dd, *J* = 6.6, 2.5 Hz, 6H). **^{13}C NMR (101 MHz, $CDCl_3$)** δ 169.00, 166.59, 166.53, 138.47, 138.39, 138.05, 138.01, 135.06, 135.02, 134.63, 133.21, 133.19, 128.81, 128.77, 128.65, 128.60, 128.54, 128.50, 128.47, 128.35, 128.33, 128.09, 128.08, 127.98, 127.92, 127.87, 127.77, 126.88, 126.56, 126.54, 126.40, 125.67, 99.94, 99.56, 97.61, 97.53, 96.84, 92.41, 79.37, 79.34, 78.50, 77.27, 76.99, 76.95, 76.80, 75.80, 75.56, 75.48, 75.07, 73.73, 72.44, 72.42, 71.13, 68.04, 67.76, 67.71, 67.58, 67.38, 67.18, 65.20, 61.46, 61.40, 61.17, 59.05, 58.59, 20.74, 17.11, 16.95, 16.77, 16.74. **HRMS**: $[M+Na]^+$ calculated for $C_{52}H_{55}N_9O_{13}Na$: 1036.38170; found 1036.38115

(Benzyl (4-*O*-acetyl-2-azido-2-deoxy-3-*O*-(2-naphthylmethyl)-*β*-D-mannopyranosiduronsyl))-(1→3)-2-azido-4-*O*-benzyl-2-deoxy-*α*-L-fucopyranosyl-(1→3)-2-azido-4-*O*-benzyl-2-deoxy-1-*O*-(*N*-phenyl-2,2,2-trifluoroacetimidoyl)-*α*/*β*-D-fucopyranose (16)

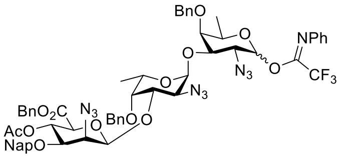

15 (576 g, 0.57 mmol) was co-evaporated with toluene (x3) and dissolved in dry acetone (2.8 mL, 0.2 M). K_2CO_3 (157 mg, 1.14 mmol, 2 equiv.) and ClC(=NPh)CF_3 (0.18 mL, 1.14 mmol, 2 equiv.) was added and the reaction was stirred overnight at rt under N_2 until TLC (Pent/EtOAc 7:3) showed full conversion. The reaction was filtered on Celite and concentrated *in vacuo*. Column chromatography (Pentane/EtOAc 8:2 → 6:4) gave **16** in 93% yield (627 mg, 0.529 mmol). **^{1}H NMR (400 MHz, CD_3CN)** δ 7.92 – 7.78 (m, 4H), 7.56 – 7.42 (m, 4H), 7.44 – 7.27 (m, 20H), 7.19 – 7.09 (m, 1H), 6.94 – 6.82 (m, 2H), 5.55 (bs, 1H), 5.24 (d, *J* = 3.7 Hz, 1H), 5.20 – 5.09 (m, 1H), 5.02 – 4.96 (m, 2H), 4.96 – 4.89 (m, 1H), 4.88 – 4.81 (m, 2H), 4.80 – 4.68 (m, 2H), 4.62 – 4.53 (m, 2H), 4.20 (dd, *J* = 11.2, 2.8 Hz, 1H), 4.11 (d, *J* = 1.4 Hz, 1H), 4.01 – 3.82 (m, 5H), 3.78 – 3.74 (m, 1H), 3.58 (m, 2H), 1.82 (s, 3H), 1.23 – 1.15 (m, 6H). **^{13}C NMR (101 MHz, CD_3CN)** δ 170.44, 167.84, 139.65, 139.59, 136.36, 136.23, 134.06, 133.92, 129.80, 129.49, 129.35, 129.29, 129.26, 129.12, 129.05, 128.70, 128.65, 128.56, 127.59, 127.27, 127.10, 126.88, 125.42, 120.03, 101.13, 97.93,

79.13, 78.83, 78.27, 77.87, 76.64, 76.24, 76.18, 74.17, 72.73, 72.69, 68.93, 68.16, 68.03, 64.34, 62.58, 59.00, 20.88, 16.87, 16.72. **HRMS**: $[M+H]^+$ calculated for $C_{60}H_{59}F_3N_{10}O_{13}H$: 1185.42934; found 1185.42829

5-(Benzyl(benzyloxycarbonyl)amino)pentyl (Benzyl (4-*O*-acetyl-2-azido-2-deoxy-3-*O*-(2-naphthylmethyl)-*β*-D-mannopyranosiduronsyl)-(1→3)-2-azido-4-*O*-benzyl-2-deoxy-*α*-L-fucopyranosyl-(1→3)-2-azido-4-*O*-benzyl-2-deoxy-*α*-D-fucopyranoside (5)

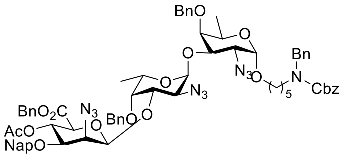

Donor **16** (202 mg, 0.17 mmol, 1 equiv.) and *N*-(Benzyl)-benzyloxycarbonyl-5-aminopentan-1-ol[13] **17** (72 mg, 0.22 mmol, 1.3 equiv.) was co-evaporated with toluene (3x). The donor, acceptor and $Ph_3P{=}O$ (284 mg, 1.021 mmol, 6 equiv.) was dissolved in dry DCM/Et_2O (1.7 mL, 1:1, 0,1 M), added 3Å molecular sieves and stirred for 1 h. The solution was added TMSI (24 µL, 0;17 mmol, 1 equiv.) and stirred for 24 h until TLC (Pent/EtOAc 3:2) showed full conversion. The reaction was quenched with Et_3N, dissolved in EtOAc, washed with $Na_2S_2O_3$ (sat. aq.; x1), $NaHCO_3$ (sat. aq.; x1) and brine (x1), dried over Na_2SO_4, filtered and concentrated *in vacuo*. Column chromatography (Pentane/EtOAc 75:25 → 55:45) gave **5** in 93% yield (209 mg, 0.158 mmol) and in a *α*/*β* ratio 75:25. For the *α*-anomer: **^{1}H NMR (400 MHz, $CDCl_3$)** δ 7.84 – 7.71 (m, 4H, Ar-*H*), 7.53 – 7.41 (m, 3H, Ar-*H*), 7.39 – 7.26 (m, 26H, Ar-*H*), 7.25 – 7.11 (m, 2H, Ar-*H*), 5.46 (t, *J* = 9.1 Hz, 1H, H-4''), 5.23 – 5.13 (m, 3H, H-1', Ar-CH_2), 5.03 (d, *J* = 2.6 Hz, 2H, Ar-CH_2), 4.90 (d, *J* = 7.7 Hz, 1H, H-1), 4.82 – 4.72 (m, 3H, Ar-CH_2), 4.69 – 4.63 (m, 2H, Ar-CH_2), 4.59 – 4.55 (m, 2H, H-1'', Ar-CH_2), 4.54 – 4.45 (m, 3H, CH_2-Linker), 4.21 (dd, *J* = 10.6, 2.8 Hz, 1H, H-3'), 4.03 (d, *J* = 10.8 Hz, 1H, H-3), 3.96 – 3.87 (m, 1H, H-5), 3.87 – 3.77 (m, 4H, H-5', H-2, H-5'', H-2'), 3.67 – 3.61 (m, 1H, H-4), 3.58 (dd, *J* = 9.1, 3.5 Hz, 1H, H-2''), 3.54 (d, *J* = 4.9 Hz, 2H), 3.47 – 3.32 (m, 2H, H-4', H-3''), 3.31 – 3.14 (m, 2H, CH_2-Linker), 1.84 (s, 3H, COCH_3), 1.73 – 1.46 (m, 4H, CH_2-Linker), 1.41 – 1.24 (m, 4H, CH_2-Linker), 1.21 (d, *J* = 6.5 Hz, 3H, H-6), 1.14 (d, *J* = 6.5 Hz, 3H, H-6). **^{13}C NMR (101 MHz, $CDCl_3$)** δ 169.34 (C=O), 166.57 (C=O), 138.61 (Ar-C_q), 138.03 (Ar-C_q), 135.09 (Ar-C_q), 134.66 (Ar-C_q), 133.22 (Ar-C_q), 128.82 (Ar-*C*), 128.67 (Ar-*C*), 128.61 (Ar-*C*), 128.55 (Ar-*C*), 128.51 (Ar-*C*), 128.48 (Ar-*C*), 128.27 (Ar-*C*), 128.06 (Ar-*C*), 127.96 (Ar-*C*), 127.94 (Ar-*C*), 127.90 (Ar-*C*), 127.77 (Ar-*C*), 127.72 (Ar-*C*), 126.91 (Ar-*C*), 126.55 (Ar-*C*), 126.40 (Ar-*C*), 125.70 (Ar-*C*), 99.89 (C-1'), 98.17 (C-1), 97.61 (C-1''), 80.05 (C-4'), 77.48 (C-3''), 76.84 (C-3'), 76.11 (C-3), 75.50 (Ar-*C*H_2), 75.25 (Ar-*C*H_2), 73.76 (C-5'), 72.43 (Ar-*C*H_2), 68.45 (C-4''), 68.05 (Ar-*C*H_2), 67.73 (C-5), 67.46 (Ar-*C*H_2), 66.87 (C-4), 61.40 (C-2''), 60.25 (C-2'), 58.91 (C-2), 29.25 (*C*H_2-Linker), 23.50 (*C*H_2-Linker), 20.76 (CO*C*H_3), 16.96 (C-6, C-6'), 16.80 (C-6, C-6'). For the *β*-anomer: **^{1}H NMR (400 MHz, $CDCl_3$)** δ 7.85 – 7.78 (m, 3H, Ar-*H*), 7.74 (d, *J* = 1.7 Hz, 1H, Ar-*H*), 7.51 – 7.41 (m, 3H, Ar-*H*), 7.37 – 7.26 (m, 24H, Ar-*H*), 7.17 (s, 1H, Ar-*H*), 5.46 (t, *J* = 9.2 Hz, 1H, H-4''), 5.25 (d, *J* = 3.8 Hz, 1H, H-1'), 5.21 – 5.13 (m, 3H, Ar-*H*), 5.04 (d, *J* = 2.5 Hz, 2H, Ar-*H*), 4.83 – 4.68 (m, 4H, Ar-*H*), 4.58 – 4.52 (m, 2H, Ar-*H*, H-1''), 4.49 (d, *J* = 6.9 Hz, 2H, CH_2-Linker), 4.17 (m, 2H, H-1, H-3'), 3.87 – 3.80 (m, 2H, H-2, H-5''), 3.70 (td, *J* = 10.9, 5.2 Hz, 2H, H-2', H-5'), 3.64 (dt, *J* = 3.6, 1.6 Hz, 1H, H-2''), 3.59 (ddd, *J* = 9.0, 3.5, 1.8 Hz, 1H, H-3''), 3.57 – 3.47 (m, 2H, H-4', H-5), 3.44 (dd, *J* = 10.5, 2.8 Hz, 1H, H-4), 3.40 – 3.36 (m, 1H, CH_2-Linker), 3.33 – 3.15 (m, 3H, CH_2-Linker), 1.85 (d, *J* = 2.4 Hz, 3H, COCH_3), 1.27 (d, *J* = 2.9 Hz, 3H, H-6, H-6'), 1.12 (d, *J* = 6.7 Hz, 3H, H-6, H-6'). **^{13}C NMR (101 MHz, $CDCl_3$)** δ 169.32 (C=O), 166.48 (C=O), 138.61 (Ar-C_q), 138.05 (Ar-C_q), 135.05 (Ar-C_q), 134.65 (Ar-C_q), 133.23 (Ar-C_q), 128.83 (Ar-*C*), 128.80 (Ar-*C*), 128.66 (Ar-*C*), 128.63 (Ar-*C*), 128.61 (Ar-*C*), 128.54 (Ar-*C*), 128.47 (Ar-*C*), 128.39 (Ar-*C*), 128.31 (Ar-*C*), 128.26 (Ar-*C*), 128.07 (Ar-*C*), 127.94 (Ar-*C*), 127.89 (Ar-*C*), 127.76 (Ar-*C*), 127.63 (Ar-*C*), 127.37 (Ar-*C*), 126.88 (Ar-*C*), 126.56 (Ar-*C*), 126.41 (Ar-*C*), 125.68 (Ar-*C*), 102.72 (C-1), 100.21 (C-1'), 97.56 (C-1''), 79.35 (C-3), 78.85 (C-4), 77.39 (C-4', C-5), 77.01 (C-3''), 75.59 (C-Ar-*C*H_2), 75.33 (C-3'), 75.14 (Ar-*C*H_2), 73.80 (C-5''), 72.44 (Ar-*C*H_2), 70.78 (C-4', C-5), 68.06 (C-5'), 67.76 (Ar-*C*H_2), 67.25 (Ar-*C*H_2), 67.20 (C-2), 63.65 (C-2''), 61.47 (C-2'), 58.43

(CH_2-Linker), 50.66 (CH_2-Linker), 50.26 (CH_2-Linker), 29.88 (CH_2-Linker), 29.22 (CH_2-Linker), 27.92, (CH_2-Linker) 27.47 (CH_2-Linker), 20.75 (COCH_3), 17.13 (C-6, C-6'), 16.78 (C-6, C-6'). **HRMS**: $[M+Na]^+$ calculated for $C_{72}H_{76}N_{10}O_{15}Na$: 1345.55458; found 1345.55403

5-aminopentyl 2-acetamide-4-*O*-acetyl-2-deoxy-*β*-D-mannopyranosiduronsyl-(1→3)-2-acetamide-2-deoxy-*α*-L-fucopyranosyl-(1→3)-2-acetamide-2-deoxy-*α*-D-fucopyranoside (1)

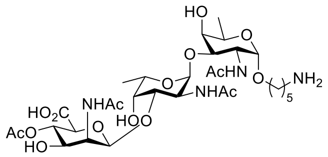

5 (59 mg, 0.0445 mmol) was deprotected following the general experimental for the deprotection yielding **1** in 45% yield over two steps (14.3 mg, 0.0198 mol). **^{1}H NMR (600 MHz, D_2O)** δ 5.00 – 4.95 (m, 2H, H-1', H-4"), 4.91 (d, *J* = 1.4 Hz, 1H, H-1"), 4.74 (d, *J* = 3.8 Hz, 1H, H-1), 4.47 (dd, *J* = 4.3, 1.4 Hz, 1H, H-2"), 4.23 (dd, *J* = 11.1, 3.8 Hz, 1H, H-2), 4.18 – 4.09 (m, 2H, H-3', H-2'), 4.07 (q, *J* = 7.7, 7.1 Hz, 1H, H-5), 4.05 – 4.01 (m, 1H, H-5'), 4.01 – 3.97 (m, 2H, H-4', H-3"), 3.89 (dd, *J* = 11.1, 3.2 Hz, 1H, H-3), 3.77 (d, *J* = 3.3 Hz, 1H, H-4), 3.75 (d, *J* = 10.1 Hz, 1H, H-5"), 3.62 (dt, *J* = 9.9, 6.5 Hz, 1H, CH_2-linker), 3.41 (dt, *J* = 10.0, 6.2 Hz, 1H, CH_2-linker), 2.95 (dd, *J* = 8.7, 6.7 Hz, 2H, CH_2-linker), 2.08 (s, 3H, $COCH_3$), 2.03 (s, 3H, $COCH_3$), 2.02 (s, 3H, $COCH_3$), 1.94 (s, 3H, COCH_3), 1.68 – 1.55 (m, 4H, CH_2-linker), 1.40 (tq, *J* = 14.4, 7.4, 6.5 Hz, 2H, CH_2-linker), 1.24 – 1.15 (m, 6H, H-6, H-6'). **^{13}C NMR (151 MHz, D_2O)** δ 176.54 (C=O), 175.73 (C=O), 174.93 (C=O), 174.67 (C=O), 173.87 (C=O), 99.91 (C-1'), 97.89 (C-1), 95.50 (C-1"), 75.30 (C-3'), 75.20 (C-3), 73.53 (C-5"), 72.14 (C-4), 71.31 (C-4"), 70.70 (C-3"), 68.64 (CH_2-linker), 68.38 (C-4'), 67.74 (C-5), 67.28 (C-5'), 54.12 (C-2"), 49.61 (C-2), 48.54 (C-2'), 40.27 (CH_2-linker), 28.95 (CH_2-linker), 27.41 (CH_2-linker), 23.23 (CH_2-linker), 23.17 (COCH_3), 22.85 (COCH_3), 22.79 (COCH_3), 21.25(COCH_3), 16.42 (C-6), 16.23 (C-6'). **HRMS**: $[M+H]^+$ calculated for $C_{31}H_{52}N_4O_{16}H$: 737.34566; found 737.34407

Synthesis of Longer fragments

5-(Benzyl(benzyloxycarbonyl)amino)pentyl (Benzyl (4-*O*-acetyl-2-azido-2-deoxy-*β*-D-mannopyranosiduronsyl)-(1→3)-2-azido-4-*O*-benzyl-2-deoxy-*α*-L-fucopyranosyl-(1→3)-2-azido-4-*O*-benzyl-2-deoxy-3-*O*-(2-naphthylmethyl)-*α*-D-fucopyranoside (18)

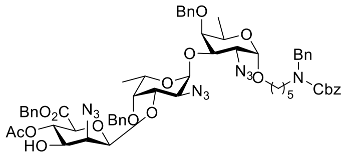

The 2-methylnaphthyl was cleaved from **5** (170 mg, 0.128 mmol, 1 equiv.) using the general experimental procedure for deprotection of the 2-methylnaphthyl group in DCM/H_2O (1.3 mL, 20:1, 0.1 M) with DDQ (58 mg, 0.256 mmol, 2 equiv.). The reaction was followed by TLC (Pent/EtOAc 3:2) and purification by column chromatography (Pentane/EtOAc 6:4 → 5:5) gave **18** in 80% yield (121 mg, 0.102 mmol). **^{1}H NMR (400 MHz, $CDCl_3$)** δ 7.42 – 7.26 (m, 25H), 5.23 (d, *J* = 3.7 Hz, 1H), 5.21 – 5.10 (m, 4H), 5.06 (s, 2H), 4.90 (d, *J* = 7.0 Hz, 1H), 4.76 (t, *J* = 12.5 Hz, 1H), 4.72 – 4.61 (m, 4H), 4.49 (d, *J* = 7.2 Hz, 2H), 4.21 (dd, *J* = 10.6, 2.9 Hz, 1H), 4.05 (d, *J* = 11.0 Hz, 1H), 3.93 (m, 1H), 3.90 – 3.80 (m, 3H), 3.75 (dd, *J* = 10.6, 3.6 Hz, 1H), 3.68 – 3.51 (m, 6H), 3.49 – 3.32 (m, 1H), 3.23 (m, 4H), 2.63 (d, *J* = 9.9 Hz, 1H), 1.86 (s, 3H), 1.64 – 1.45 (m, 5H), 1.40 – 1.25 (m, 4H), 1.21 (t, *J* = 6.1 Hz, 8H). **^{13}C NMR (101 MHz, $CDCl_3$)** δ 170.59, 166.29, 138.66, 138.02, 137.89, 135.14, 128.79, 128.69, 128.67, 128.59, 128.51, 128.34, 128.29, 128.06, 127.96, 127.93, 127.87, 127.73, 127.38, 99.90, 98.20, 98.00, 80.09, 76.84, 75.79, 75.33, 73.75, 71.12, 69.80, 68.31, 67.69, 67.43, 67.28, 66.88, 64.02, 60.20, 58.58, 50.76, 50.44, 47.09, 29.73, 29.23, 23.47, 20.65, 16.98, 16.86. **HRMS**: $[M+Na]^+$ calculated for $C_{61}H_{70}N_{10}O_{15}Na$: 1205.49198; found 1205.49143

Hexasaccharide protected (6)

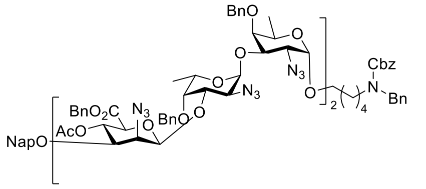

The glycosylation was performed using the general glycosylation procedure with donor **16** (142 mg, 0.120 mmol, 1.3 equiv.) and acceptor **18** (109 mg, 0.0921 mmol, 1 equiv.) dissolved in dry DCM (4.6 mL, 0.02 M) and added TBSOTf (4 µL, 0.0184 mmol, 0.2 equiv.). The reaction was followed with TLC (Pent/EtOAc 1:1) and purification by column chromatography (Pentane/EtOAc 7:3 → 5:5) and size exclusion gave **6** in 87% yield (175 mg, 0.0803 mmol). **^{1}H NMR (500 MHz, $CDCl_3$)** δ 7.85 – 7.70 (m, 5H), 7.51 – 7.41 (m, 4H), 7.39 – 7.26 (m, 45H), 7.21 – 7.14 (m, 2H), 5.45 (t, *J* = 9.2 Hz, 1H), 5.33 (t, *J* = 9.9 Hz, 1H), 5.24 (d, *J* = 3.7 Hz, 1H), 5.22 – 5.14 (m, 4H), 5.03 – 4.99 (m, 4H), 4.95 (d, *J* = 2.5 Hz, 1H), 4.94 – 4.87 (m, 2H), 4.84 – 4.77 (m, 4H), 4.75 – 4.72 (m, 1H), 4.71 – 4.64 (m, 3H), 4.64 – 4.54 (m, 6H), 4.53 – 4.45 (m, 3H), 4.26 (td, *J* = 10.6, 2.9 Hz, 2H), 4.20 (q, *J* = 5.9, 5.3 Hz, 1H), 4.10 – 4.01 (m, 1H), 3.96 (t, *J* = 1.9 Hz, 2H), 3.95 – 3.89 (m, 2H), 3.89 – 3.79 (m, 8H), 3.79 – 3.74 (m, 1H), 3.64 (dd, *J* = 3.5, 1.4 Hz, 1H), 3.62 – 3.51 (m, 8H), 3.49 – 3.35 (m, 1H), 3.23 (m, 2H), 1.87 (s, 3H), 1.83 (s, 4H), 1.70 – 1.47 (m, 6H), 1.40 – 1.24 (m, 5H), 1.24 – 1.11 (m, 14H). **^{13}C NMR (126 MHz, $CDCl_3$)** δ 169.31, 169.17, 166.52, 166.14, 138.57, 138.37, 138.12, 138.03, 137.92, 135.06, 135.03, 134.64, 133.24, 133.22, 128.84, 128.77, 128.66, 128.63, 128.61, 128.55, 128.53, 128.51, 128.41, 128.27, 128.23, 128.19, 128.05, 127.95, 127.92, 127.89, 127.87, 127.77, 127.38, 126.87, 126.54, 126.39, 125.67, 100.76, 99.87, 99.59, 98.18, 97.39, 97.35, 79.96, 79.64, 79.47, 76.77, 76.35, 75.50, 75.33, 75.29, 74.88, 73.97, 73.73, 72.61, 72.36, 68.52, 68.06, 67.74, 67.67, 67.64, 67.51, 66.89, 63.70, 61.27, 61.01, 60.28, 58.98, 58.54, 50.59, 50.26, 47.22, 46.26, 29.22, 23.46, 20.72, 20.48, 17.05, 16.95, 16.91, 16.74. **HRMS**: $[M+Na]^+$ calculated for $C_{113}H_{123}N_{19}O_{27}Na$: 2201.87670; found 2201.87586

CP8-hexasaccahride (2)

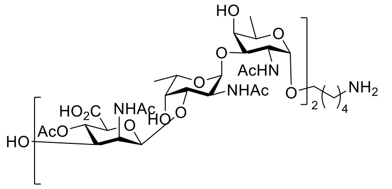

6 (60 mg, 0.0275 mmol) was deprotected following the general experimental for the deprotection yielding the **2** in 37% yield over two steps (8.2 mg, 0.00598 mg). **^{1}H NMR (600 MHz, D_2O)** δ 5.13 (t, *J* = 10.0 Hz, 1H, H^{3}-4), 5.03 – 4.95 (m, 2H, H^{6}-4, H^{2}-1), 4.95 – 4.87 (m, 4H, H^{3}-1, H^{6}-1, H^{4}-1,H H^{5}-1,1), 4.75 (d, *J* = 3.9 Hz, 1H, H^{1}-1), 4.50 – 4.44 (m, 2H, H^{6}-2, H^{3}-2), 4.27 – 4.21 (m, 2H, H^{5}-2, H^{4}-5), 4.24 – 4.17 (m, 1H, H^{1}-2), 4.18 – 4.09 (m, 4H, H^{1}-2, H^{5}-4, H^{2}-4, H^{3}-3), 4.07 (ddd, *J* = 20.1, 13.9, 7.2 Hz, 2H, H^{1}-5, H^{5}-5), 4.04 – 3.98 (m, 4H, H^{3}-5, H^{5}-3, H^{2}-3, H^{6}-3), 3.90 (dd, *J* = 10.8, 3.4 Hz, 1H, H^{1}-3), 3.78 (d, *J* = 3.8 Hz, 1H, H^{1}-4), 3.75 (dd, *J* = 10.1, 1.4 Hz, 2H, H^{6}-5, H^{3}-5), 3.73 (d, *J* = 2.6 Hz, 1H, H^{4}-4), 3.70 (dd, *J* = 10.9, 3.1 Hz, 1H H^{1}-4), 3.67 – 3.59 (m,1H, CH_2-Linker), 3.43 (dt, *J* = 10.0, 6.2 Hz, 1H, CH_2-Linker), 3.00 – 2.93 (m, 2H, CH_2-Linker), 2.09 (s, 3H, COCH_3), 2.06 – 1.99 (m, 12H, COCH_3), 1.95 (s, 3H, COCH_3), 1.91 (s, 3H, COCH_3), 1.70 – 1.55 (m, 4H, CH_2-Linker), 1.45 – 1.36 (m, 2H, CH_2-Linker), 1.25 – 1.16 (m, 12H, H^{1}-6, H^{2}-6, H^{4}-6, H^{5}-6). **^{13}C NMR (151 MHz, D_2O)** δ 176.49 (C=O), 175.92 (C=O), 175.71 (C=O), 175.44 (C=O), 174.90 (C=O), 174.87 (C=O), 174.64 (C=O), 174.49 (C=O), 173.86 (C=O), 173.32 (C=O), 99.92 (C^{2}-1), 99.70 (C^{4}-1/ C^{5}-1), 99.43 (C^{4}-1/ C^{5}-1), 97.88 (C^{1}-1), 95.37 (C^{3}-1/ C^{6}-1), 95.24 (C^{3}-1/ C^{6}-1), 75.28 (C^{3}-5/ C^{6}-5), 75.19 (C^{3}-5/ C^{6}-5), 74.93, 74.87, 74.56, 73.48, 73.38, 72.13, 71.91, 71.30, 71.19, 70.71, 68.62, 68.38, 68.30, 67.73, 67.69, 67.58, 67.27, 54.10, 53.42, 49.59, 49.38, 48.48, 48.40, 40.26, 28.93, 27.39, 23.21, 23.13, 22.92, 22.82, 22.77, 22.70, 21.24, 21.11, 16.44, 16.40, 16.22, 16.17. **HRMS**: $[M+H]^+$ calculated for $C_{57}H_{92}N_7O_{31}H$: 1370.58377; found 1370.58302

Hexasaccharide-acceptor (19)

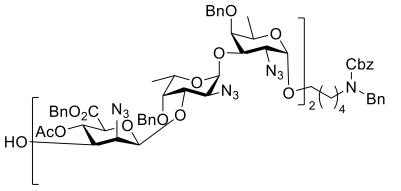

The 2-methylnaphthyl was cleaved from **6** (179 mg, 0.0820 mmol) using the general experimental procedure for deprotection of the 2-methylnaphthyl group in DCM/H_2O (4.1 mL, 20:1, 0.02 M) with DDQ (37 mg, 0.164 mmol, 2 equiv.). The reaction was followed by TLC (Pent/EtOAc 3:2) and purification by column chromatography (Pentane/EtOAc 6:4 → 5:5) gave **19** in 54% yield (90 mg, 0.0441 mmol). **^{1}H NMR (500 MHz, CDCl$_3$)** δ 7.40 – 7.26 (m, 39H), 7.22 – 7.14 (m, 2H), 5.33 (t, *J* = 9.8 Hz, 2H), 5.24 (d, *J* = 3.7 Hz, 1H), 5.22 – 5.11 (m, 4H), 5.02 (d, *J* = 5.6 Hz, 4H), 4.96 (d, *J* = 3.1 Hz, 1H), 4.90 (d, *J* = 9.8 Hz, 1H), 4.80 (dd, *J* = 11.6, 9.5 Hz, 2H), 4.75 – 4.61 (m, 7H), 4.59 – 4.55 (m, 1H), 4.49 (d, *J* = 9.1 Hz, 2H), 4.30 – 4.17 (m, 3H), 4.05 (d, *J* = 11.1 Hz, 1H), 3.98 (dd, *J* = 4.3, 2.6 Hz, 2H), 3.95 – 3.89 (m, 1H), 3.89 – 3.82 (m, 5H), 3.80 (d, *J* = 6.5 Hz, 1H), 3.80 – 3.74 (m, 2H), 3.65 – 3.53 (m, 8H), 3.48 – 3.34 (m, 1H), 3.34 – 3.16 (m, 2H), 2.58 (d, *J* = 9.9 Hz, 1H), 1.87 (s, 3H), 1.84 (s, 3H), 1.59 (m, 10H), 1.36 – 1.25 (m, 5H), 1.21 (td, *J* = 10.9, 10.4, 5.4 Hz, 12H). **^{13}C NMR (126 MHz, CDCl$_3$)** δ 170.52, 169.18, 166.29, 166.15, 138.58, 138.43, 138.04, 137.92, 135.14, 135.04, 128.78, 128.72, 128.70, 128.67, 128.66, 128.63, 128.60, 128.55, 128.54, 128.51, 128.30, 128.29, 128.19, 128.03, 127.96, 127.93, 127.87, 127.77, 127.39, 100.76, 99.86, 99.66, 98.19, 97.80, 97.39, 79.96, 79.64, 79.58, 76.91, 76.58, 76.39, 75.64, 75.51, 75.38, 75.29, 75.01, 74.90, 73.98, 73.72, 71.09, 69.82, 68.54, 68.31, 67.75, 67.63, 67.60, 67.52, 67.27, 66.99, 66.90, 63.92, 63.71, 60.99, 60.29, 58.70, 58.57, 50.68, 50.34, 29.83, 29.23, 28.08, 27.43, 22.79, 20.61, 20.01, 17.07, 16.95, 16.91, 16.82. **HRMS**: [M+Na]$^+$ calculated for $C_{102}H_{115}N_{19}O_{27}Na$: 2061.81410; found 2061.81322

Nonasaccharide protected (7)

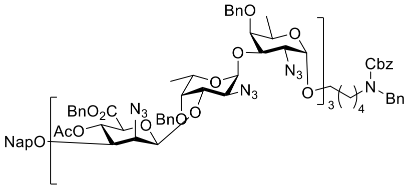

The glycosylation was performed using the general glycosylation procedure with donor **16** (66 mg, 0.0564 mmol, 1.3 equiv.) and acceptor **19** (91 mg, 0.0446 mmol, 1 equiv.) dissolved in dry DCM (2.2 mL, 0.02 M) and added TBSOTf ((2 µL, 0.00892 mmol, 0.2 equiv.) The reaction was followed with TLC (Pent/EtOAc 1:1) and purification by size exclusion to give **7** in 77% yield (104 mg, 0.0342 mmol). **^{1}H NMR (500 MHz, CDCl$_3$)** δ 7.83 – 7.71 (m, 5H), 7.50 – 7.41 (m, 4H), 7.40 – 7.27 (m, 52H), 7.22 – 7.13 (m, 2H), 5.44 (t, *J* = 9.2 Hz, 1H), 5.35 – 5.28 (m, 3H), 5.24 (d, *J* = 3.7 Hz, 1H), 5.21 – 5.12 (m, 4H), 5.03 – 4.99 (m, 4H), 4.98 (s, 2H), 4.97 – 4.92 (m, 2H), 4.90 (d, *J* = 8.6 Hz, 1H), 4.84 – 4.77 (m, 4H), 4.75 – 4.67 (m, 5H), 4.67 – 4.53 (m, 9H), 4.49 (d, *J* = 8.9 Hz, 2H), 4.30 – 4.22 (m, 3H), 4.22 – 4.16 (m, 2H), 4.05 (d, *J* = 10.9 Hz, 1H), 4.00 – 3.94 (m, 4H), 3.93 – 3.71 (m, 14H), 3.66 – 3.50 (m, 12H), 3.48 – 3.33 (m, 2H), 3.33 – 3.14 (m, 2H), 1.87 (s, 5H), 1.85 (s, 3H), 1.82 (s, 4H), 1.66 – 1.43 (m, 9H), 1.27 – 1.12 (m, 21H). **^{13}C NMR (126 MHz, CDCl$_3$)** δ 169.31, 169.20, 166.54, 166.17, 166.14, 138.59, 138.39, 138.37, 138.14, 137.93, 135.08, 135.05, 135.03, 134.66, 133.25, 133.24, 128.78, 128.74, 128.69, 128.67, 128.64, 128.62, 128.56, 128.53, 128.51, 128.31, 128.28, 128.24, 128.20, 128.19, 128.06, 128.04, 127.96, 127.94, 127.92, 127.90, 127.87, 127.77, 127.38, 126.88, 126.55, 126.40, 125.68, 100.78, 99.87, 99.60, 98.20, 97.43, 97.38, 97.25, 79.98, 79.64, 79.59, 79.51, 79.44, 77.16, 76.37, 75.52, 75.35, 75.30, 73.99, 73.92, 73.75, 72.38, 68.54, 68.09, 67.75, 67.68, 67.65, 67.53, 67.02, 66.91, 63.73, 63.63, 61.31, 61.00, 60.30, 59.01, 58.74, 58.58, 29.24, 23.56, 23.44, 20.73, 20.49, 20.46, 17.07, 17.05, 16.96, 16.91, 16.84, 16.74. **HRMS**: [M+Na]$^+$ calculated for $C_{154}H_{168}N_{28}O_{39}Na$: 3056.19212; found 1528.12675

CP8-Nonasaccharide (3)

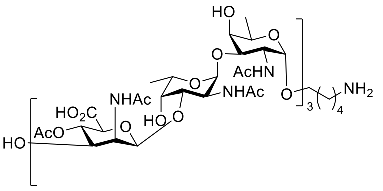

7 (26 mg, 0.0084 mmol, 1 equiv.) was deprotected following the general experimental for the deprotection yielding **3** in 57% yield over two steps (9.7 mg,0.0048 mmol). **^{1}H NMR (850 MHz, D$_2$O)** δ 5.21 – 5.12 (m, 2H), 5.06 – 4.99 (m, 3H), 4.97 (dt, *J* = 11.1, 6.4 Hz, 5H), 4.54 – 4.49 (m, 2H), 4.30 – 4.21 (m, 5H), 4.21 – 4.12 (m, 7H), 4.11 (t, *J* = 6.4 Hz, 2H), 4.10 – 4.00 (m, 7H), 3.96 – 3.85 (m, 5H), 3.81 (d, *J* = 3.2 Hz, 1H), 3.80 – 3.70 (m, 3H), 3.67 (dt, *J* = 10.5, 6.5 Hz, 1H), 3.47 (dt, *J* = 12.1, 6.3 Hz, 1H), 3.00 (t, *J* = 7.7 Hz, 2H), 2.14 (s, 3H), 2.09 – 2.05 (m, 20H), 2.04 (t, *J* = 2.8 Hz, 4H), 1.99 (s, 4H), 1.95 (d, *J* = 5.8 Hz, 5H), 1.66 (ddq, *J* = 43.9, 14.0, 7.4 Hz, 4H), 1.44 (dt, *J* = 16.6, 7.6 Hz, 2H), 1.28 – 1.21 (m, 18H). **^{13}C NMR (214 MHz, D$_2$O)** δ 176.48, 175.91, 174.82, 174.66, 174.48, 174.07, 173.96, 173.41, 99.90, 99.70, 99.64, 99.48, 97.87, 95.83, 95.72, 95.53, 95.49, 76.79, 75.19, 74.76, 74.60, 74.54, 74.48, 74.33, 74.20, 73.84, 73.68, 72.11, 71.87, 71.07, 70.98, 70.53, 68.63, 68.46, 68.42, 68.39, 67.80, 67.74, 67.69, 67.61, 67.27, 53.99, 53.32, 51.46, 49.56, 49.33, 48.48, 48.44, 48.41, 48.37, 40.25, 28.91, 27.37, 23.19, 23.17, 23.13, 22.90, 22.82, 22.78, 22.72, 22.53, 21.17, 21.12, 21.05, 16.40, 16.36, 16.33, 16.19, 16.14. **HRMS**: [M+H]$^+$ calculated for $C_{83}H_{130}N_{10}O_{46}H$: 2003.82189; found 2003.82134

Nona-acceptor (20)

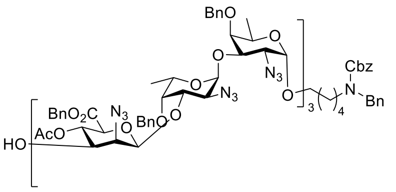

The 2-methylnaphthyl was cleaved from **7** (38.3 mg, 0.0126 mmol) using the general experimental procedure for deprotection of the 2-methylnaphthyl group in DCM/H_2O (1.3 mL, 10:1, 0.01 M) with DDQ (6 mg, 0.0252 mmol, 2 equiv.). The reaction was followed by TLC (Pent/EtOAc 1:1) and purification by column chromatography (Pentane/EtOAc 60:40 → 45:55) gave **20** in 57% yield (20.9 mg, 0.0072 mmol). **^{1}H NMR (850 MHz, CDCl$_3$)** δ 5.21 – 5.14 (m, 2H), 5.05 – 5.01 (m, 2H), 4.99 – 4.93 (m, 5H), 4.54 – 4.49 (m, 2H), 4.30 – 4.21 (m, 5H), 4.21 – 4.15 (m, 7H), 4.14 – 4.09 (m, 2H), 4.09 – 4.01 (m, 6H), 3.96 – 3.85 (m, 5H), 3.81 (d, *J* = 3.2 Hz, 1H), 3.78 – 3.71 (m, 3H), 3.67 (dt, *J* = 10.5, 6.5 Hz, 1H), 3.47 (dt, *J* = 12.1, 6.3 Hz, 1H), 3.00 (t, *J* = 7.7 Hz, 2H), 2.14 (s, 3H), 2.09 – 2.05 (m, 20H), 2.04 (d, *J* = 3.4 Hz, 6H), 1.99 (s, 3H), 1.95 (s, 5H), 1.66 (ddq, *J* = 43.9, 14.0, 7.4 Hz, 4H), 1.44 (dt, *J* = 16.6, 7.6 Hz, 2H), 1.29 – 1.20 (m, 20H). **^{13}C NMR (214 MHz, D$_2$O)** δ 176.48, 175.91, 174.83, 174.66, 174.48, 174.07, 173.96, 173.41, 99.90, 99.70, 99.64, 99.56, 99.48, 97.87, 95.83, 95.72, 95.53, 95.49, 76.79, 75.19, 74.76, 74.60, 74.54, 74.32, 74.20, 73.84, 73.68, 72.11, 71.87, 71.07, 70.98, 70.53, 68.63, 68.46, 68.42, 68.39, 67.80, 67.74, 67.69, 67.61, 67.27, 53.99, 53.32, 51.46, 49.56, 49.33, 48.48, 48.44, 48.41, 48.37, 40.25, 28.91, 27.37, 23.19, 23.17, 23.13, 22.90, 22.82, 22.78, 22.72, 22.53, 21.17, 21.12, 21.05, 16.40, 16.36, 16.33, 16.19, 16.14. **HRMS**: [M+Na]$^+$ calculated for $C_{143}H_{160}N_{28}O_{39}Na$: 2916.12952; found 1458.09829

Dodeca-saccharide (8)

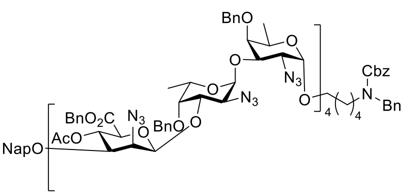

The glycosylation was performed using the general glycosylation procedure with donor **16** (13 mg, 0.0104 mmol, 1.5 equiv.) and acceptor **20** (20 mg, 0.00691 mmol, 1 equiv.) dissolved in dry DCM (1 mL, 0.007 M) and added (TBSOTf 0.3 μL, 0.00138 mmol, 0.2 equiv.) The reaction was followed with TLC (Pent/EtOAc 6:4) and purification by size exclusion to give **8** in 68% yield (18.3 mg, 0.0047 mmol). **^{1}H NMR (850 MHz, CDCl$_3$)** δ 7.83 – 7.77 (m, 3H), 7.72 (s, 1H), 7.50 – 7.40 (m, 4H), 7.40 – 7.27 (m, 61H), 7.23 – 7.13 (m, 3H), 5.43 (t, *J* = 9.1 Hz, 1H), 5.34 – 5.27 (m, 3H), 5.23 (t, *J* = 4.5 Hz, 1H), 5.19 – 5.11 (m, 5H), 5.02 – 4.87 (m, 12H), 4.82 – 4.75 (m, 5H), 4.75 – 4.62 (m, 9H), 4.62 – 4.55 (m, 5H), 4.55 – 4.44 (m, 6H), 4.28 – 4.13 (m, 7H), 4.04 (t, *J* = 10.9 Hz, 1H), 4.01 – 3.90 (m, 7H), 3.90 – 3.66 (m, 18H), 3.66 – 3.48 (m,

15H), 3.47 – 3.32 (m, 2H), 3.25 (dt, J = 27.9, 7.2 Hz, 1H), 3.21 – 3.10 (m, 1H), 1.85 (d, J = 6.9 Hz, 3H), 1.82 (d, J = 2.3 Hz, 6H), 1.81 (d, J = 4.7 Hz, 3H), 1.72 – 1.43 (m, 12H), 1.40 – 1.02 (m, 40H). **^{13}C NMR (214 MHz, $CDCl_3$)** δ 170.59, 169.41, 169.25, 166.51, 166.37, 166.24, 166.13, 166.10, 166.09, 156.83, 156.28, 138.56, 138.53, 138.47, 138.43, 138.35, 138.28, 138.23, 138.21, 138.00, 137.96, 137.91, 137.79, 137.78, 137.72, 136.94, 136.91, 136.78, 135.11, 135.01, 134.92, 134.89, 134.86, 134.53, 133.15, 133.09, 129.53, 128.94, 128.86, 128.84, 128.79, 128.71, 128.67, 128.64, 128.62, 128.59, 128.57, 128.55, 128.53, 128.51, 128.44, 128.41, 128.36, 128.29, 128.26, 128.10, 128.08, 128.02, 127.93, 127.91, 127.89, 127.80, 127.78, 127.69, 127.61, 127.42, 127.36, 127.28, 126.93, 126.56, 126.42, 125.69, 100.76, 99.96, 99.91, 99.68, 98.08, 97.32, 97.24, 97.22, 97.19, 96.99, 79.86, 79.56, 79.50, 79.37, 79.30, 79.29, 76.82, 76.69, 76.59, 76.55, 76.29, 76.07, 75.67, 75.58, 75.50, 75.35, 75.33, 75.30, 74.75, 74.71, 74.38, 74.33, 73.82, 73.74, 73.55, 72.26, 68.25, 68.20, 67.82, 67.77, 67.73, 67.70, 67.69, 67.61, 67.55, 67.28, 67.24, 67.22, 67.19, 66.80, 66.70, 63.59, 63.48, 60.90, 60.87, 60.21, 60.18, 58.73, 58.43, 58.33, 57.89, 50.51, 50.20, 47.11, 46.54, 46.23, 46.17, 32.06, 29.84, 29.80, 29.52, 29.23, 29.16, 27.94, 27.53, 23.46, 23.38, 23.27, 23.20, 22.85, 20.78, 20.51, 20.48, 17.13, 17.06, 17.05, 16.95, 16.93, 16.83, 16.79.

CP8-Dodeca (4)

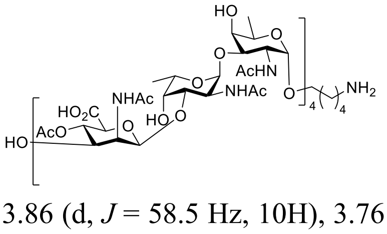

8 (12.2 mg, 0.00299 mmol, 1 equiv.) was deprotected following the general experimental for the deprotection yielding **4** in 33% yield over two steps (2.65 mg, 0.0010 mmol). **^{1}H NMR (850 MHz, D_2O)** δ 5.26 (s, 2H), 5.17 (d, J = 10.2 Hz, 3H), 5.08 (s, 2H), 5.07 – 4.89 (m, 10H), 4.60 (s, 1H), 4.50 (t, J = 20.3 Hz, 4H), 4.35 – 3.96 (m, 28H), 3.86 (d, J = 58.5 Hz, 10H), 3.76 (s, 4H), 3.70 – 3.59 (m, 2H), 3.46 (t, J = 5.2 Hz, 1H), 3.00 (t, J = 7.7 Hz, 2H), 2.15 – 1.92 (m, 49H), 1.66 (dq, J = 39.1, 7.6 Hz, 5H), 1.50 – 1.36 (m, 2H), 1.28 – 1.20 (m, 25H). **^{13}C NMR (302 MHz, D_2O)** δ 99.28, 99.00, 98.59, 98.58, 96.98, 74.47, 73.94, 73.50, 72.64, 71.64, 70.99, 70.22, 69.76, 69.16, 68.82, 67.52, 67.32, 66.86, 66.80, 66.74, 66.71, 66.37, 54.30, 53.16, 52.49, 50.62, 48.69, 48.45, 48.17, 47.55, 39.37, 28.01, 26.47, 22.37, 22.24, 22.03, 21.94, 21.89, 21.83, 21.63, 20.32, 20.25, 20.19, 20.14, 15.51, 15.47, 15.42, 15.35, 15.31, 15.25. **HRMS**: $[M+2H]^+$ calculated for $C_{109}H_{170}N_{13}O_{61}H_2$: 2638.06783; found 1329.03337

CP8-deAc-Hexasaccharide (2-deAc)

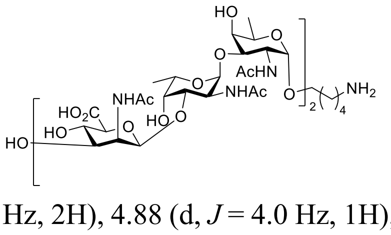

2 (1.76 mg, 0.00128 mmol) was dissolved in 1 M NaOH (0.5 mL) and stirred overnight at rt. The solution was diluted with H_2O and neutralized with Amberlite IR-120 H^+ resins, filtered and lyophilized. Purification by HW-40 with NH_4OAc gave **2-deAc** in 41% yield (0.68 mg, 0.000529 mmol). **^{1}H NMR (850 MHz, D_2O)** δ 4.91 (t, J = 3.6 Hz, 2H), 4.88 (d, J = 4.0 Hz, 1H), 4.80 (d, J = 1.4 Hz, 1H), 4.78 (d, J = 1.3 Hz, 1H), 4.38 (d, J = 3.7 Hz, 1H), 4.34 (dd, J = 4.4, 1.4 Hz, 1H), 4.19 – 4.16 (m, 2H), 4.12 – 4.07 (m, 3H), 4.08 – 4.00 (m, 3H), 3.98 (dt, J = 11.8, 6.6 Hz, 2H), 3.94 (dd, J = 12.5, 2.7 Hz, 2H), 3.83 (dd, J = 11.0, 3.1 Hz, 2H), 3.79 – 3.63 (m, 10H), 3.60 – 3.54 (m, 4H), 3.51 (t, J = 9.8 Hz, 1H), 3.36 (dt, J = 10.0, 6.2 Hz, 1H), 2.90 (t, J = 7.7 Hz, 2H), 1.95 – 1.93 (m, 14H), 1.89 – 1.86 (m, 7H), 1.58 (q, J = 7.7 Hz, 2H), 1.54 (q, J = 8.2, 7.4 Hz, 2H), 1.35 (dq, J = 16.2, 7.7 Hz, 2H), 1.18 – 1.08 (m, 20H). **HRMS**: $[M+H]^+$ calculated for $C_{53}H_{82}N_7O_{29}H$: 1286.56264; found 1286.56234

CP8-deAc-Nonasaccharide (3-deAc)

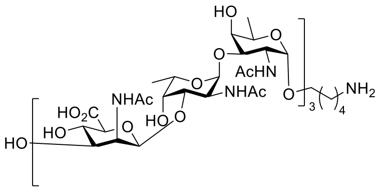

2 (1.4 mg, 0.000699 mmol) was dissolved in 1 M NaOH (0.5 mL) and stirred overnight at rt. The solution was diluted with H_2O and neutralized AcOH and lyophilized. Purification by HW-40 with NH_4OAc gave **2-deAc** in 46% yield (0.6 mg, 0.000319 mmol). **^{1}H NMR (850 MHz, D_2O)** δ 4.93 – 4.89 (m, 4H), 4.80 (d, J = 16.8 Hz, 3H), 4.39 (d, J = 4.2 Hz, 1H), 4.36 (d, J = 4.3 Hz, 1H), 4.19 (dd, J = 11.0, 3.8 Hz, 3H), 4.13 – 3.92 (m, 18H), 3.87 – 3.83 (m, 1H), 3.77 – 3.67 (m, 10H), 3.62 – 3.50 (m, 6H), 3.37 (p, J = 6.1 Hz, 1H), 2.91 (t, J = 7.9 Hz, 2H), 1.99 – 1.93 (m, 19H), 1.90 – 1.87 (m, 9H), 1.57 (dp, J = 45.1, 7.3 Hz, 6H), 1.35 (dt, J = 16.1, 8.1 Hz, 2H), 1.16 – 1.13 (m, 12H), 1.12 (d, J = 7.1 Hz, 7H). **HRMS**: $[M+H]^+$ calculated for $C_{77}H_{124}N_{10}O_{43}H$: 1877.79020; found 1877.79204

Preparation of S. aureus type 8 conjugates

Preparation of S. aureus type 8 conjugates (CRM$_{197}$ in PBS x1)

The CP8-OS were solubilized in 350 µL of a 9:1 DMSO:water solution with either 30 equiv. (for **1**) or 15 equiv. (for **2** and **3**) of linker (suberic acid bis(*N*-hydroxysuccinimide ester)) and stirred for 2 h at rt. The derivatized CP8-OS were purified by EtOAc precipitation. The solution was first incubated with 5 mL cold EtOAc and 250 µL NaCl (3 M, aq.) for 1 h at 4 °C. The EtOAc layer was discarded and the bottom phase was washed with cold EtOAc (3 mL) 10-15 times. The resulting solids were lyophilized overnight. The mass after linker installation was measured and a 90% recovery was predicted.

For conjugation, a 20 mg/mL CRM$_{197}$ solution in phosphate-buffered saline (PBS) was used with estimated 10, 20 and 30 eq of weighed derivatized CP8-OS. The reaction is incubated overnight at 4 °C.

Evaluation by SDS-PAGE:

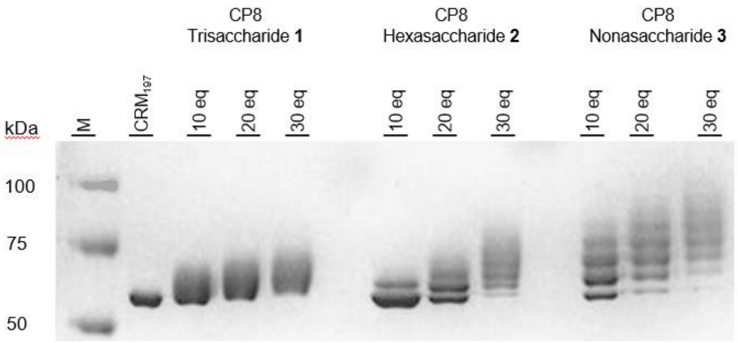

Figure S1: Evaluation of the CP8-conjugates performed in PBS

Preparation of S. aureus type 8 conjugates (CRM$_{197}$ in HEPES 25 mM)

A CRM$_{197}$ stock solution was buffer-exchanged to 25 mM HEPES pH 8.0 through Zeba™ Spin Desalting Column 7K MWCO. The derivatized **1**, **2** and **3** from earlier were used. 1.24 mg of 12-mer were derivatized following the same procedure as for the others.

To avoid weighing or not fully solubilizing the sugar in DMSO due to the remaining NaCl, the whole derivatized sample of **4** was used for conjugation, corresponding to approx. 35 eq. For the **1**, **2** and **3**, the conjugation was made at 10.8 mg/mL of CRM$_{197}$ with an estimated 30 eq. of sugar. However, due to the initial low loading, the rest of the derivatized sugar solubilized in DMSO was added, for an estimated 60 eq. of sugar (at approx. 8.8, 6.3 and 4.4 mg/mL of CRM$_{197}$ for the **1**, **2** and **3**, respectively).

The conjugates were filtered (ø 0.2 µm) under sterile conditions. The protein content was quantified in triplicates with the Qubit™ Protein Assay (Invitrogen).

Evaluation by SDS-PAGE:

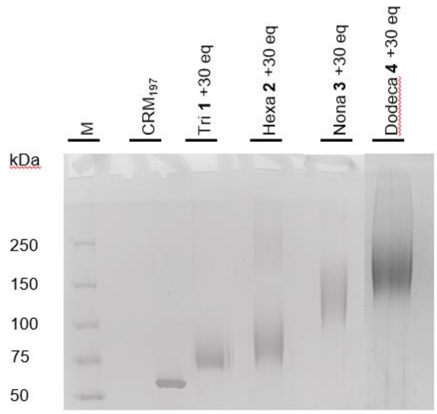

Figure S2: Evaluation of the CP8-conjugates performed in HEPES 25 mM

Table S4: Evaluation of the conjugates.

	CRM-1	CRM-2	CRM-3	CRM-4
**Average number of conjugated sugar chains (MALDI)**	11	8	13	14
**Protein quantification (BCA) [mg/mL]**	1.68	1.19	0.92	0.75
**Average protein [µM]**	19.0	10.3	9.2	12.8
**Average sugar [µM]**	316	163	206	187
**Average sugar [µg/mL]**	228	224	409	490
**Saccharide/protein w/w**	0.14	0.19	0.44	0.65

MALDI-TOF MS

For MALDI analysis (MALDI-TOF MS, AXIMA Performance, Shimadzu), 40 µL of conjugate samples (1 mg/mL), and CRM_{197} (0.5 mg/mL) were desalted in Amicon 0.5 mL MWCO 3K and exchanged to 1% TFA in MilliQ. For adding the sample to the matrix, a saturated solution of sinapic acid in 70% 1%TFA in MilliQ and 30% MeCN was used while a saturated solution of sinapic acid in absolute EtOH was used for priming the matrix.

CP8 – Trisaccharide 30 eq.

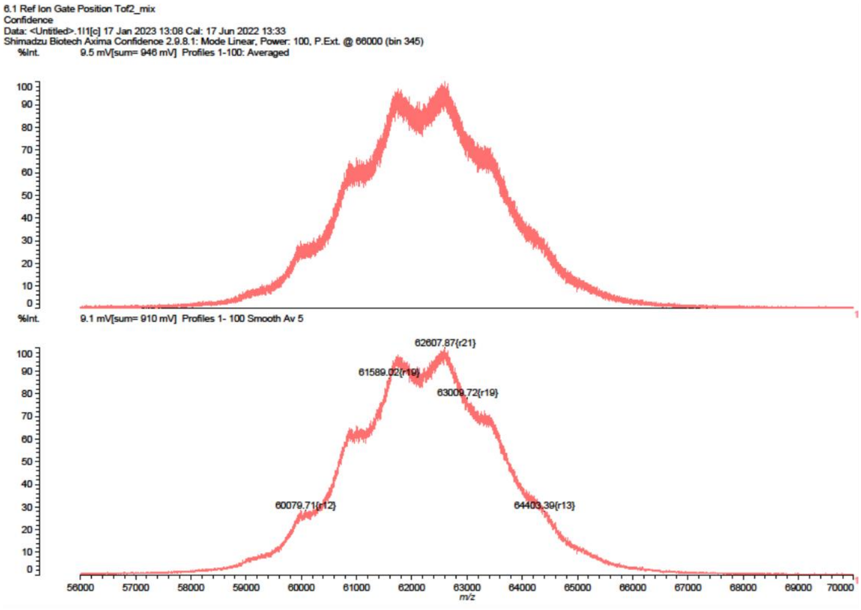

Figure S3: MALDI results of **1** with 30 equiv. in PBSx1

CP8 – Hexasaccharide 30 eq.

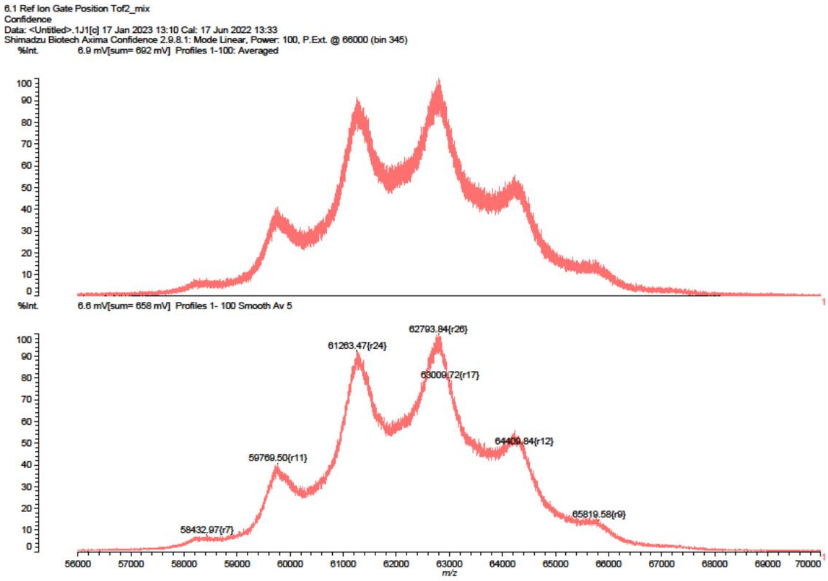

Figure S4:MALDI results of **2** with 30 equiv. in PBSx1

CP8 – Nonasaccharide 30 eq

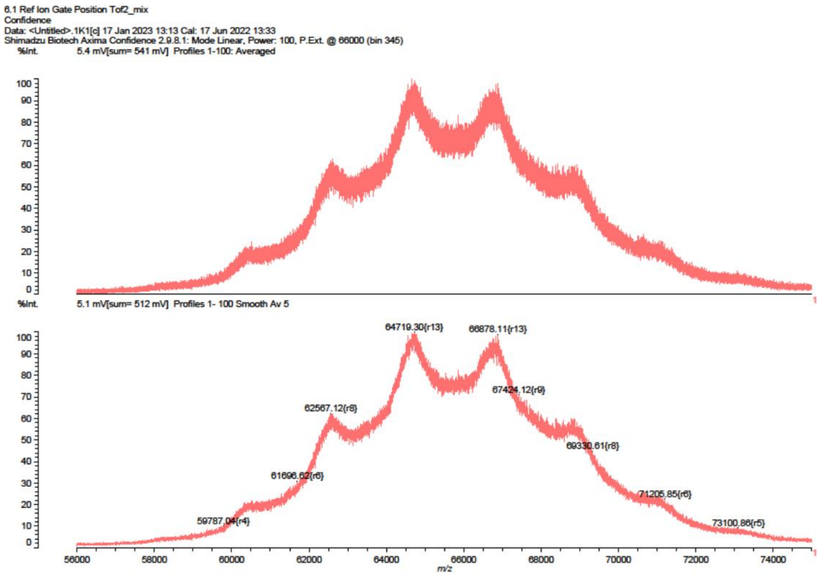

Figure S5: MALDI results of 3 with 30 equiv. in PBSx1

CP8 – Trisaccharide +30 eq. and HEPES 25 nM

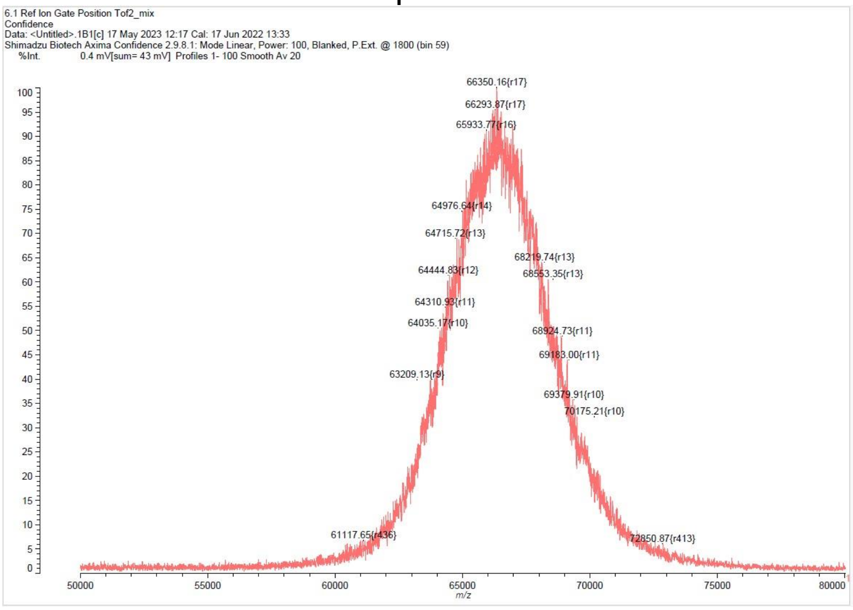

Figure S6: MALDI results of **1** with 30 equiv. in HEPES 25 nM

CP8 – Hexasaccharide +30 eq. and HEPES 25 nM

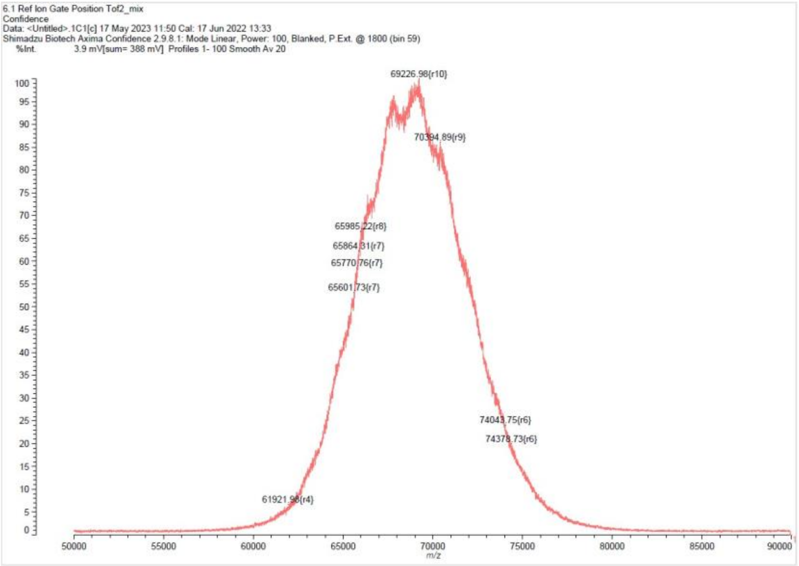

Figure S7: MALDI results of **2** with 30 equiv. in HEPES 25 nM

CP8 – Nonasaccharide +30 eq. and HEPES 25 nM

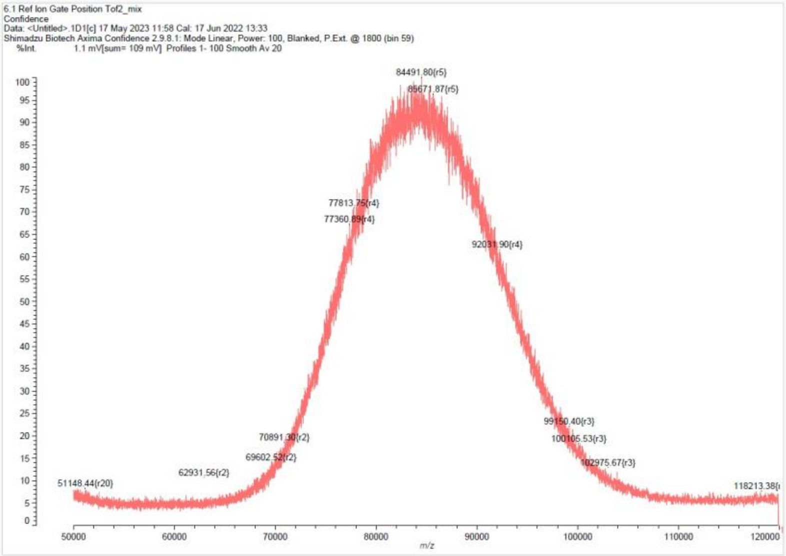

Figure S8: MALDI results of **3** with 30 equiv. in HEPES 25 nM

CP8 – Dodecasaccharide +30 eq. and HEPES 25 nM

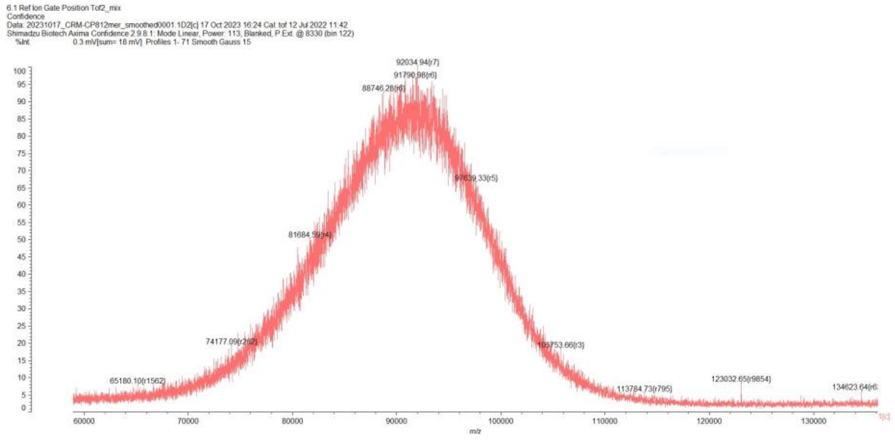

Figure S9: MALDI results of **4** with 30 equiv. in HEPES 25 nM

Protocol for Western Blot (using mAb and pAb)

SDS-PAGE were run the **1**-, **2**-, **3**- and **4** conjugates and a BSA-Pel-CRM_{197} conjugate (negative control), with a 7.5% acrylamide gel. The gel was transferred to a membrane for 30 min, which was blocked in 5% w/v milk in PBST (PBS supplemented with 0.1% Tween20) blocking solution for 1 h at rt. The membrane was then incubated for 1 h at rt with 1:1000 anti-CP8 mAb or 1:1000 anti-CP8 pAb (in blocking solution) followed by washing with PBST three times. Next, the membrane was incubated for 30 min at rt with 1:2000 IgGκ (m-IgGκ BP-HRP: sc-516102, Santa Cruz Biotechnology, in blocking solution) and again washed with PBST three times. The membrane was detected with Clarity Max Western ECL Substrate (Bio-Rad).

Western Blot performed with pAb and ***1****,* ***2*** *and* ***3****:*

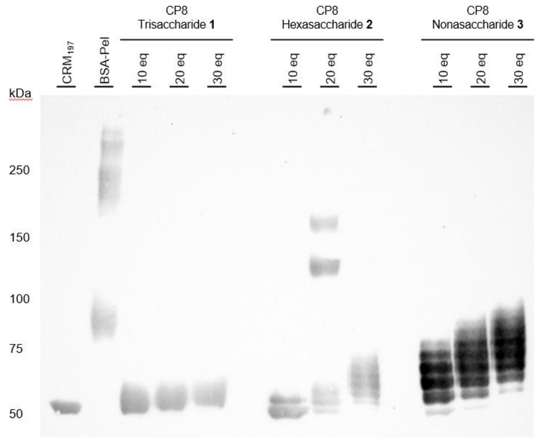

Figure S10: Western Blot with the CP8-Crm_{197} conjugates in PBS with pAb

Protocol for competitive ELISA with mAb

A 96-well plate was coated with 50 µL CP8 10 µg/mL in PBS, incubated at 4 °C overnight then washed with PBST (0.1% Tween-20 in PBS pH 7.4). The plate was blocked with 3% milk in PBST 0.1%) at 37 °C for 1.5 h.

A 1.5-fold serial dilution of the CP8-OS competitors was prepared, followed by a pre-incubation with anti-CP8 mAb (final 0.63 µg/mL) at 37 °C for 30 min, after which 50 µL of each competitor sample were pipetted into their corresponding wells. The final competitor concentrations can be found in Table S5.

The plate was incubated at 37 °C for 1 h, then washed with PBST. 50 µL of anti-mouse IgG (secondary antibody, m-IgGκ BP-HRP: sc-516102, Santa Cruz Biotechnology) diluted 1:1000 in PBST 0.3% milk were pipetted into each well. The plate was incubated at 37 °C for 1.5 h, washed with PBST, then developed with 50 µL of coloring solution (Invitrogen, 1X TMB Substrate Solution) for 30 min at rt. The reaction was stopped with 25 µL of 0.16 M H_2SO_4 after which it was read at 450 nm.

Table S5: The final competitor concentrations for competitive ELISA with mAb.

	Competitor concentrations (µg/mL)											
**1**	1000	667	444	296	198	132	88	59	39	26	17	0
**2**	1000	667	444	296	198	132	88	59	39	26	17	0
**3**	700	467	311	207	138	92	61	41	27	18	12	0
**4**	300	200	133	89	59	40	26	18	12	8	5	0
**CP8 PS**	7	4.67	3.11	2.07	1.38	0.92	0.61	0.41	0.27	0.18	0.12	0

ELISA titers (synthetic fragments and mAb)

The calculation of IC50 values were performed with GraphPad Prism software using the variable slope model (GraphPad Prism Inc.). The means of each group were compared with a one-way ANOVA analysis; "**" denotes the significant result within p < 0.01, "ns" means not significant.

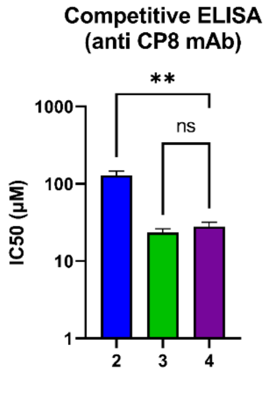

Figure S11: ELISA titers with synthetic fragments and mAb

Protocol for competitive ELISA with pAb

The competitive ELISA with a polyclonal serum against CP8-DT conjugate was run in the same fashion as for the mAb one (see above).

A 2-fold (**2**, **3**, **4**) or 3-fold (**1** and **3**-deAc) serial dilution of the CP8-OS competitors was prepared, followed by a pre-incubation with anti-CP8 pAb (final dilution 1:500) at 37 °C for 30 min, after which 50 µL of each competitor sample were pipetted into their corresponding wells. The final competitor concentrations can be found in Table S6.

Table S6: The final competitor concentrations for competitive ELISA with pAb.

	Competitor concentrations (µg/mL)											
**2**	1000	500	250	125	63	31	16	8	4	2	1	0
**3**	700	350	175	88	44	22	11	5	3	1	1	0
**4**	700	350	175	88	44	22	11	5	3	1	1	0
**1 / 3-deAc**	1000	333	111	37	12	-	-	-	-	-	-	-

ELISA titers (synthetic fragments and pAb)

The calculation of IC50 values were performed with GraphPad Prism software using the variable slope model (GraphPad Prism Inc.). The means of each group were compared with a one-way ANOVA analysis; "***" denotes the significant result within $p < 0.001$, "ns" means not significant.

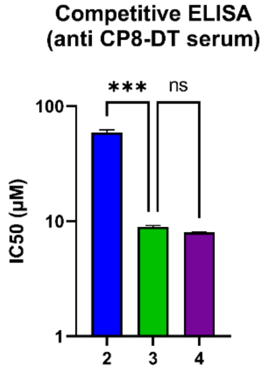

Figure S12: ELISA titers with synthetic fragments and pAb

Structural conformation

Structure and conformational studies

NMR methods. NMR experiments were performed in a Bruker Avance III 800 MHz spectrometer equipped with a TCI cryoprobe. Samples were dissolved in D_2O at 1.0 mM concentration. Experiments were acquired at the temperature of 298 K.

^{1}H and ^{13}C NMR resonances of the molecules **1**, **2**, **2-deAc**, and **3** were assigned through standard 2D-TOCSY, 2D-ROESY, 2D-NOESY, 2D ^{1}H-^{13}C-HSQC. 2D-TOCSY experiments were acquired with 30 ms mixing time, 1.0 s of relaxation delay, 4 scans, and 4096x256 (F2xF1) points with a spectral width of 6556.0 Hz. 2D-ROESY experiment was acquired with mixing time of 200 ms, 1.0 s of relaxation delay, 48 scans, and 4096x256 (F2xF1) points with a spectral width of 6880.7 Hz. 2D-NOESY experiment was acquired with mixing time of 200 ms, 1.5 s of relaxation delay, 32 scans, and 4096x256 (F2xF1) points with a spectral width of 6242.2 Hz. 2D ^{1}H,^{13}C-HSQC experiments were acquired with

1.0 s of relaxation delay, 48 scans, and 4096x220 (F2xF1) points with a spectral width of 6250.0 Hz (F2) and 24144.6 Hz (F1). The data were processed with Topspin 4.2 (Bruker Biospin) using a 90° shifted qsine window function to a total of 16K × 2K data points (F2 × F1), followed by automated baseline- and phase correction.

NMR spectra and NMR signal assignment.

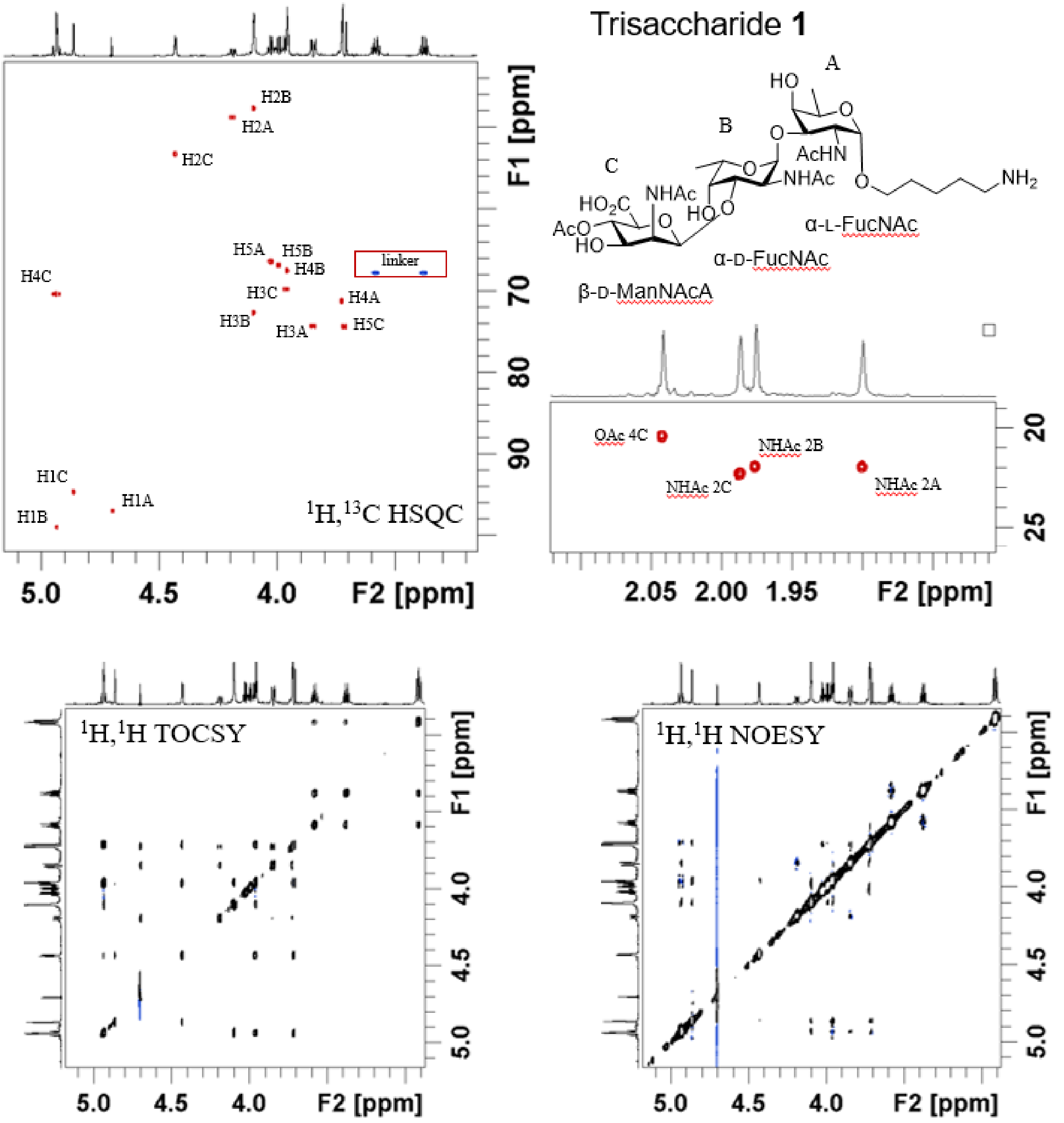

Figure S13: Trisaccharide **1** NMR characterization. The ^{1}H and ^{13}C NMR signals of the trisaccharide **1** were assigned through the combined use of herein reported 2D NMR spectra.

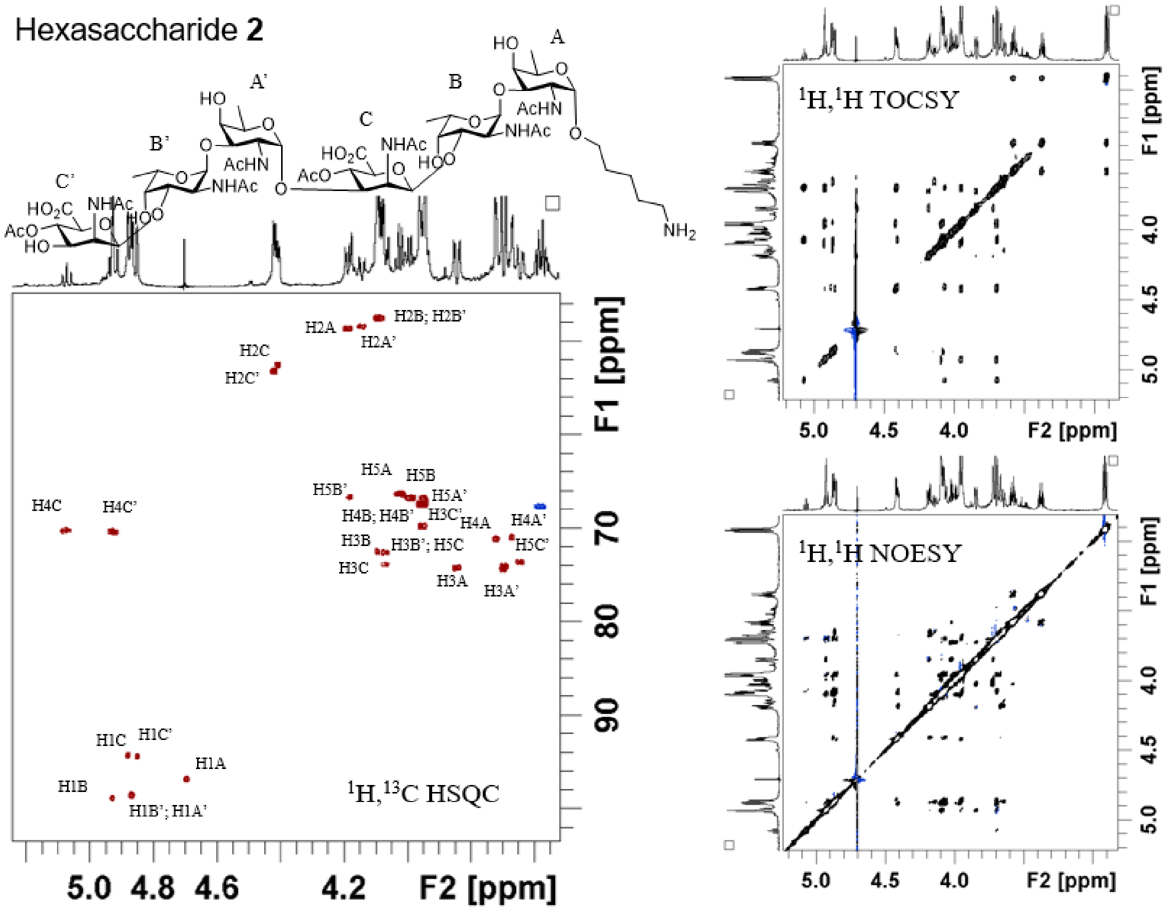

Figure S14: Hexasaccharide **2** NMR characterization. The ^{1}H and ^{13}C NMR signals of the hexasaccharide **2** were assigned through the combined use of herein reported 2D NMR spectra.

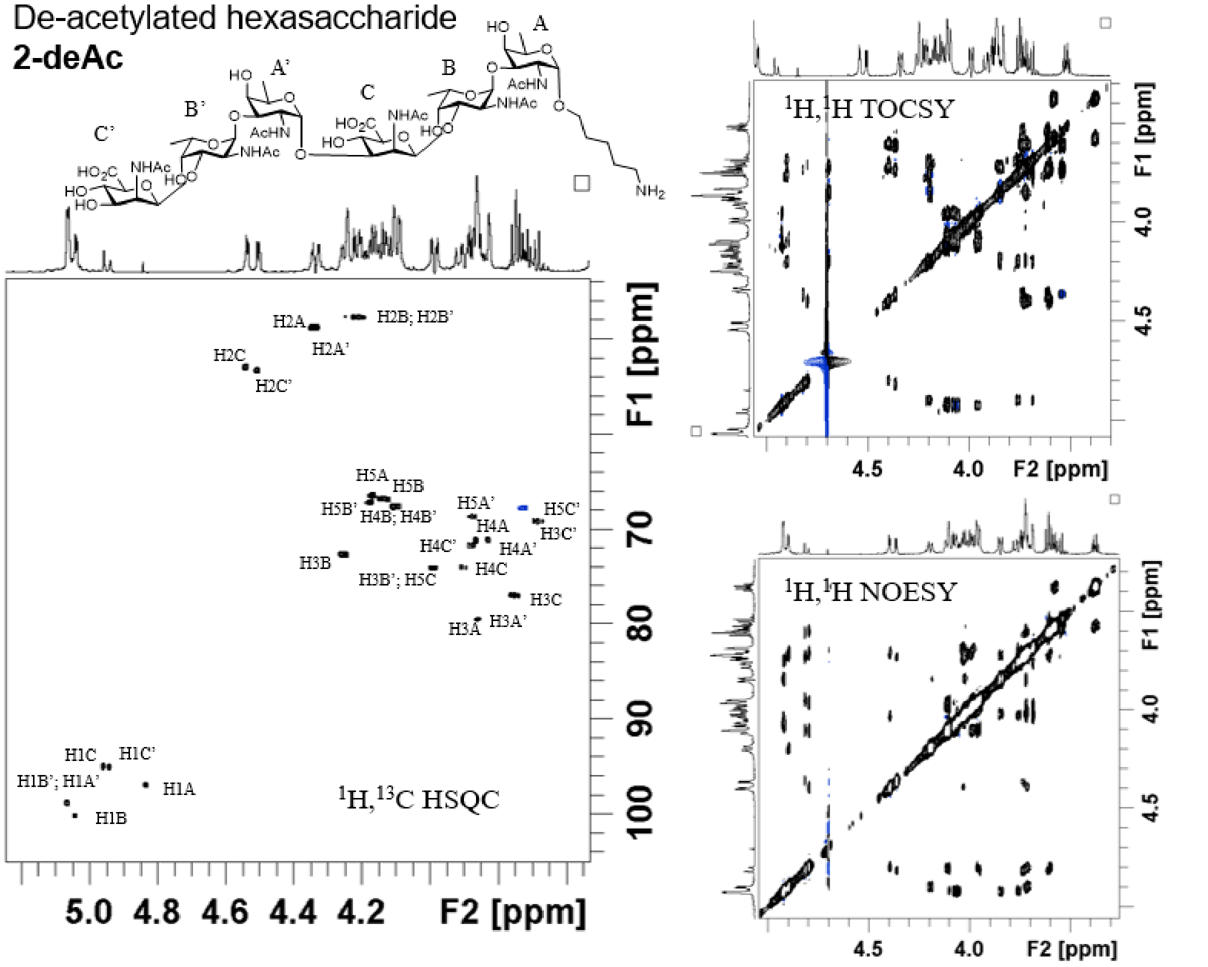

Figure S15: De-acetylated hexasaccharide **2-deAc** NMR characterization. The ^{1}H and ^{13}C NMR signals of the hexasaccharide **2-deAc** were assigned through the combined use of herein reported 2D NMR spectra.

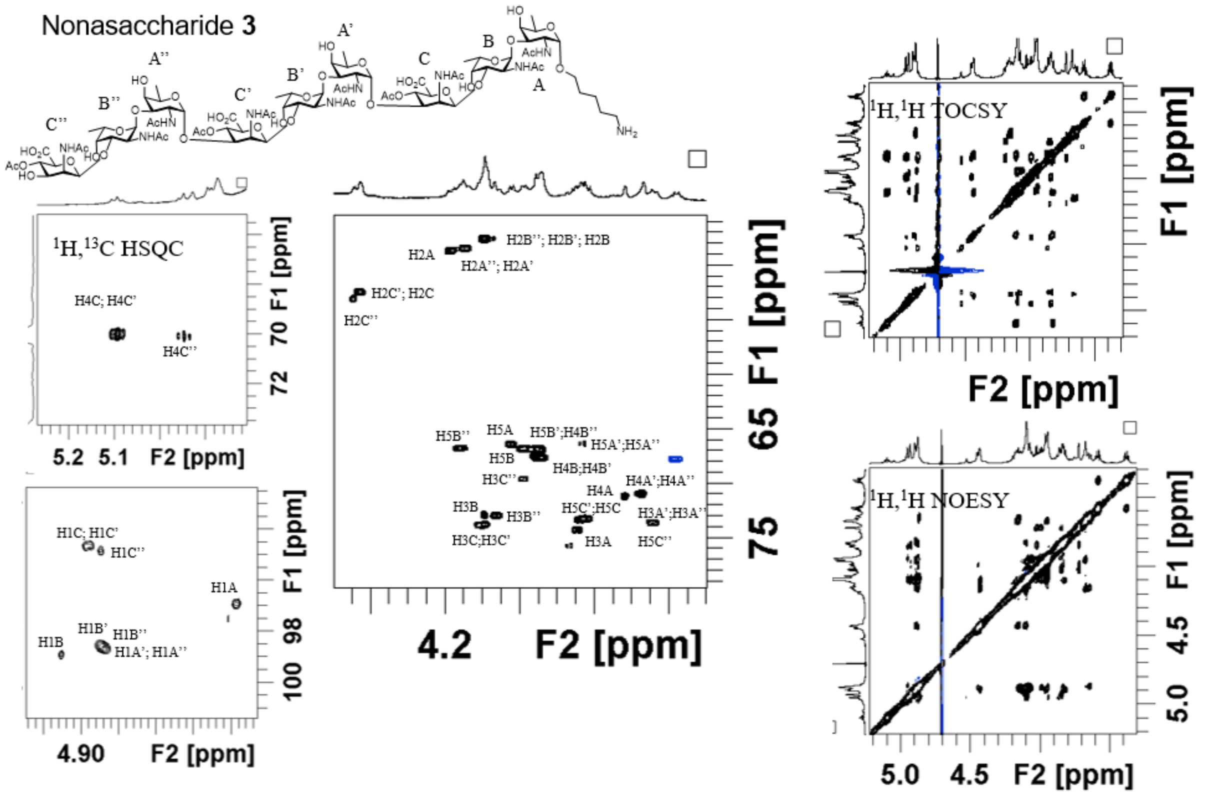

Figure S16: Nonasaccharide **3** NMR characterization. The ^{1}H and ^{13}C NMR signals of the nonasaccharide **3** were assigned through the combined use of herein reported 2D NMR spectra.

Molecular Mechanics Calculations. The geometry optimization was performed by using the Jaguar/Schroedinger package (version 13.5) and the AMBER* force field, with the GB/SA continuum solvent model for water. The glycosidic torsion angles were defined as ϕ (H1'-C1'-Ox-Cx) and ψ (C1'-Ox-Cx-Hx). Extended nonbonded cut-off distances (van der Waals cut-off of 8.0 Å and electrostatic cut-off of 20.0 Å) were used. The conformers for the tri- and nona-saccharide molecules **1** and **3** were generated employing geometric restrictions to respect the *exo*-anomeric effect. The possible staggered rotamers around ψ were selected and minimized. The coordinates of the obtained local minima were employed to measure the key inter-proton distances that were then compared to those obtained experimentally by the ROESY and NOESY NMR experiments through integration of the observed NOEs cross peaks using the ISPA approximation. The resulting conformations and NOE distances analysis are reported in Table S7 and Figure S17, for the trisaccharide **1**, and in Table S8 and Figure S18 for the nonasachharide **3**.

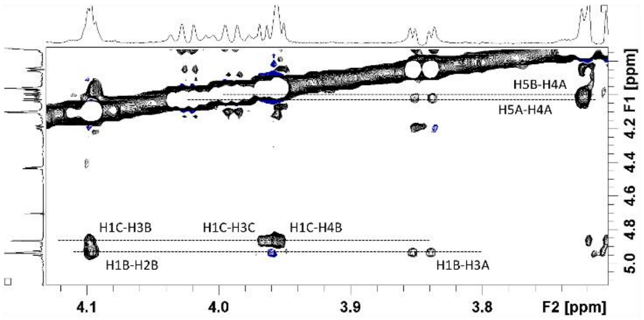

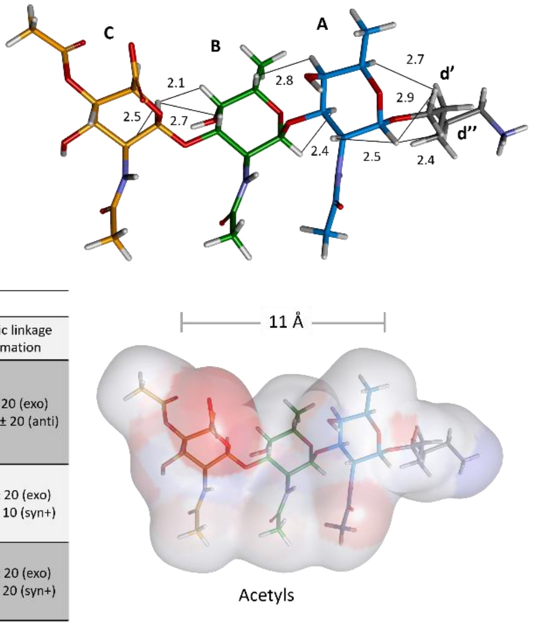

Compund 1

Atom Pair	Calculated Distance for the selected conformer (Å)	Observed NOE Intensity	Experimental Distance (Å) ±5%	Glycosidic linkage Conformation
H5A-H4A	2.5 (ref)	Strong	2.5	Φ = -45 ± 20 (exo)<br>Ψ = +180 ± 20 (anti)
H1A-Hd'	2.9	Weak	>3.5	
H1A-Hd''	2.4	Medium	2.5	
H5A-Hd'	2.7	Weak	3.1	
H1B-H2B	2.5 (ref)	Strong	2.5	Φ = +44 ± 20 (exo)<br>Ψ = +20 ± 10 (syn+)
H1B-H3A	2.4	Strong	2.5	
H5B-H4A	2.8	Strong	2.7	
H1C-H2C	2.5 (ref)	Strong	2.5	Φ = +45 ± 20 (exo)<br>Ψ = +48 ± 20 (syn+)
H1C-H3B	2.7	Strong	2.4	
H1C-H4B	2.1	Strong	2.3	

Figure S17 and **Table S7**: Expansion of NOESY spectrum of trimer **1**. Key cross-peaks defining the conformation around the glycosidic linkages are indicated. Table **S7** reporting the analysis of the experimental NMR-NOEs data and the derived conformation. Main conformation of **1** as determined by NOEs based calculated interatomic distances. Stick and surface representation of 3D structure of **1** in solution. The oligosaccharide length and orientation of the hydrophobic acetyl groups is represented.

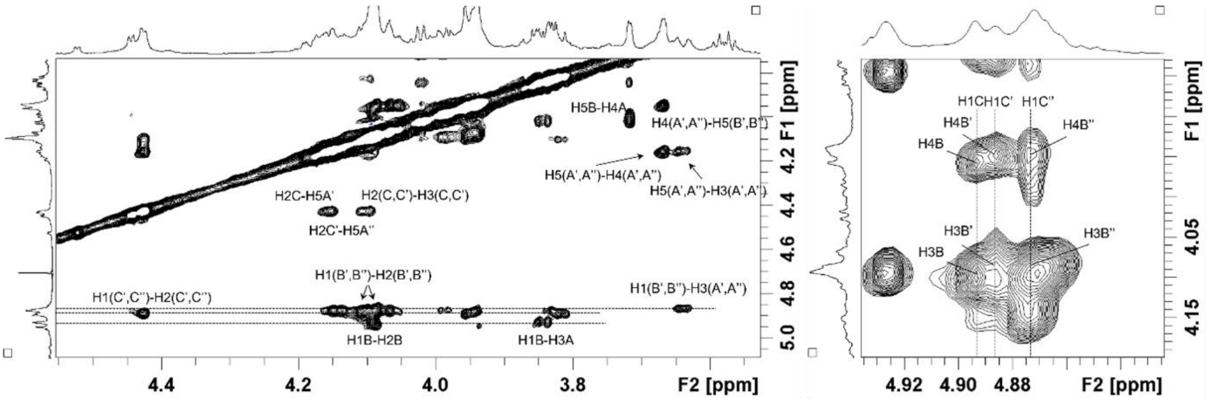

Compund 2

Atom Pair	Calculated Distance for the selected conformer (Å)	Observed NOE Intensity	Glycosidic linkage Conformation
H1A-Hd′	2.9	Weak	Φ = -45 ± 20 (exo) Ψ = +178 ± 20 (anti)
H1A-Hd′′	2.4	Strong	
H5A-Hd′	2.7	Medium	
H1B-H3A	2.4	Strong	Φ = +44 ± 20 (exo) Ψ = +20 ± 10 (syn+)
H5B-H4A	2.8	Medium/Strong	
H1C-H4B	2.1	Strong	Φ = +44 ± 20 (exo) Ψ = +50 ± 20 (syn+)
H1C-H3B	2.7	Medium/Strong	
H1A′-(OAc)C	2.9	Weak	Φ = -45 ± 20 (exo) Ψ = -26 ± 10 (syn-)
H5A′-H2C	2.4	Strong	
H1A′-H2C	4.3	Absent	
H1B′-H3A′	2.3	Strong	Φ = +44 ± 20 (exo) Ψ = +20 ± 10 (syn+)
H1B′-H4A′	3.0	Absent	
H5B′-H4A′	2.8	Weak	

Compund 2

Atom Pair	Calculated Distance for the selected conformer (Å)	Observed NOE Intensity	Glycosidic linkage Conformation
H1C′-H4B′	2.6	Strong	Φ = +45 ± 20 (exo) Ψ = +51± 20 (syn+)
H1C′-H3B′	2.6	Medium/Strong	
H1A′′-H2C′	4.2	Absent	Φ = -46 ± 20 (exo) Ψ = -27 ± 10 (syn-)
H1A′′-H4C′	3.8	Absent	
H5A′′-H2C′	2.3	Strong	
H1B′′-H3A′′	2.1	Strong	Φ = +44 ± 20 (exo) Ψ = +20 ± 10 (syn+)
H1B′′-H4A′′	3.0	Absent	
H1B′′-H2A′′	4.6	Absent	
H5B′′-H4A′′	2.4	Strong	
H1C′′-H4B′′	2.2	Strong	Φ = +46 ± 20 (exo) Ψ = +54 ± 20 (syn+)
H1C′′-H3B′′	2.6	Medium/Strong	

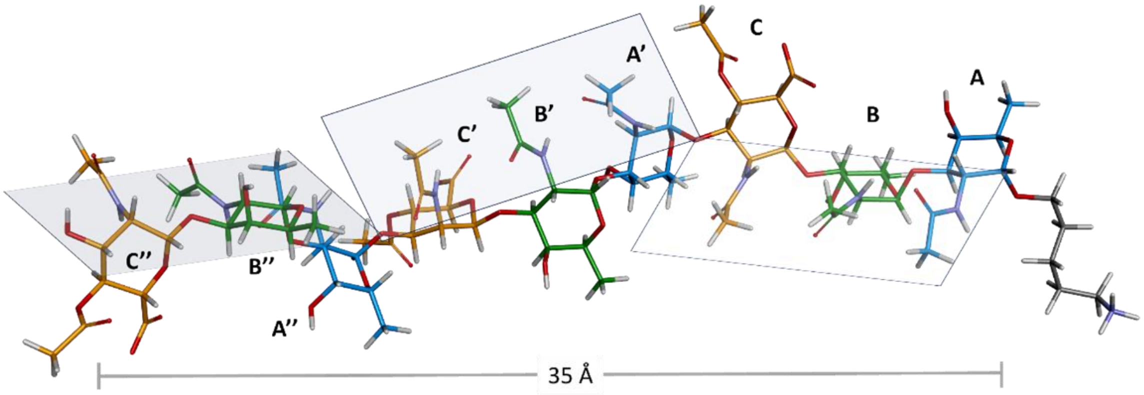

Figure S18 and **Table S8:** Expansion of NOESY spectrum of nonasaccharide **3**. Key cross-peaks defining the conformation around the glycosidic linkages are indicated, together with some intra-residue NOE as reference. Table **S8** reporting the qualitative analysis of the experimental NMR-NOEs data and the derived conformation. The major conformation of **3** as determined by the NOE analysis, assisted by MM calculations. The relative orientation of the imaginary planes containing the sugar's rings of each RU are represented.

Ligands-Antibody interaction studies

^{1}H-STD NMR experiments & methods. For the acquisition of the ^{1}H-STD-NMR experiments the mAb-CP8 antibody was buffer exchanged to deuterated PBS 1X pD 7.8 using centrifuge filters (Sartorius Vivaspin 6 50000 MWCO) up to an antibody concentrated of 2 µM. 100 equivalents of ligands (**1-3**) were added, which resulted into a solution of 2 µM of mAb and 200 µM of the ligand.

The STD experiments were recorded using Bruker AVANCE II 800 MHz NMR spectrometer equipped with cryo-probe (Bruker Inc.; Billerica, MA, US) at different temperatures that ranged between 288 and 310 K. The used ^{1}H-STD pulse sequence includes T2 filter, for protein NMR signal suppression, and

excitation sculpting, for residual water NMR signal suppression. The STD NMR spectra were acquired with 2880 scans and 5 s of relaxation delay. Different conditions were screened for STD experiments. All the STD experiments were performed at both on-resonances, at the aliphatic (0.8 ppm) and aromatic (7.0 ppm) regions. The resulting STD spectra provided similar results (Figure S19). The on- and off-resonance spectra were registered in the interleaved mode with the same number of scans. The on-resonance protein saturation was obtained using a Gaussian shape pulse of 50 ms with a total saturation time of 2 s at a frequency of δ 0.8 ppm (aliphatic region). The off-resonance frequency was always set at δ 100 ppm.

The analysis was carried out using the ^{1}H NMR signals of the STD spectrum and from their comparison with the off-resonance spectrum, the STD-AF (Average Factor) was obtained. The strongest STD intensity was used as reference (100% of STD effect). On this basis, the relative STD intensities for the other protons were estimated from the comparison of the corresponding integrals. These relative STD intensities (STD%) were used to map the ligand-binding epitope.

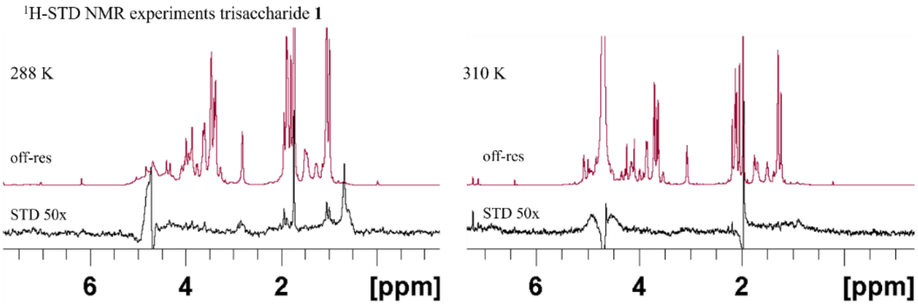

Figure S19: ^{1}H-STD NMR experiments of the trisaccharide **1** in presence on the mAb CP8.

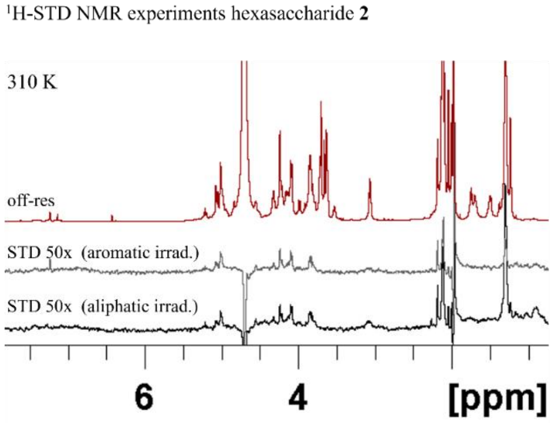

Figure S20: ^{1}H-STD NMR experiments of the hexasaccharide **2** in presence on the mAb CP8.

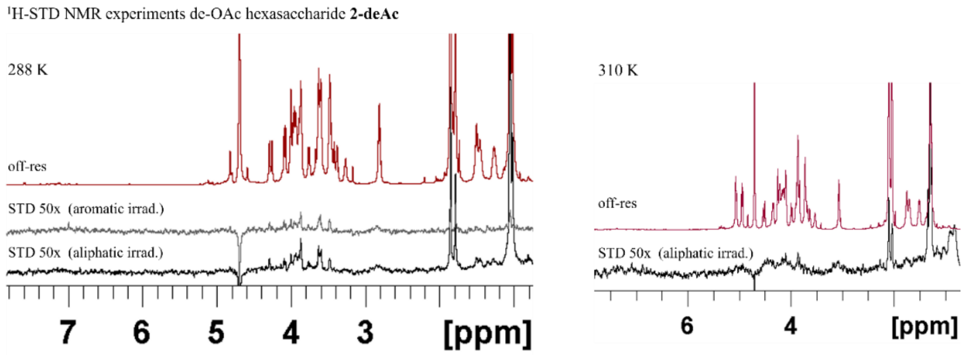

Figure S21: ^{1}H-STD NMR experiments of the de O-acetylated hexasaccharide **2-deAc** in presence on the mAb CP8.

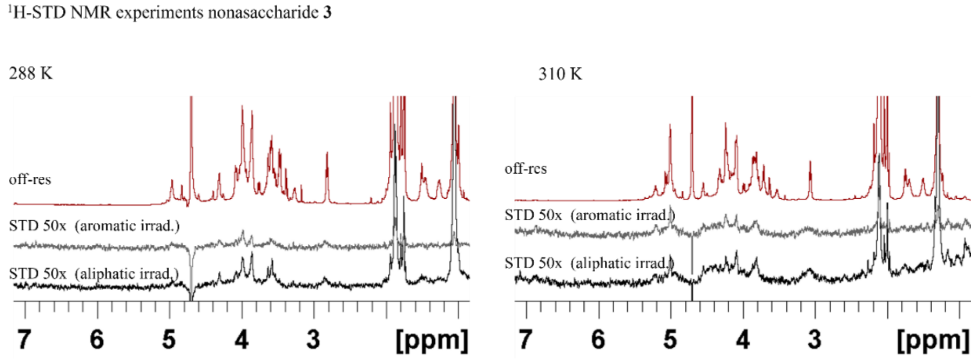

Figure 22: 1H-STD NMR experiments of the nonasaccharide **3** in presence on the mAb CP8.

Characterization of CP8-conjugated used for *in vivo* studies

Table S9: Characterization of the CP8-conjugated used for the *in vivo* studies.

Sample	Saccharide (µg/ml)	Protein (µg/ml)	Sacch/Prot (w/w)	LAL test (EU/ug)	Buffer
**CP8 PS 100% OAc-CRM Lot FC09Ago21**	121.5	145.7	0.8	0.40	PBS 1x pH 7.2
**CRM197-CP8 TRI (lot KE230525-TRI)**	228.1	1680.0	0.14	0.29	PBS 1x pH 7.2
**CRM197-CP8 HEXA (lot KE230525-HEXA)**	223.7	1191.8	0.19	<0.02	PBS 1x pH 7.2
**CRM197-CP8 NONA (lot KE230525-NONA)**	409.2	924.6	0.44	0.02	PBS 1x pH 7.2
**CRM197-CP8 12-MER (lot KE230623)**	491.1	749.4	0.65	0.07	PBS 1x pH 7.2

Immunizations

Animal studies were ethically reviewed by the local AWB and carried out at a GSK facility (or at the GSK Animal Facility in Siena) in accordance with national/European legislation, and the GSK Policies on the Care, Welfare and Treatment of Animals. The welfare of the animals was maintained in accordance with the general principles of the Association for Assessment and Accreditation of Laboratory Animal Care.

Five groups of 10 CD1 mice (5 week old) were immunized on day 1, 22 and 36 with 1.0 µg (saccharide titer) of conjugated carbohydrate antigen formulated with aluminum hydroxide as adjuvant. Sera was collected at day 0 (before first injection), 35 (32 days after first immunization) and 50 (14 days after second injection).

ELISA protocol for *in vivo* studies

IgG titers in collected sera were estimated by ELISA. 96-well microtiter plates were coated with 0.1 µg of CP8 polysaccharide. The plates were incubated overnight at 2-8°C, then washed three times with PBST (0.05% Tween-20 in PBS pH 7.4). The well were saturated with 250 µL/well of blocking buffer (2% Bovine Serum Albumin in PBST) for 90 min at 37°C. Two-fold serial dilutions of sera in blocking buffer were added to each well. The plates were then incubated at 37°C for 1h, washed with PBST, and then incubated for 90 min at 37°C with anti-mouse (whole molecule) IgG-alkaline phosphatase (Sigma-Aldrich) diluted 1:2000 in blocking buffer. The plates were washed with PBST and developed with a 4 mg/mL solution of p-Nitrophenyl Phosphate (pNPP) in 1 M diethanolamine (DEA) pH 9.8, at rt for 30 min. The absorbance was measured using a SPECTRAmax plate reader 405 nm. IgG titers were calculated by the reciprocal serum dilution giving an Optical Density (OD) of 1.

ELISA titers from the in vivo studies

The calculation of IC50 values were performed with GraphPad Prism software using Kruskal-Wallis with Dunn's multiple comparisons; "***" denotes the significant result within $p < 0.001$, "ns" means not significant.

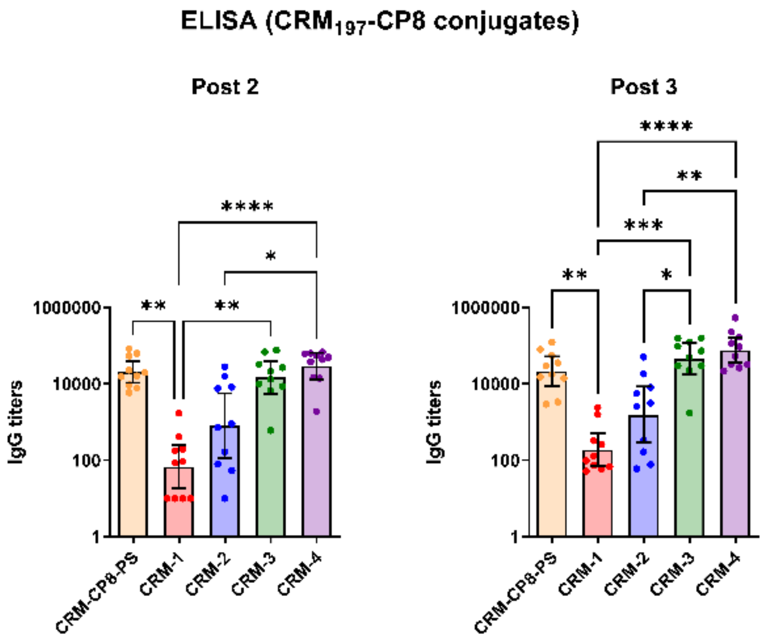

References

(1) Cheng, J. M. H.; Dangerfield, E. M.; Timmer, M. S. M.; Stocker, B. L. A Divergent Approach to the Synthesis of IGb3 Sugar and Lipid Analogues via a Lactosyl 2-Azido-Sphingosine Intermediate. *Org. Biomol. Chem.* **2014**, *12* (17), 2729–2736. https://doi.org/10.1039/c4ob00241e.

(2) Fomitskaya, P. A.; Argunov, D. A.; Tsvetkov, Y. E.; Lalov, A. V.; Ustyuzhanina, N. E.; Nifantiev, N. E. Further Investigation of the 2-Azido-Phenylselenylation of Glycals. *European J. Org. Chem.* **2021**, *2021* (44), 5897–5904. https://doi.org/10.1002/ejoc.202101167.

(3) David, S.; Hanessian, S. Regioselective Manipulation of Hydroxyl Groups via Organotin Derivatives. *Tetrahedron* **1985**, *41* (4), 643–663. https://doi.org/10.1016/S0040-4020(01)96443-9.

(4) Yu, B.; Tao, H. Glycosyl Trifluoroacetimidates. Part 1: Preparation and Application as New Glycosyl Donors. *Tetrahedron Lett.* **2001**, *42* (12), 2405–2407. https://doi.org/10.1016/S0040-4039(01)00157-5.

(5) Yan, R. B.; Yang, F.; Wu, Y.; Zhang, L. H.; Ye, X. S. An Efficient and Improved Procedure for Preparation of Triflyl Azide and Application in Catalytic Diazotransfer Reaction. *Tetrahedron Lett.* **2005**, *46* (52), 8993–8995. https://doi.org/10.1016/j.tetlet.2005.10.103.

(6) Hagen, B.; Ali, S.; Overkleeft, H. S.; van der Marel, G. A.; Codée, J. D. C. Mapping the Reactivity and Selectivity of 2-Azidofucosyl Donors for the Assembly of N-Acetylfucosamine-Containing Bacterial Oligosaccharides. *J. Org. Chem.* **2017**, *82* (2), 848–868. https://doi.org/10.1021/acs.joc.6b02593.

(7) Litjens, R. E. J. N.; Leeuwenburgh, M. A.; van der Marel, G. A.; Van Boom, J. H. A Novel Approach towards the Stereoselective Synthesis of 2-Azido-2-Deoxy-β-D-Mannosides. *Tetrahedron Lett.* **2001**, *42* (49), 8693–8696. https://doi.org/10.1016/S0040-4039(01)01880-9.

(8) van den Bos, L. J.; Codée, J. D. C.; Toorn, J. C. Van Der; Boltje, T. J.; Boom, J. H. Van; Overkleeft, H. S.; van der Marel, G. A. Thioglycuronides: Synthesis and Oligosaccharides in the Assembly of Acidic Oligosaccharides. *Org. Lett.* **2004**, *6* (13), 2165–2168. https://doi.org/10.1021/ol049380+.

(9) De Mico, A.; Margarita, R.; Parlanti, L.; Vescovi, A.; Piancatelli, G. A Versatile and Highly Selective Hypervalent Iodine (III)/ 2,2,6,6-Tetraniethyl-1-Piperidinyloxyl-Mediated Oxidation of Alcohols to Carbonyl Compounds. *J. Org. Chem.* **1997**, *62* (20), 6974–6977. https://doi.org/10.1021/jo971046m.

(10) Njeri, D. K.; Valenzuela, E. A.; Ragains, J. R. Leveraging Trifluoromethylated Benzyl Groups toward the Highly 1,2- Cis -Selective Glucosylation of Reactive Alcohols. *Org. Biomol. Chem.* **2021**, *23*, 8214–8218. https://doi.org/10.1021/acs.orglett.1c02947.

(11) Schumann, B.; Parameswarappa, S. G.; Lisboa, M. P.; Kottari, N.; Guidetti, F.; Pereira, C. L.; Seeberger, P. H. Nucleophile-Directed Stereocontrol Over Glycosylations Using Geminal-Difluorinated Nucleophiles. *Angew. Chemie Int. Ed.* **2016**, *55* (46), 14431–14434. https://doi.org/10.1002/anie.201606774.

(12) Gagarinov, I. A.; Srivastava, A. D.; Boons, G.-J.; Wang, Z. *A Multigram Synthesis of Phenyl 2-Azido-3-O-Benzyl-4,6-O-Benzylidene-2-Deoxy-1-Thio-α-d-Mannopyranoside*; 2017.

(13) Noti, C.; Paz, J. L. De; Polito, L.; Seeberger, P. H. Preparation and Use of Microarrays Containing Synthetic Heparin Oligosaccharides for the Rapid Analysis of Heparin – Protein Interactions. *Chem. - A Eur. J.* **2006**, *12*, 8664–8686. https://doi.org/10.1002/chem.200601103.

Spectra

1,2,3,4-tetra-*O*-acetyl-D-fucopyranose (S1)

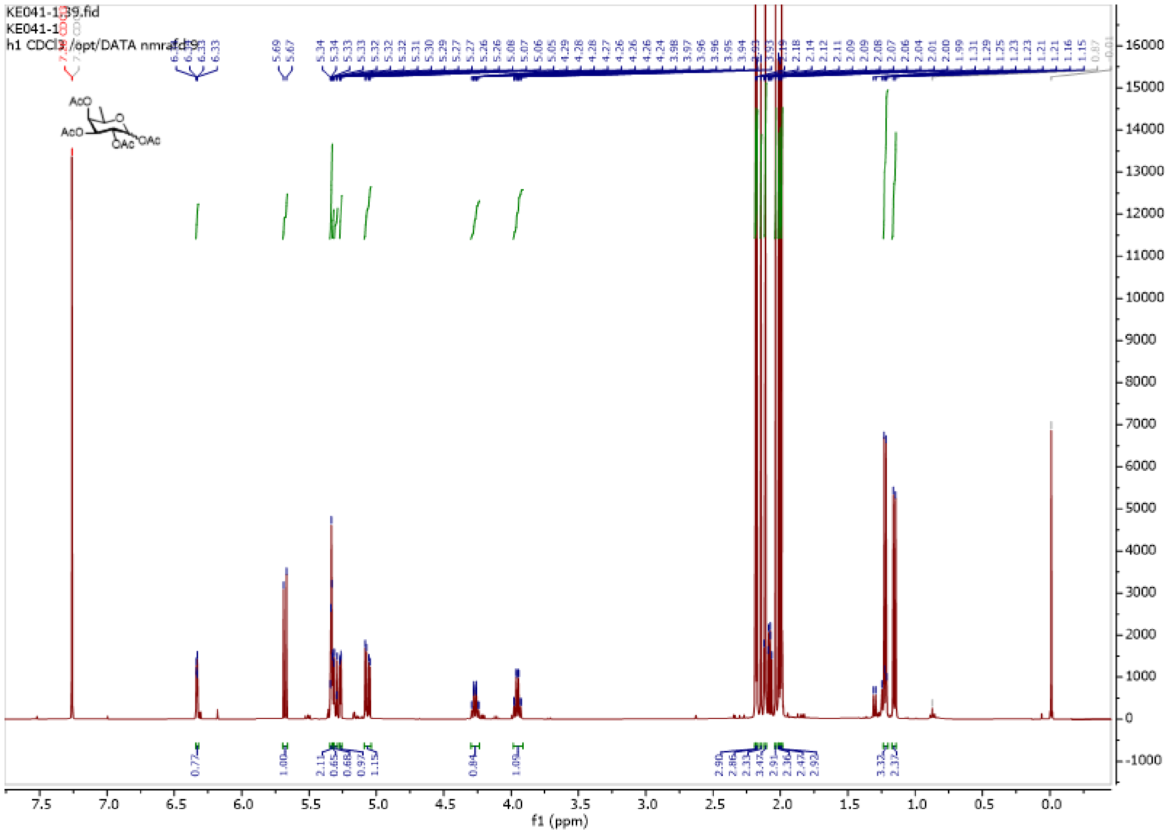

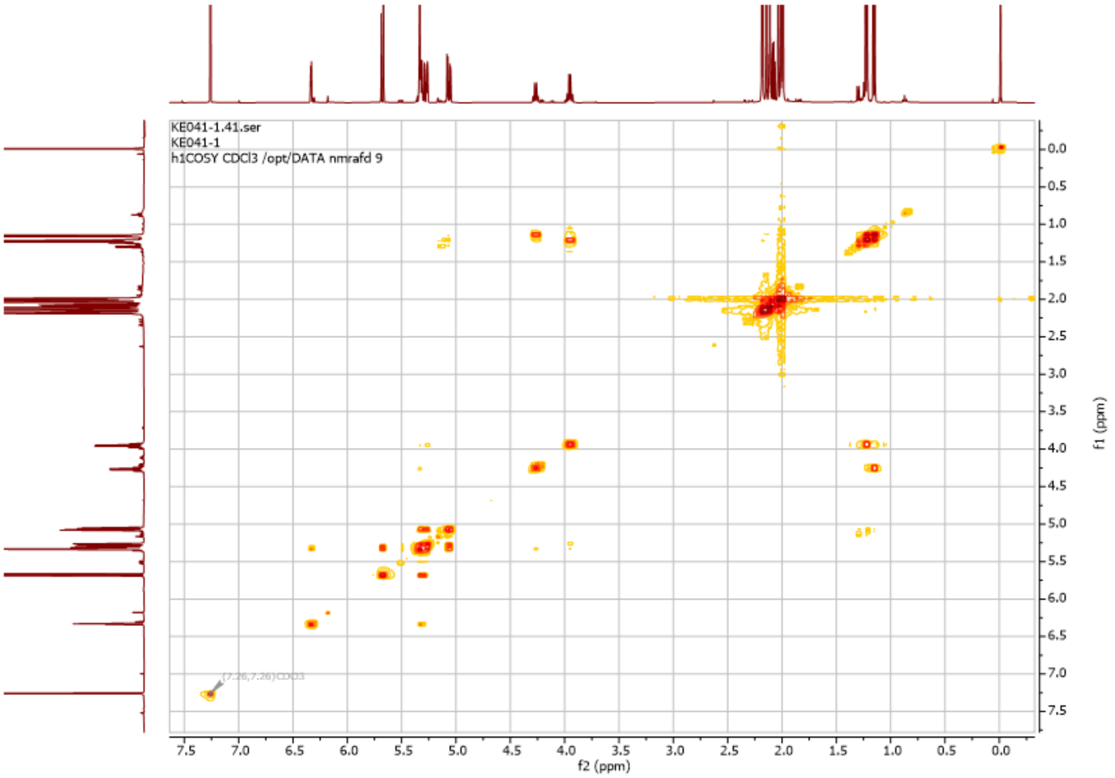

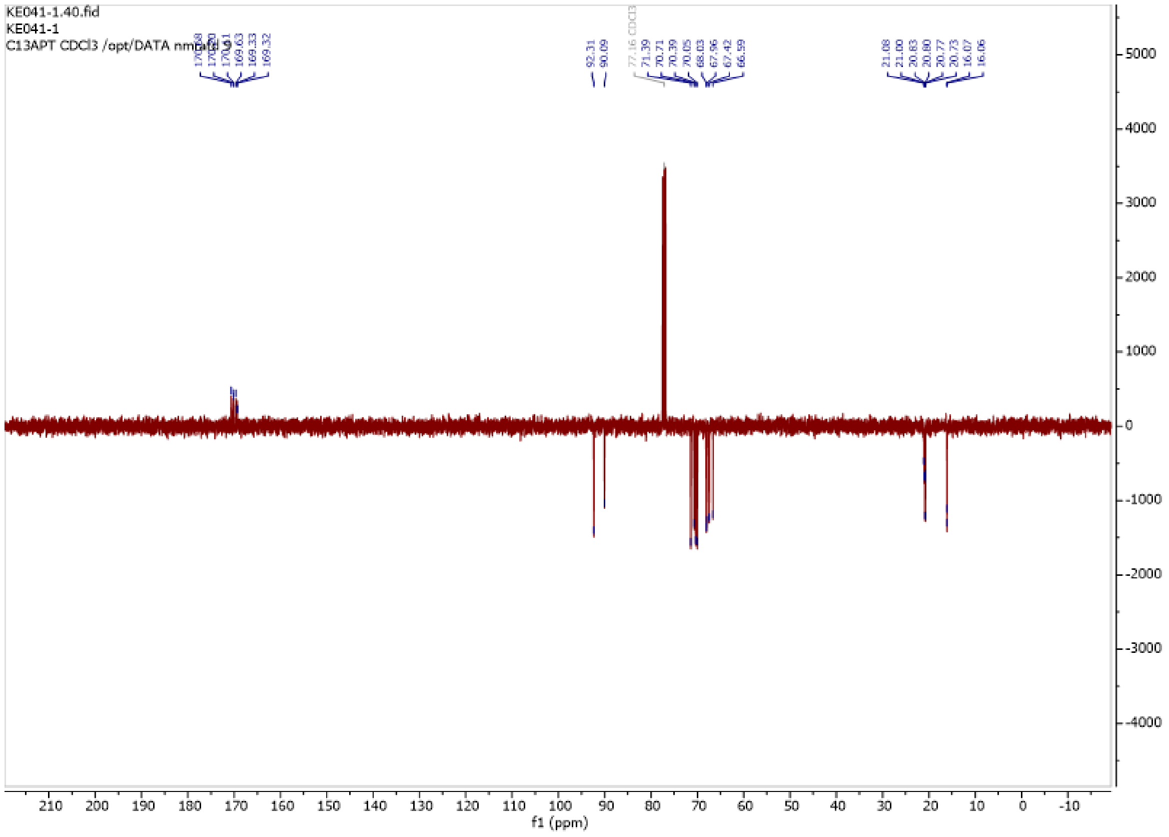
KE041-1.40.fid
KE041-1
C13APT CDCl3 /opt/DATA nmrafd 9
169.63
169.33
169.32
92.31
90.09
77.16 CDCl3
71.39
70.71
70.39
70.05
68.03
67.96
67.42
66.59
21.08
21.00
20.83
20.80
20.77
20.73
16.07
16.06
f1 (ppm)

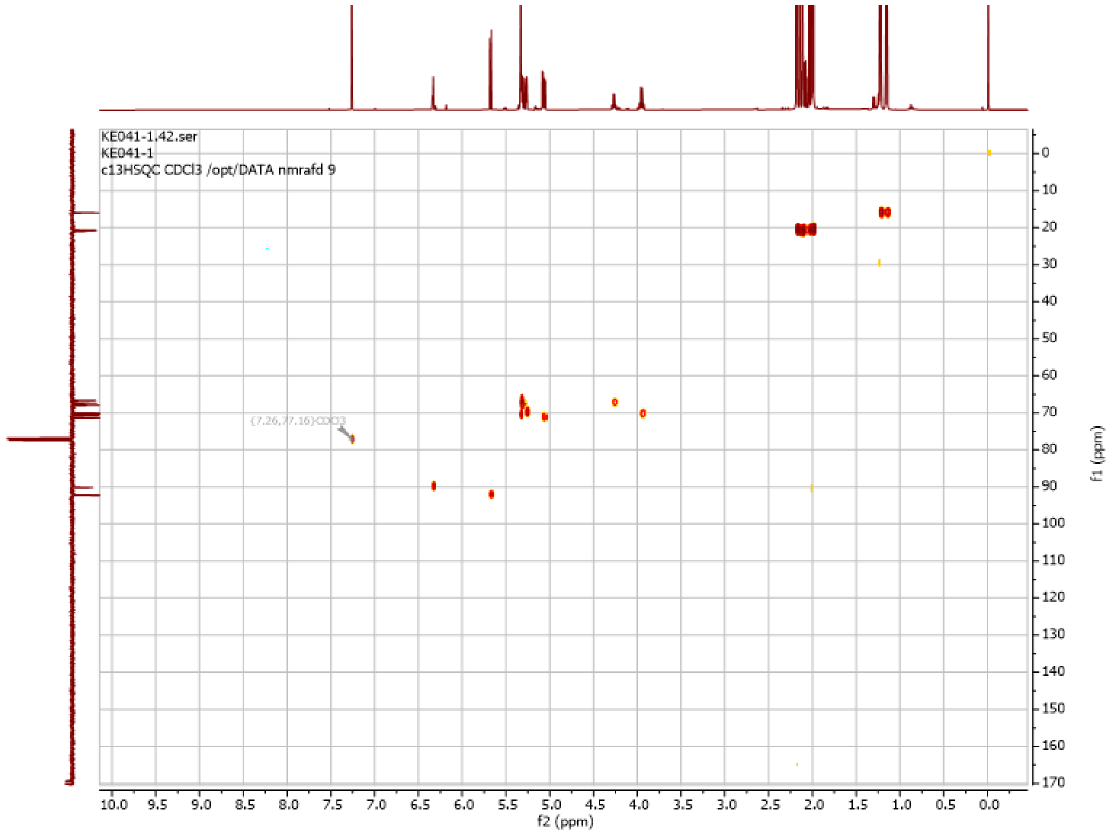
KE041-1.42.ser
KE041-1
c13HSQC CDCl3 /opt/DATA nmrafd 9
(7.26,77.16)CDCl3
f2 (ppm)
f1 (ppm)

3,4-di-*O*-acetyl-D-fucal (S2)

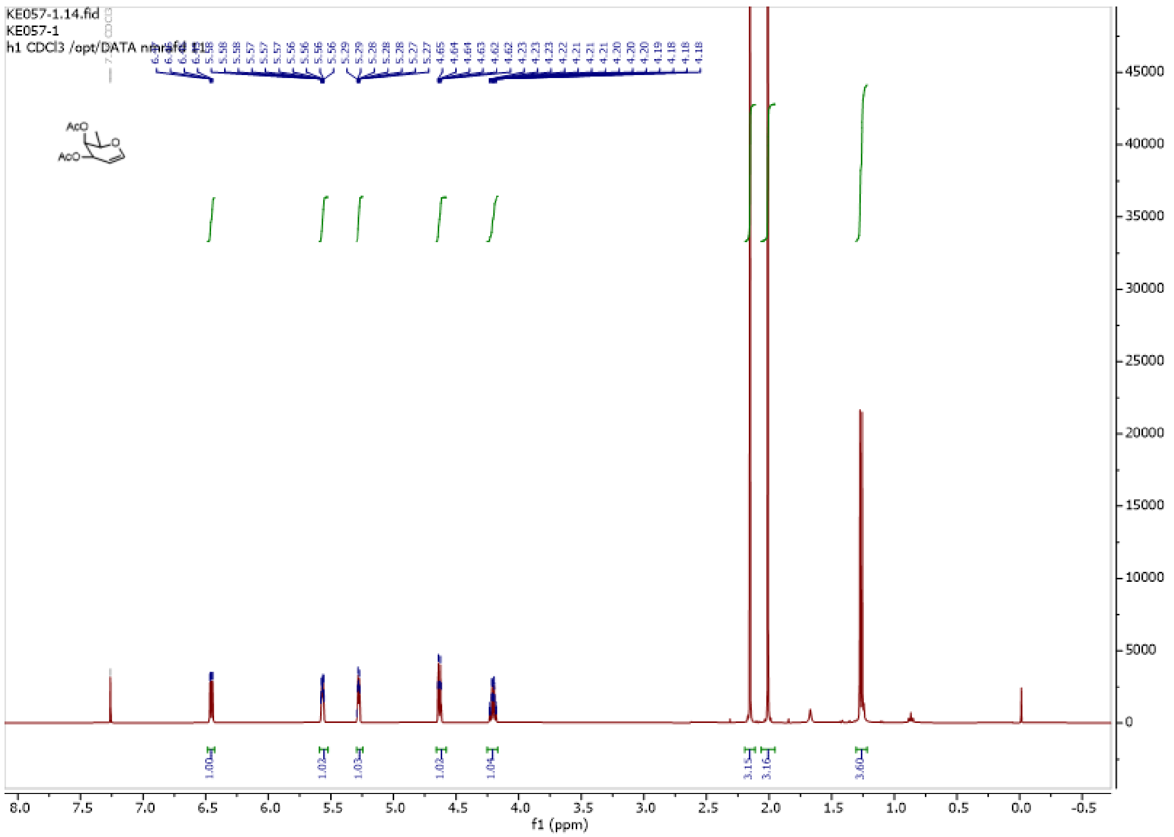

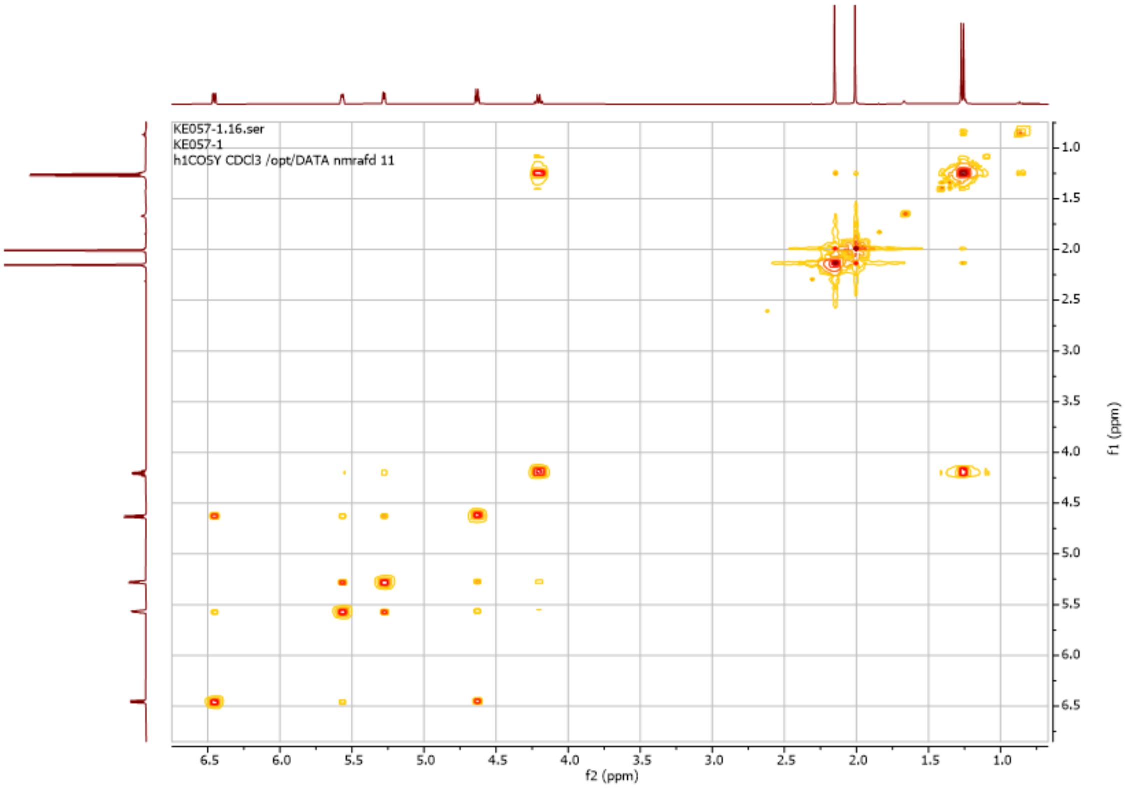

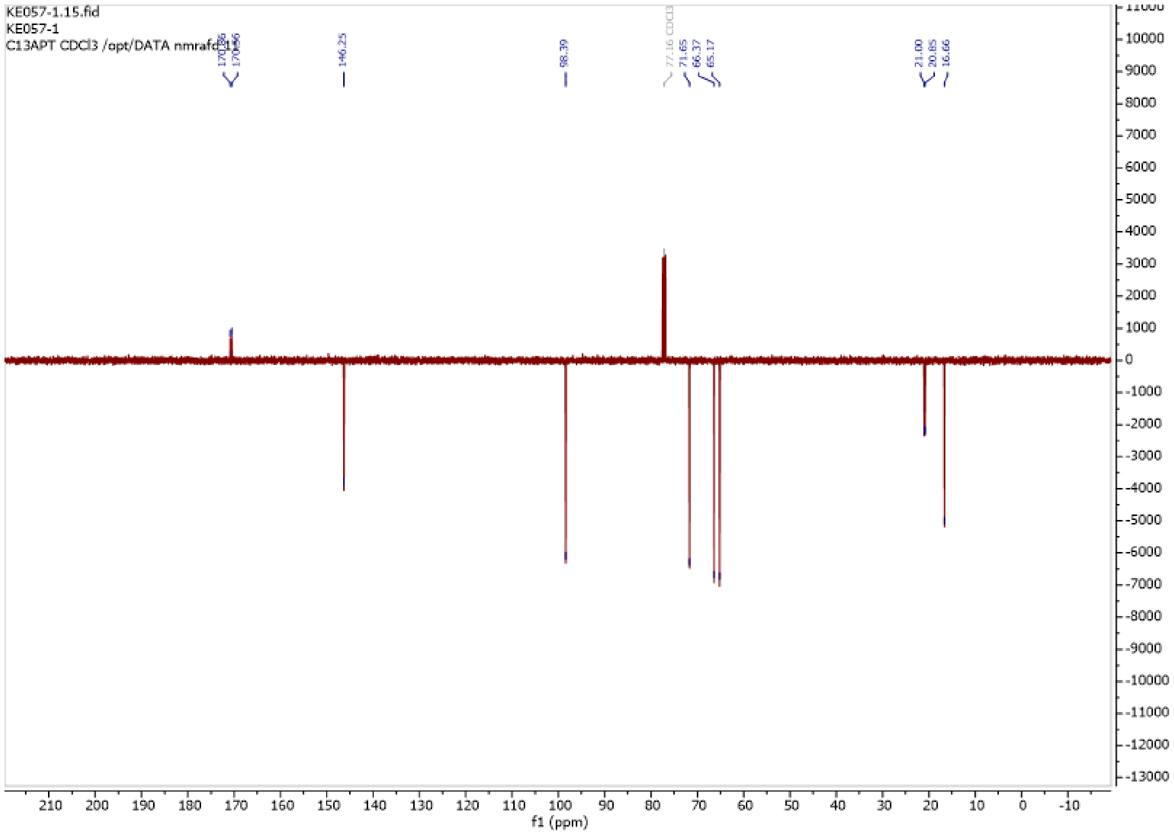
KE057-1.15.fid
KE057-1
C13APT CDCl3 /opt/DATA nmrafd 11
146.25
98.39
77.16 CDCl3
71.65
66.37
65.17
21.00
20.85
16.66
f1 (ppm)

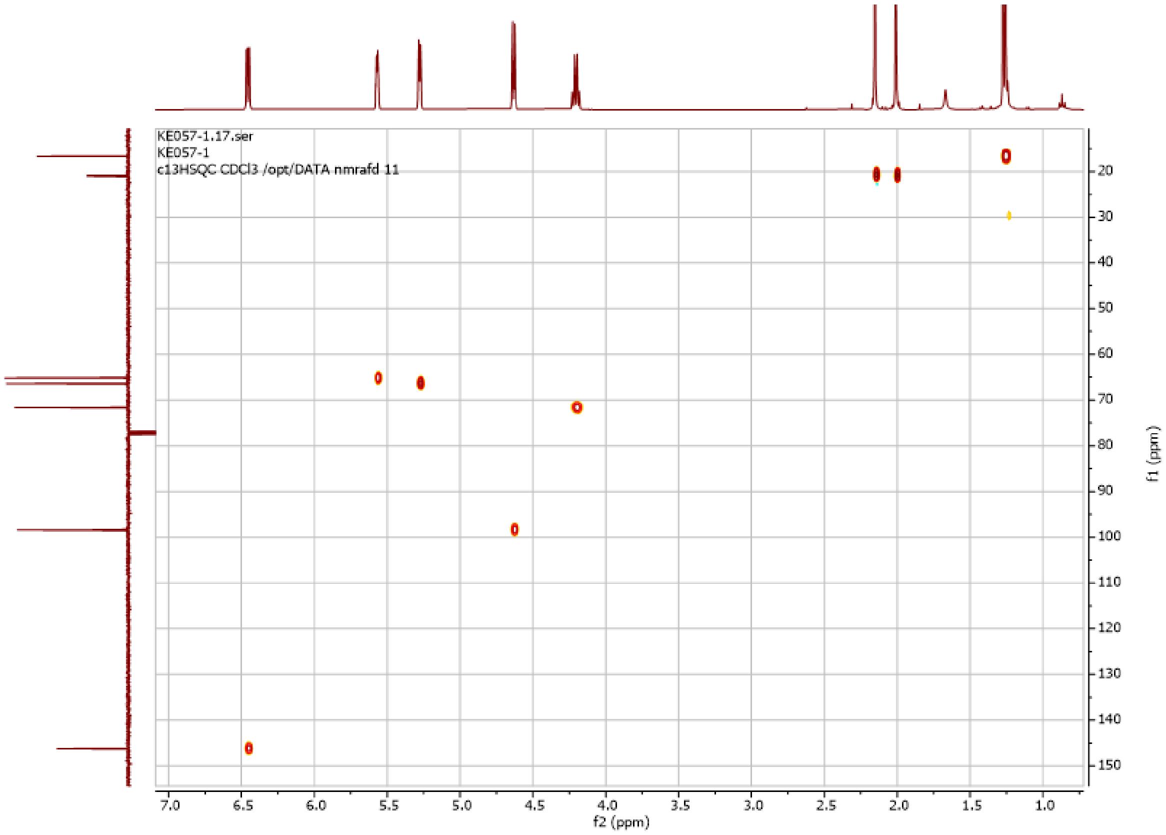
KE057-1.17.ser
KE057-1
c13HSQC CDCl3 /opt/DATA nmrafd 11
f2 (ppm)
f1 (ppm)

Phenyl 2-azido-2-deoxy-1-seleno-*α*-D-fucopyranoside (S3)

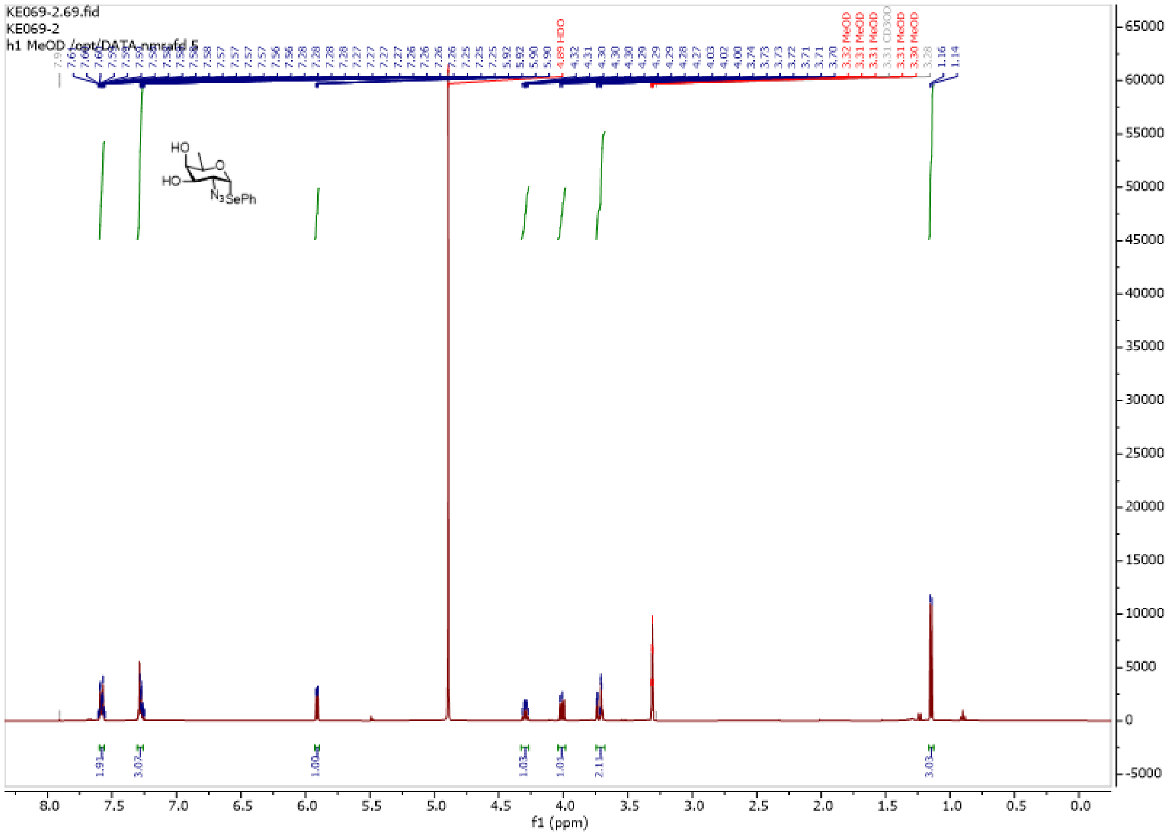

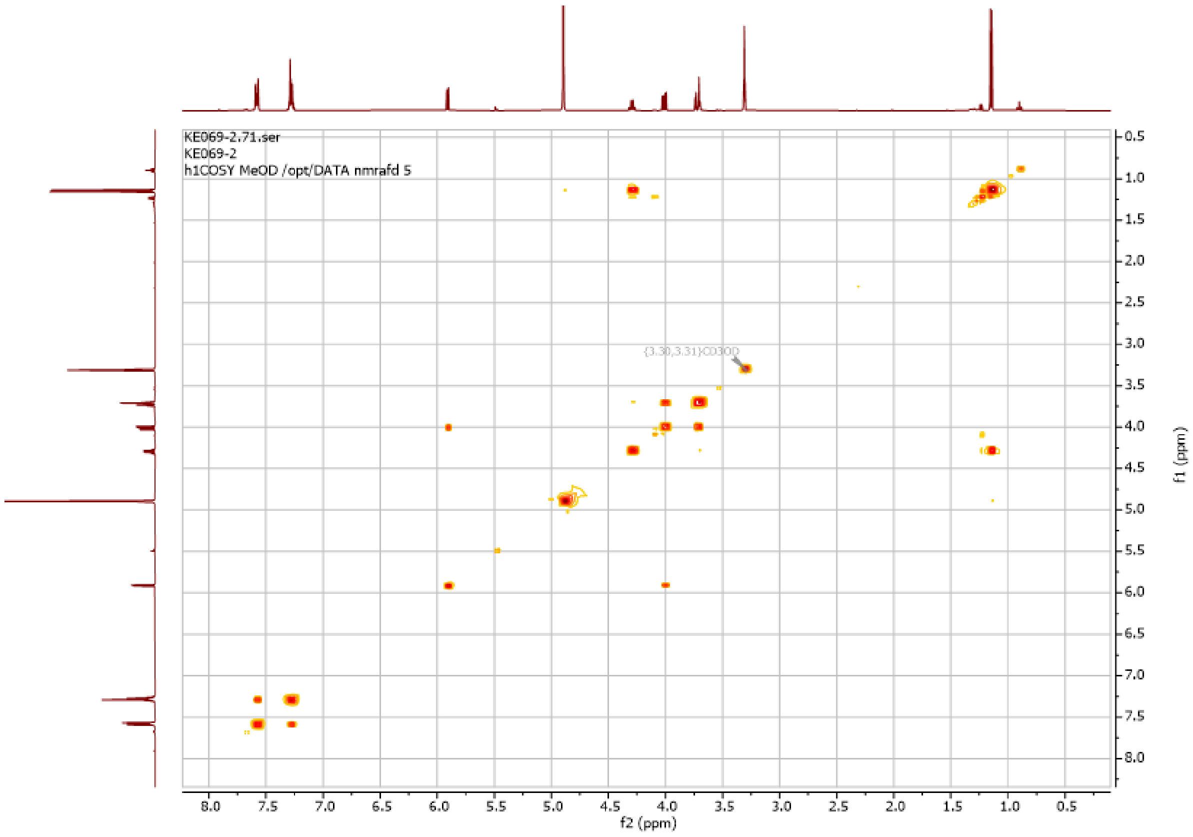

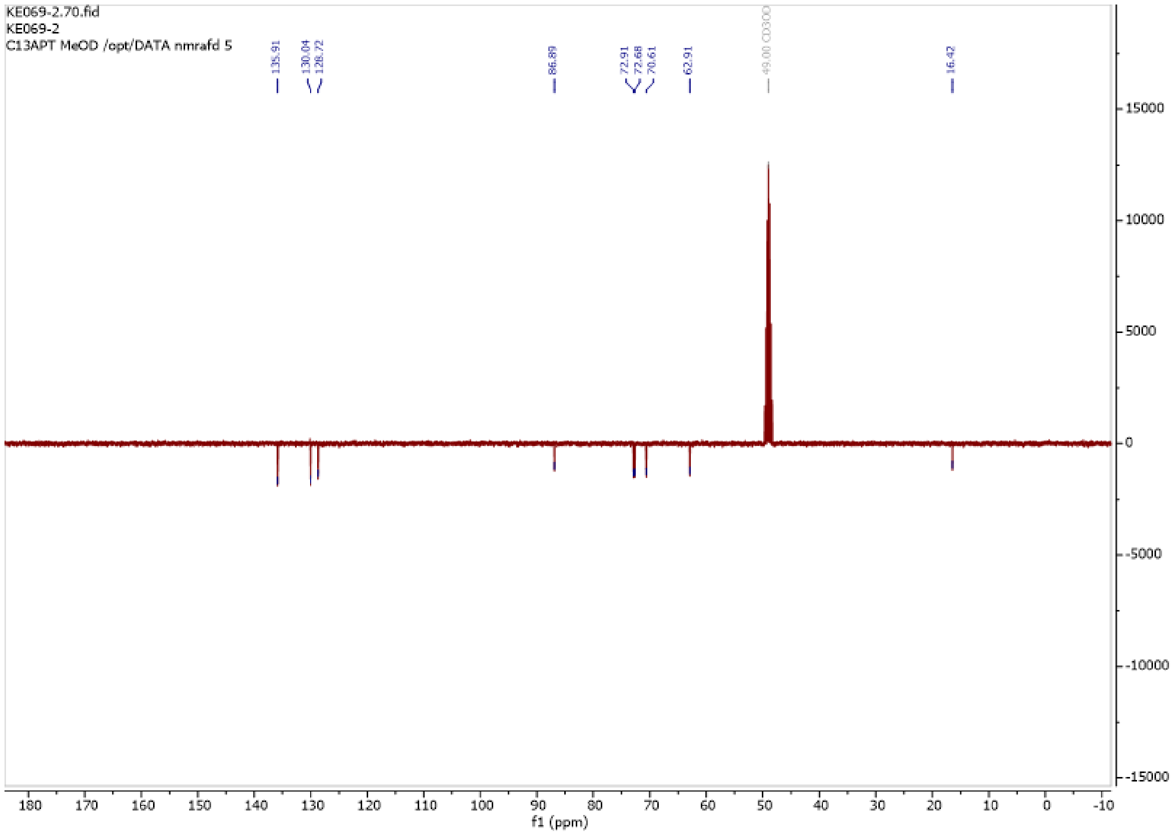
KE069-2.70.fid
KE069-2
C13APT MeOD /opt/DATA nmrafd 5
135.91
130.04
128.72
86.89
72.91
72.68
70.61
62.91
49.00 CD3OD
16.42
f1 (ppm)

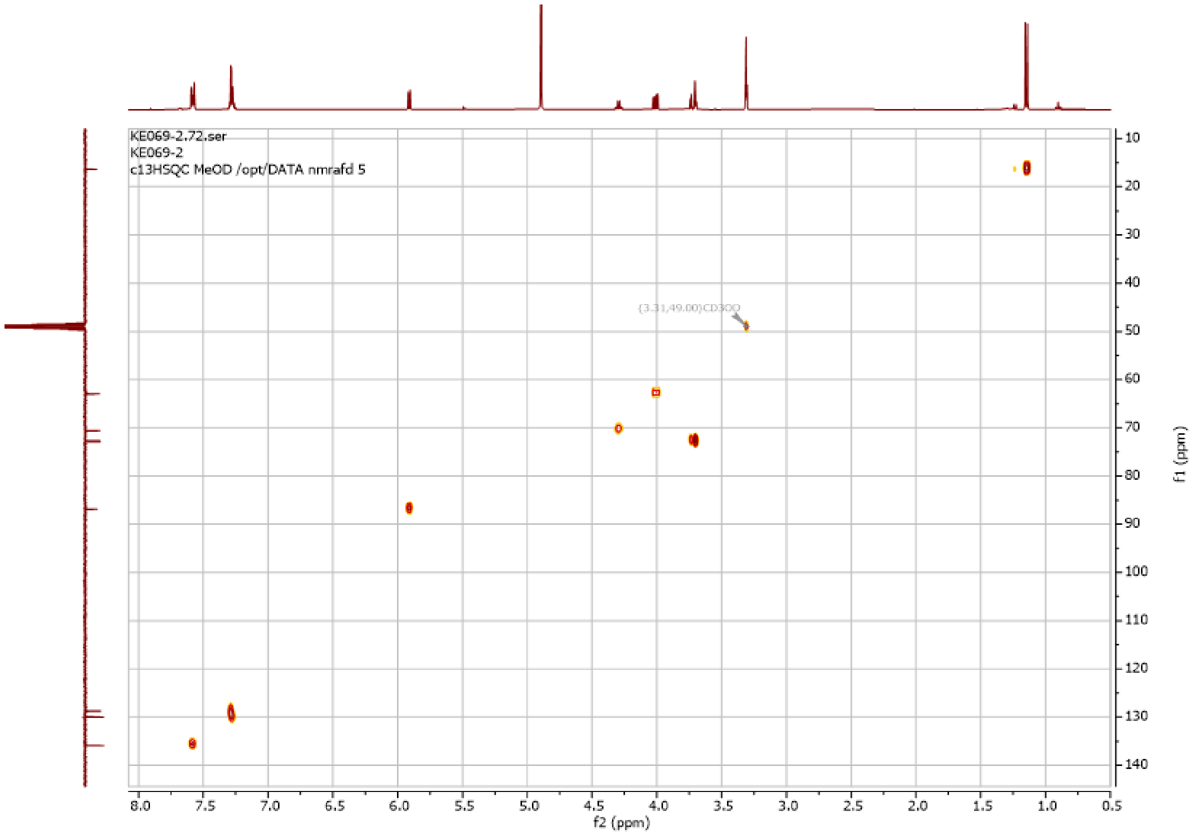
KE069-2.72.ser
KE069-2
c13HSQC MeOD /opt/DATA nmrafd 5
{3.31,49.00}CD3OD
f2 (ppm)
f1 (ppm)

Phenyl 2-azido-2-deoxy-3-*O*-(2-naphthylmethyl)-1-seleno-*α*-D-fucopyranoside (S4)

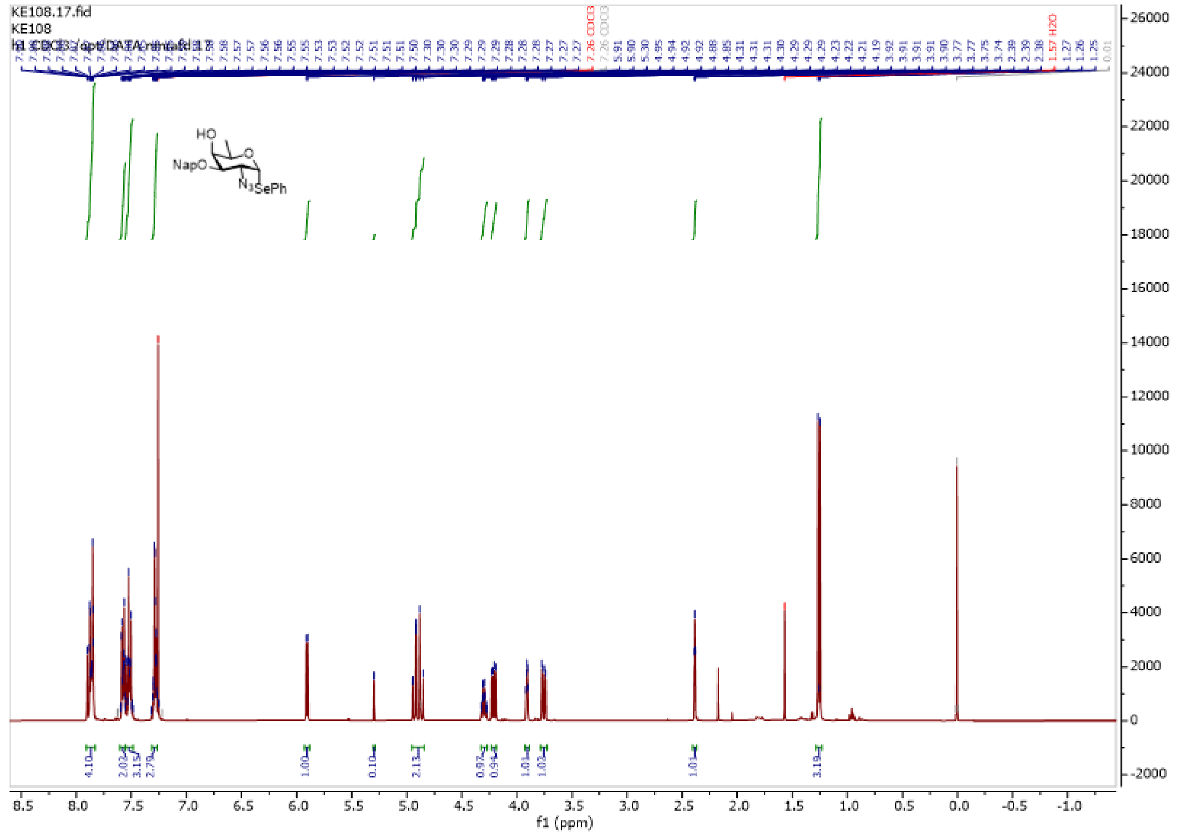

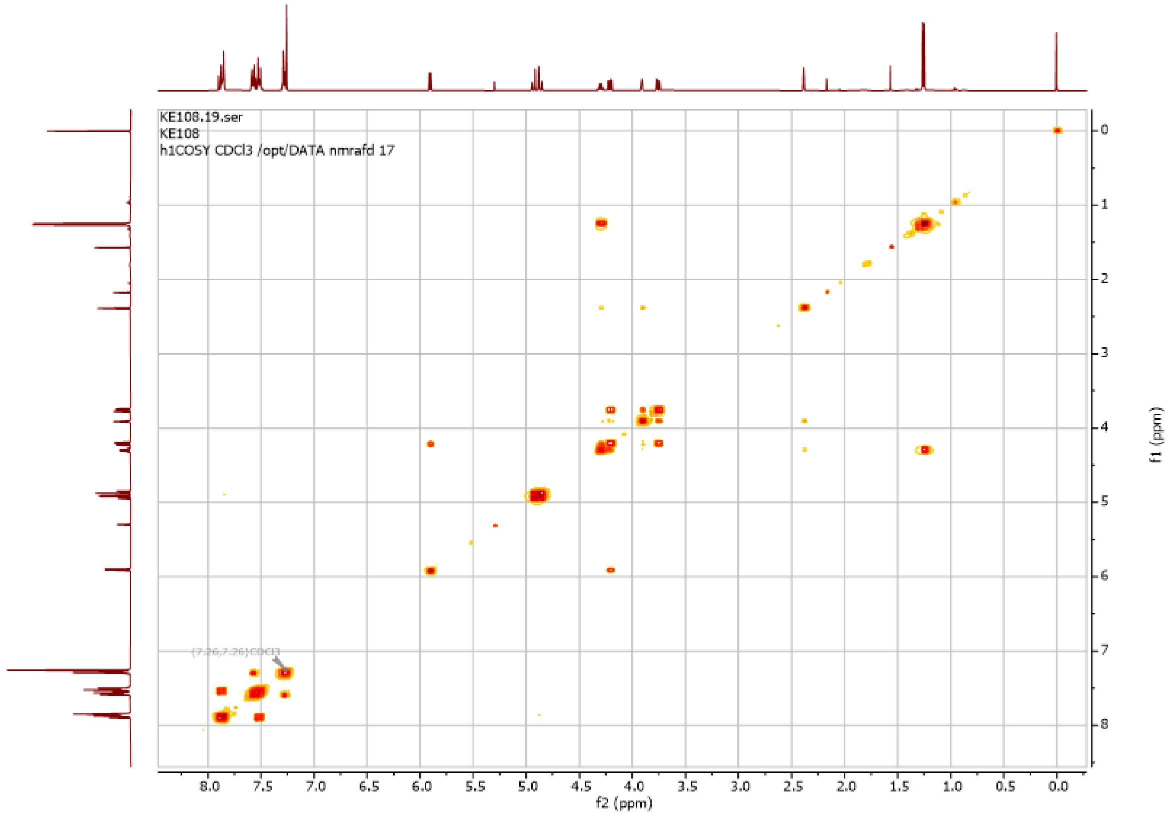

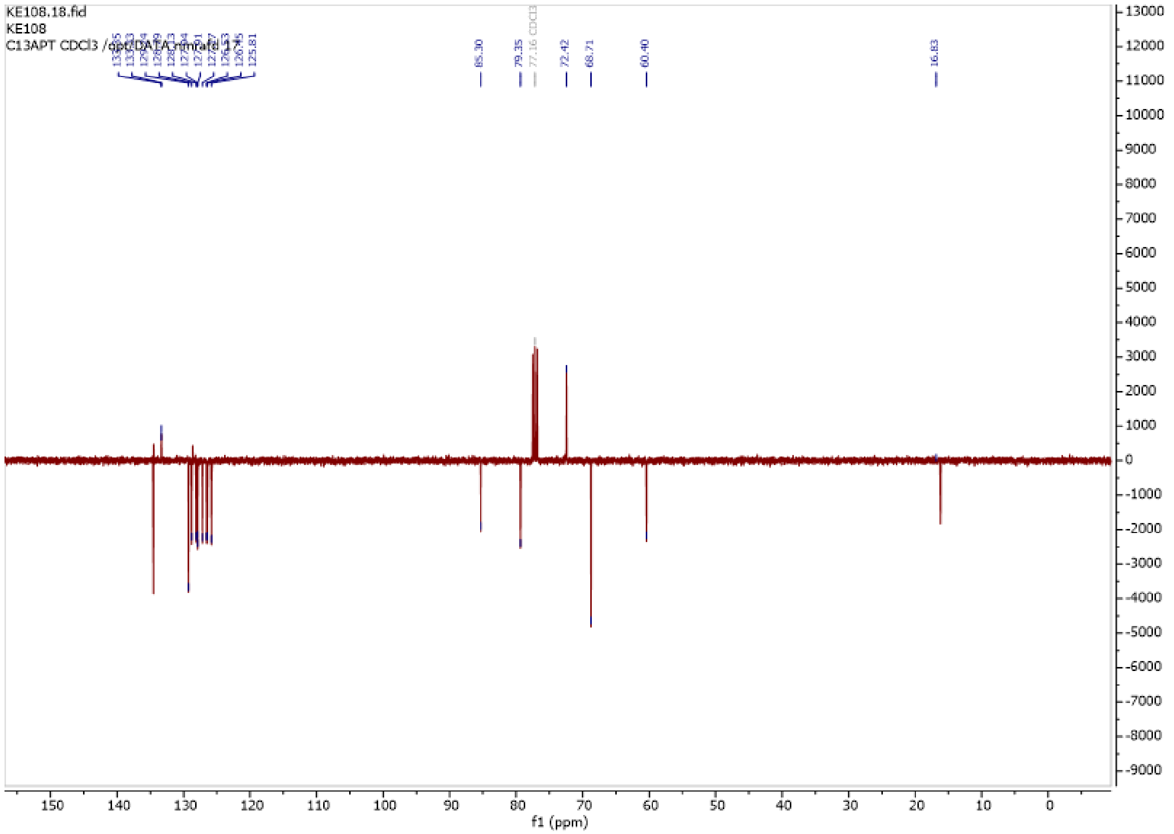
KE108.18.fid
KE108
C13APT CDCl3 /opt/DATA nmrafd 17
85.30
79.35
77.16 CDCl3
72.42
68.71
60.40
16.83
125.81
f1 (ppm)

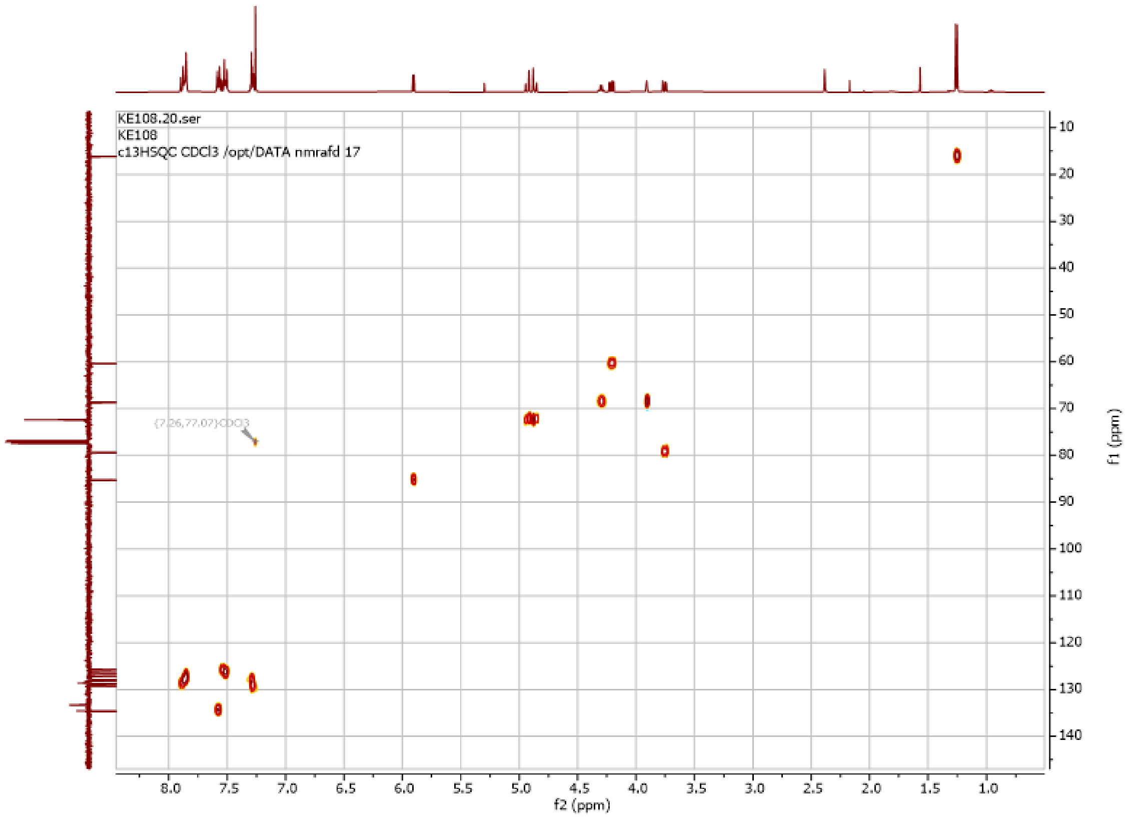
KE108.20.ser
KE108
c13HSQC CDCl3 /opt/DATA nmrafd 17
f2 (ppm)
f1 (ppm)

Phenyl 2-azido-4-*O*-benzyl-2-deoxy-3-*O*-(2-naphthylmethyl)-1-seleno-*α*-D-fucopyranoside (S5)

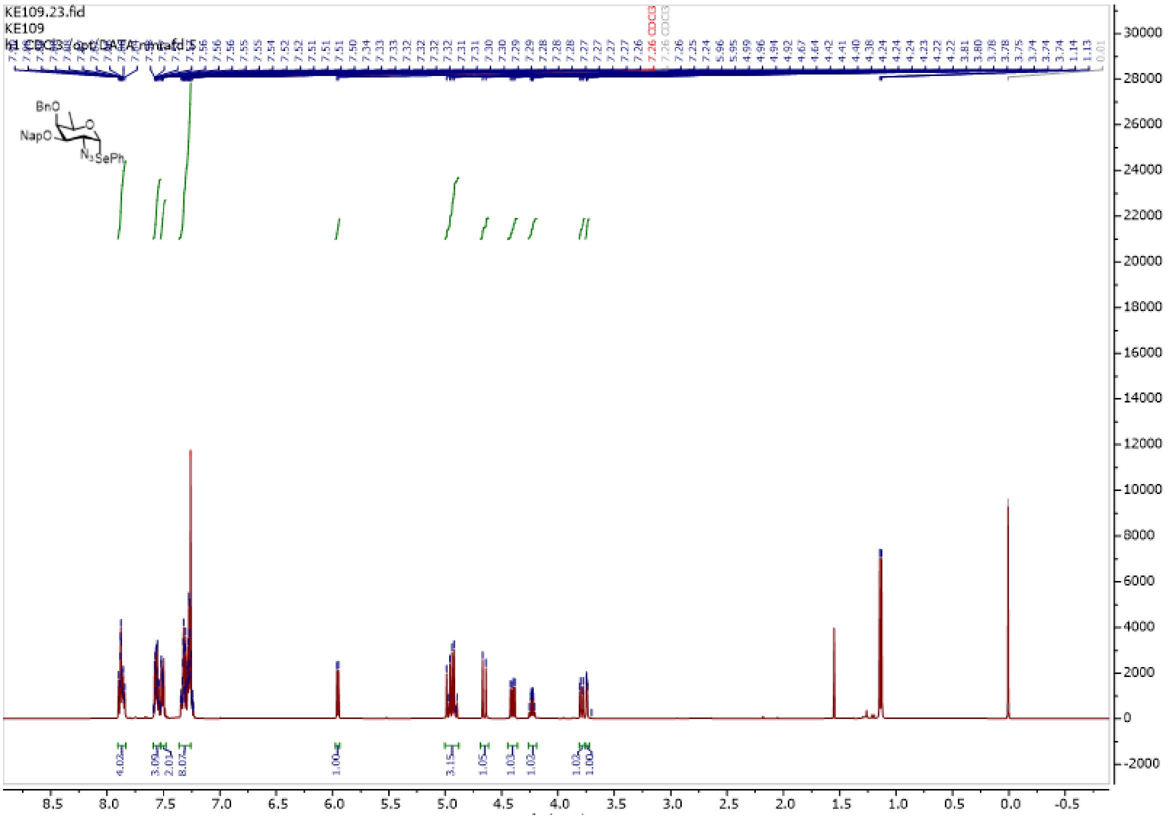

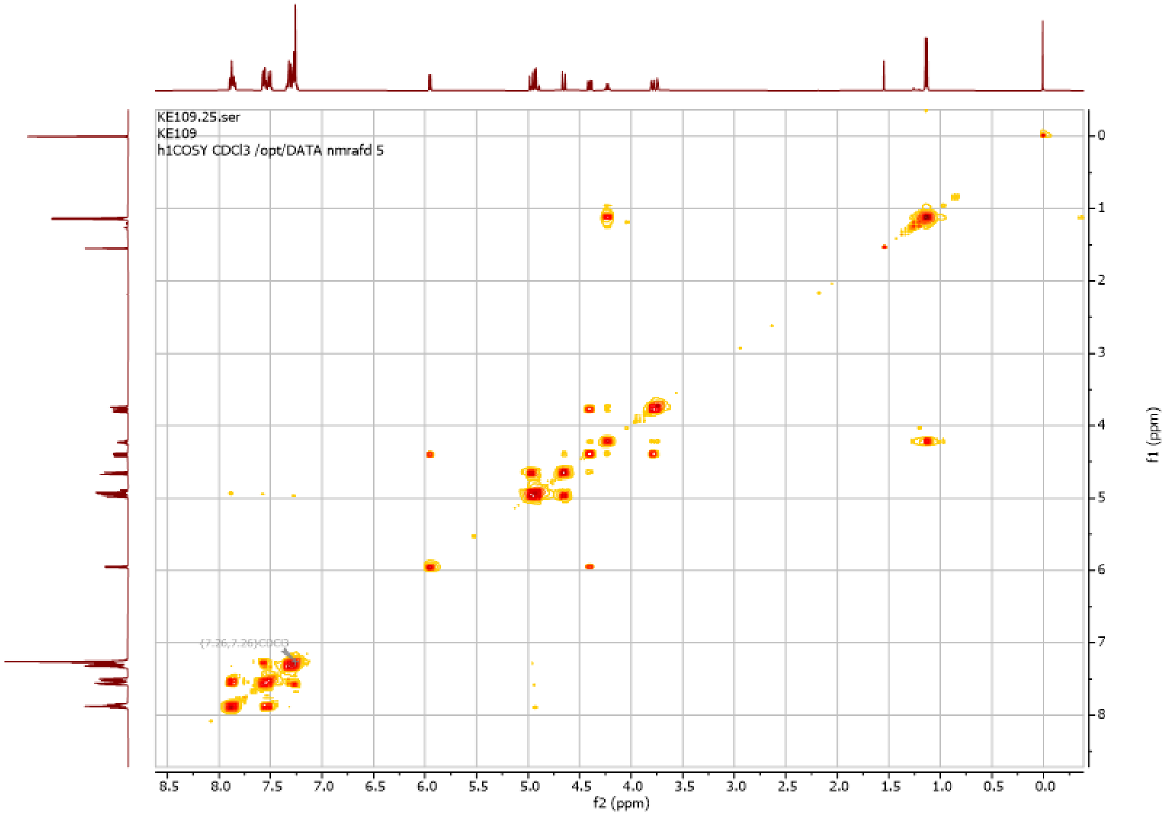

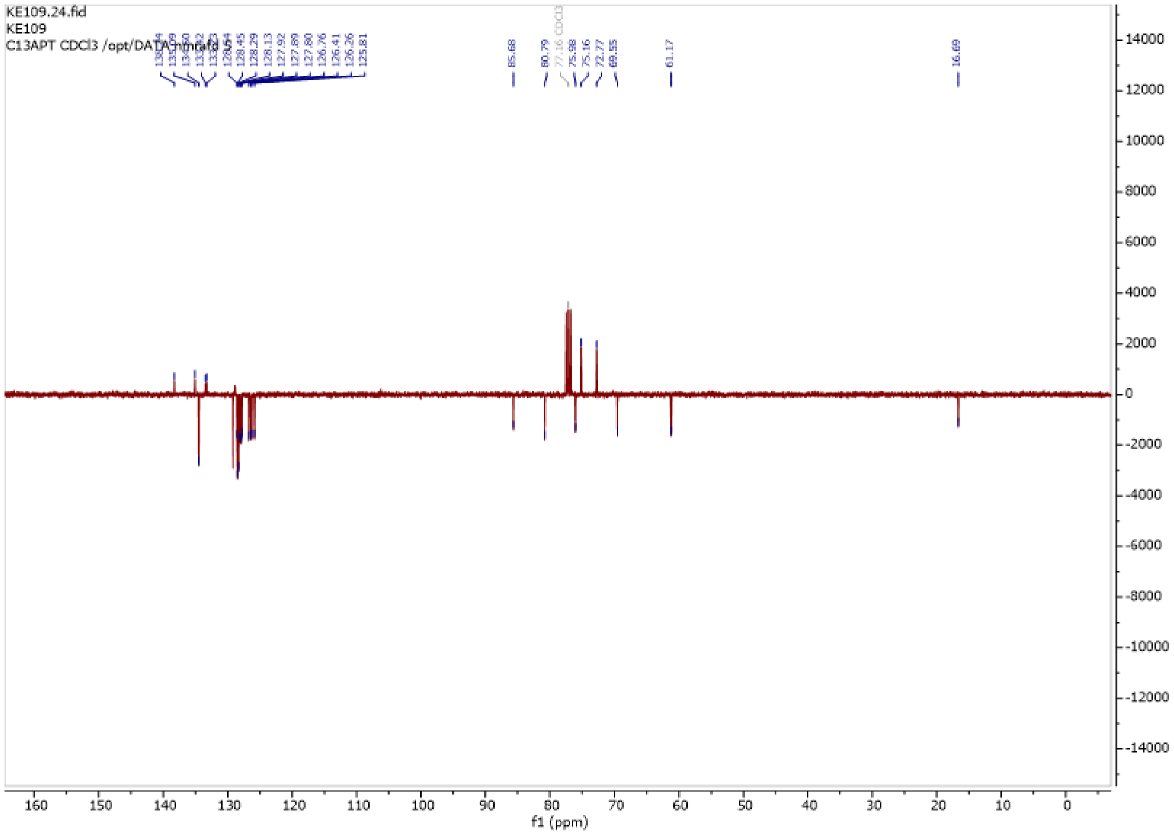
KE109.24.fid
KE109
C13APT CDCl3 /opt/DATA nmrafd 5
85.68
80.79
77.16 CDCl3
75.98
75.16
72.77
69.55
61.17
16.69
f1 (ppm)

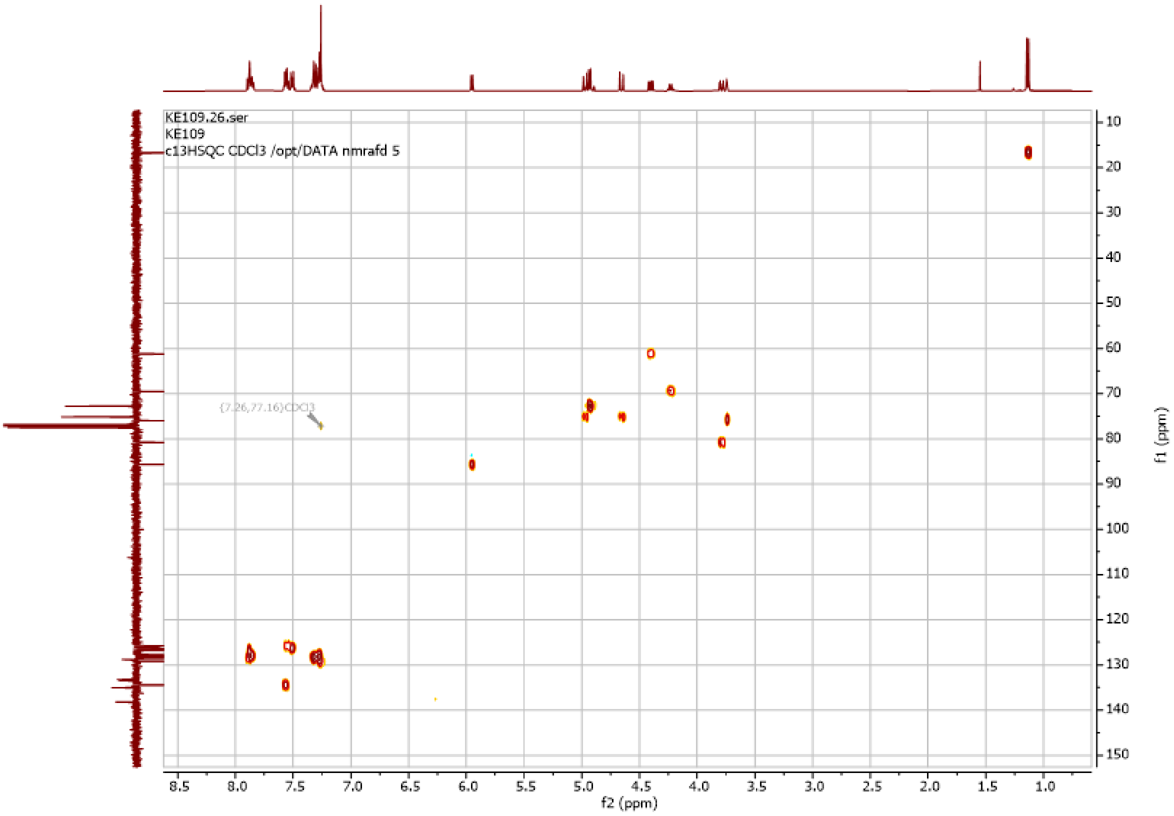
KE109.26.ser
KE109
c13HSQC CDCl3 /opt/DATA nmrafd 5
{7.26,77.16}CDCl3
f2 (ppm)
f1 (ppm)

2-azido-4-*O*-benzyl-2-deoxy-3-*O*-(2-naphthylmethyl)-*α*/*β*-D-fucopyranose (S6)

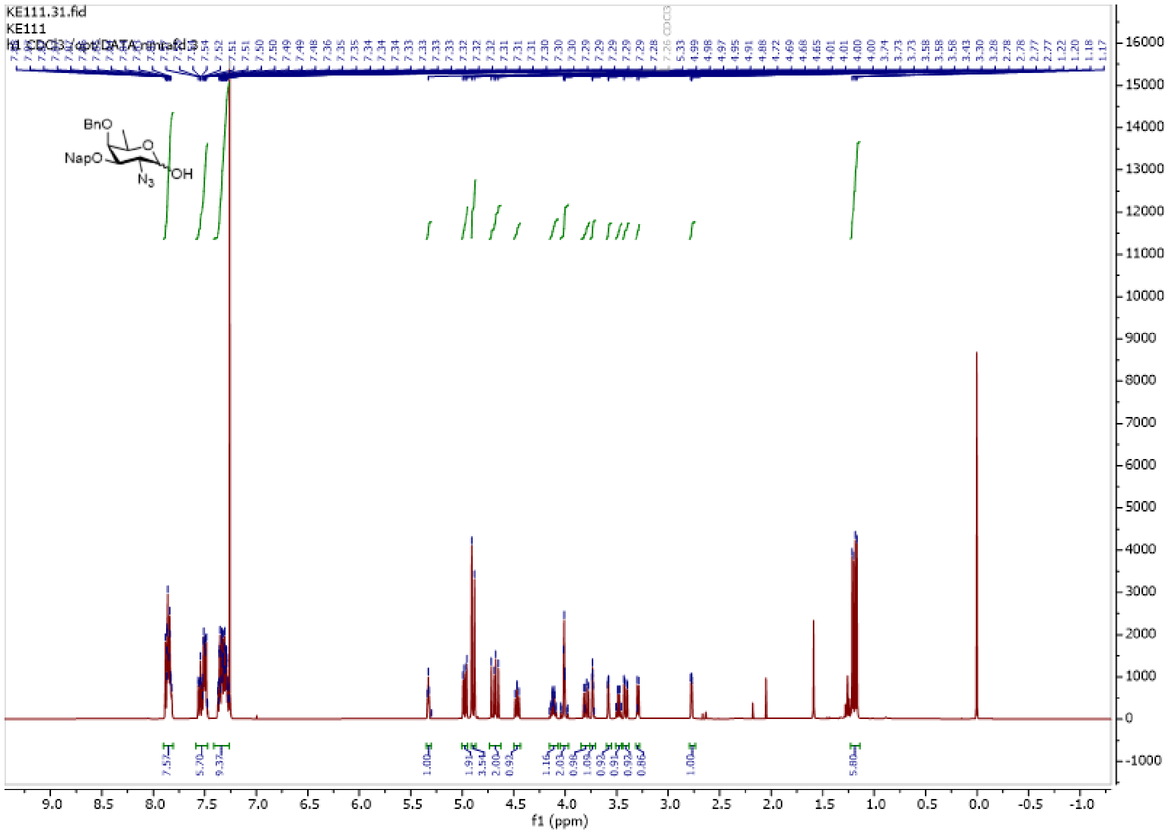

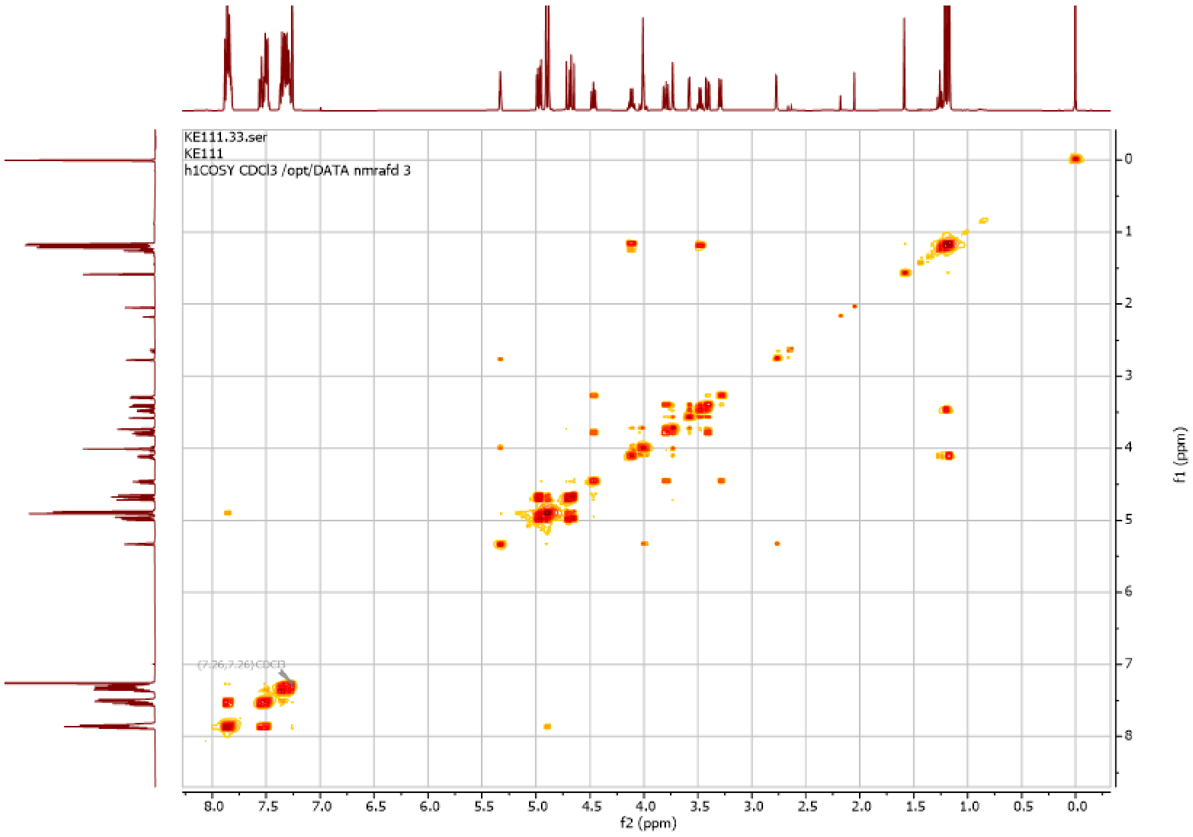

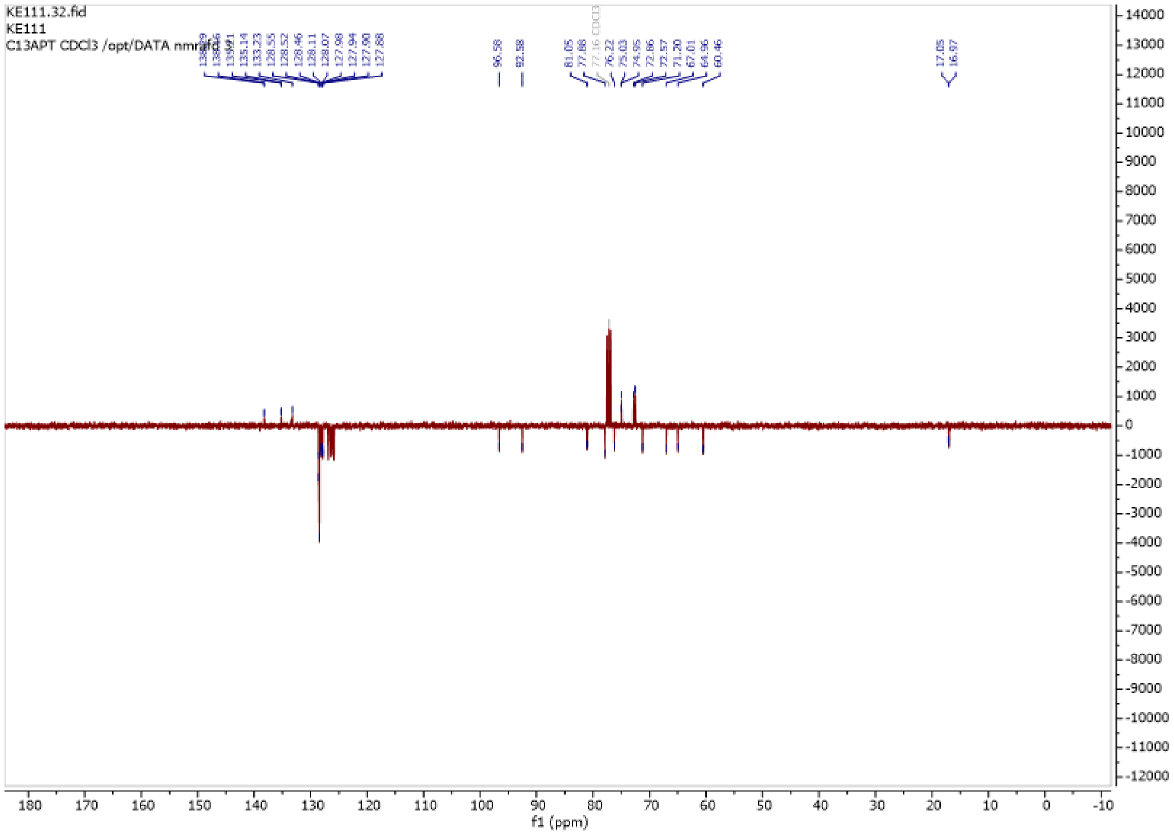
KE111.32.fid
KE111
C13APT CDCl3 /opt/DATA nmrafd
96.58
92.58
81.05
77.88
76.22
75.03
74.95
72.86
72.57
71.20
67.01
64.96
60.46
17.05
16.97
f1 (ppm)

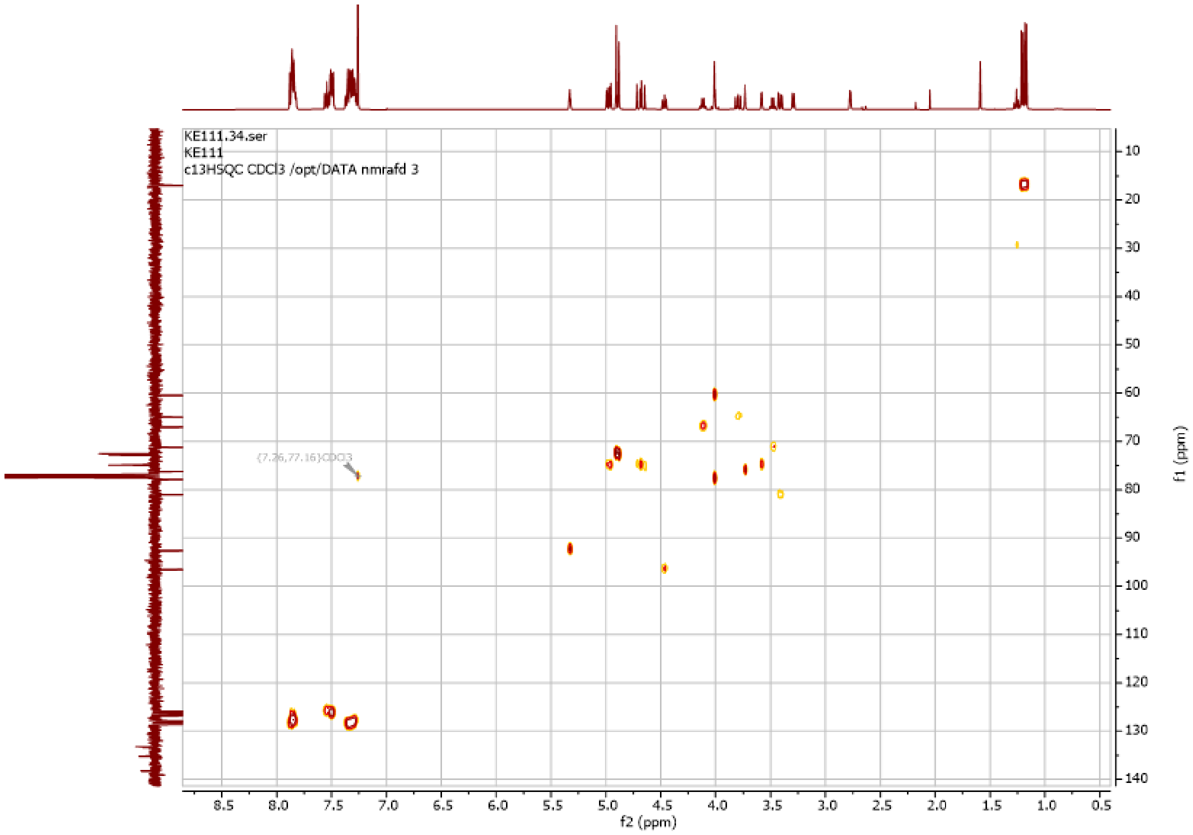
KE111.34.ser
KE111
c13HSQC CDCl3 /opt/DATA nmrafd 3
f2 (ppm)
f1 (ppm)

***Tert*-butyldiphenylsilyl 2-azido-4-*O*-benzyl-2-deoxy-3-*O*-(2-naphthylmethyl)-*β*-D-fucopyranoside (S7)**

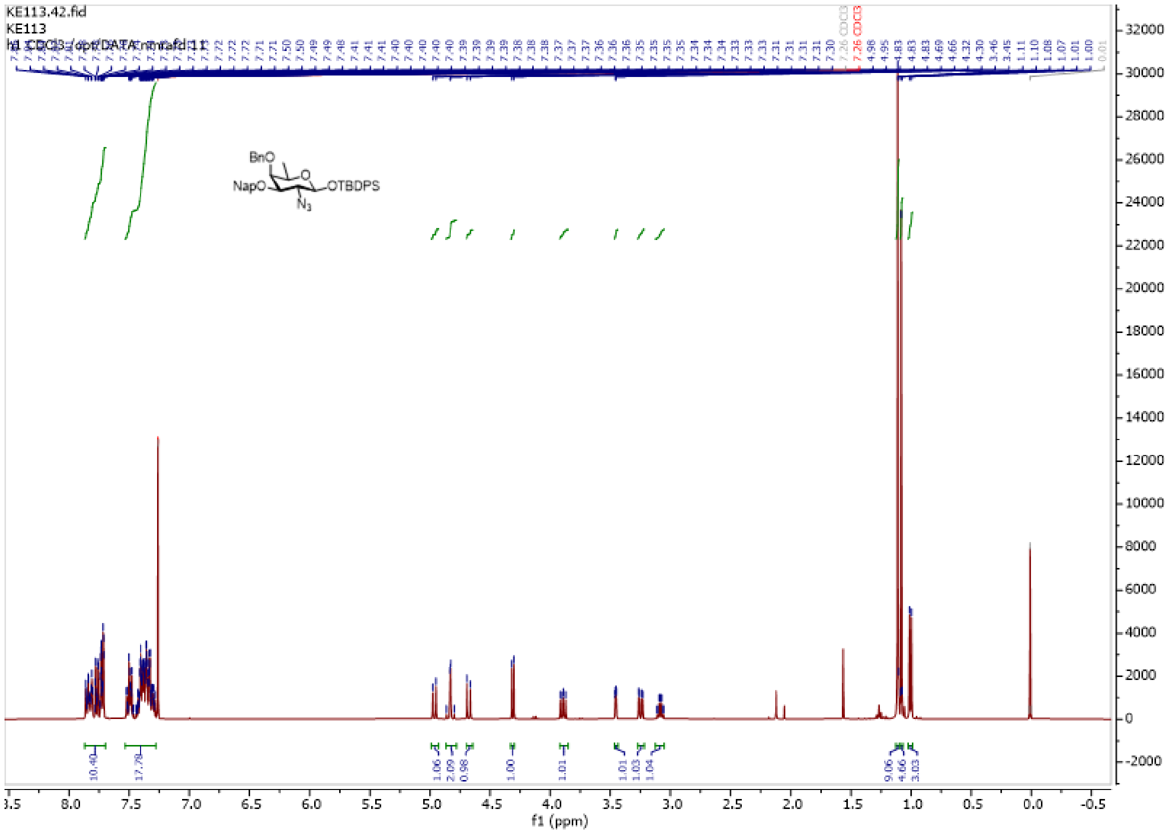

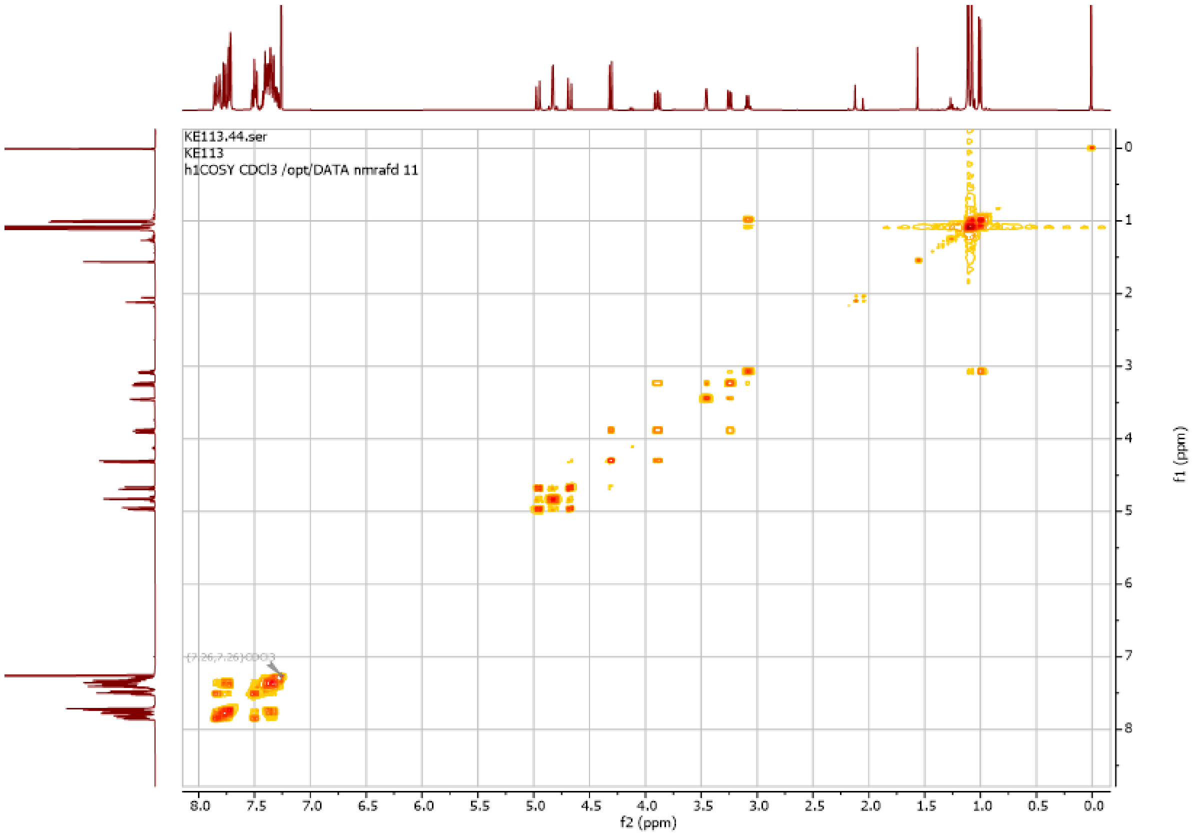

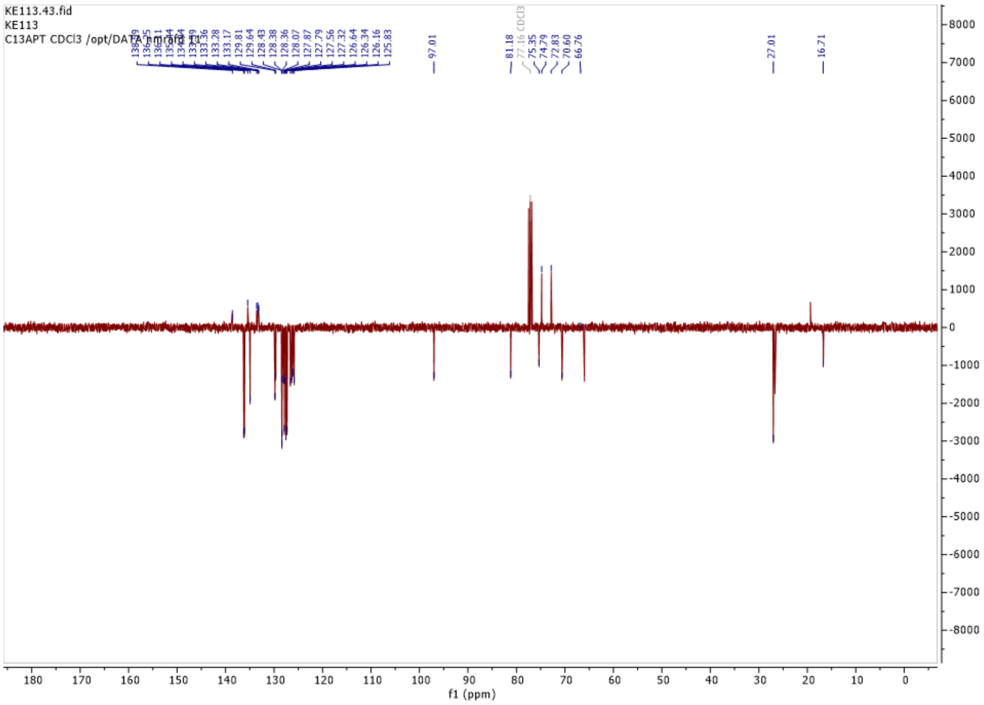

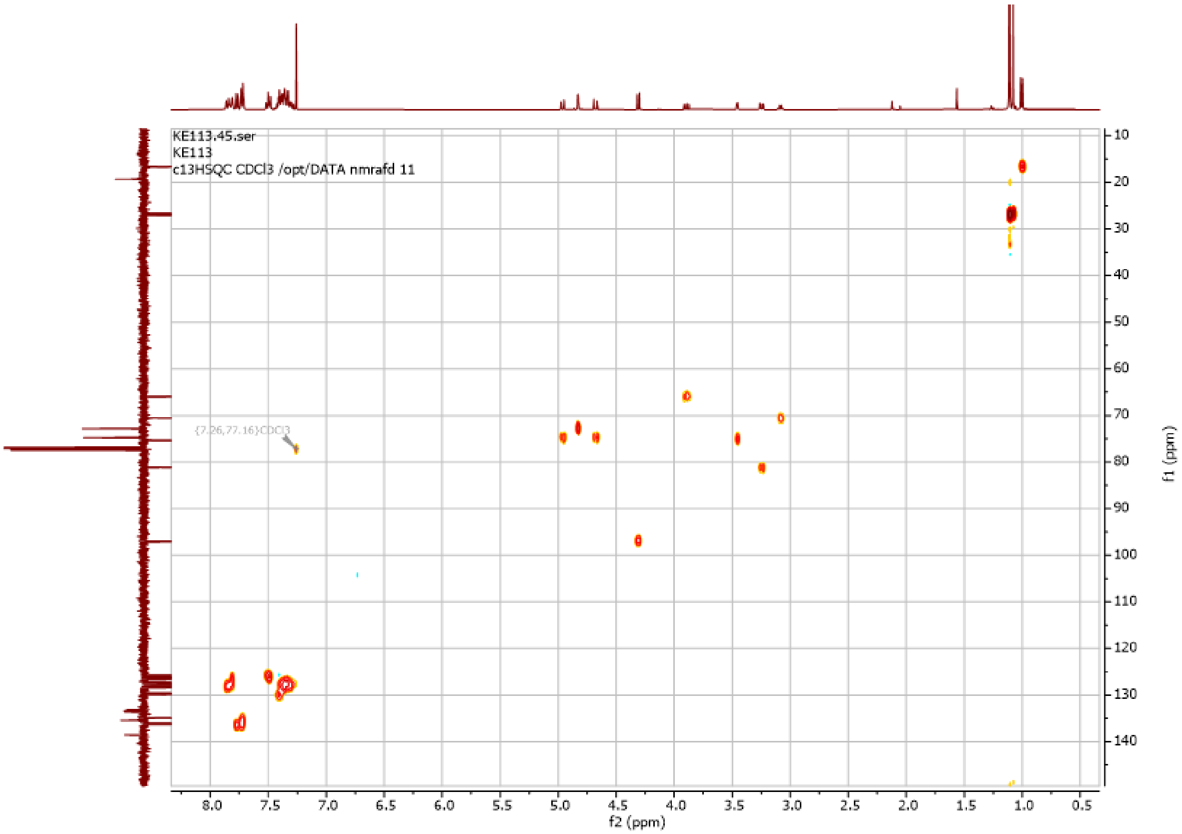

***Tert*-butyldiphenylsilyl 2-azido-4-*O*-benzyl-2-deoxy-*β*-D-fucopyranoside (10)**

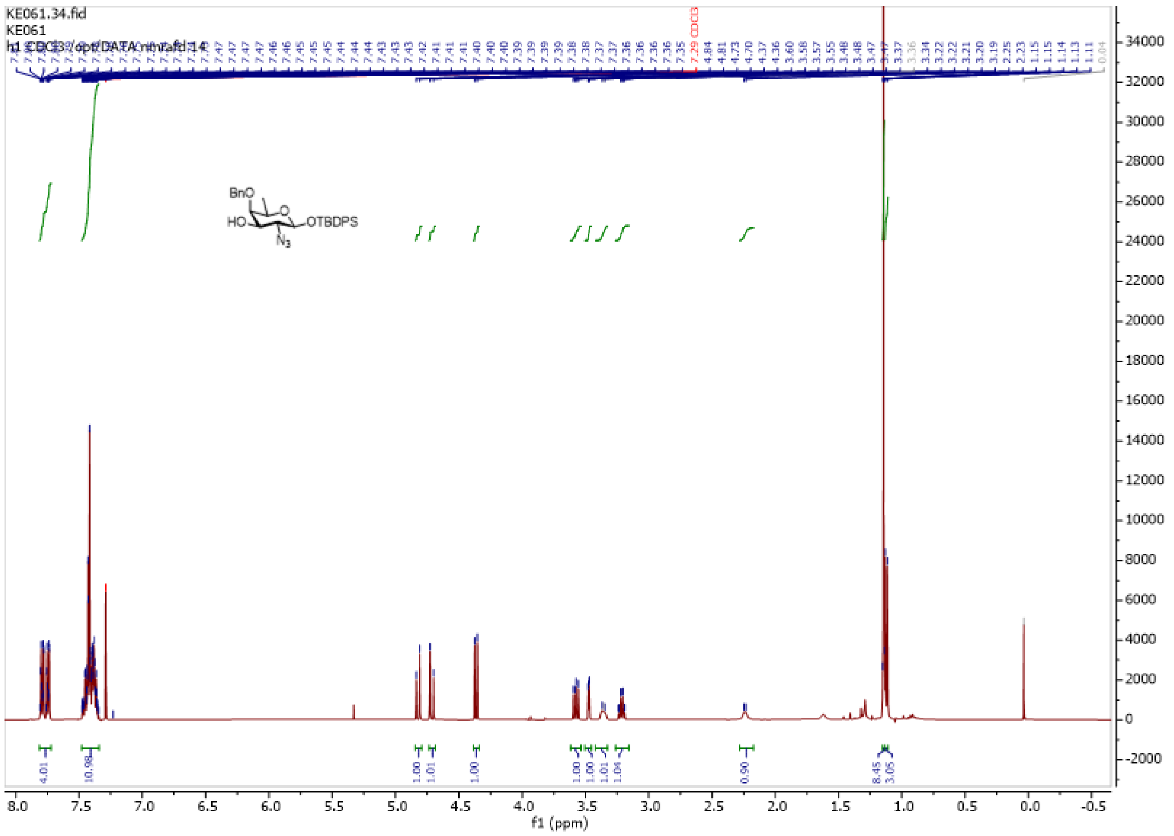

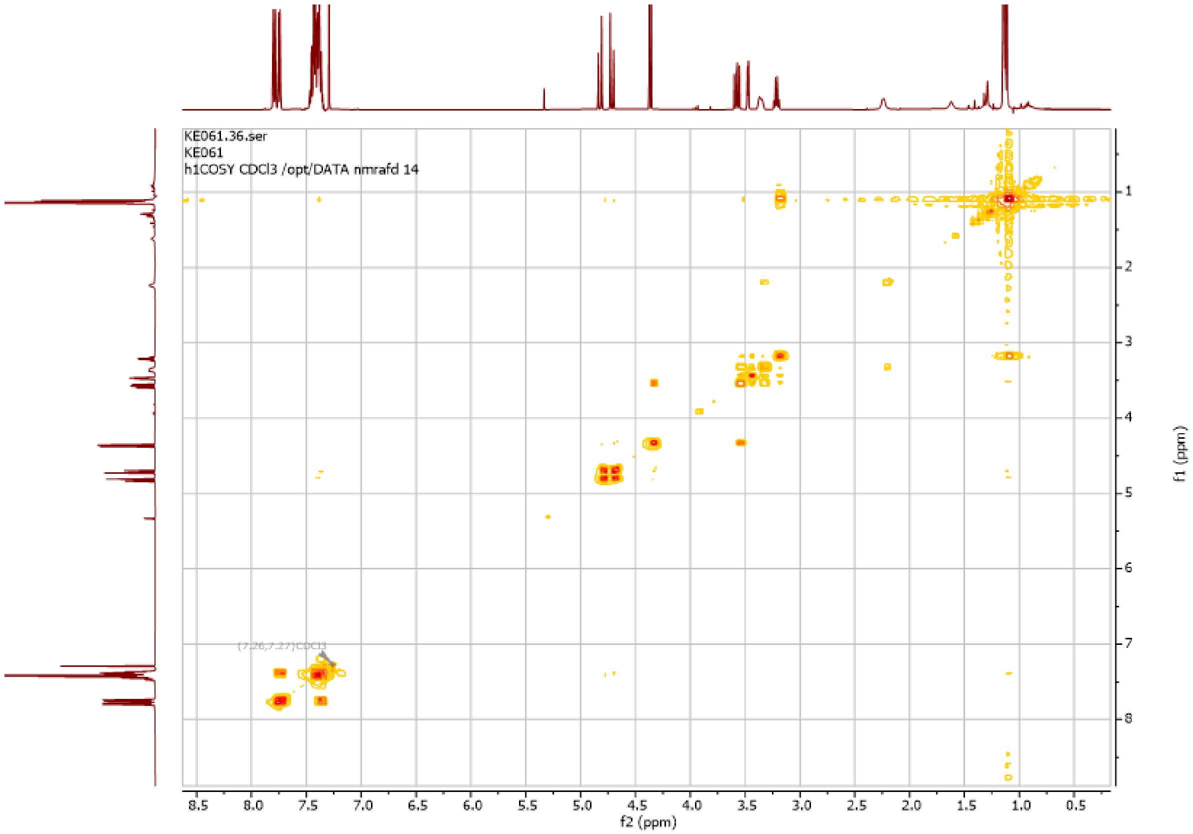

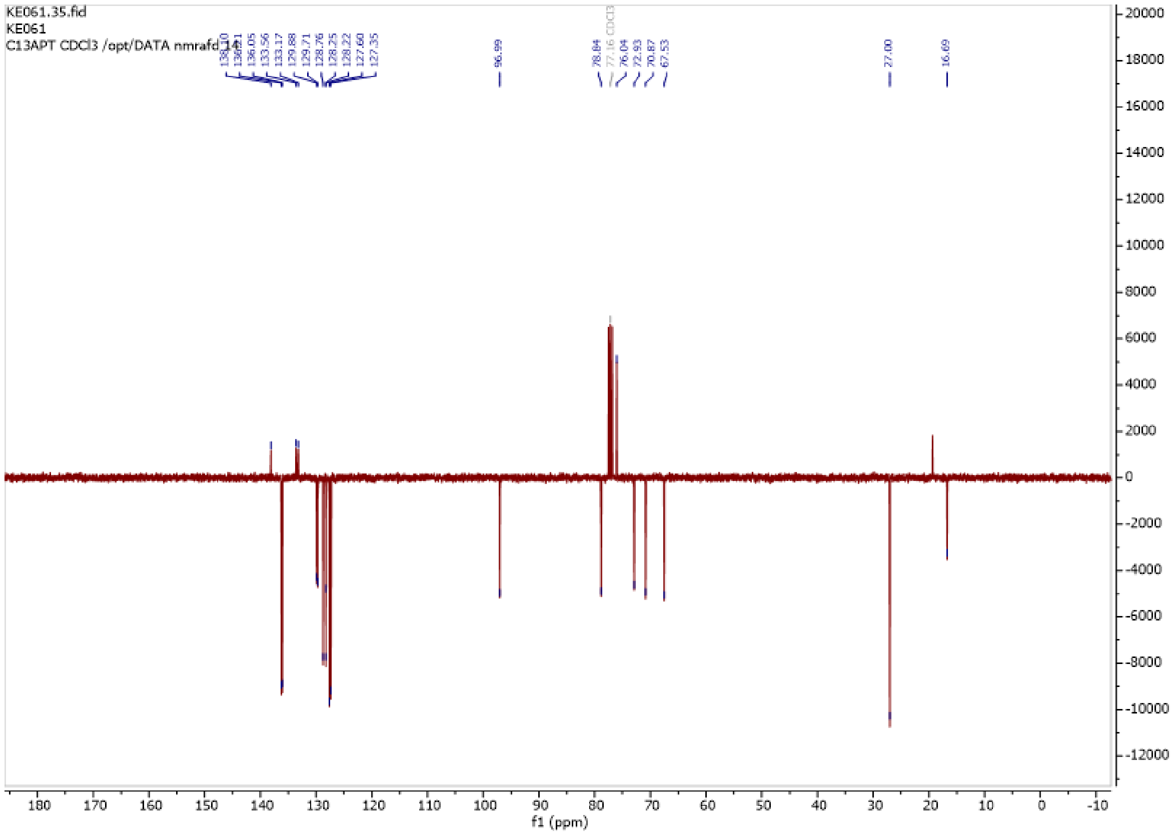
KE061.35.fid
KE061
C13APT CDCl3 /opt/DATA nmrafd 14
96.99
78.84
77.16 CDCl3
76.04
72.93
70.87
67.53
27.00
16.69
f1 (ppm)

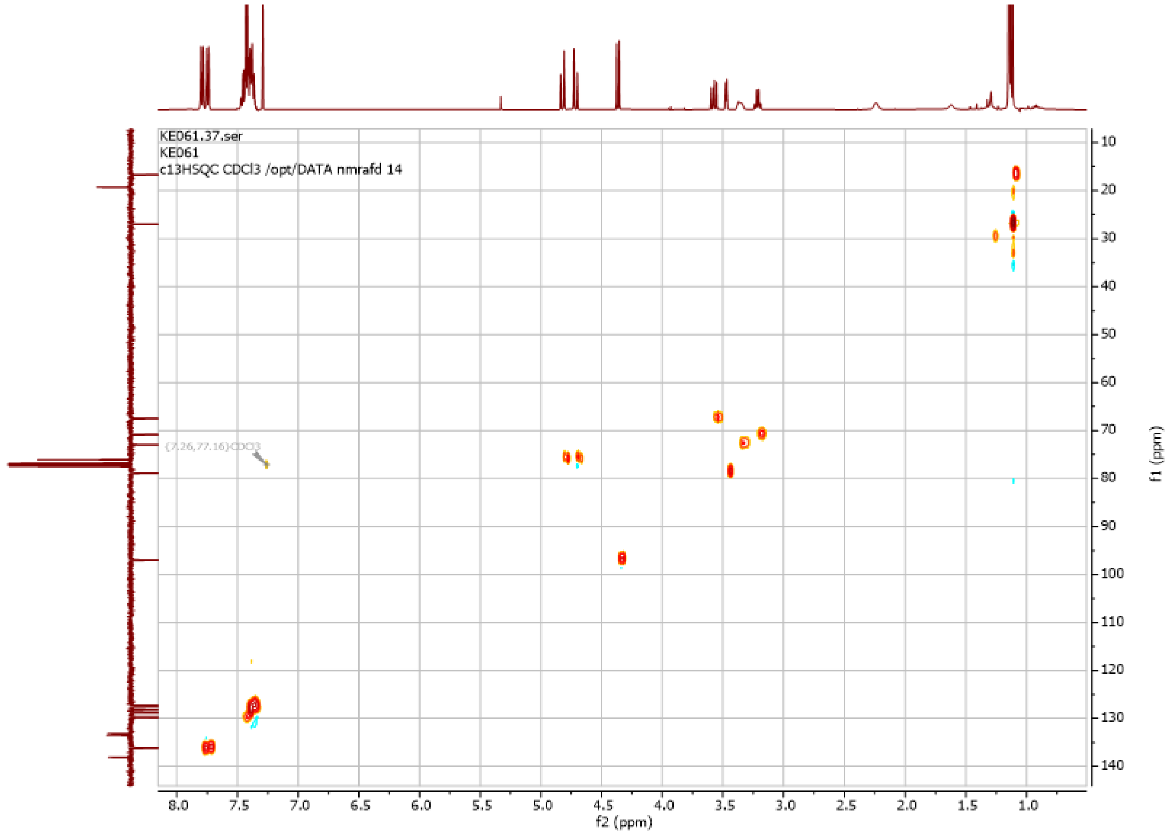
KE061.37.ser
KE061
c13HSQC CDCl3 /opt/DATA nmrafd 14
f2 (ppm)
f1 (ppm)

3,4-di-*O*-acetyl-L-fucal (S8)

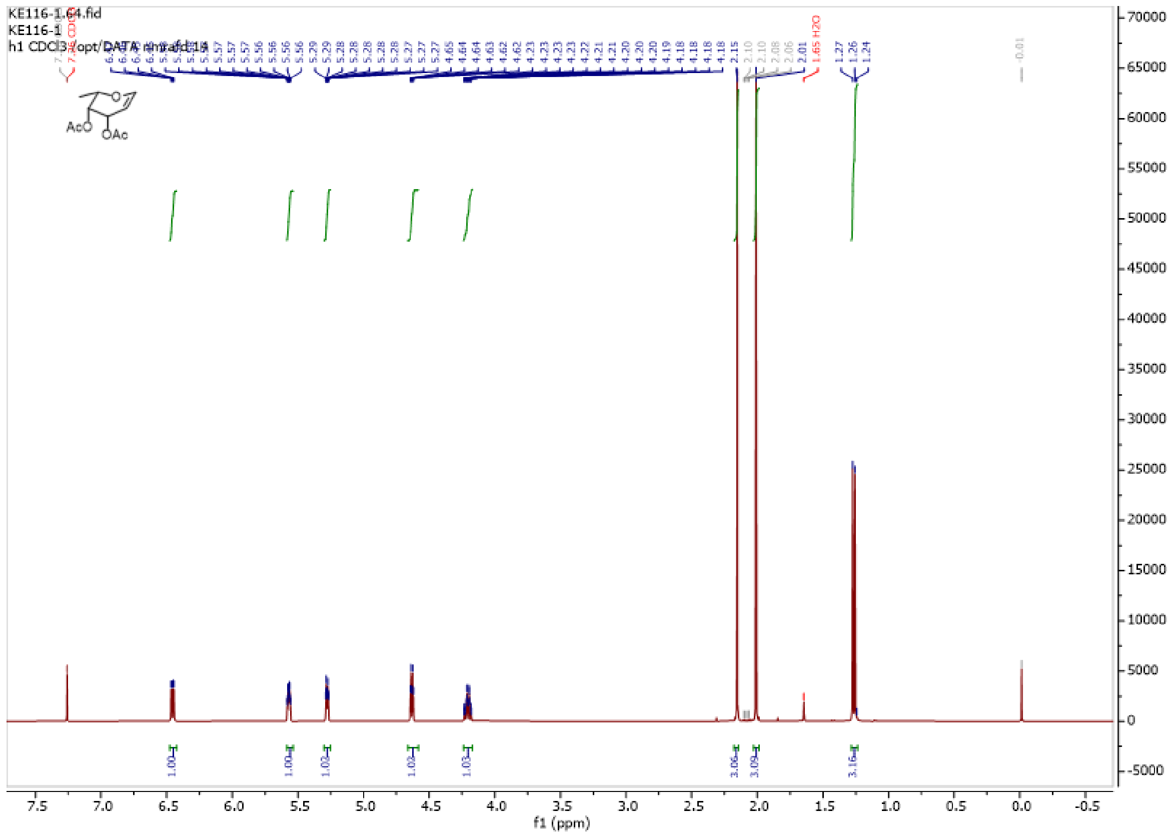

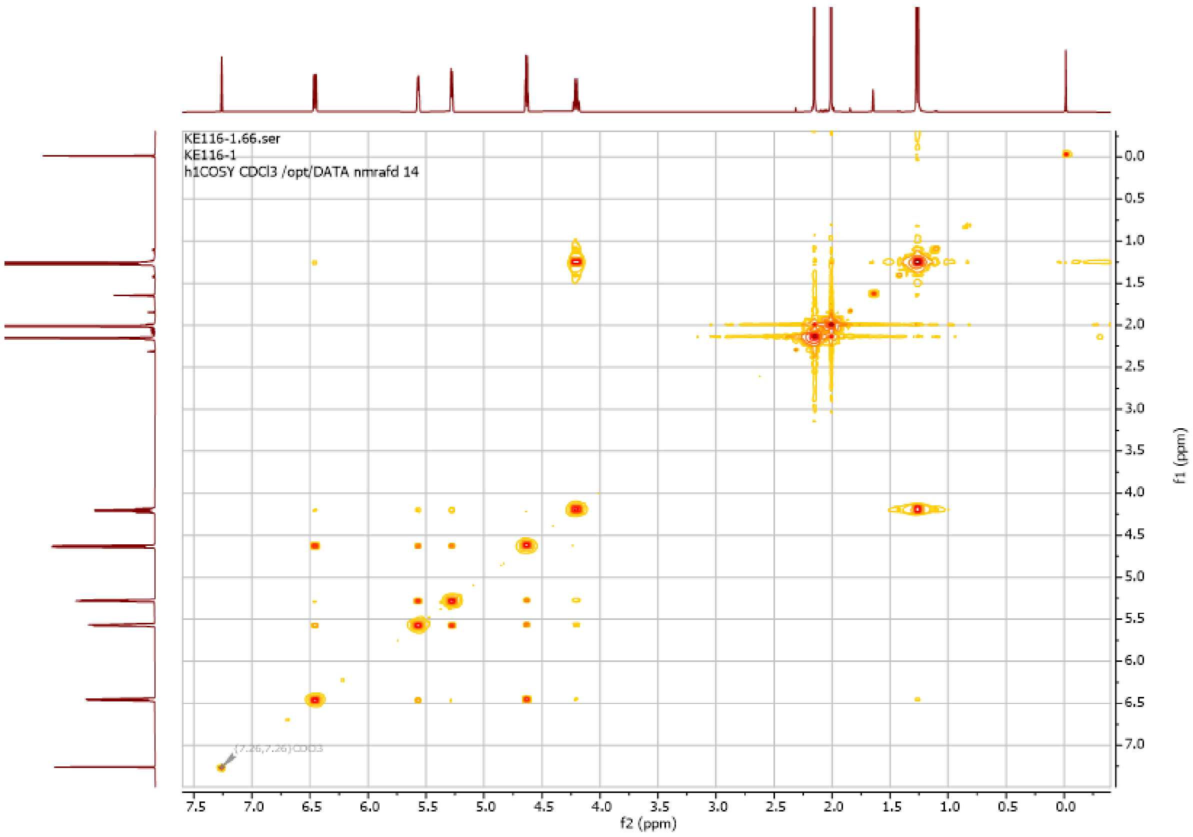

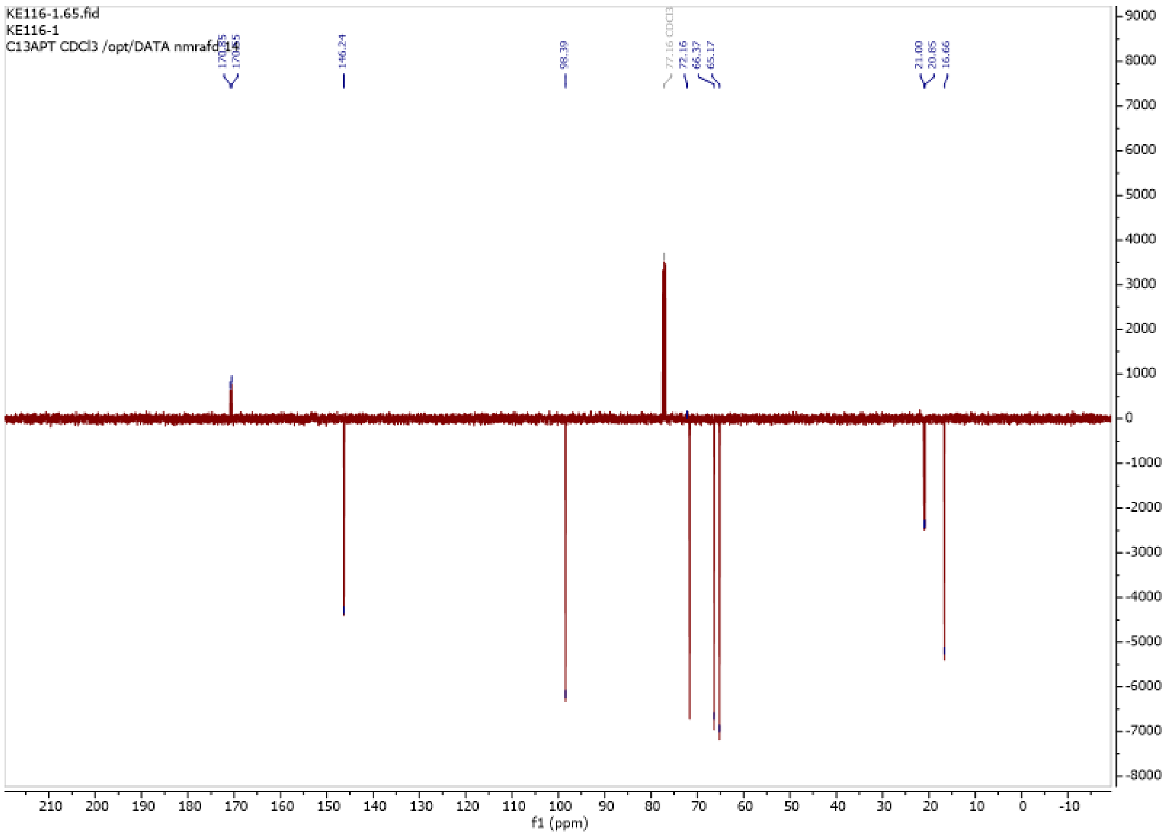
KE116-1.65.fid
KE116-1
C13APT CDCl3 /opt/DATA nmrafd 14
f1 (ppm)

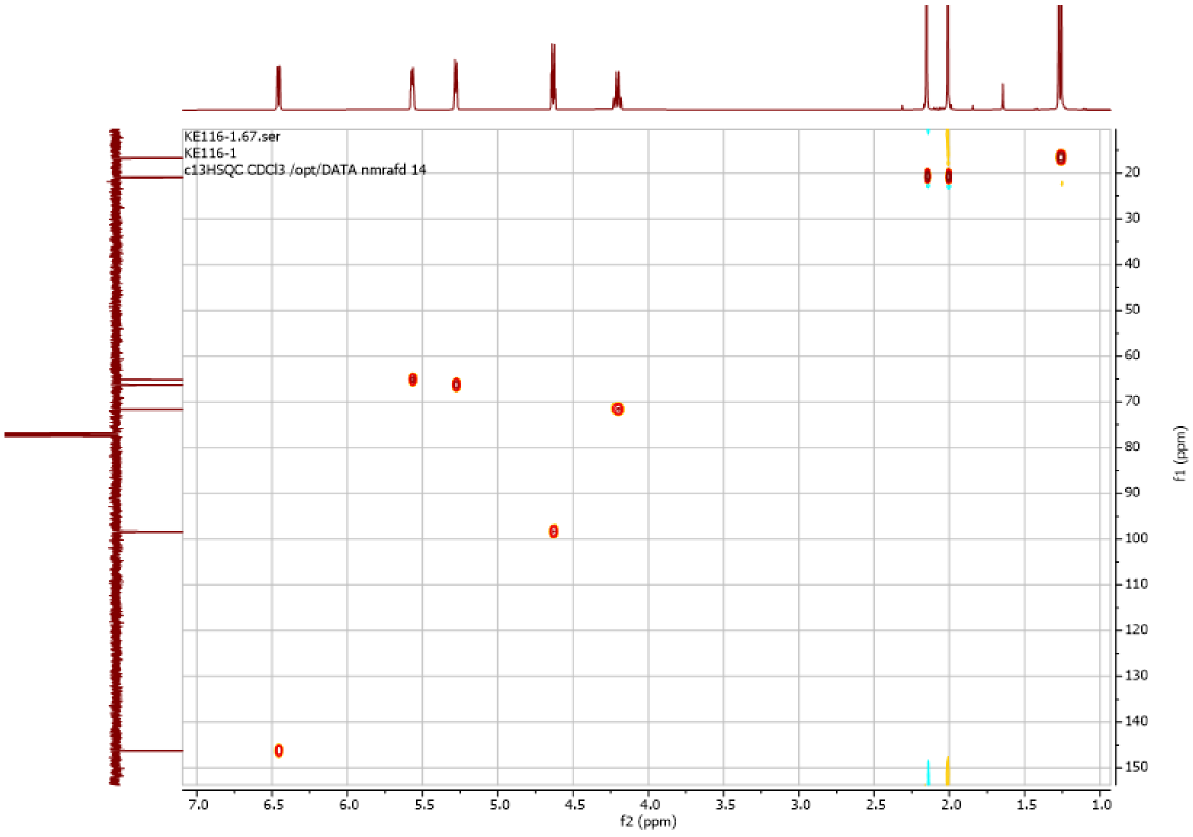
KE116-1.67.ser
KE116-1
c13HSQC CDCl3 /opt/DATA nmrafd 14
f2 (ppm)
f1 (ppm)

Phenyl 2-azido-2-deoxy-1-seleno-α-L-fucopyranoside (S9)

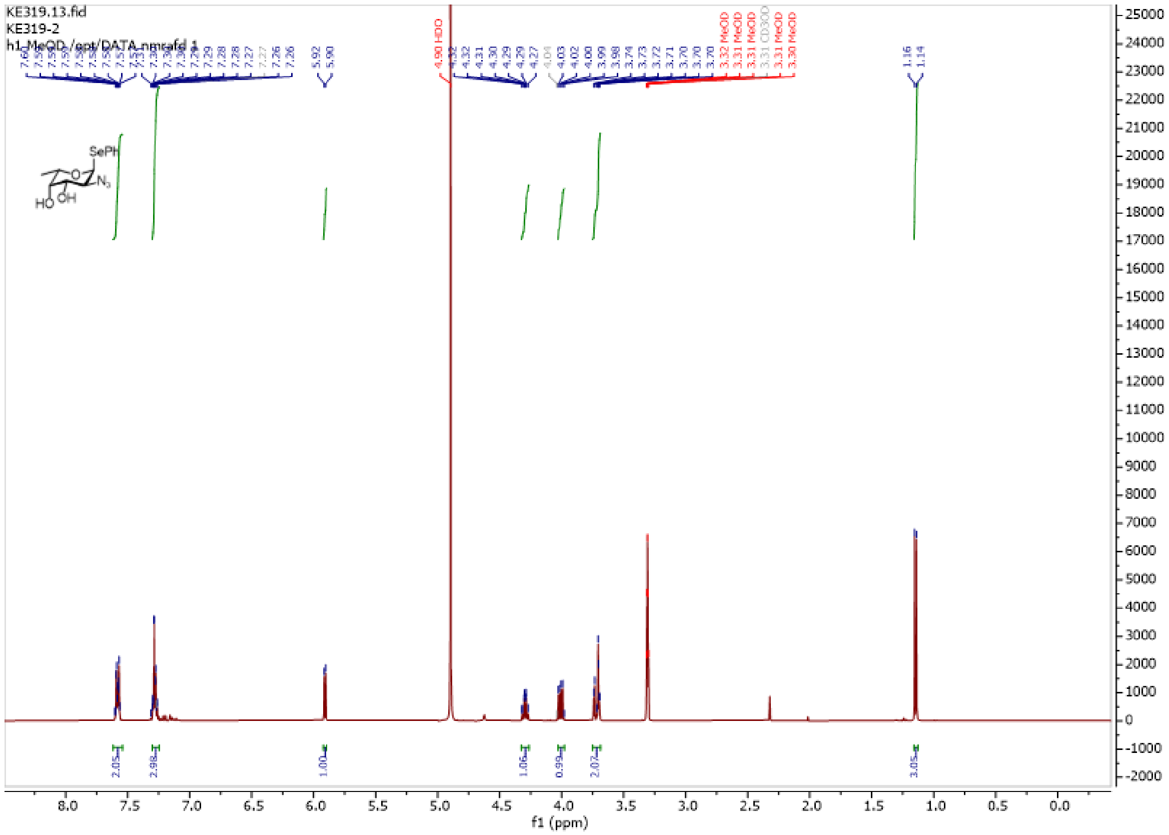

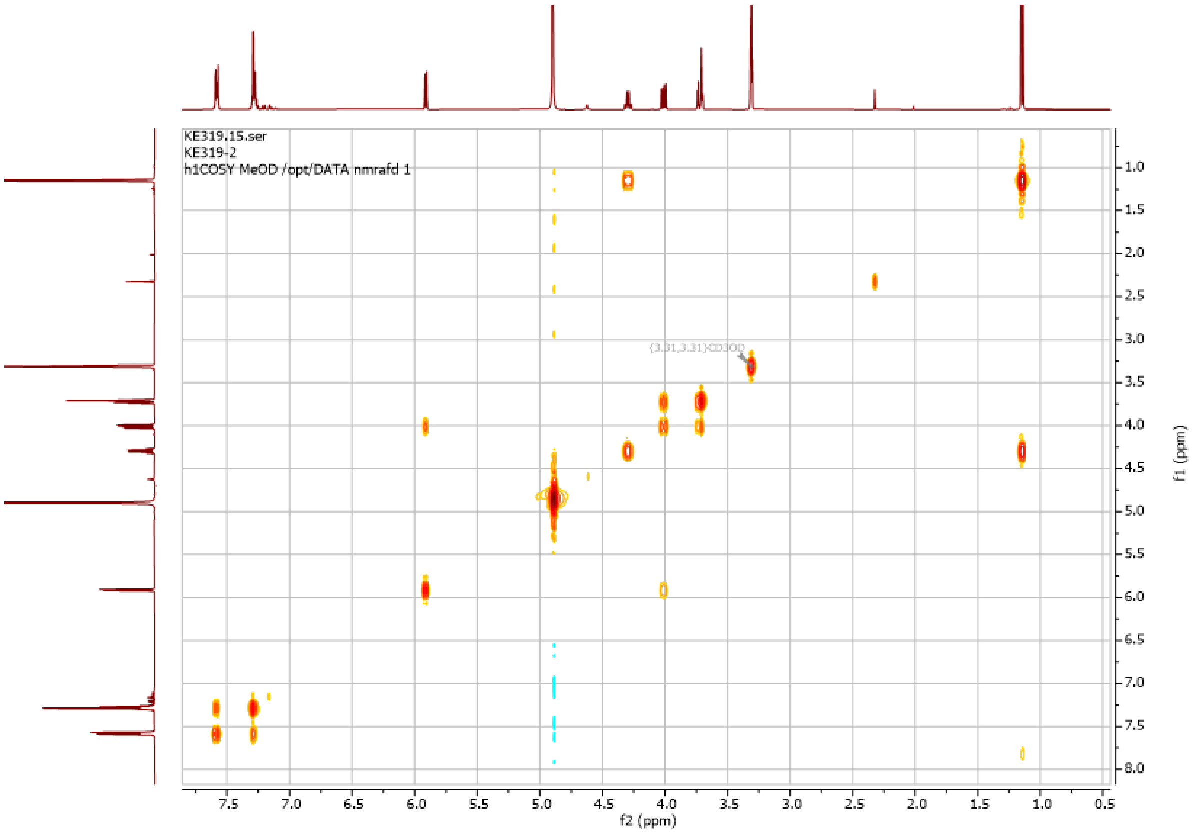

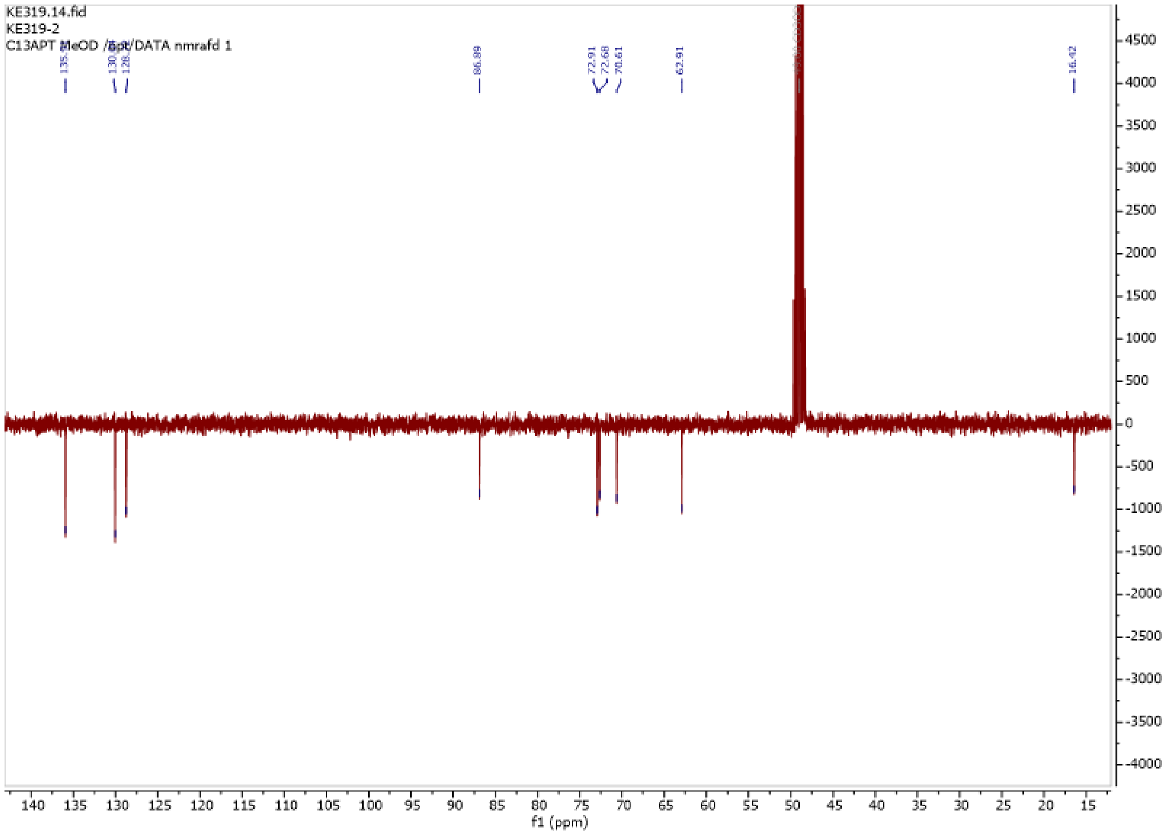
KE319.14.fid
KE319-2
C13APT MeOD /opt/DATA nmrafd 1
86.89
72.91
72.68
70.61
62.91
16.42
f1 (ppm)

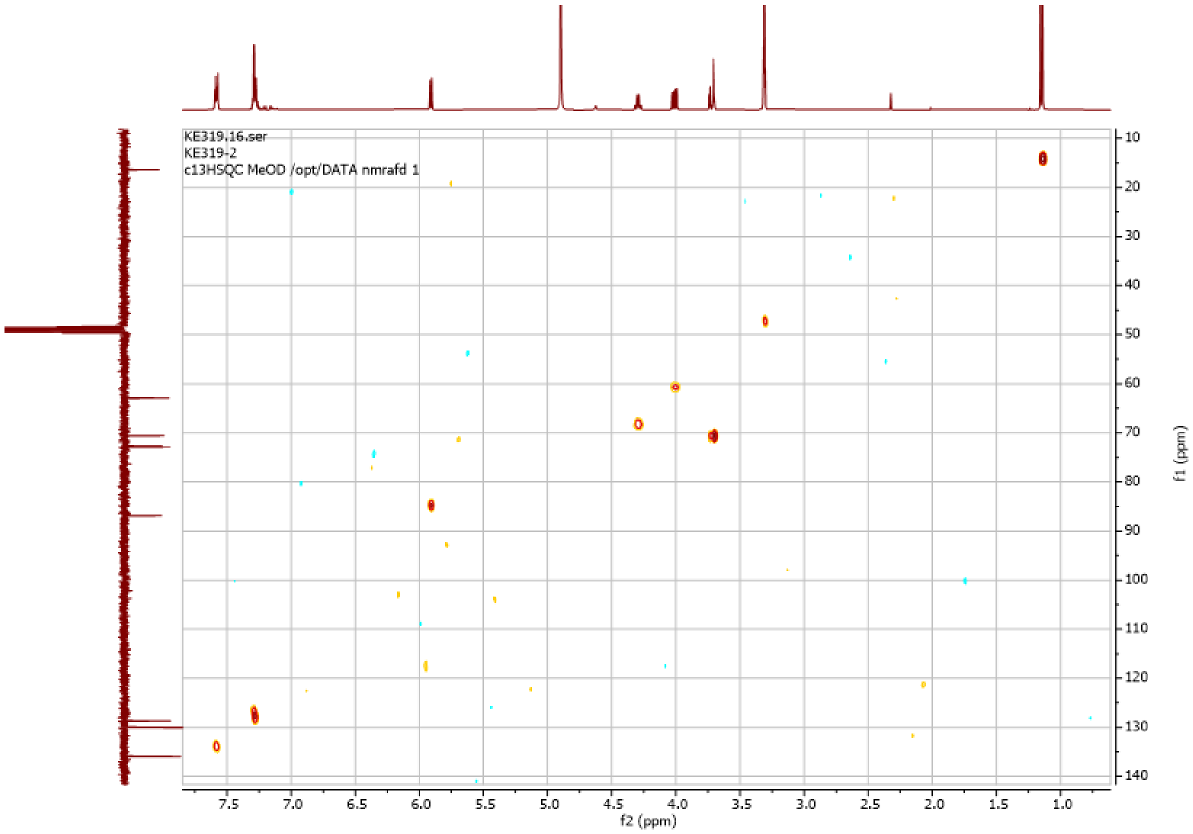
KE319.16.ser
KE319-2
c13HSQC MeOD /opt/DATA nmrafd 1
f2 (ppm)
f1 (ppm)

Phenyl 2-azido-2-deoxy-3-*O*-(2-naphthylmethyl)-1-seleno-*α*-L-fucopyranoside (S10)

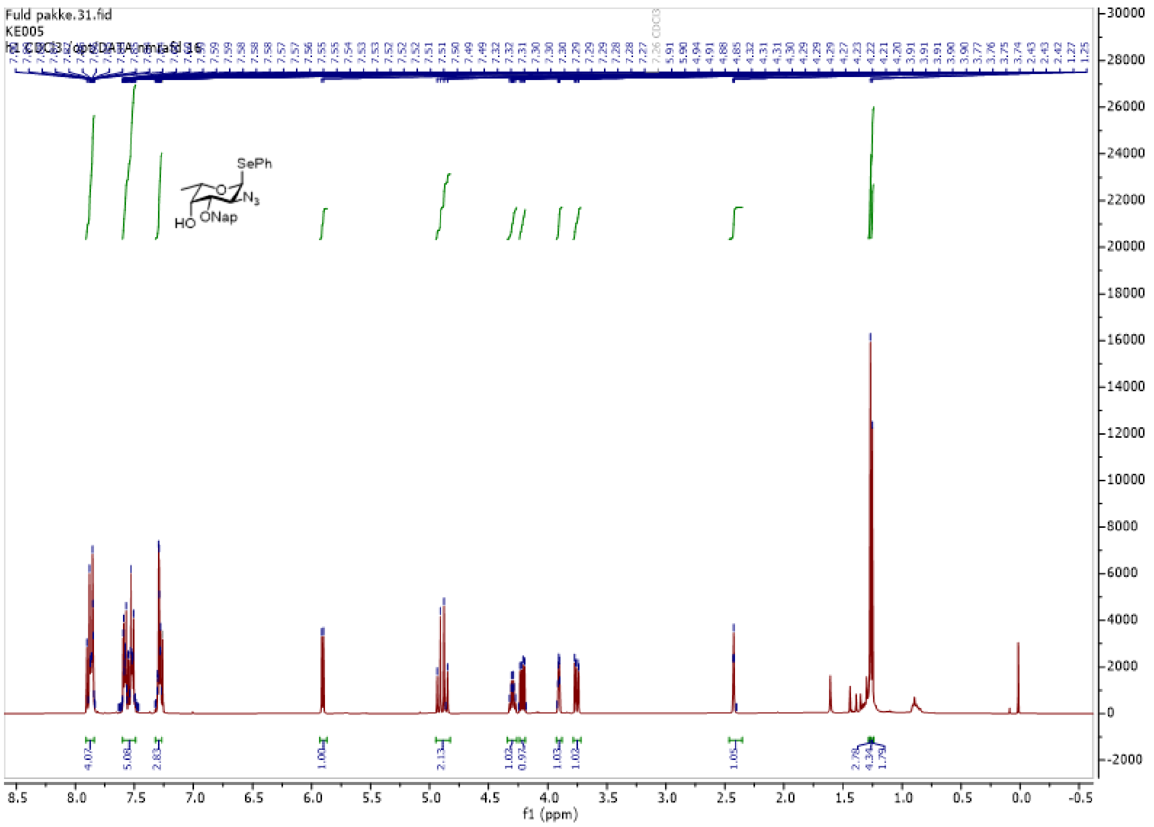

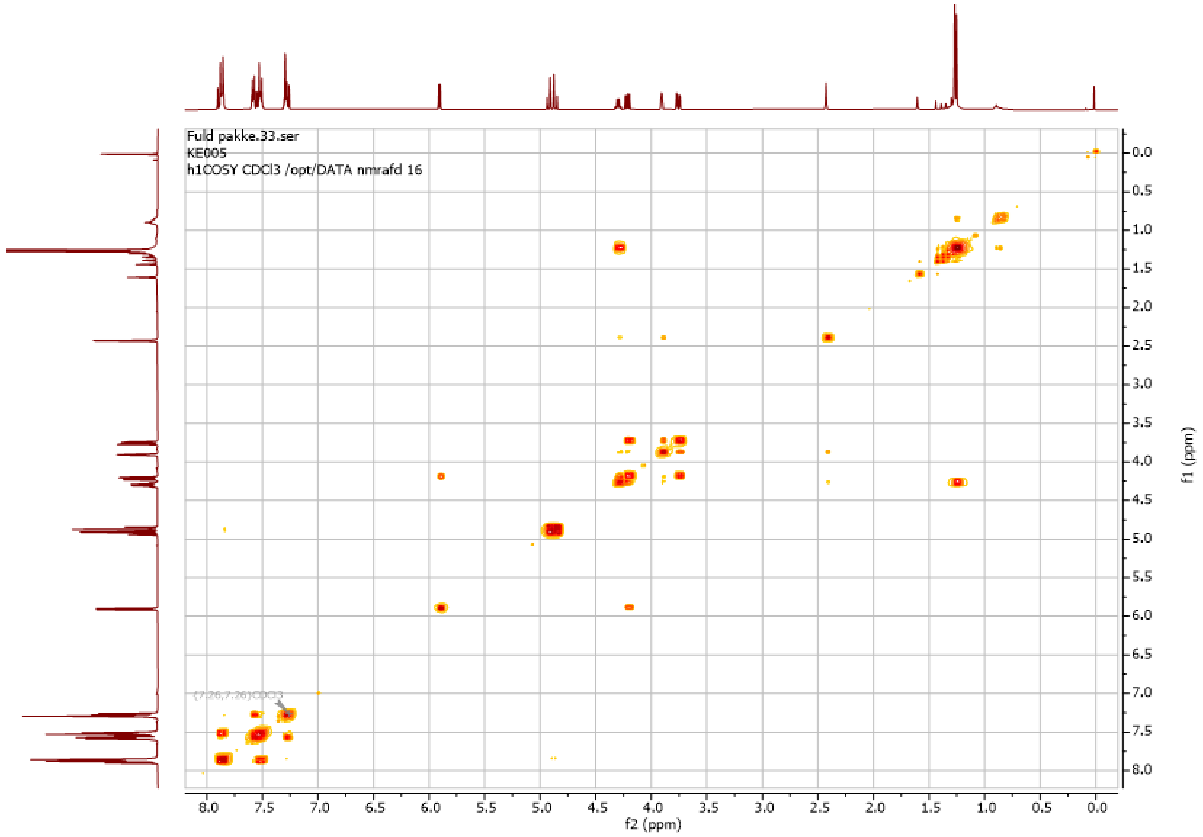

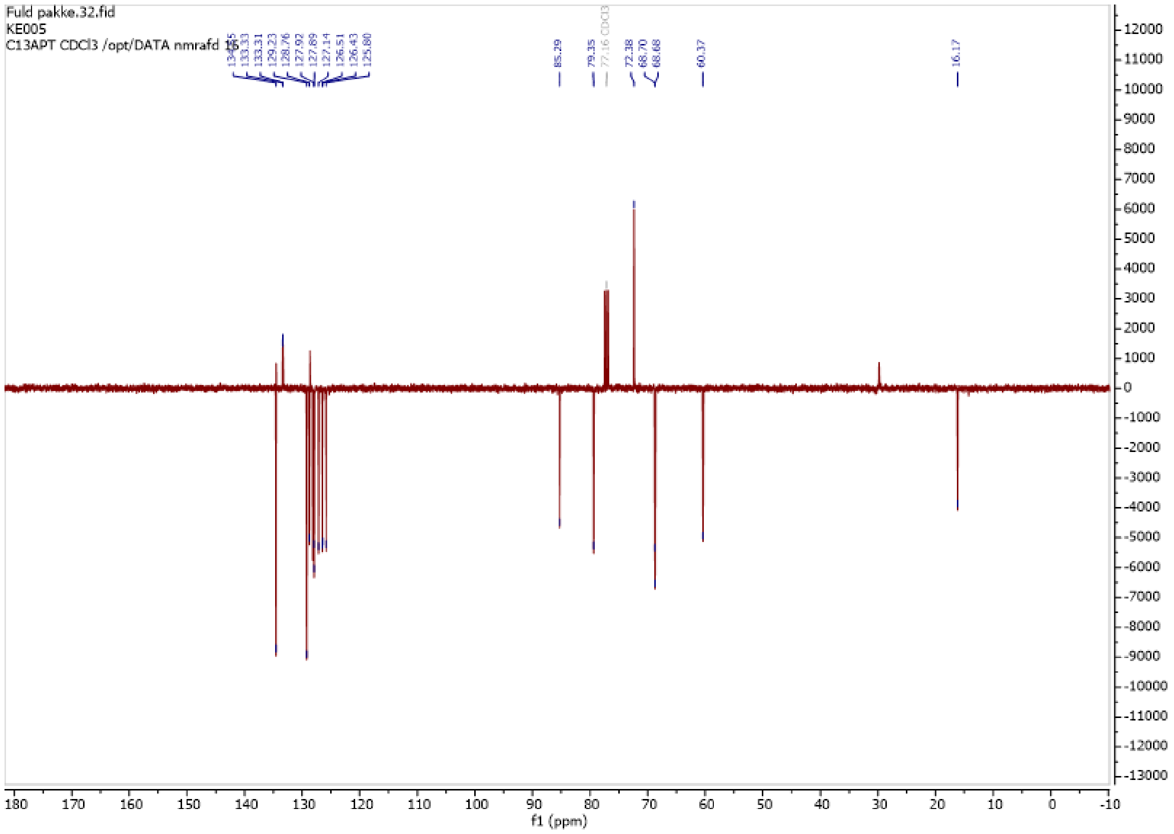
Fuld pakke.32.fid
KE005
C13APT CDCl3 /opt/DATA nmrafd 15
85.29
79.35
77.16 CDCl3
72.38
68.70
68.68
60.37
16.17
f1 (ppm)

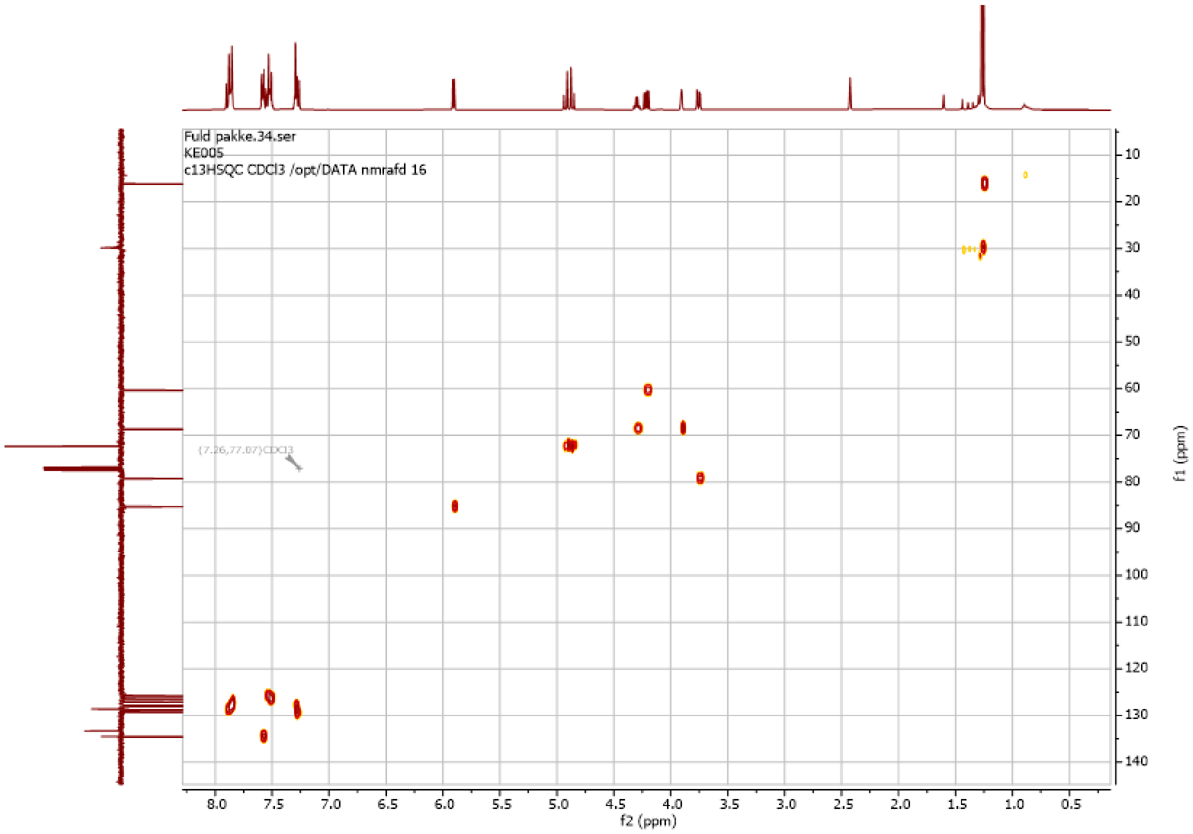
Fuld pakke.34.ser
KE005
c13HSQC CDCl3 /opt/DATA nmrafd 16
(7.26,77.07)CDCl3
f2 (ppm)
f1 (ppm)

Phenyl 2-azido-4-*O*-benzyl-2-deoxy-3-*O*-(2-naphthylmethyl)-L-fucopyranoside (11a)

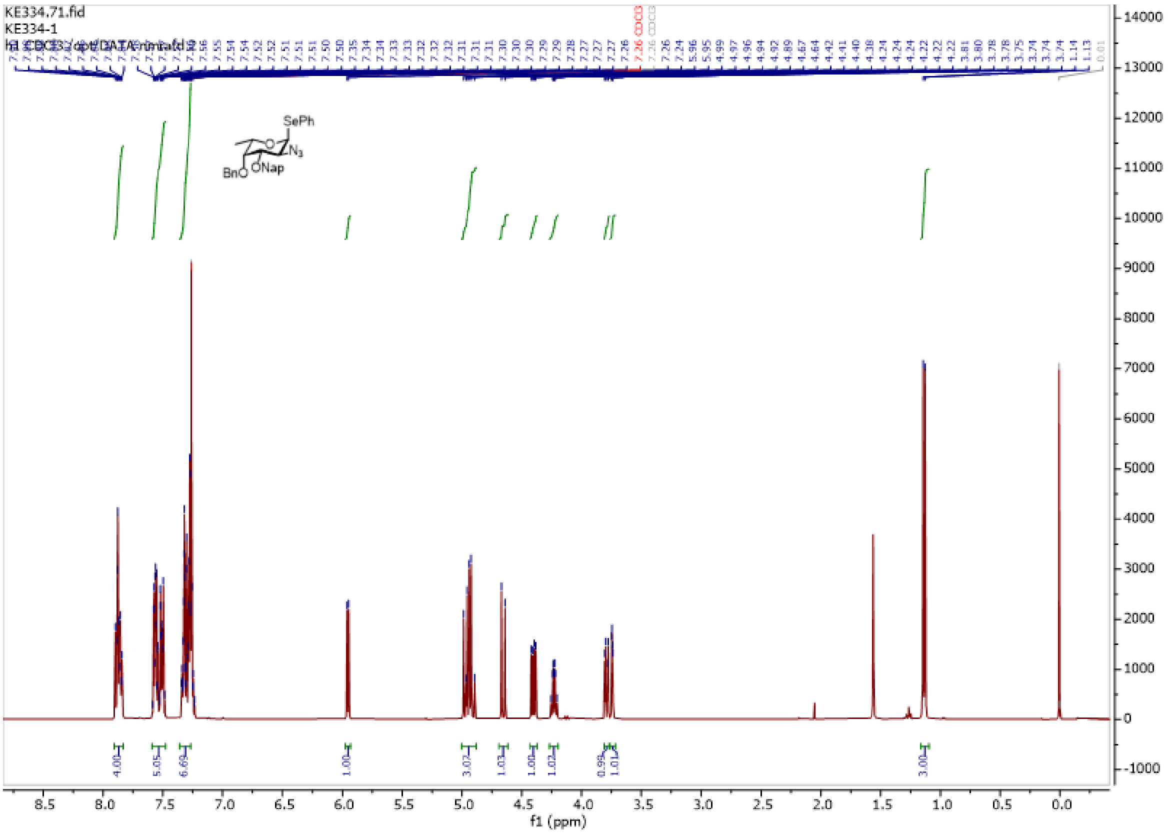

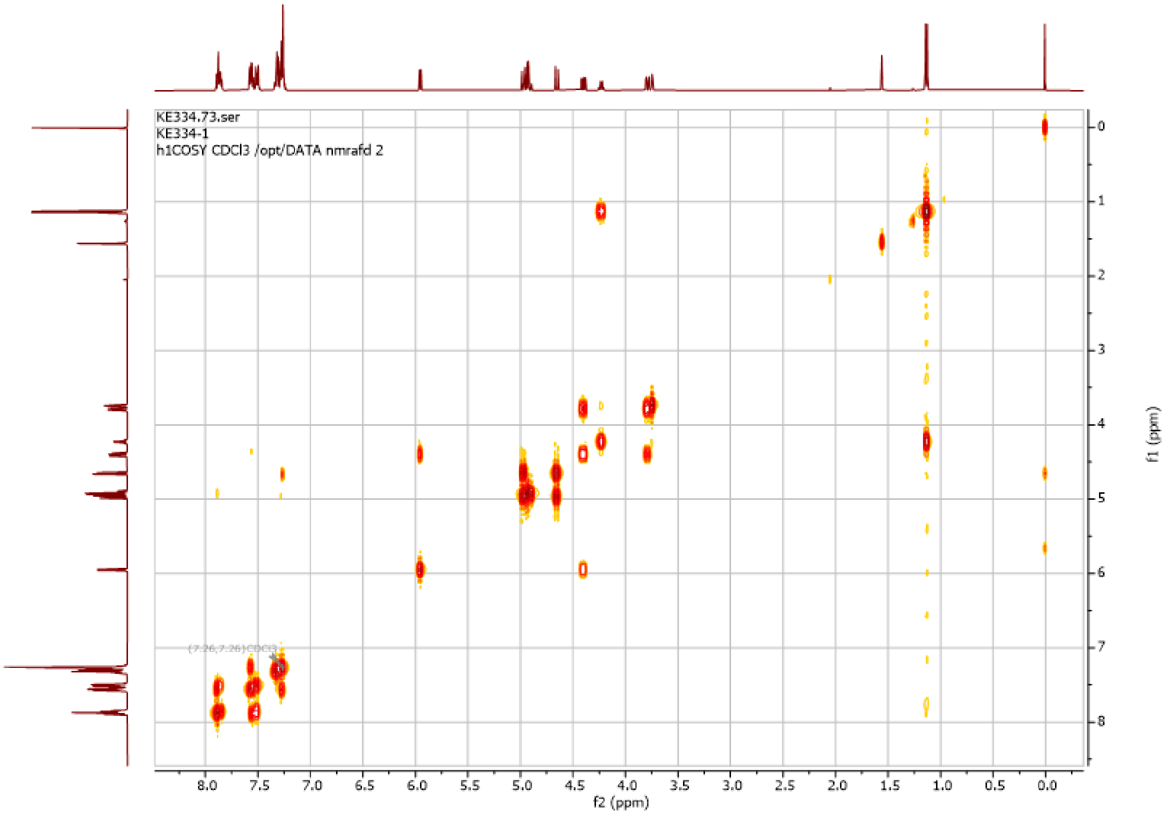

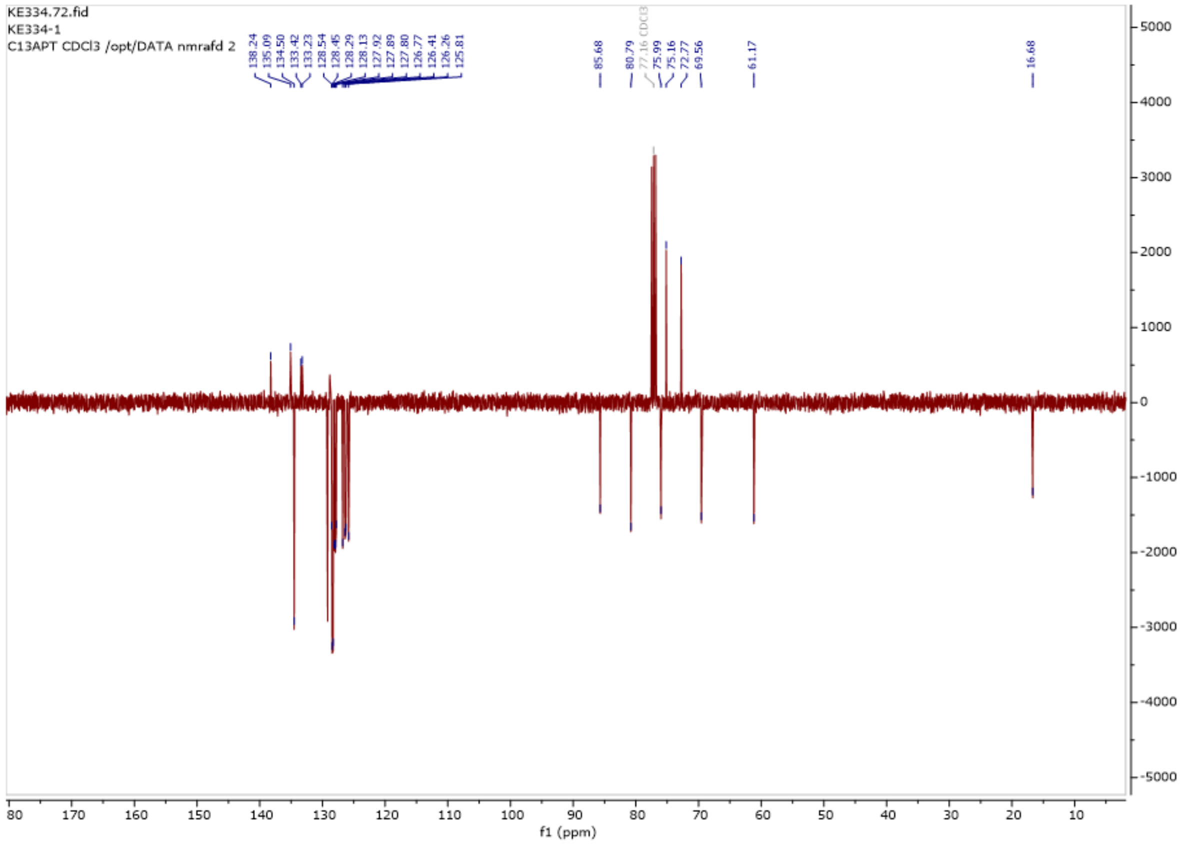
KE334.72.fid
KE334-1
C13APT CDCl3 /opt/DATA nmrafd 2
138.24
135.09
134.50
133.42
133.23
128.54
128.45
128.29
128.13
127.92
127.89
127.80
126.77
126.41
126.26
125.81
85.68
80.79
77.16 CDCl3
75.99
75.16
72.77
69.56
61.17
16.68
f1 (ppm)

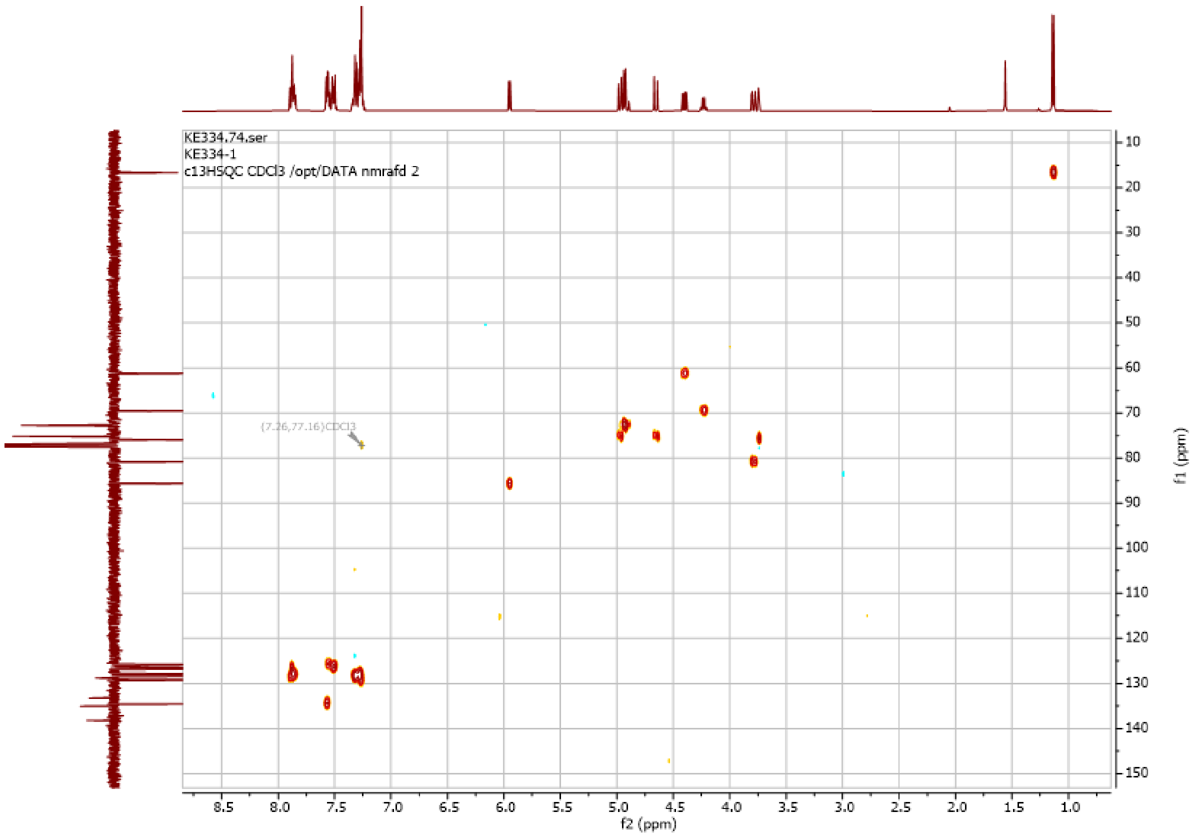
KE334.74.ser
KE334-1
c13HSQC CDCl3 /opt/DATA nmrafd 2
{7.26,77.16}CDCl3
f2 (ppm)
f1 (ppm)

2-azido-4-*O*-benzyl-2-deoxy-3-*O*-(2-naphthylmethyl)-*α*/*β*-L-fucopyranose (S11)

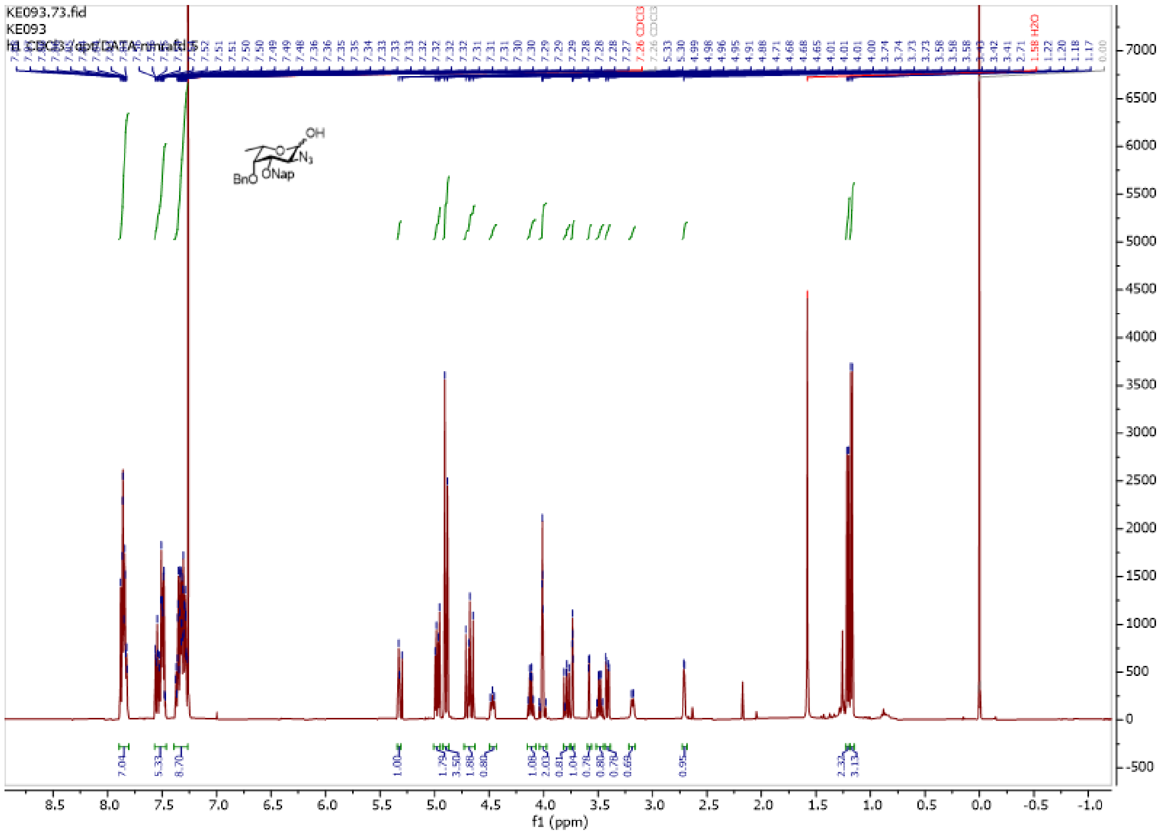

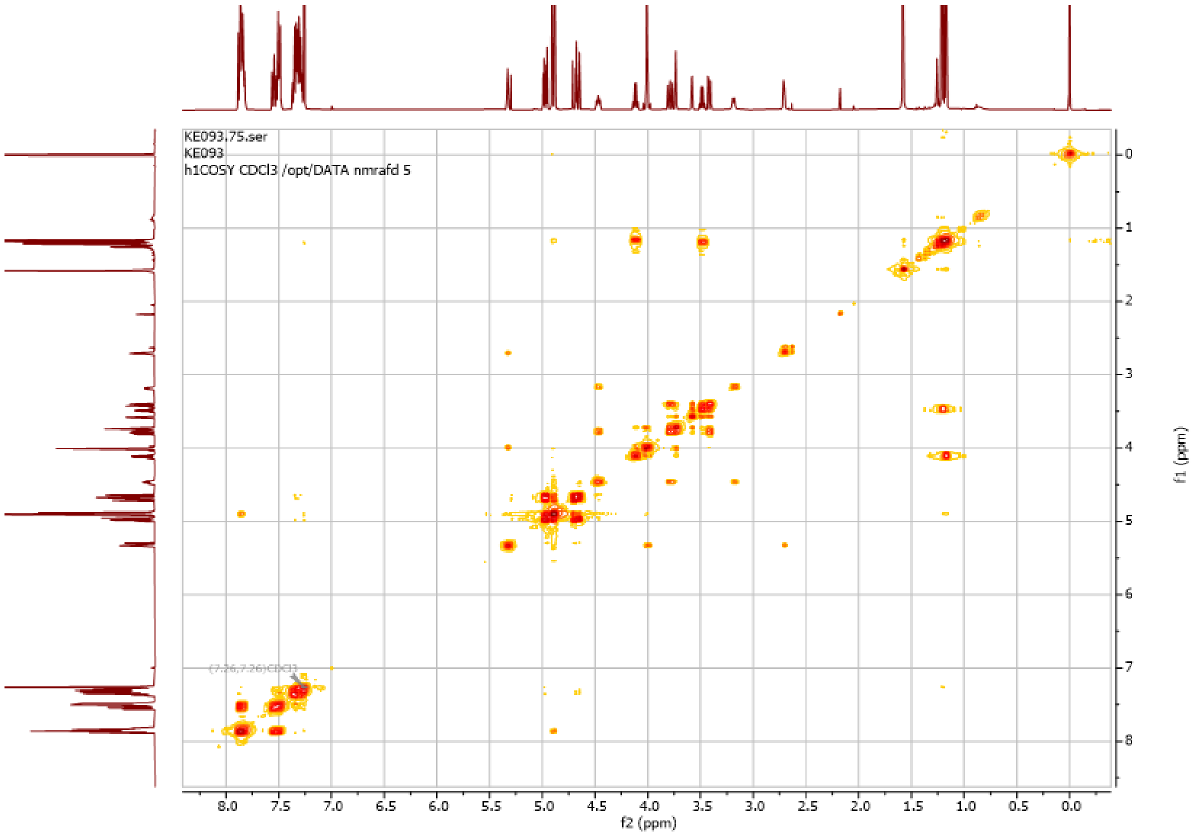

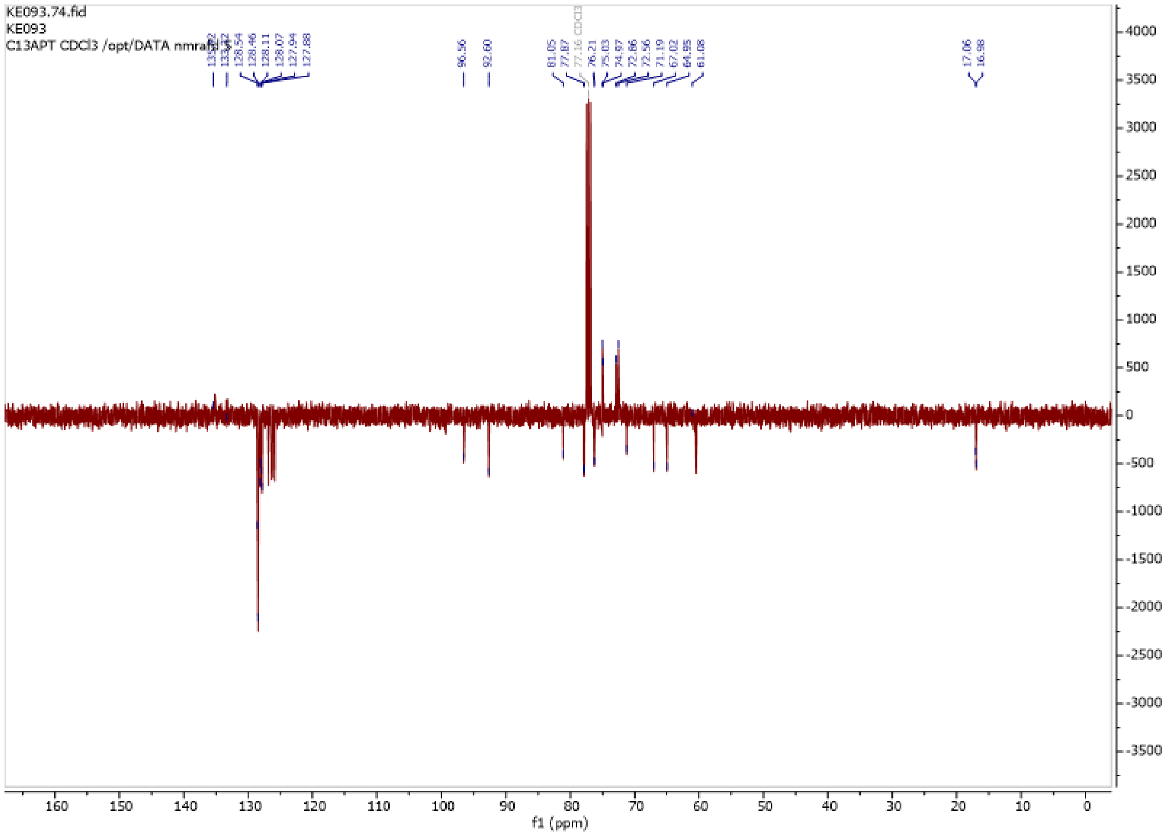
KE093.74.fid
KE093
C13APT CDCl3 /opt/DATA nmrafd 5
f1 (ppm)

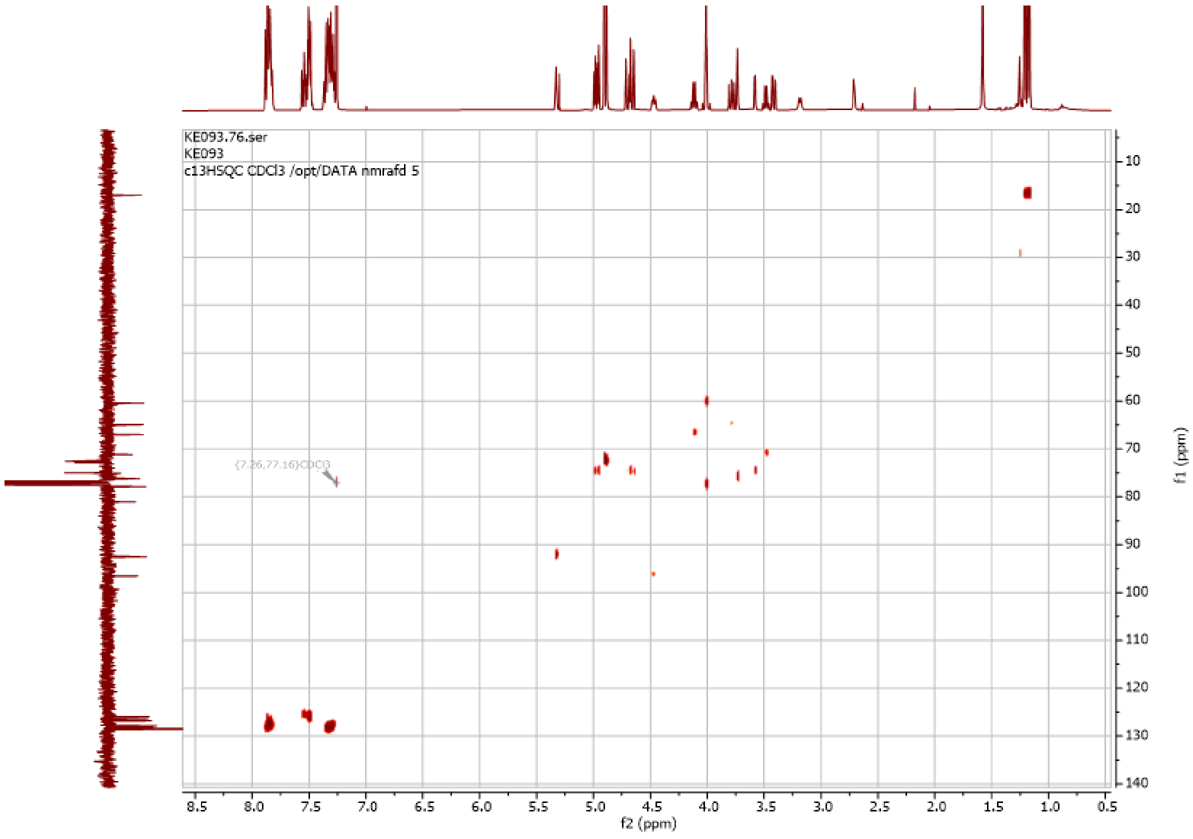
KE093.76.ser
KE093
c13HSQC CDCl3 /opt/DATA nmrafd 5
f2 (ppm)
f1 (ppm)

2-azido-4-*O*-benzyl-2-deoxy-3-*O*-(2-naphthylmethyl)-1-*O*-(*N*-phenyl-2,2,2-trifluoroacetimidoyl)-*α*/*β*-L-fucopyranose (11b)

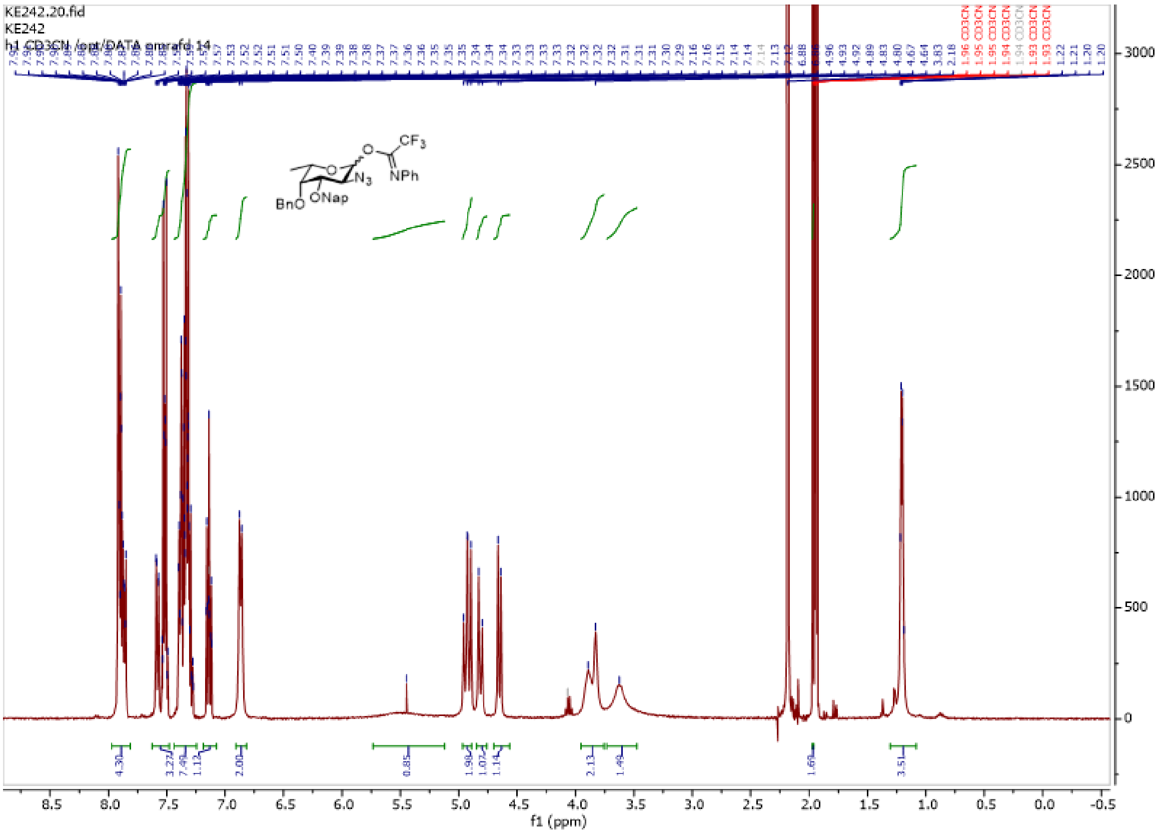

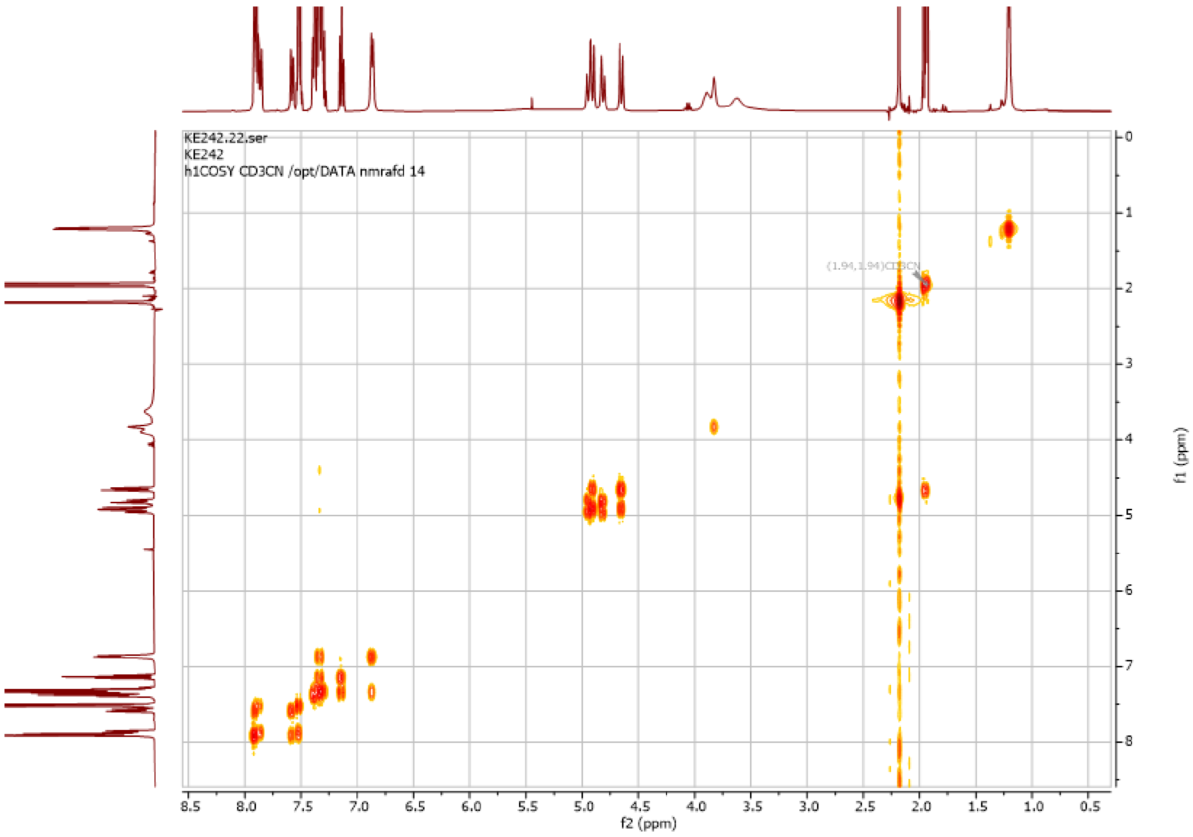

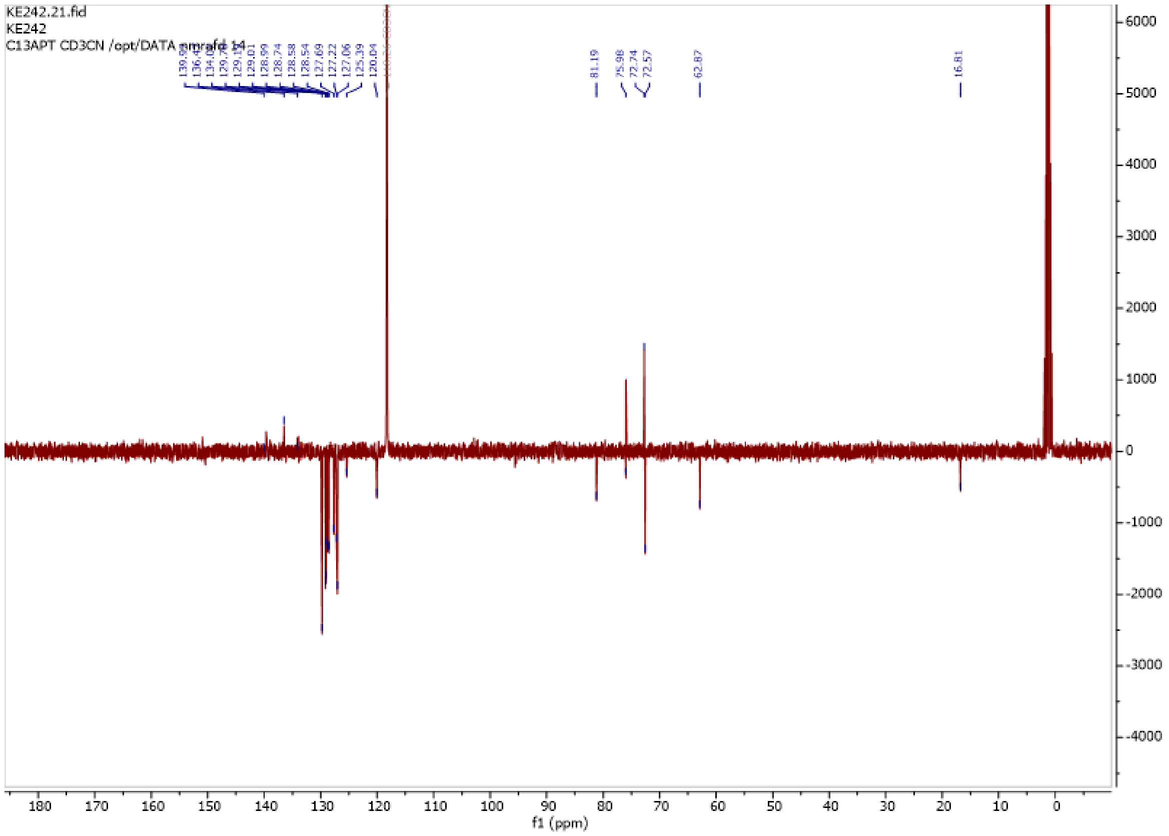
KE242.21.fid
KE242
C13APT CD3CN /opt/DATA nmrafd 14
129.01
128.99
128.74
128.58
128.54
127.69
127.22
127.06
125.39
120.04
81.19
75.98
72.74
72.57
62.87
16.81
f1 (ppm)

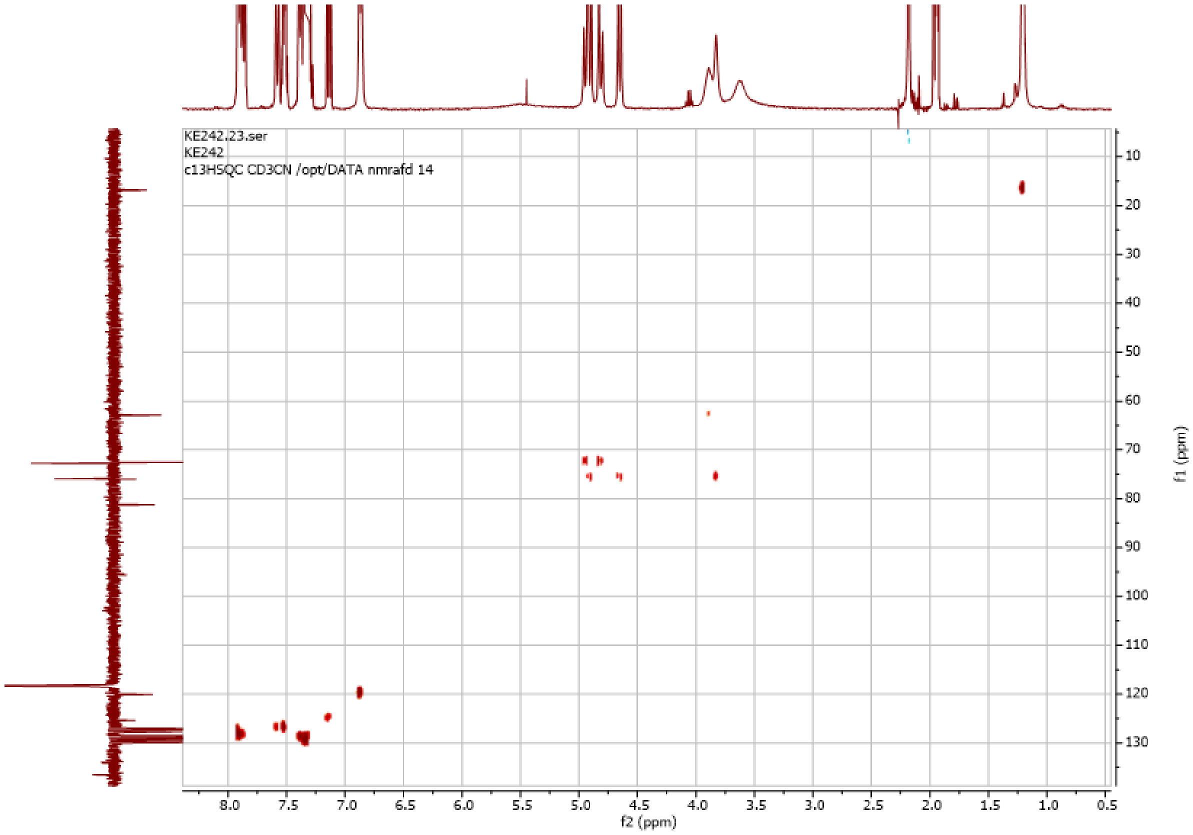
KE242.23.ser
KE242
c13HSQC CD3CN /opt/DATA nmrafd 14
f2 (ppm)
f1 (ppm)

1,3,4,6 Tetra-*O*-acetyl-*α*/*β*-D-mannopyranose (S12)

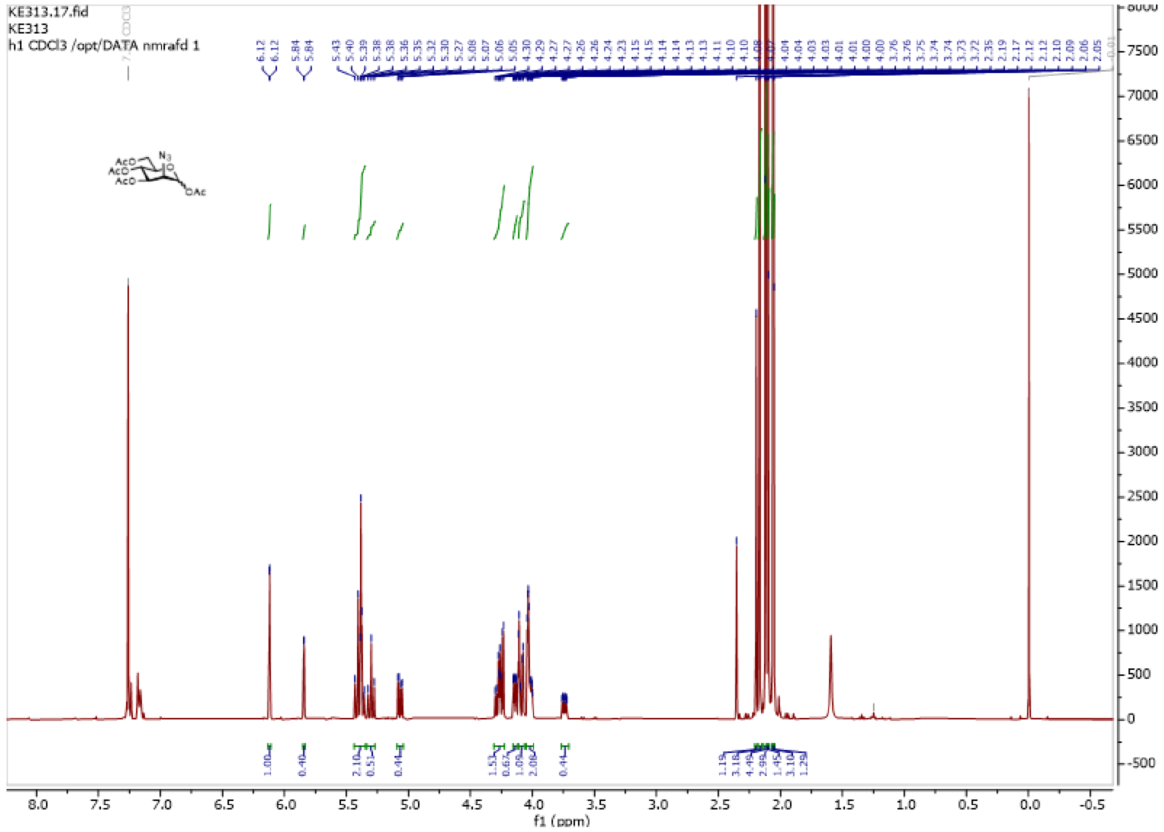

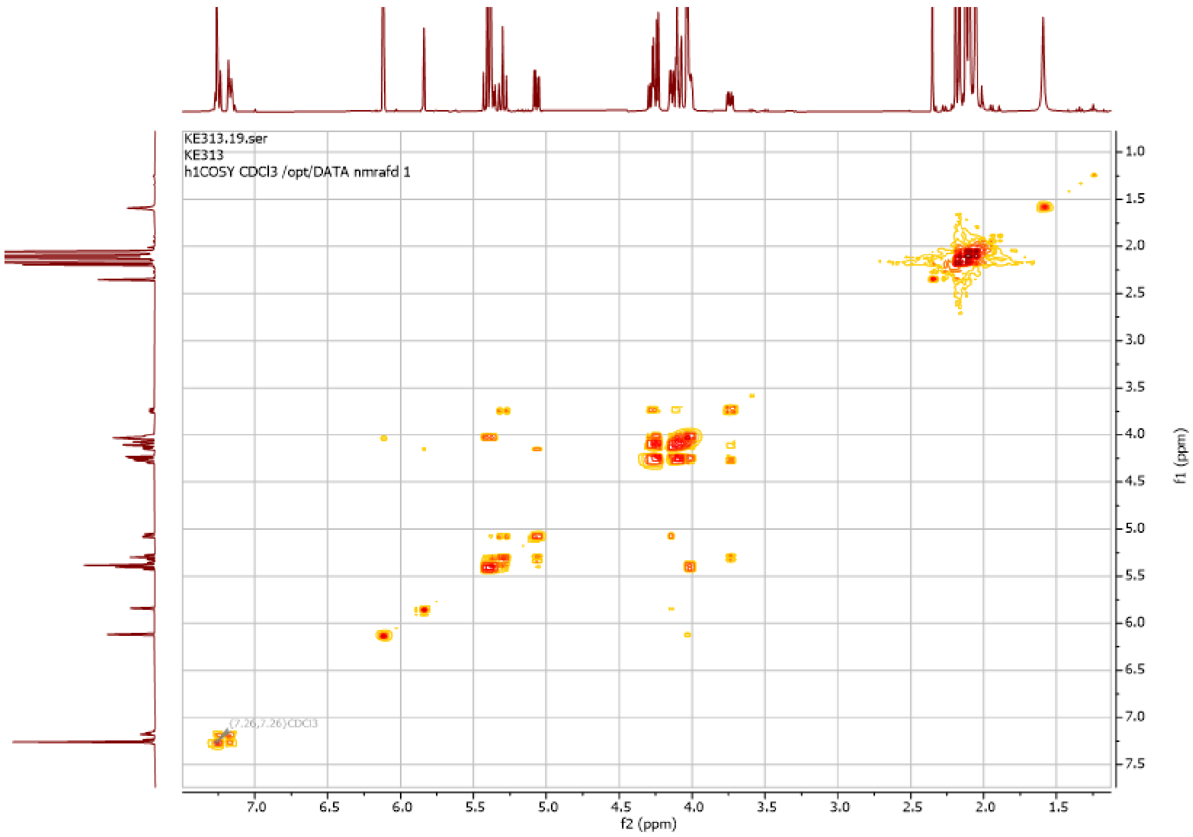

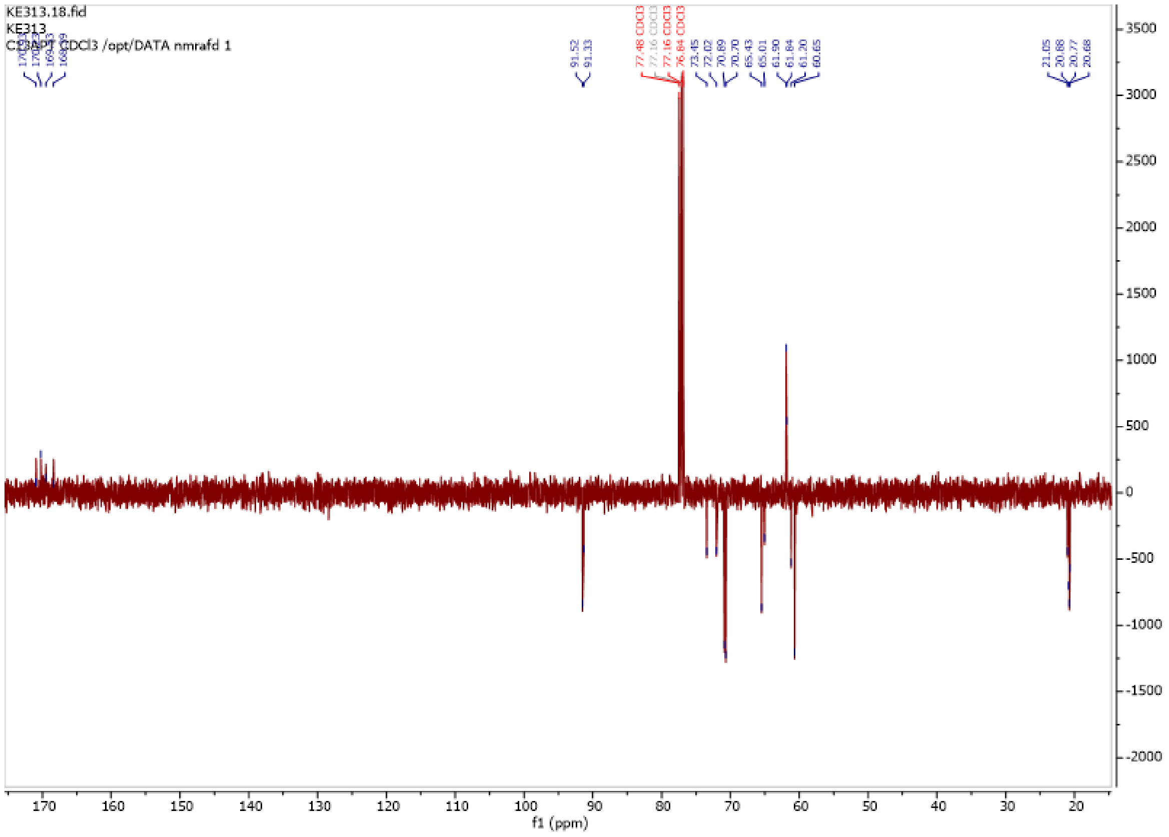
KE313.18.fid
KE313
C13APT CDCl3 /opt/DATA nmrafd 1
91.52
91.33
77.48 CDCl3
77.16 CDCl3
77.16 CDCl3
76.84 CDCl3
73.45
72.02
70.89
70.70
65.43
65.01
61.90
61.84
61.20
60.65
21.05
20.88
20.77
20.68
f1 (ppm)

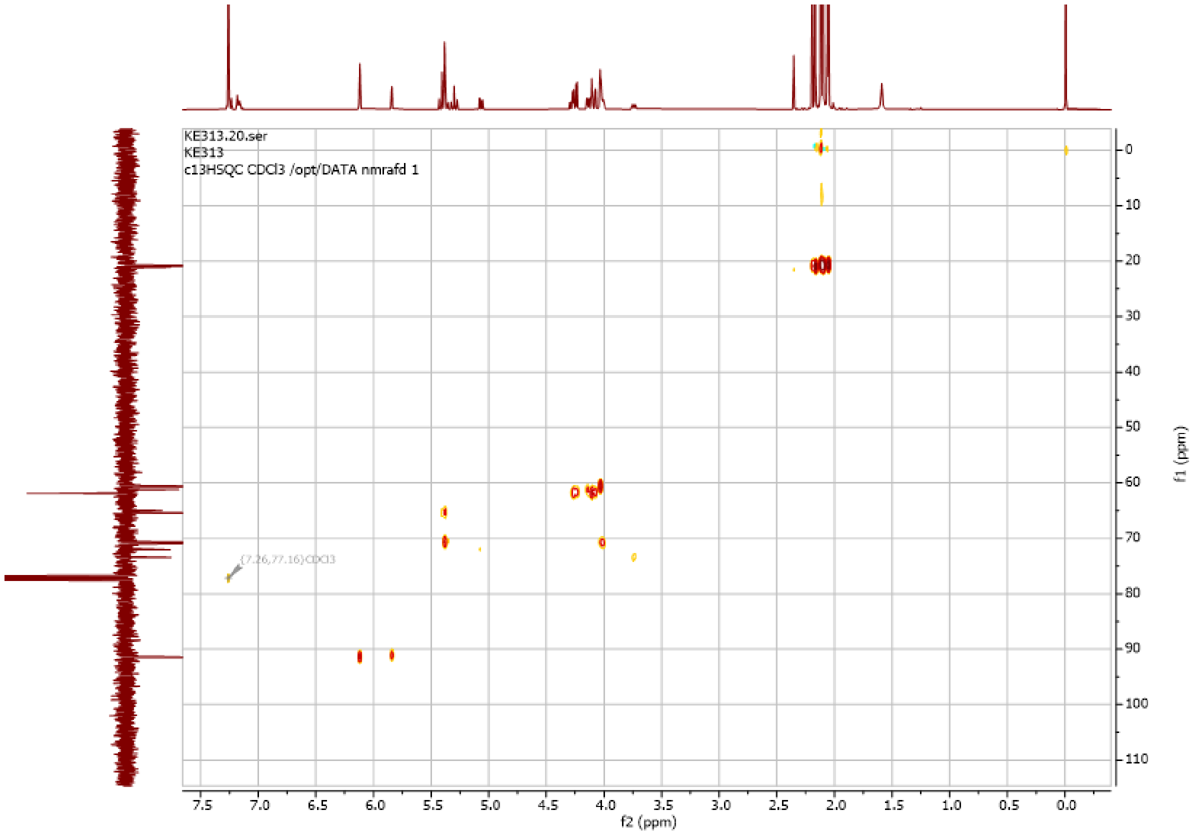
KE313.20.ser
KE313
c13HSQC CDCl3 /opt/DATA nmrafd 1
(7.26,77.16)CDCl3
f2 (ppm)
f1 (ppm)

Phenyl 3,4,6 tri-*O*-acetyl-2-azido-2-deoxy-1-thio-*α*/*β*-D-mannopyranoside (S13)

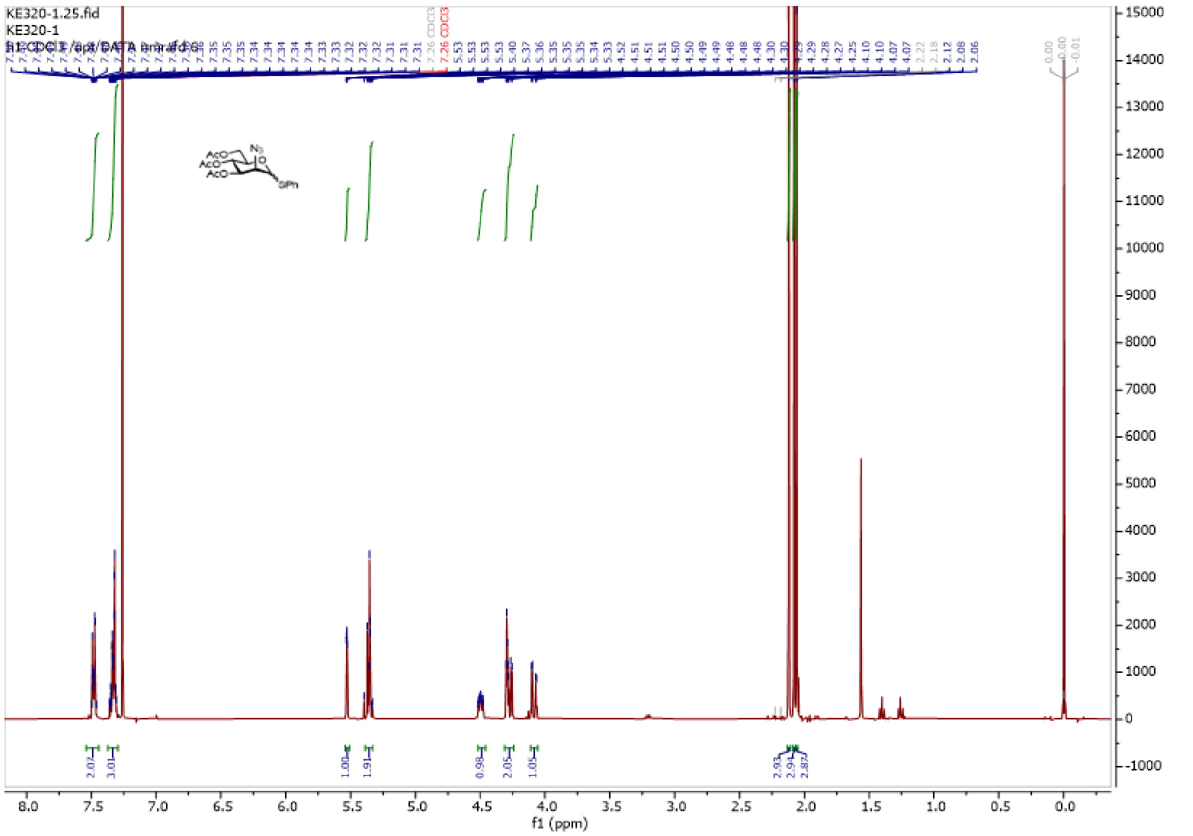

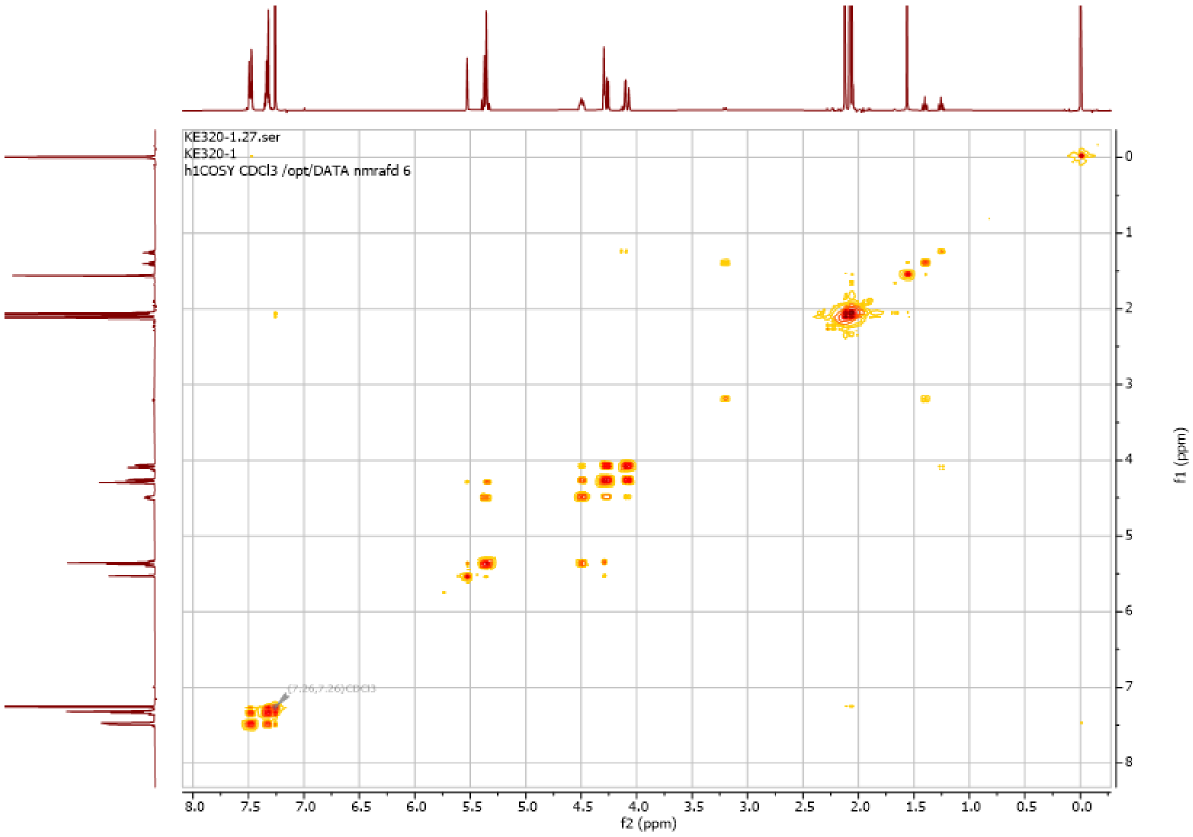

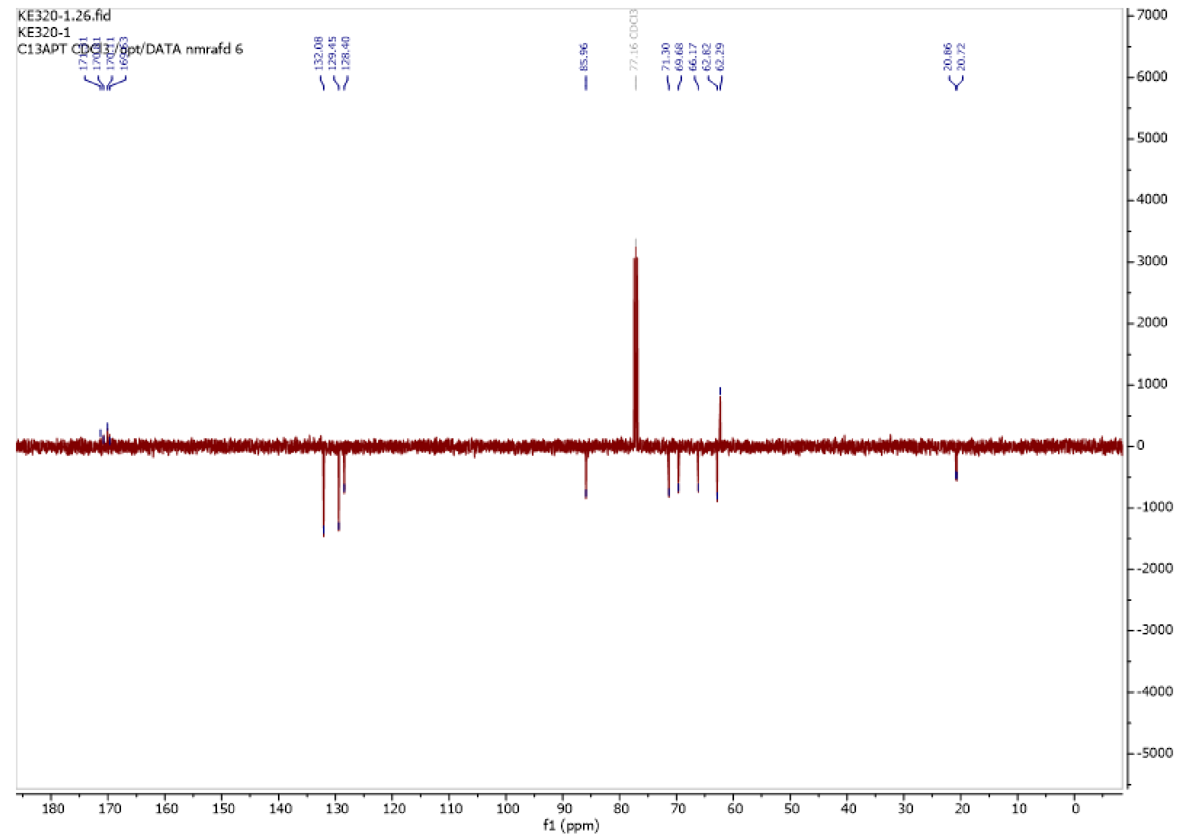
KE320-1.26.fid
KE320-1
C13APT CDCl3 /opt/DATA nmrafd 6
132.08
129.45
128.40
85.96
77.16 CDCl3
71.30
69.68
66.17
62.82
62.29
20.86
20.72
f1 (ppm)

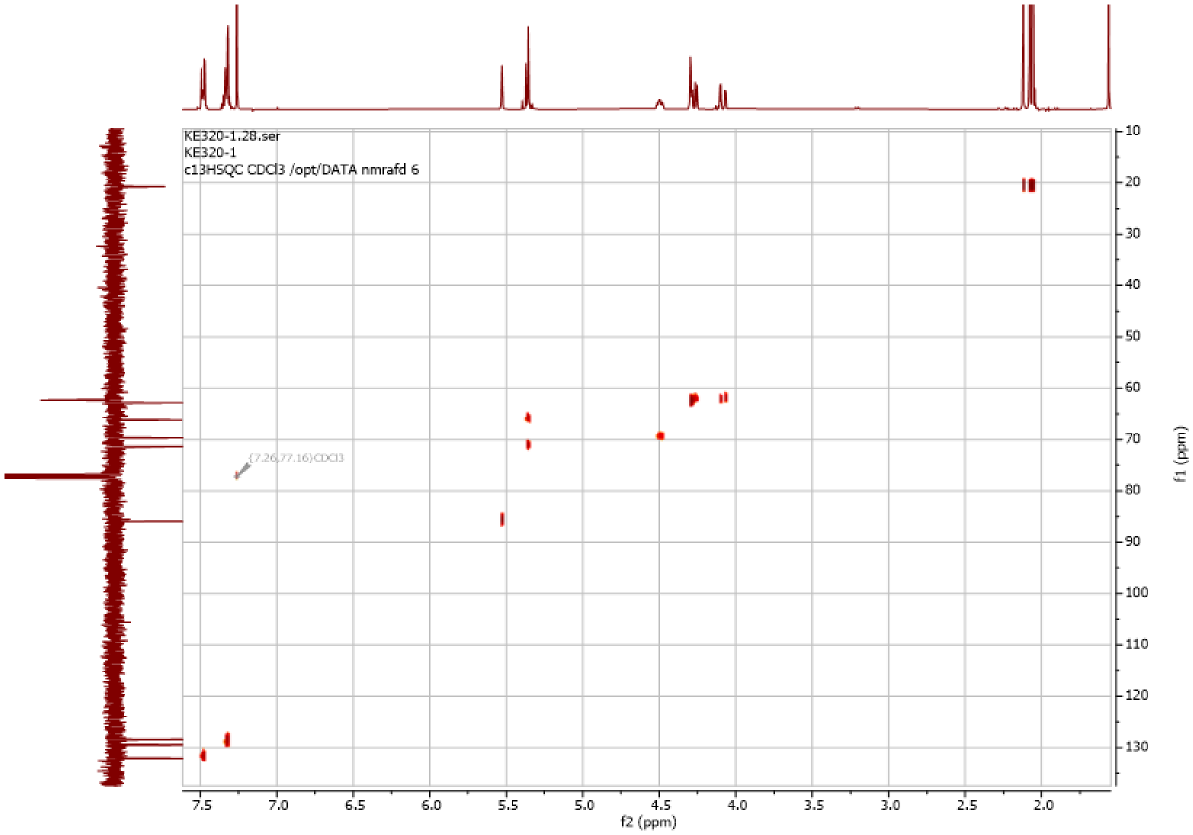
KE320-1.28.ser
KE320-1
c13HSQC CDCl3 /opt/DATA nmrafd 6
(7.26,77.16)CDCl3
f2 (ppm)
f1 (ppm)

Phenyl 2-azido-2-deoxy-1-thio-*α*-D-mannopyranoside (S14)

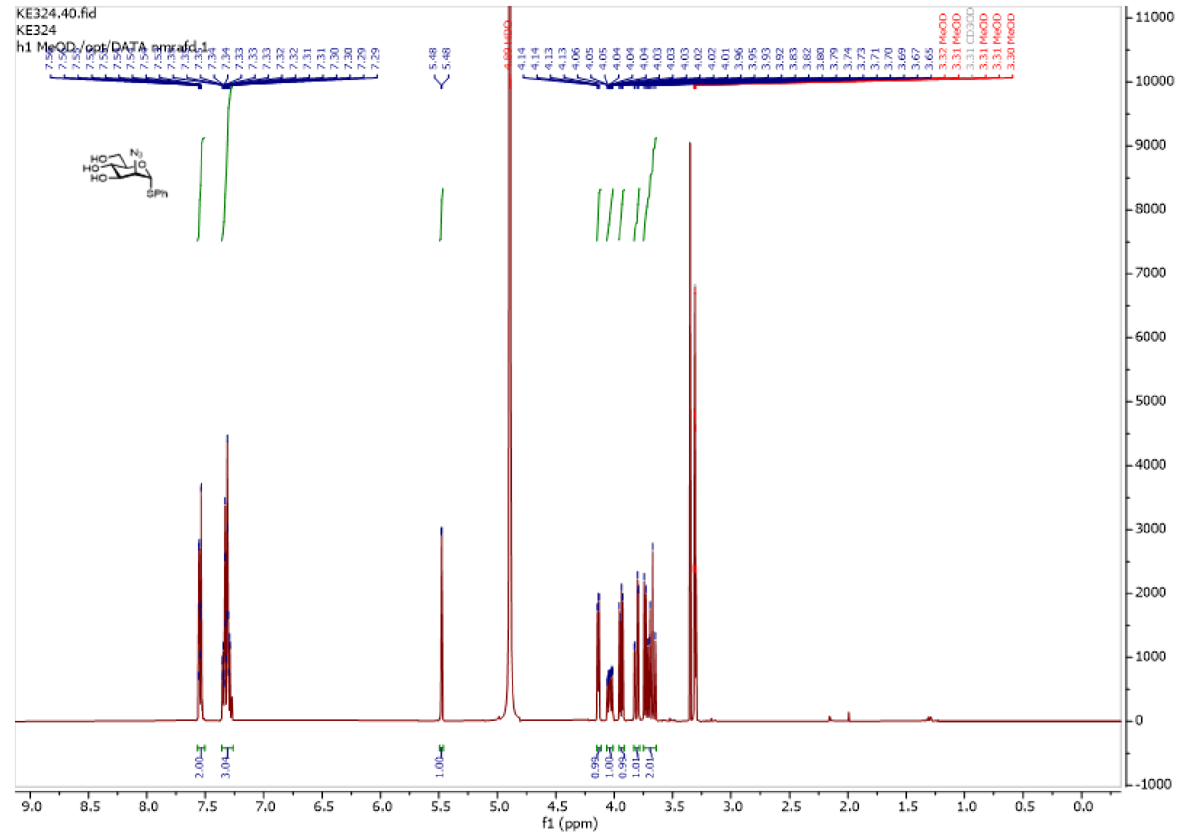

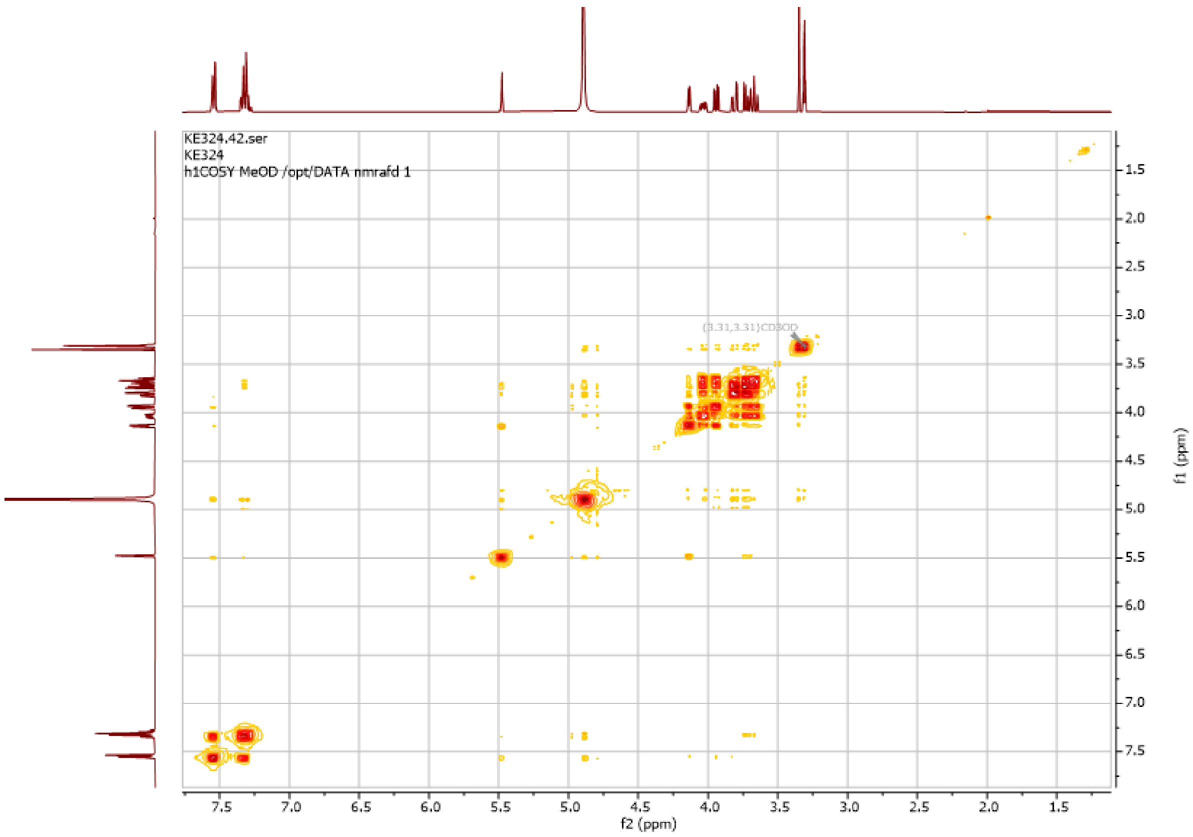

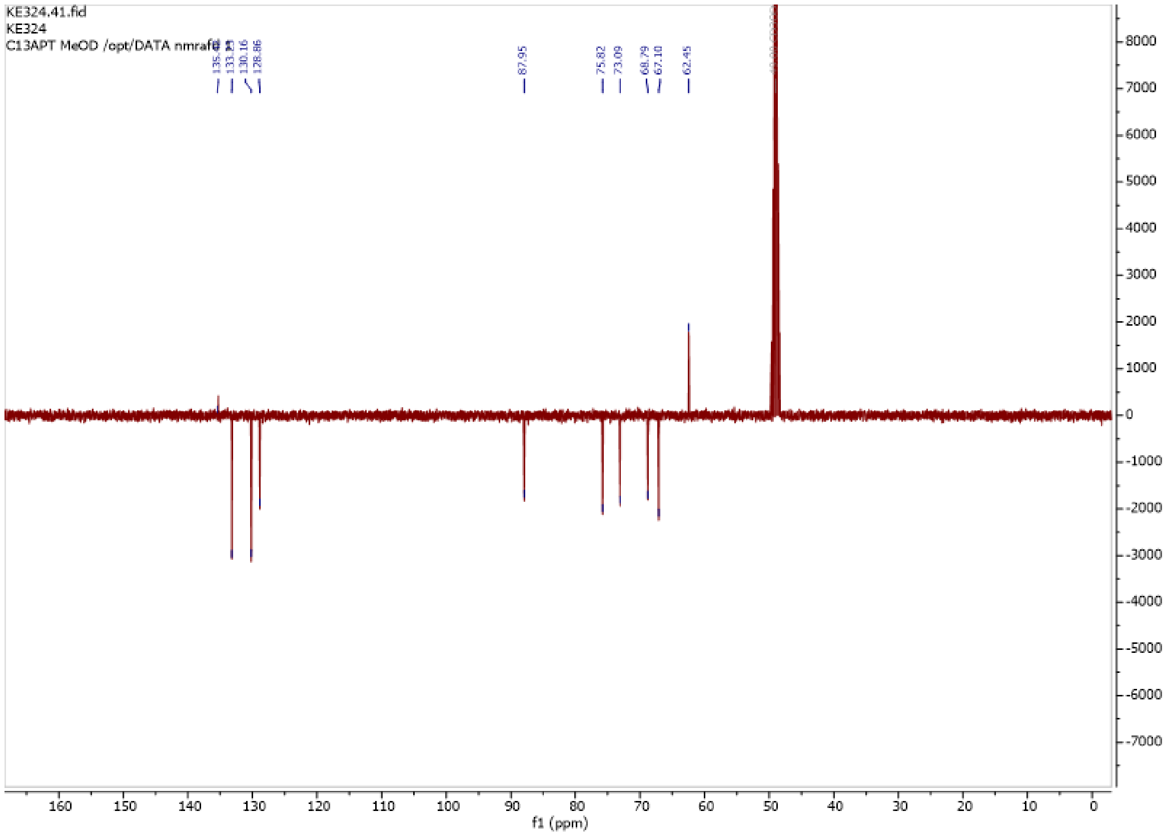
KE324.41.fid
KE324
C13APT MeOD /opt/DATA nmrafd 1
87.95
75.82
73.09
68.79
67.10
62.45
f1 (ppm)

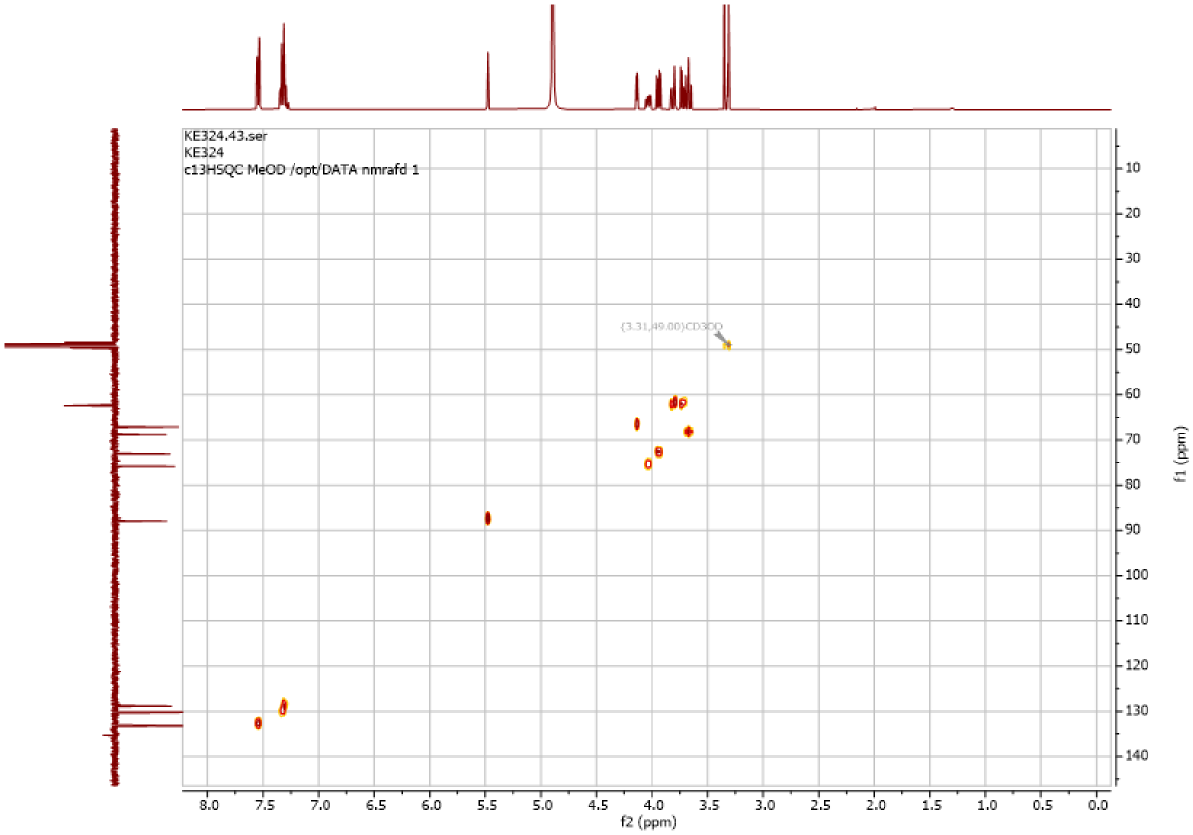
KE324.43.ser
KE324
c13HSQC MeOD /opt/DATA nmrafd 1
(3.31,49.00)CD3OD
f2 (ppm)
f1 (ppm)

Phenyl 2-azido-2-deoxy-4,6-*O*-(*p*-methoxybenzylidene)-1-thio-*α*-D-mannopyranoside (S15)

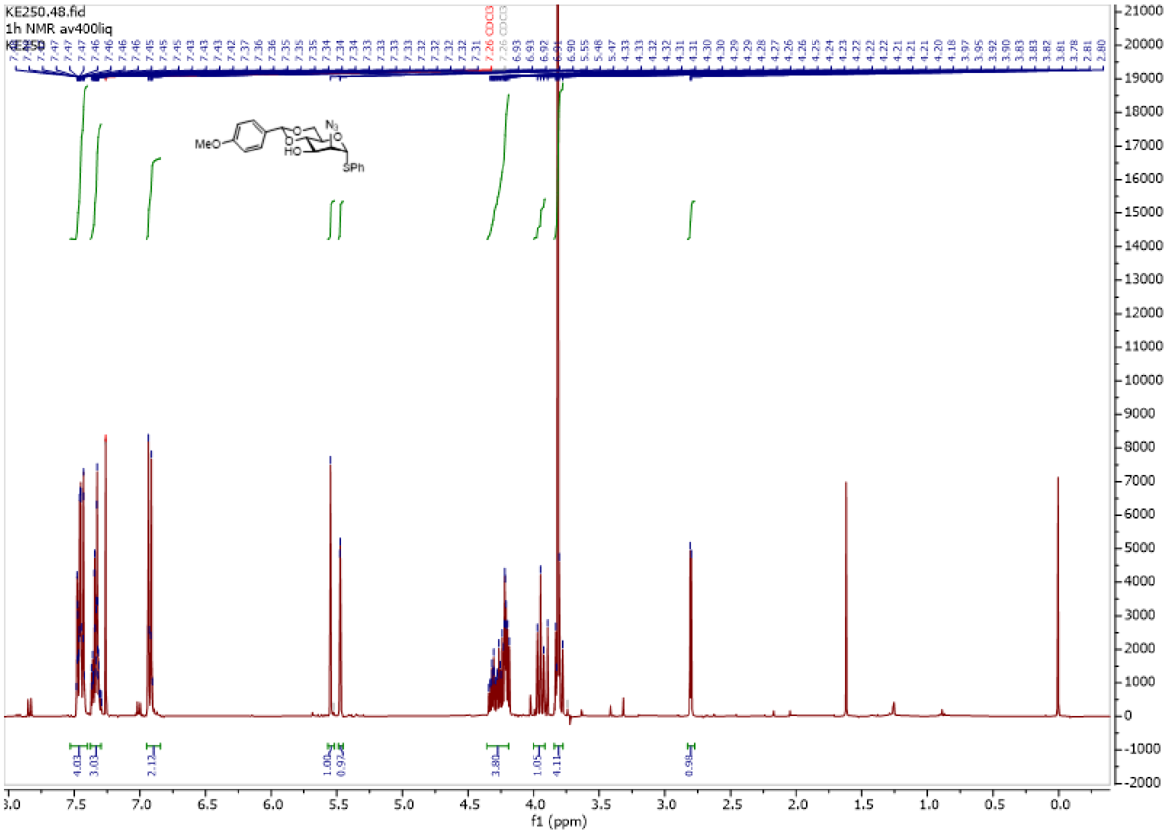

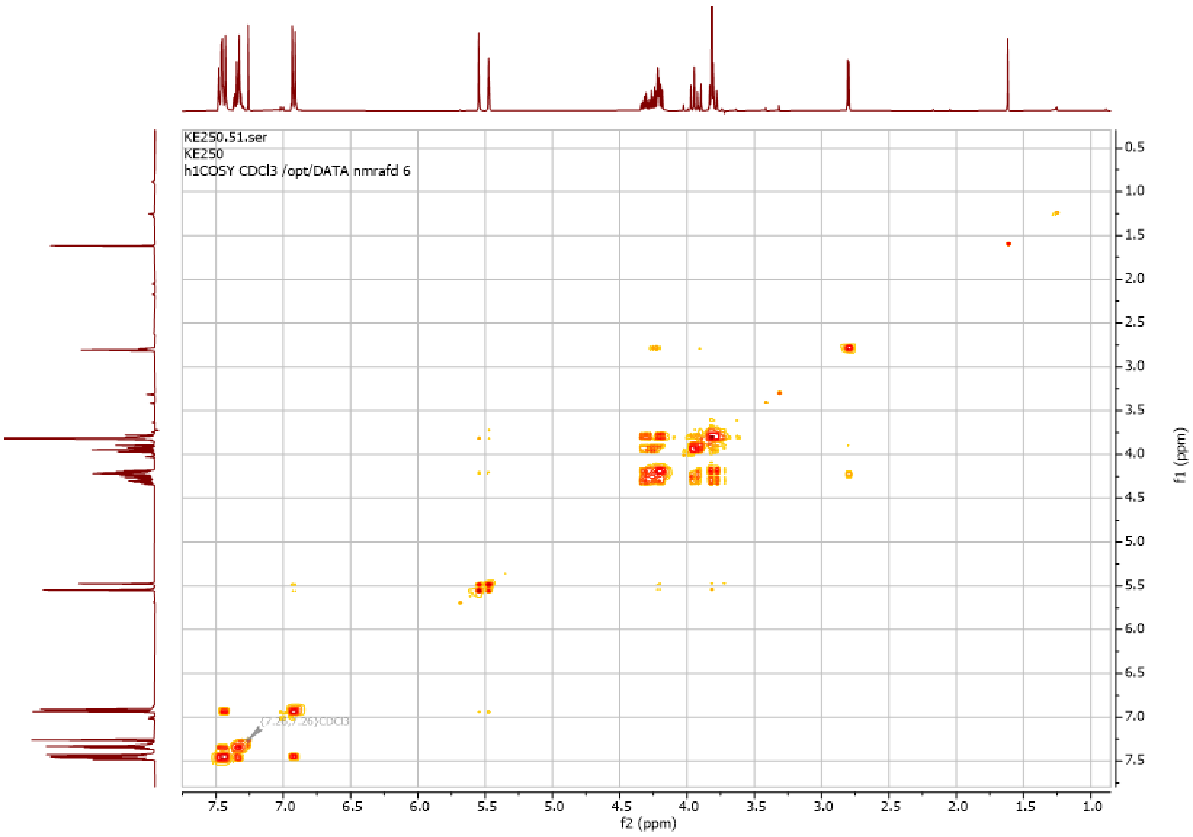

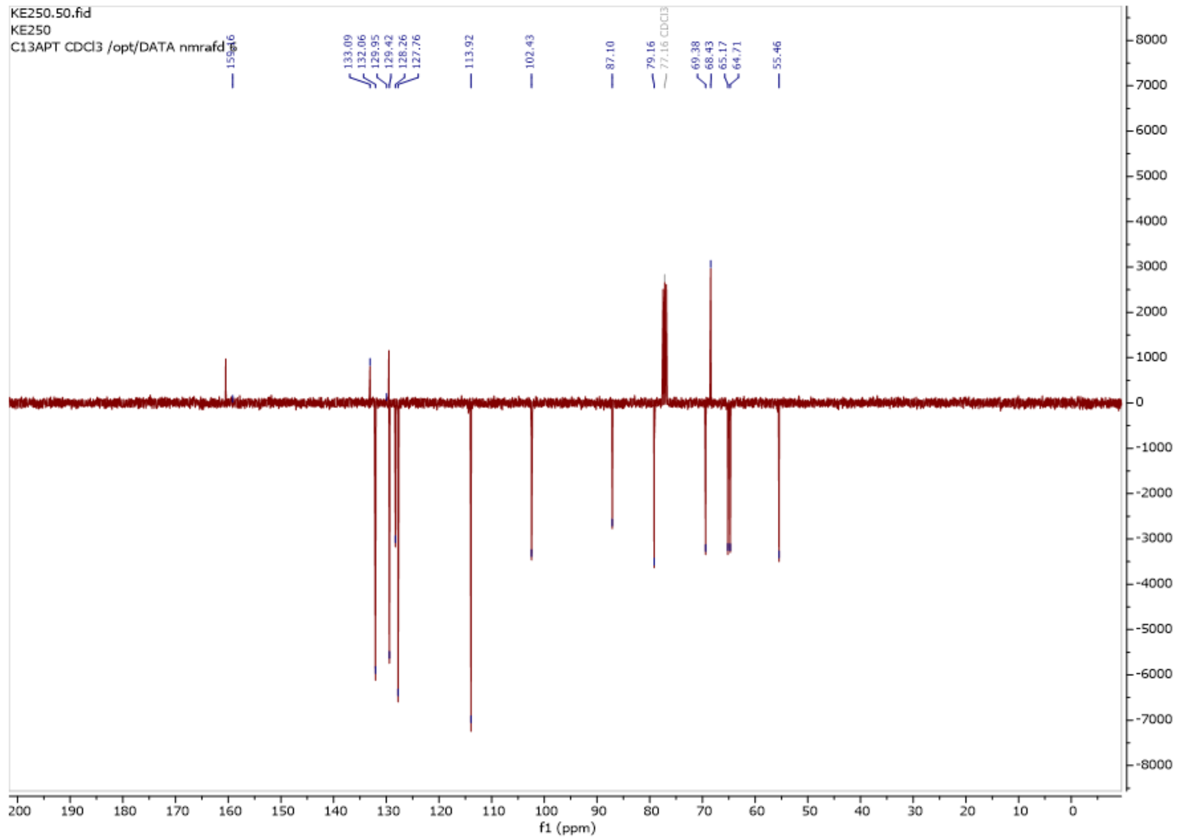
KE250.50.fid
KE250
C13APT CDCl3 /opt/DATA nmrafd 6
159.46
133.09
132.06
129.95
129.42
128.26
127.76
113.92
102.43
87.10
79.16
77.16 CDCl3
69.38
68.43
65.17
64.71
55.46
f1 (ppm)

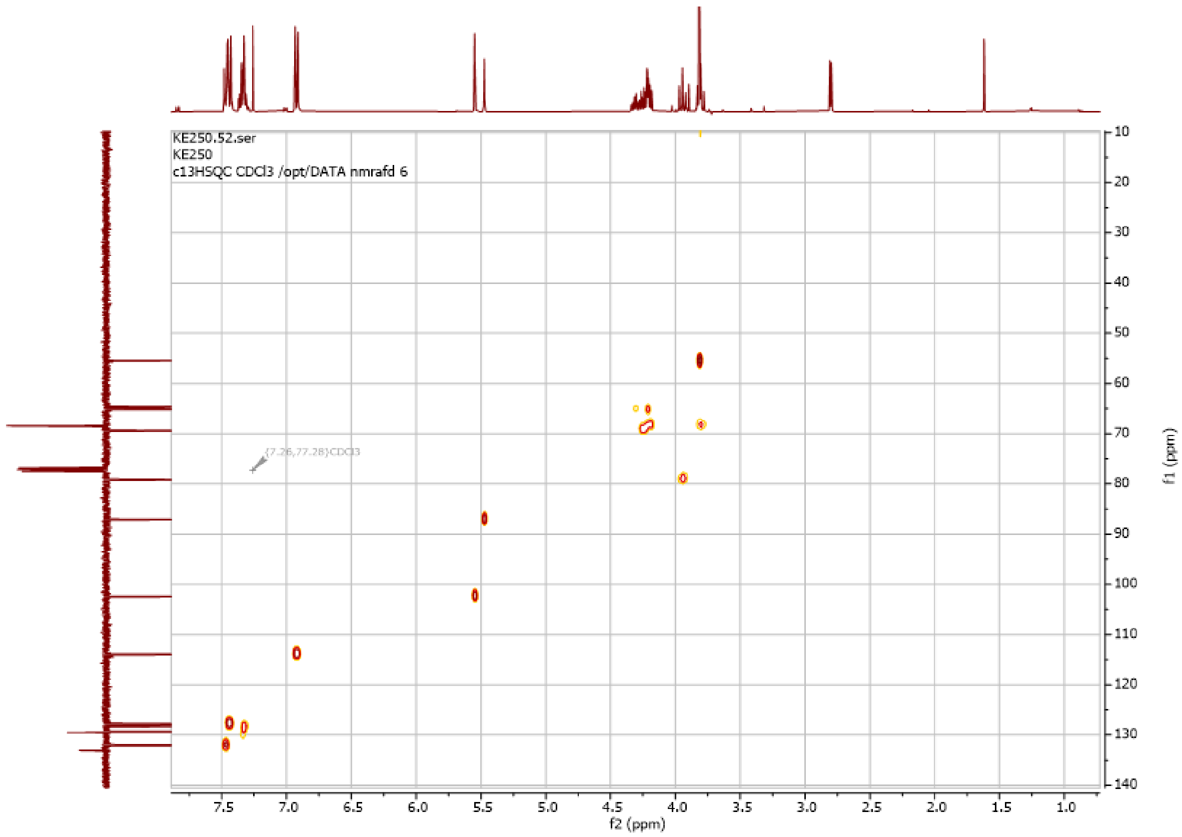
KE250.52.ser
KE250
c13HSQC CDCl3 /opt/DATA nmrafd 6
{7.26,77.28}CDCl3
f2 (ppm)
f1 (ppm)

Phenyl 2-azido-2-deoxy-4,6-*O*-(*p*-methoxybenzylidene)-3-*O*-(2-naphthylmethyl)-1-thio-*α*-D-mannopyranoside (S16)

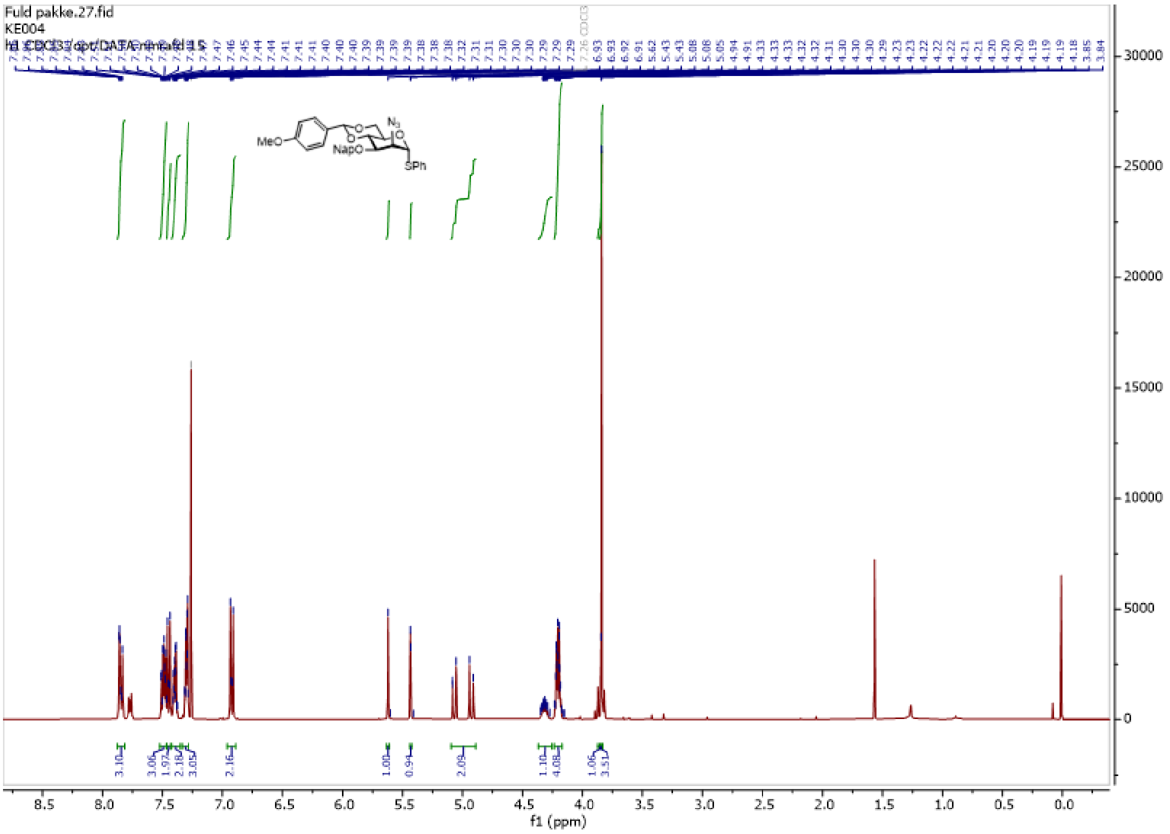

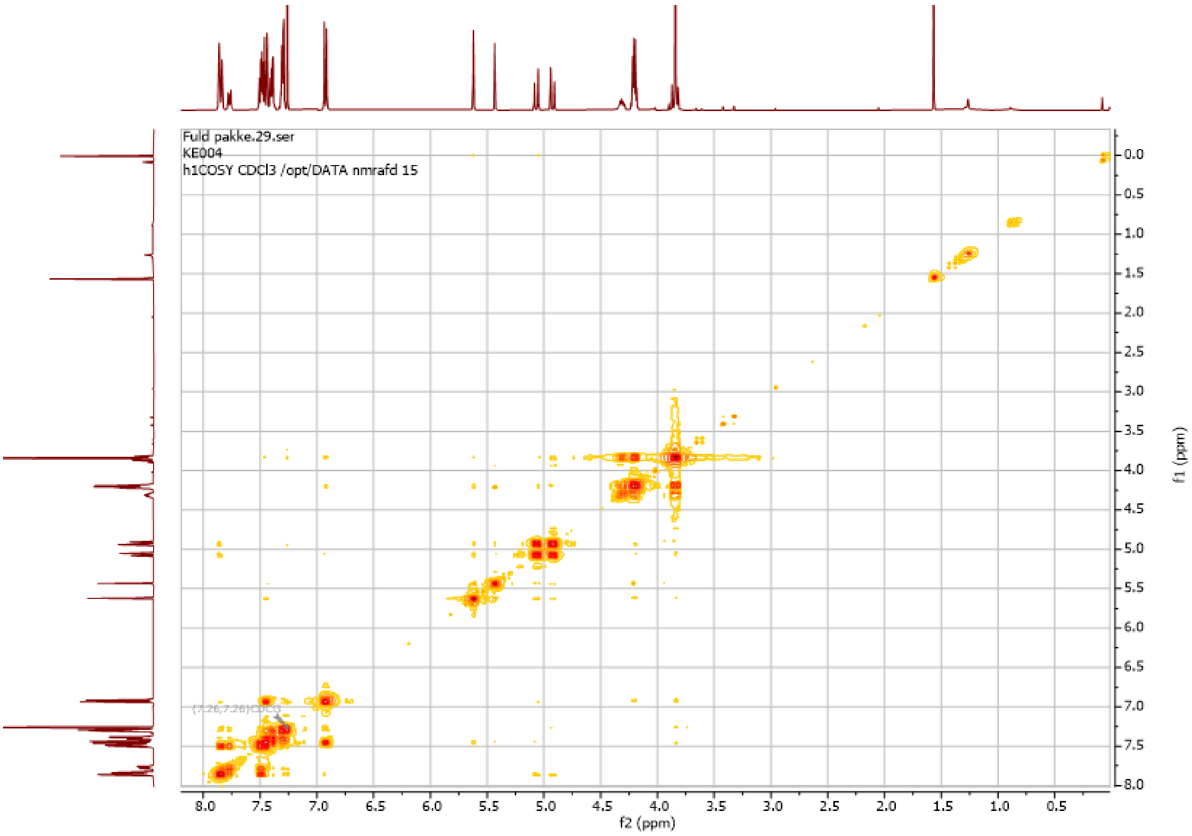

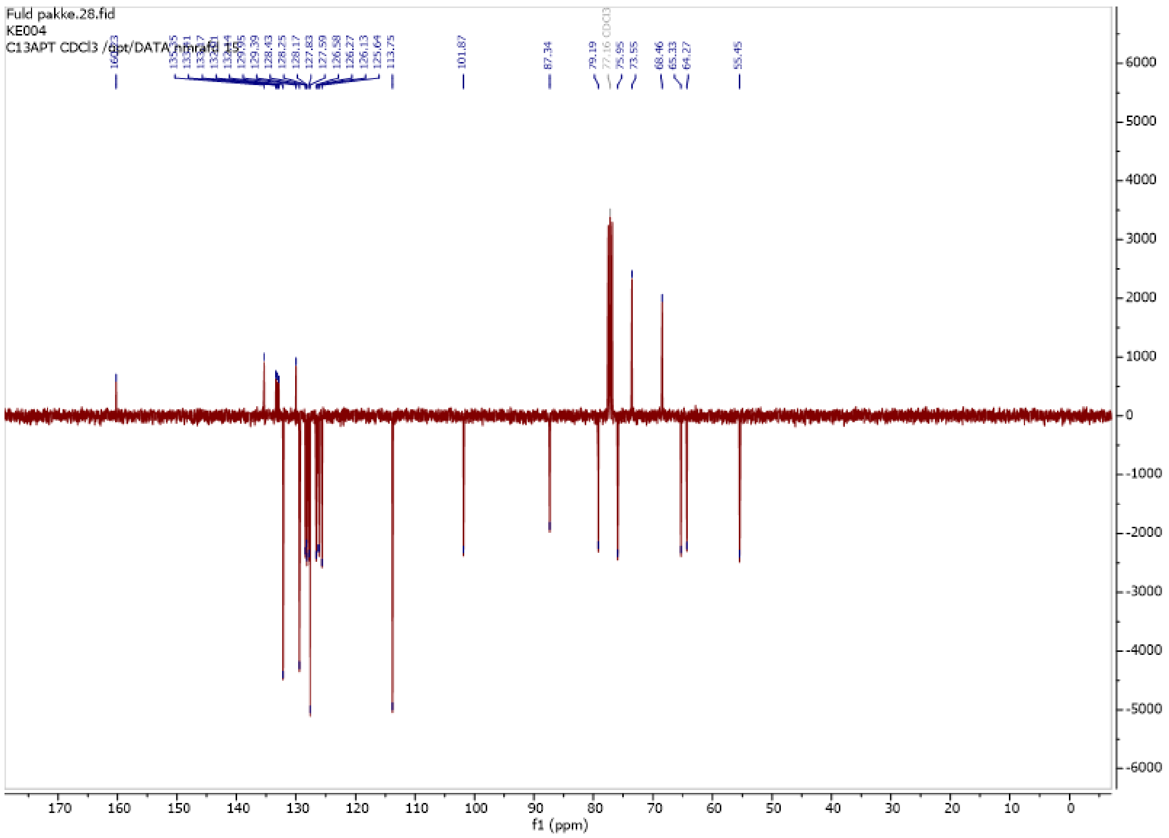
Fuld pakke.28.fid
KE004
C13APT CDCl3 /opt/DATA nmrafd 15
135.55
129.39
128.43
128.25
128.17
127.83
127.59
126.58
126.27
126.13
125.64
113.75
101.87
87.34
79.19
77.16 CDCl3
75.95
73.55
68.46
65.33
64.27
55.45
f1 (ppm)

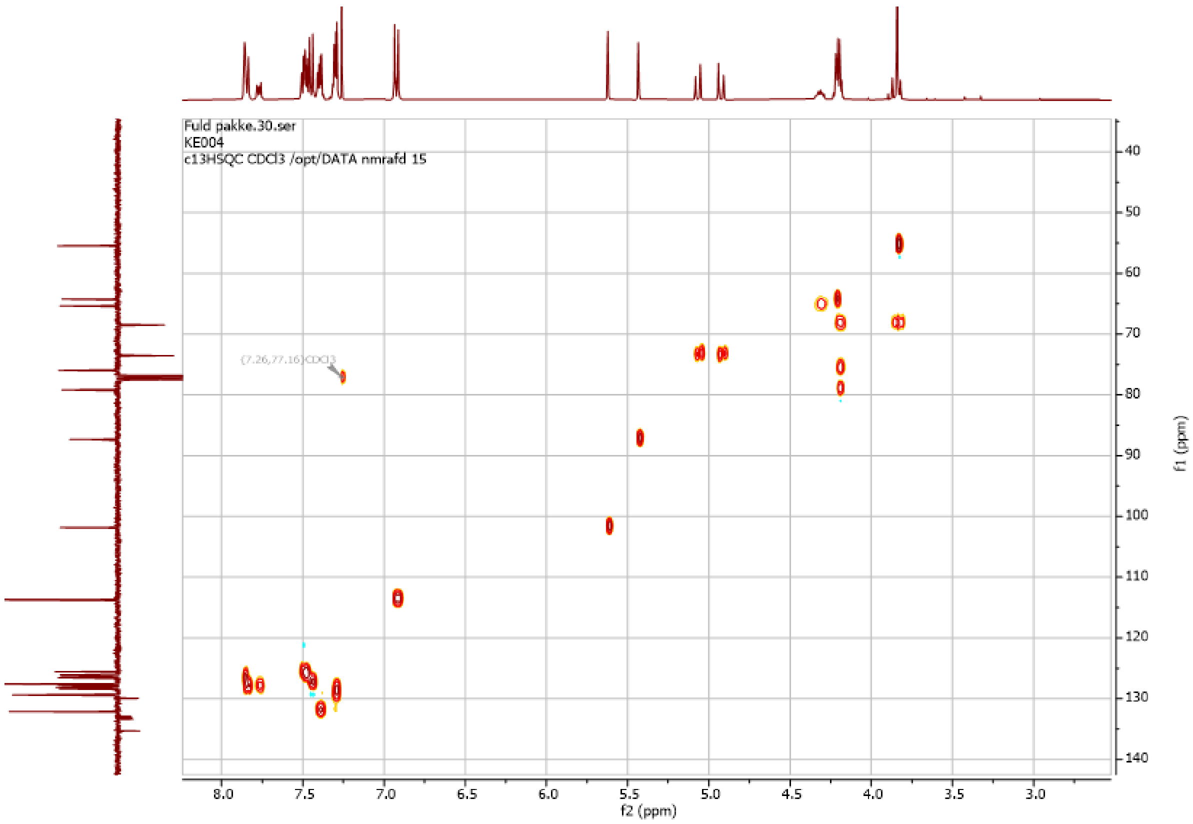
Fuld pakke.30.ser
KE004
c13HSQC CDCl3 /opt/DATA nmrafd 15
(7.26,77.16)CDCl3
f2 (ppm)
f1 (ppm)

Phenyl 2-azido-2-deoxy-3-*O*-(2-naphthylmethyl)-1-thio-*α*-D-mannopyranoside (S17)

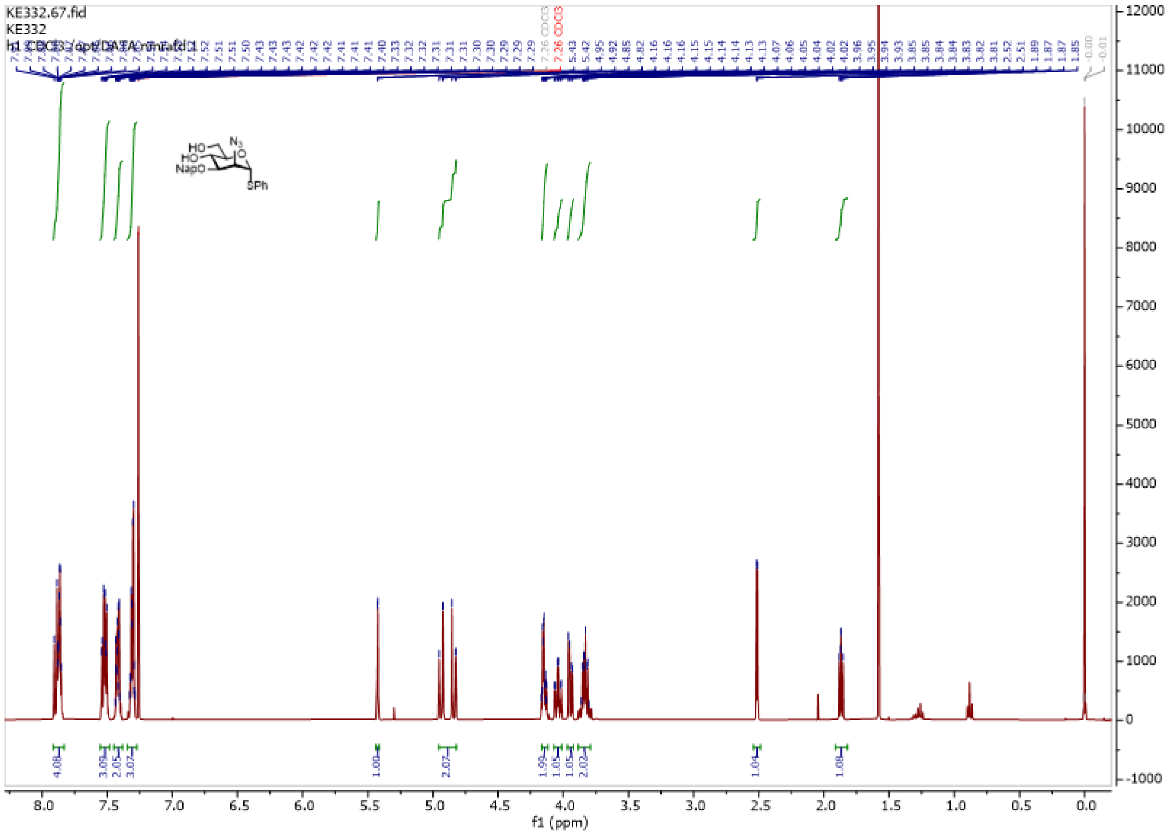

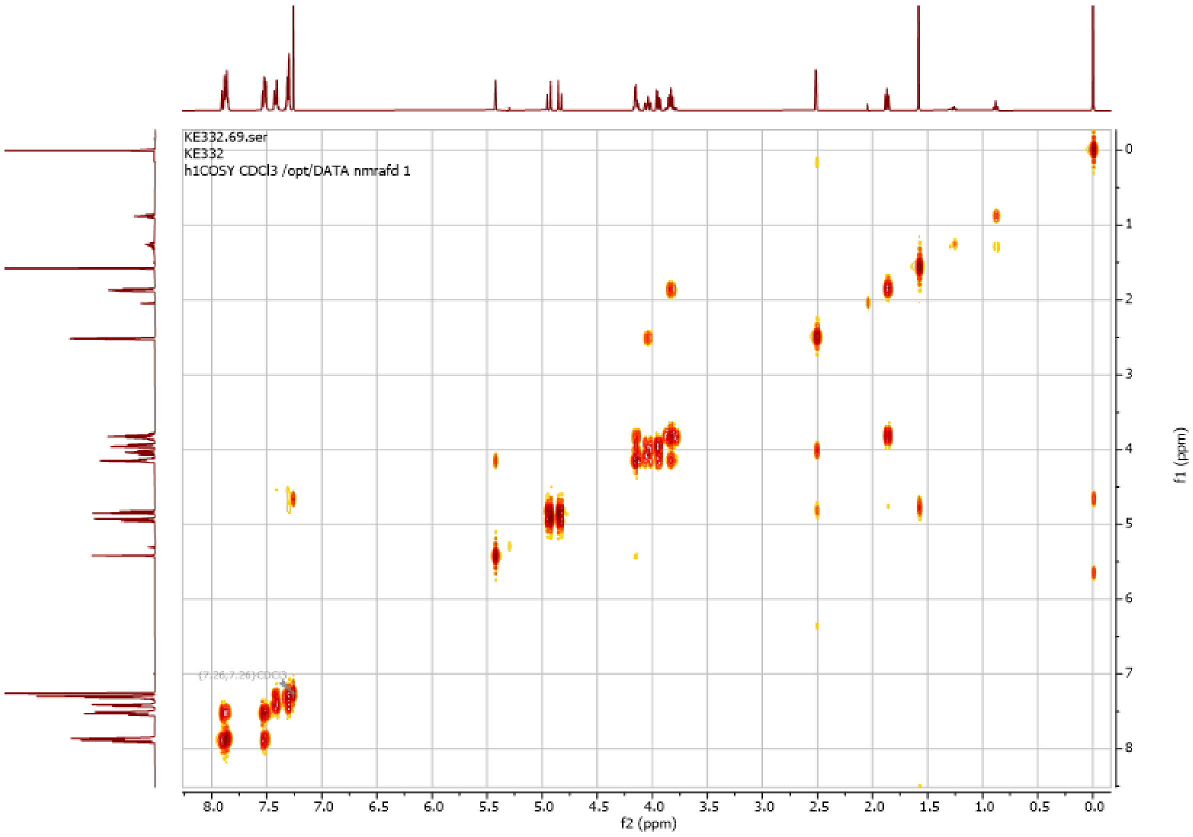

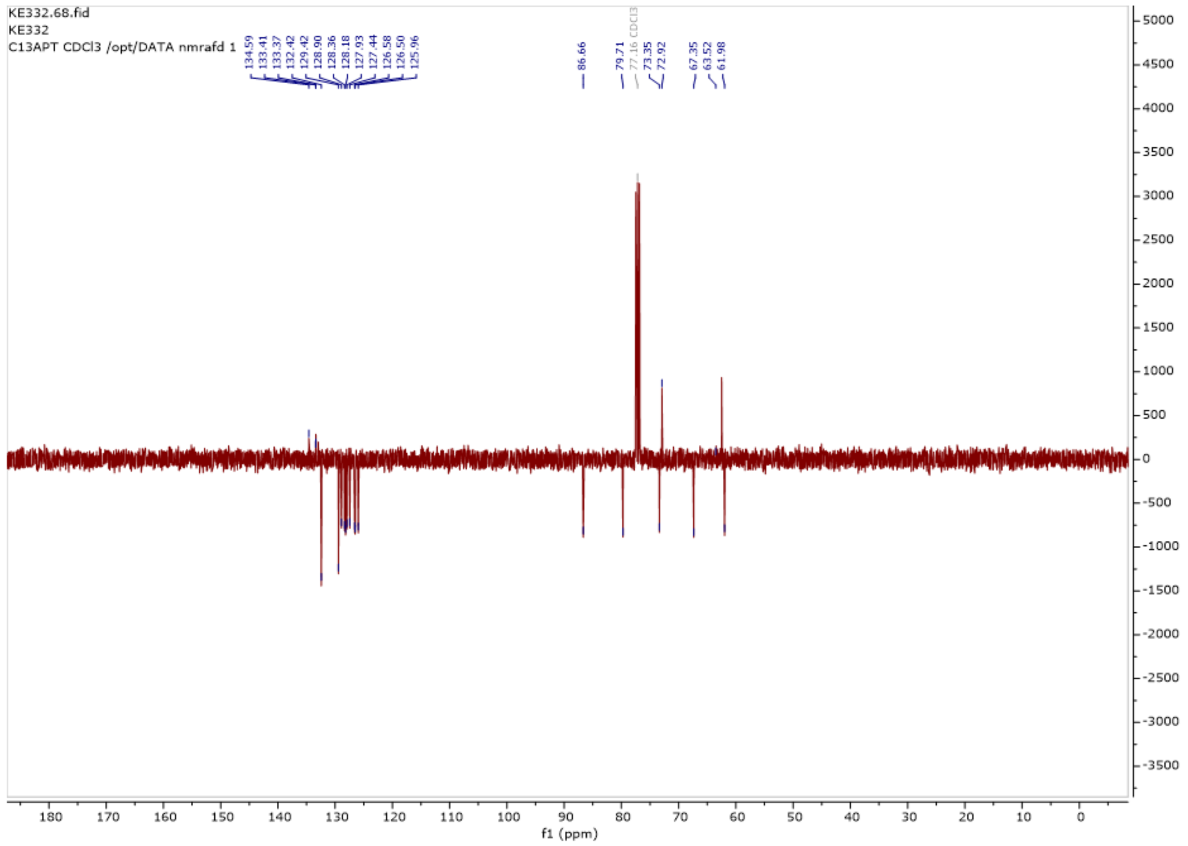

KE332.68.fid
KE332
C13APT CDCl3 /opt/DATA nmrafd 1
134.59
133.41
133.37
132.42
129.42
128.90
128.36
128.18
127.93
127.44
126.58
126.50
125.96
86.66
79.71
77.16 CDCl3
73.35
72.92
67.35
63.52
61.98
f1 (ppm)

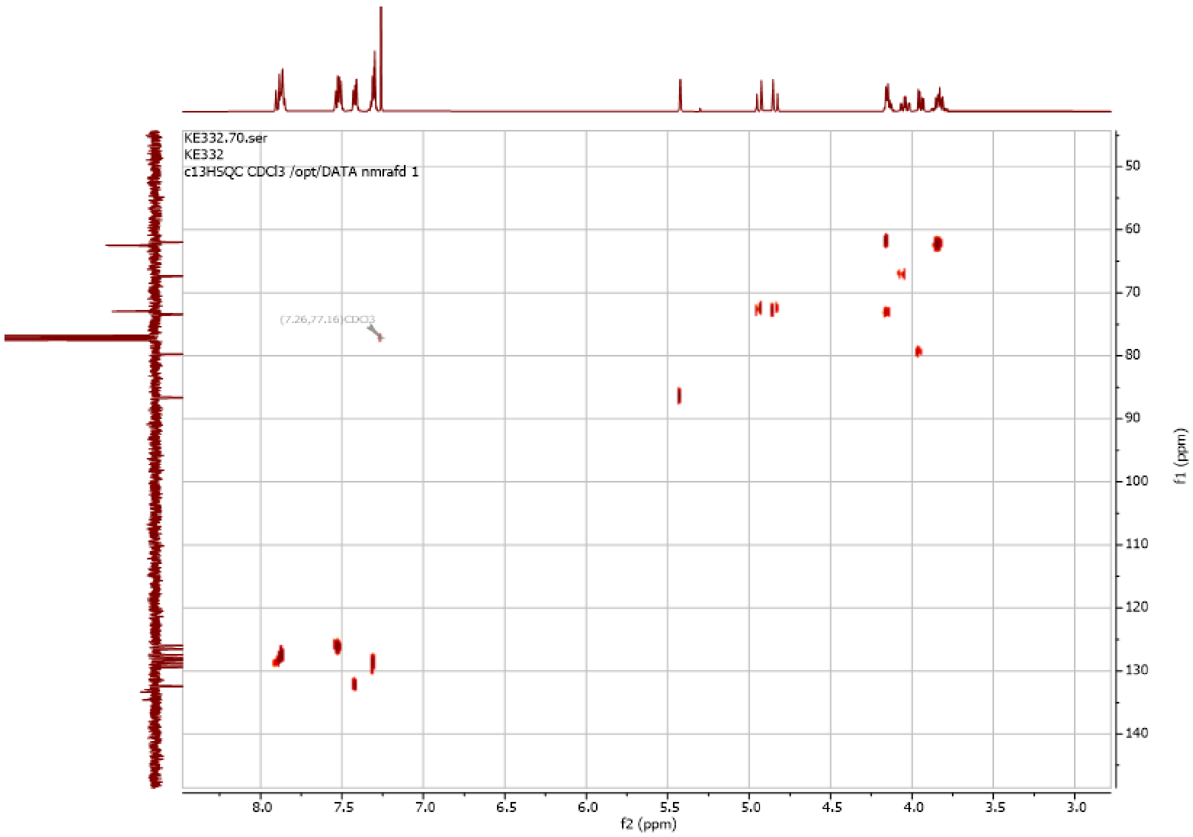

KE332.70.ser
KE332
c13HSQC CDCl3 /opt/DATA nmrafd 1
(7.26,77.16) CDCl3
f2 (ppm)
f1 (ppm)

Benzyl (phenyl 2-azido-2-deoxy-3-*O*-(2-naphthylmethyl)-1-thio-*α*-D-mannopyranosiduronate) (S18)

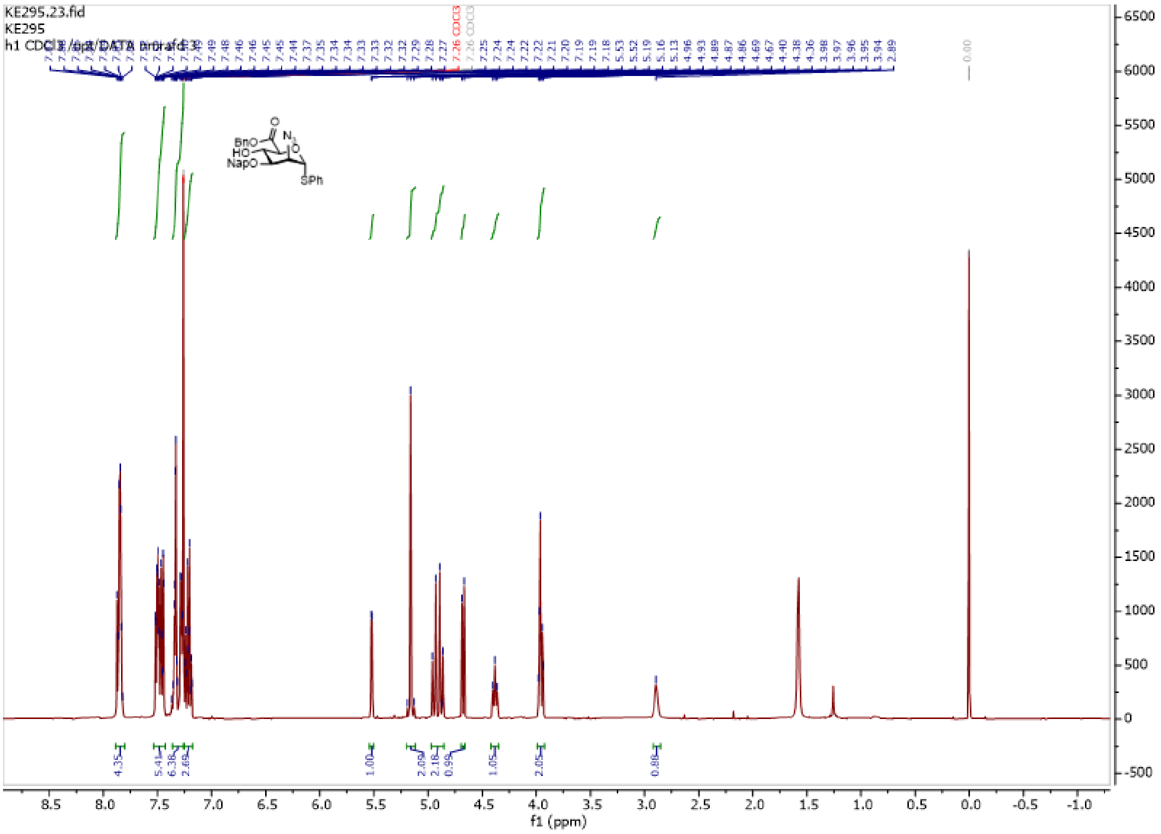

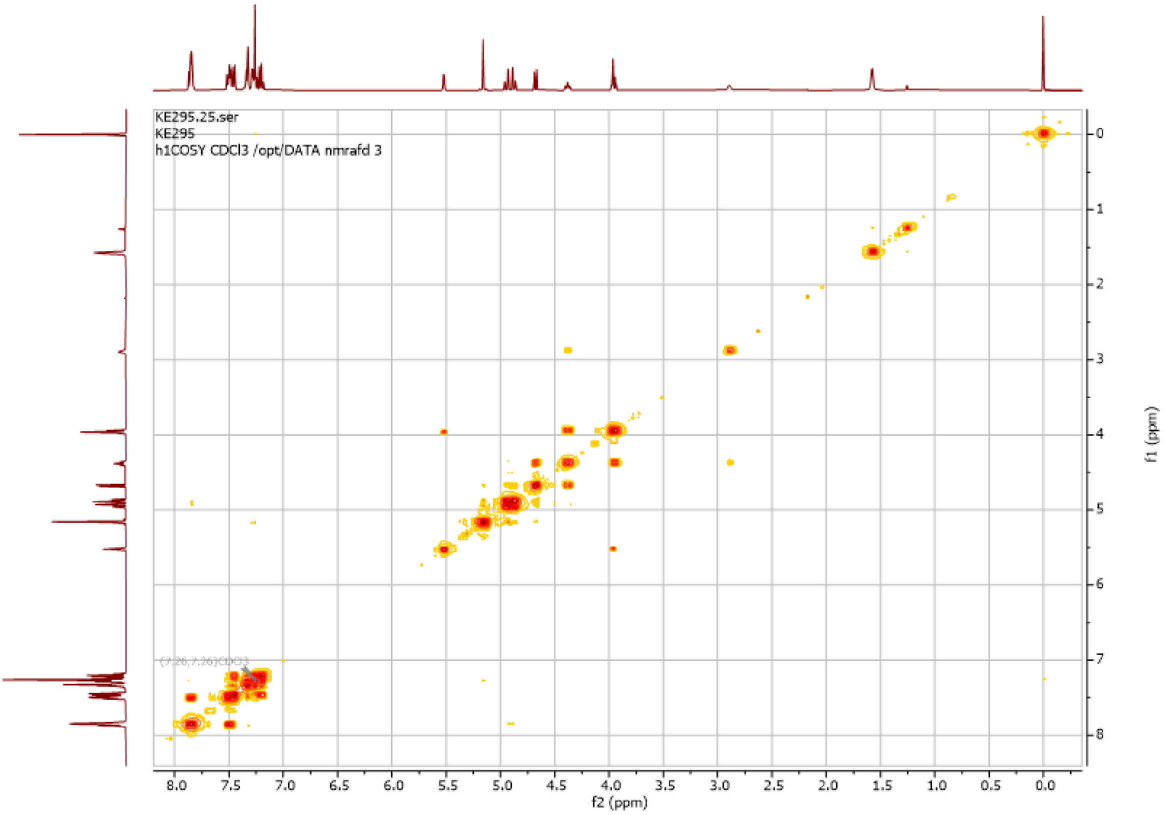

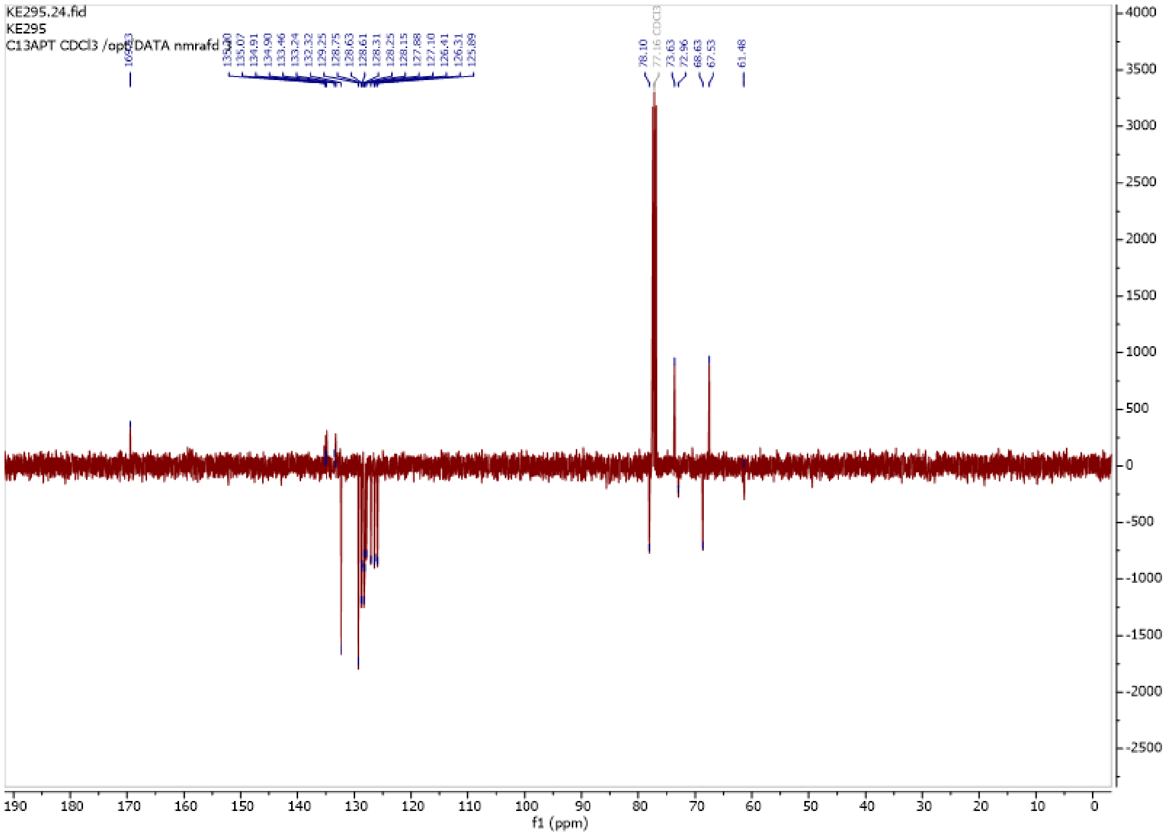
KE295.24.fid
KE295
C13APT CDCl3 /opt/DATA nmrafd
135.07
134.91
134.90
133.46
133.24
132.32
129.25
128.75
128.63
128.61
128.31
128.25
128.15
127.88
127.10
126.41
126.31
125.89
78.10
77.16 CDCl3
73.63
72.96
68.63
67.53
61.48
f1 (ppm)

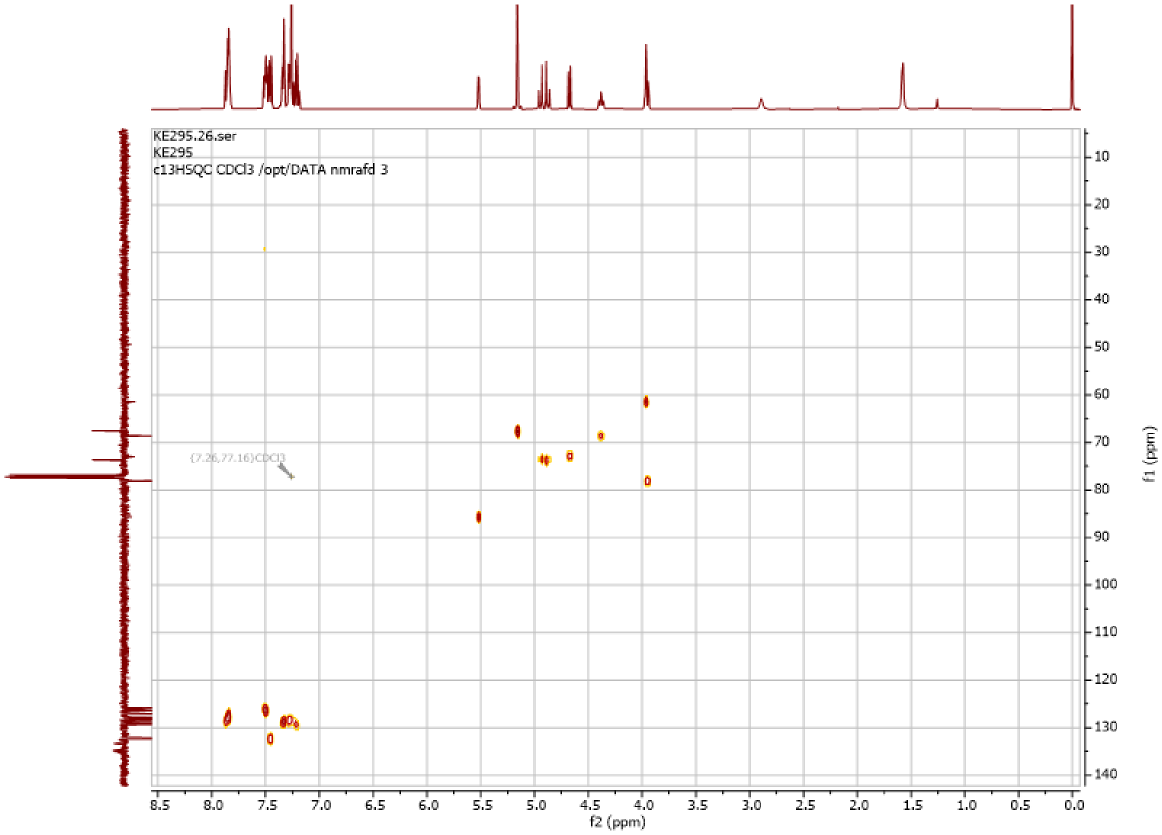
KE295.26.ser
KE295
c13HSQC CDCl3 /opt/DATA nmrafd 3
{7.26,77.16}CDCl3
f2 (ppm)
f1 (ppm)

Benzyl (phenyl 4-*O*-acetyl-2-azido-2-deoxy-3-*O*-(2-naphthylmethyl)-1-thio-*α*-D-mannopyranosiduronate) (12a)

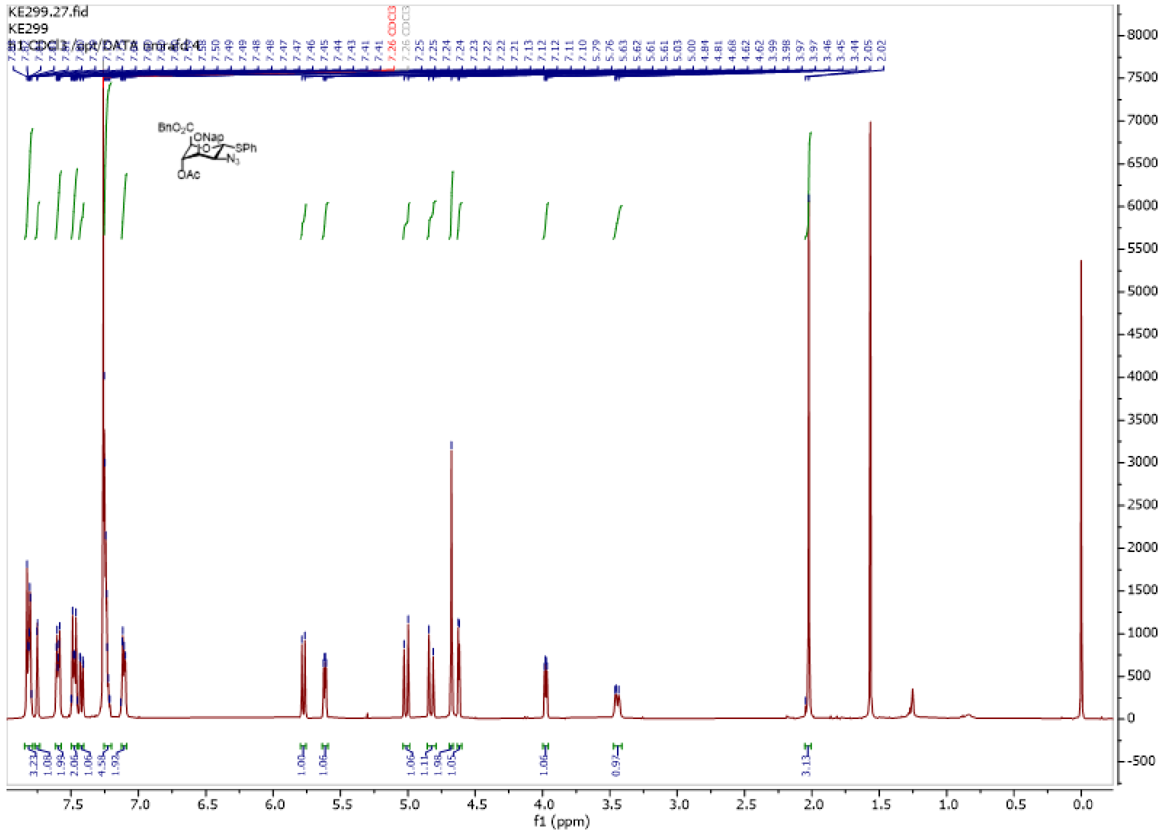

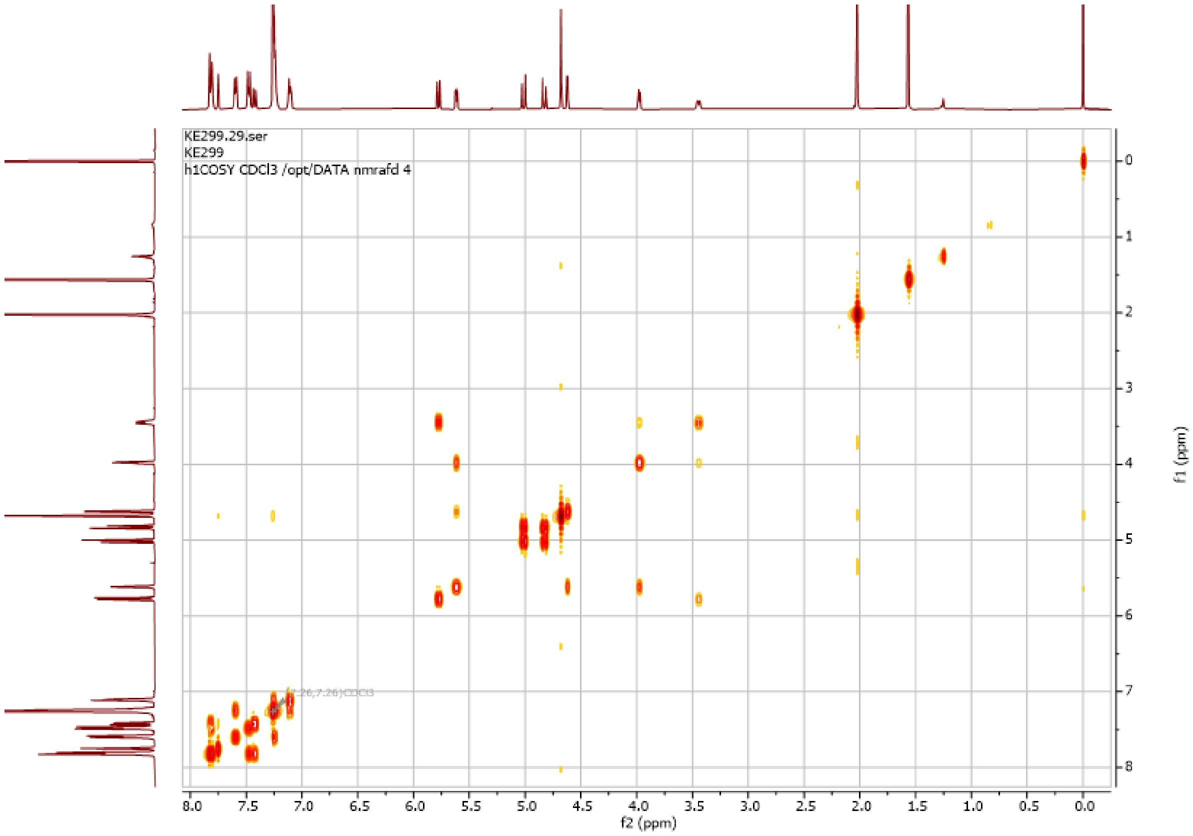

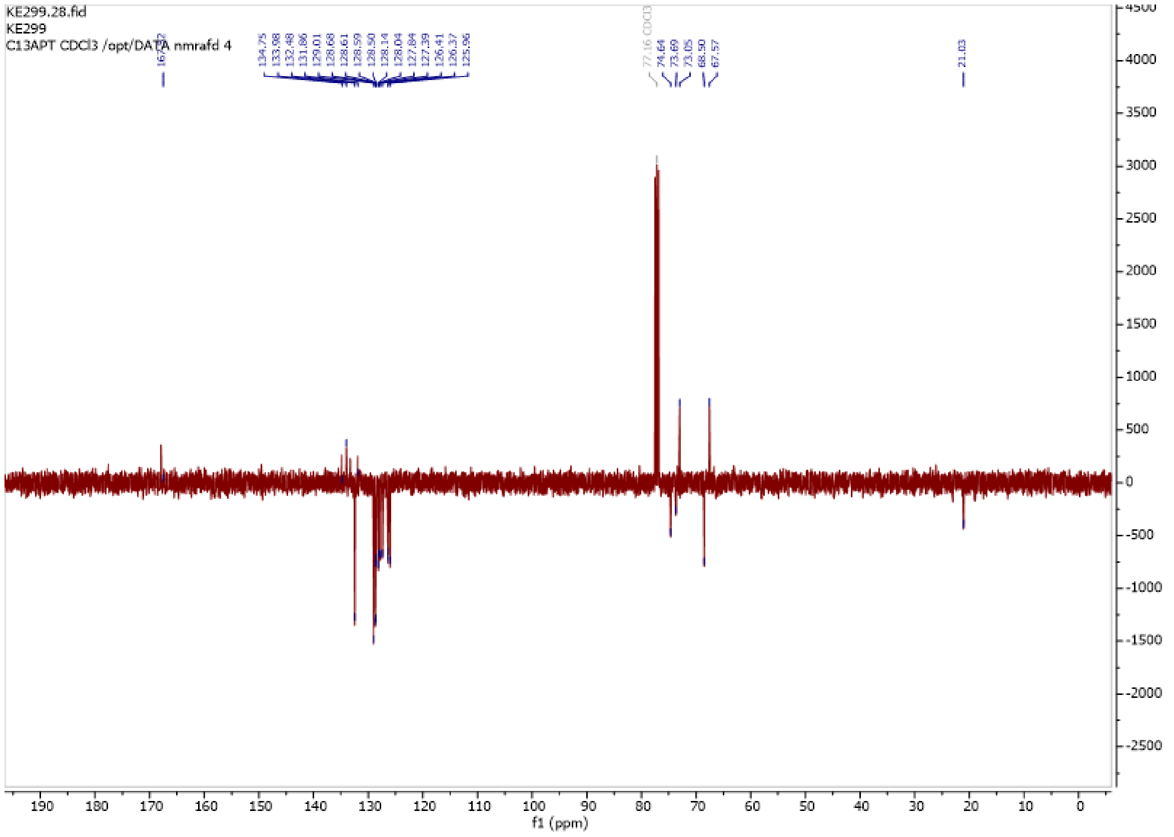
KE299.28.fid
KE299
C13APT CDCl3 /opt/DATA nmrafd 4
134.75
133.98
132.48
131.86
129.01
128.68
128.61
128.59
128.50
128.14
128.04
127.84
127.39
126.41
126.37
125.96
77.16 CDCl3
74.64
73.69
73.05
68.50
67.57
21.03
f1 (ppm)

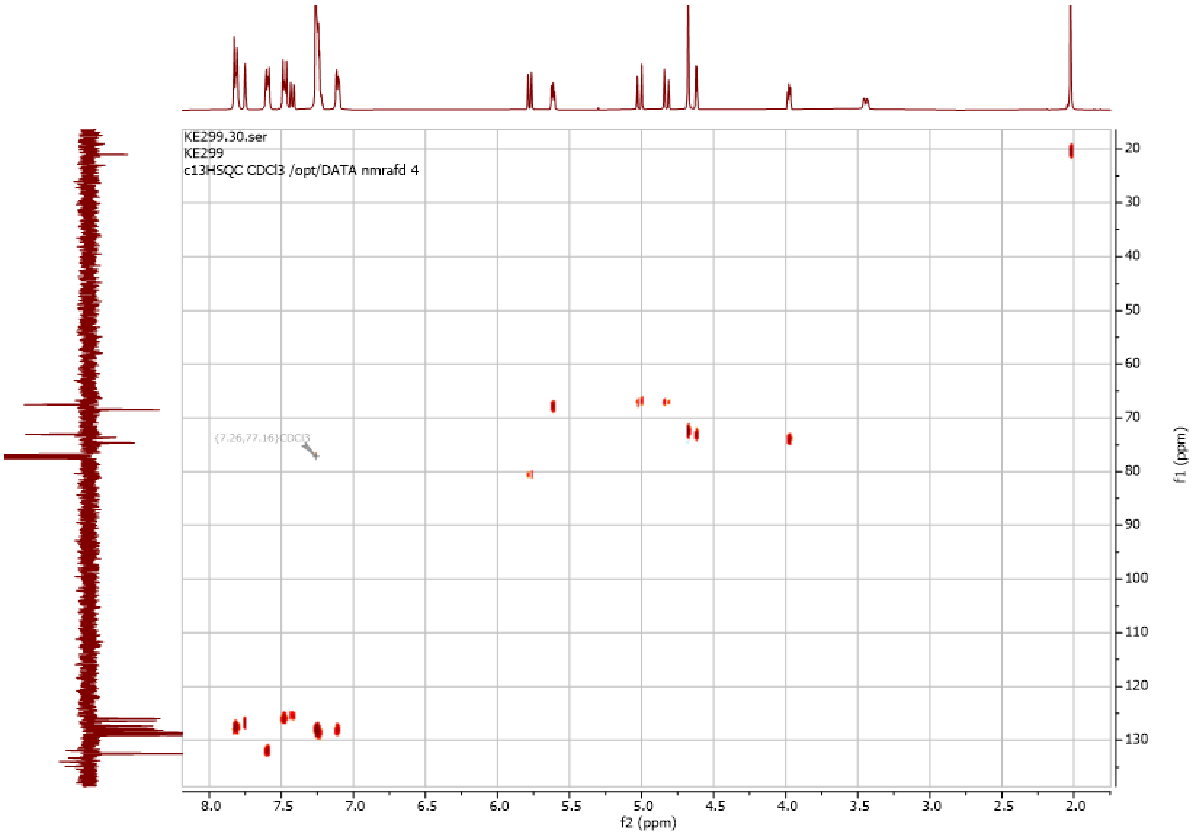
KE299.30.ser
KE299
c13HSQC CDCl3 /opt/DATA nmrafd 4
(7.26,77.16)CDCl3
f2 (ppm)
f1 (ppm)

Benzyl (4-*O*-acetyl-2-azido-2-deoxy-3-*O*-(2-naphthylmethyl)-*α*-D-mannopyranosiduronate) (S19)

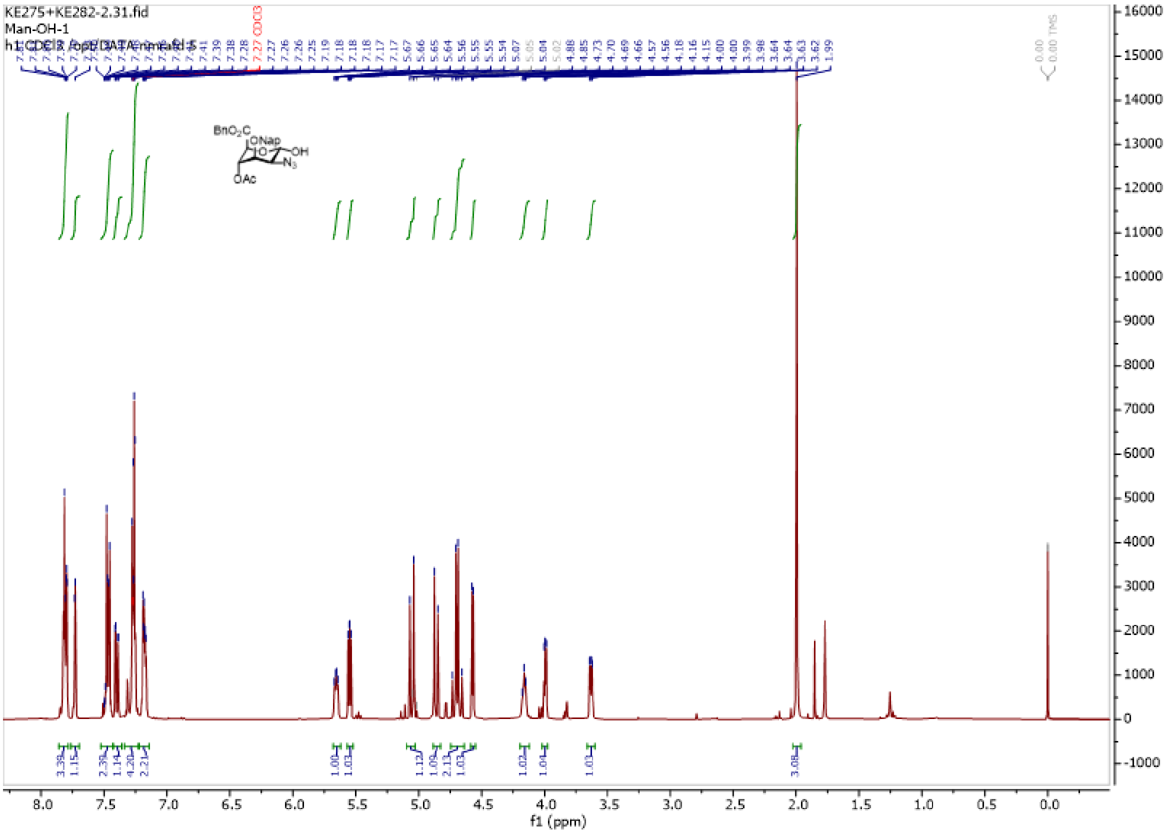

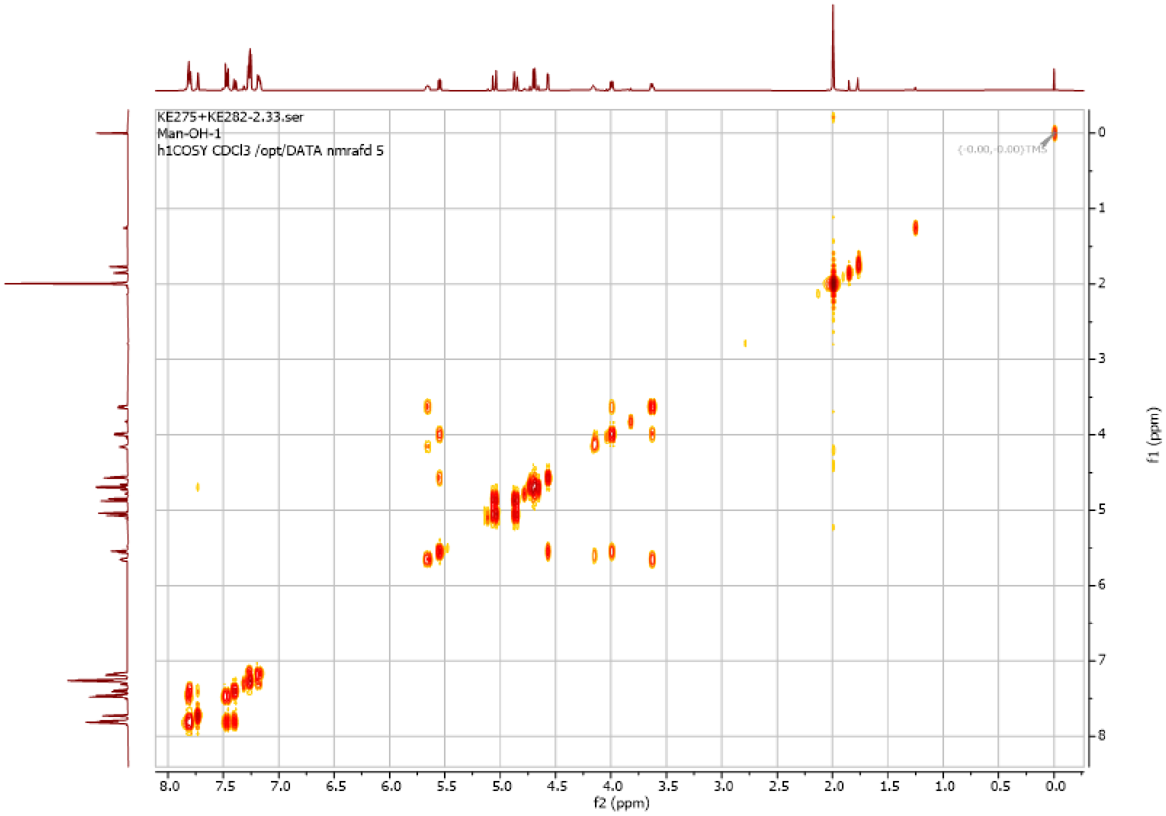

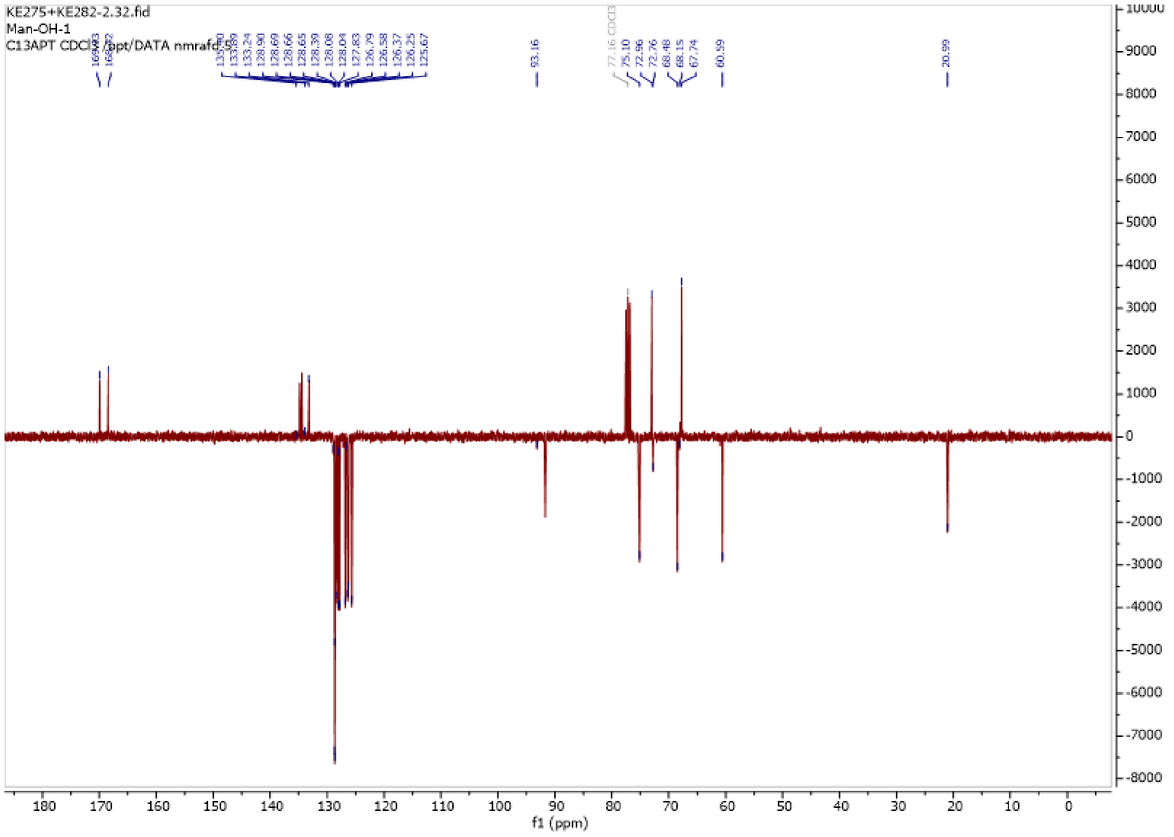
KE275+KE282-2.32.fid
Man-OH-1
C13APT CDCl3 /opt/DATA nmrafd 5
f1 (ppm)

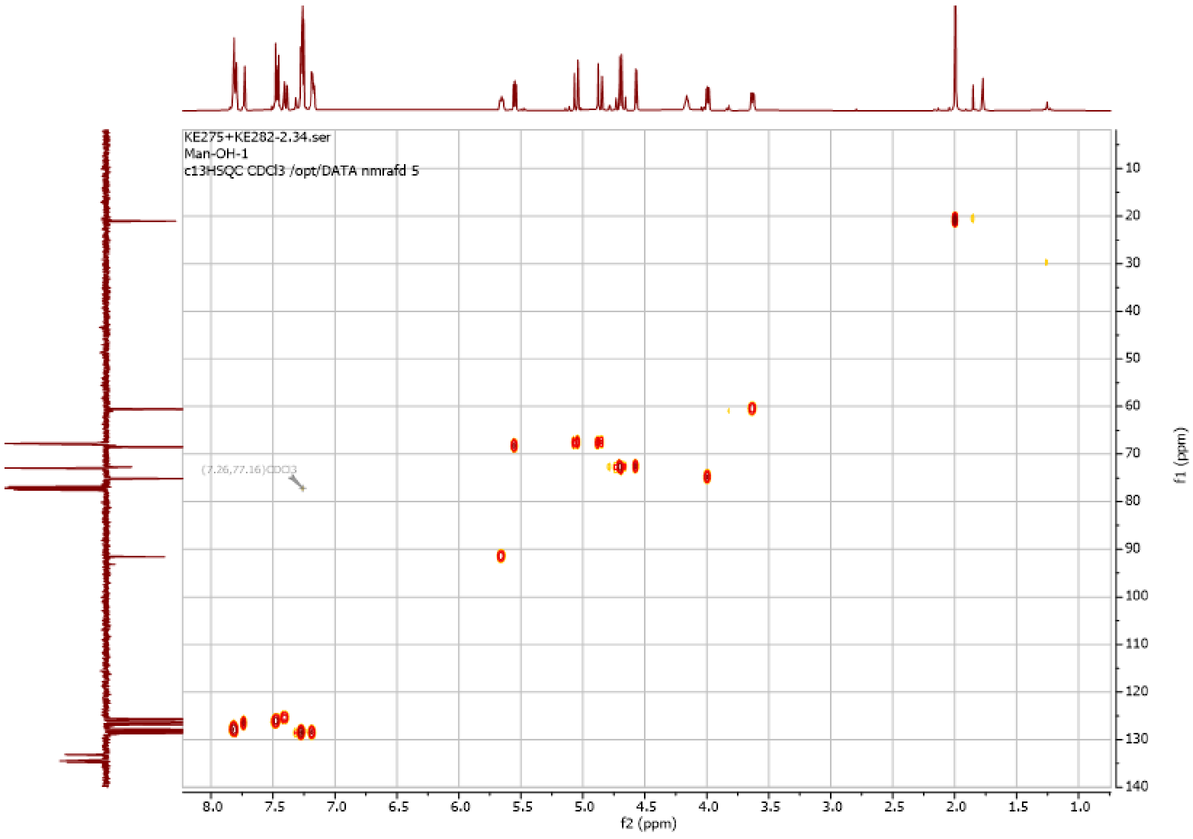
KE275+KE282-2.34.ser
Man-OH-1
c13HSQC CDCl3 /opt/DATA nmrafd 5
f2 (ppm)
f1 (ppm)

Benzyl (4-*O*-acetyl-2-azido-2-deoxy-3-*O*-(2-naphthylmethyl)-1-*O*-(N-phenyl-2,2,2-trifluoroacetimidoyl)-*α*/*β*-D-mannopyranosiduronate) (12b)

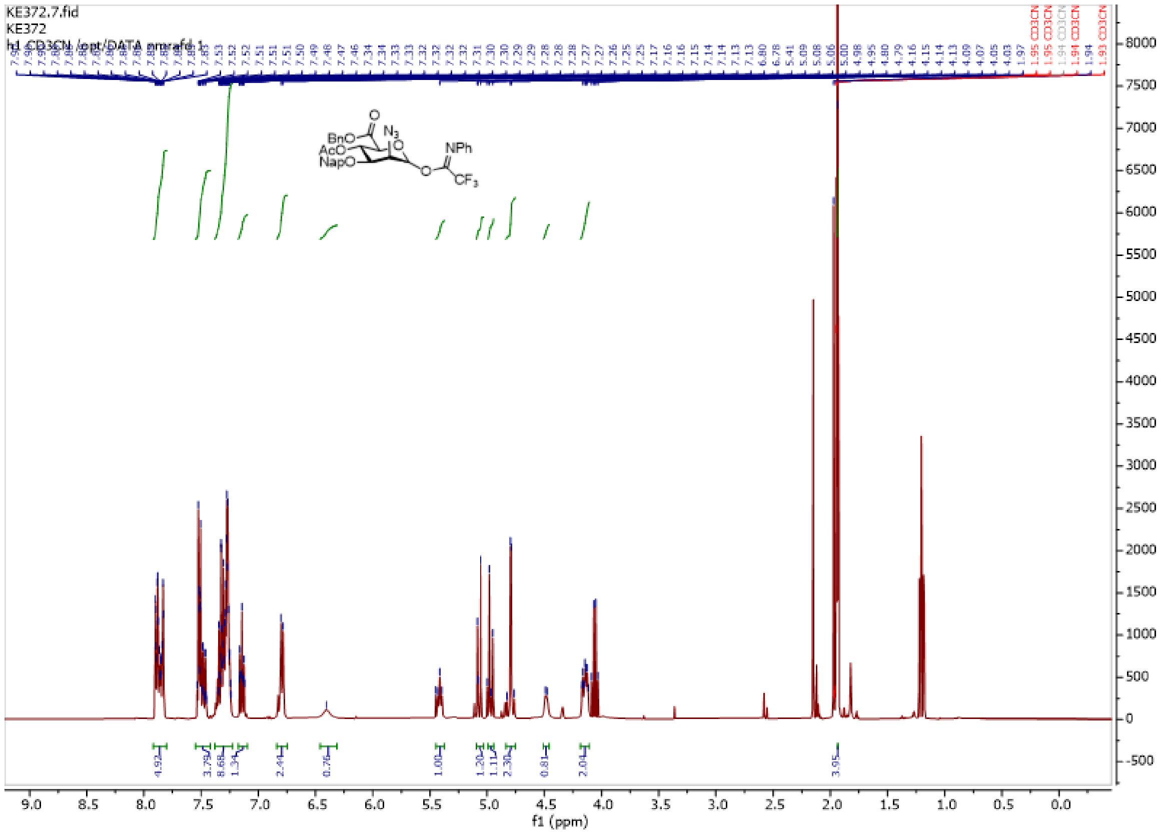

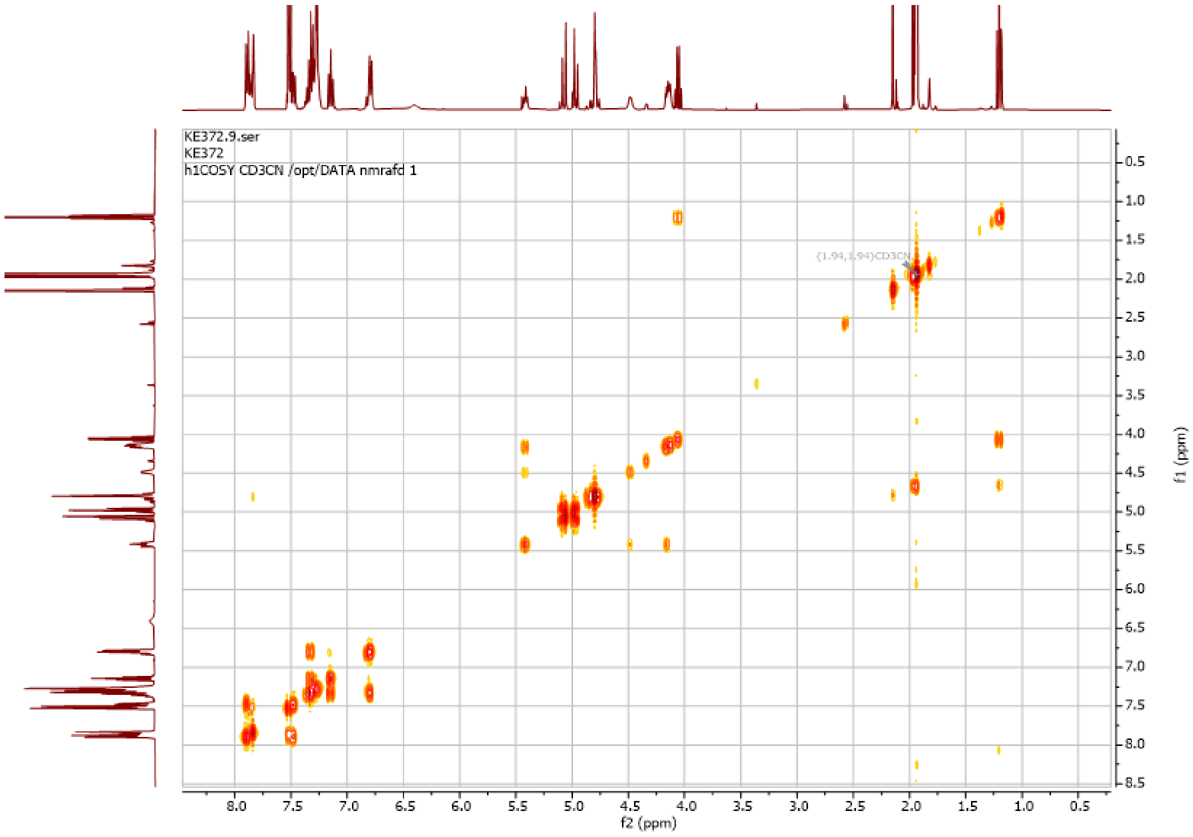

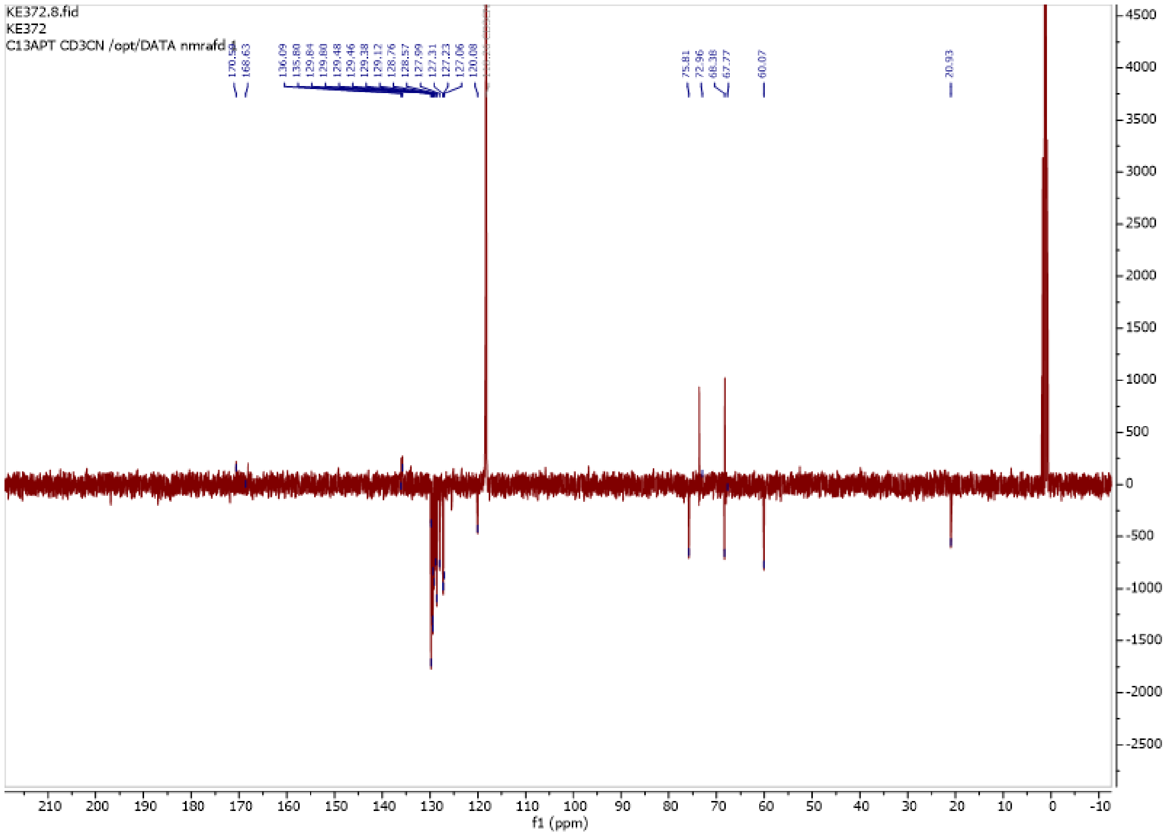
KE372.8.fid
KE372
C13APT CD3CN /opt/DATA nmrafd 1
170.58
168.63
136.09
135.80
129.84
129.80
129.48
129.46
129.38
129.12
128.76
128.57
127.99
127.31
127.23
127.06
120.08
75.81
72.96
68.38
67.77
60.07
20.93
f1 (ppm)

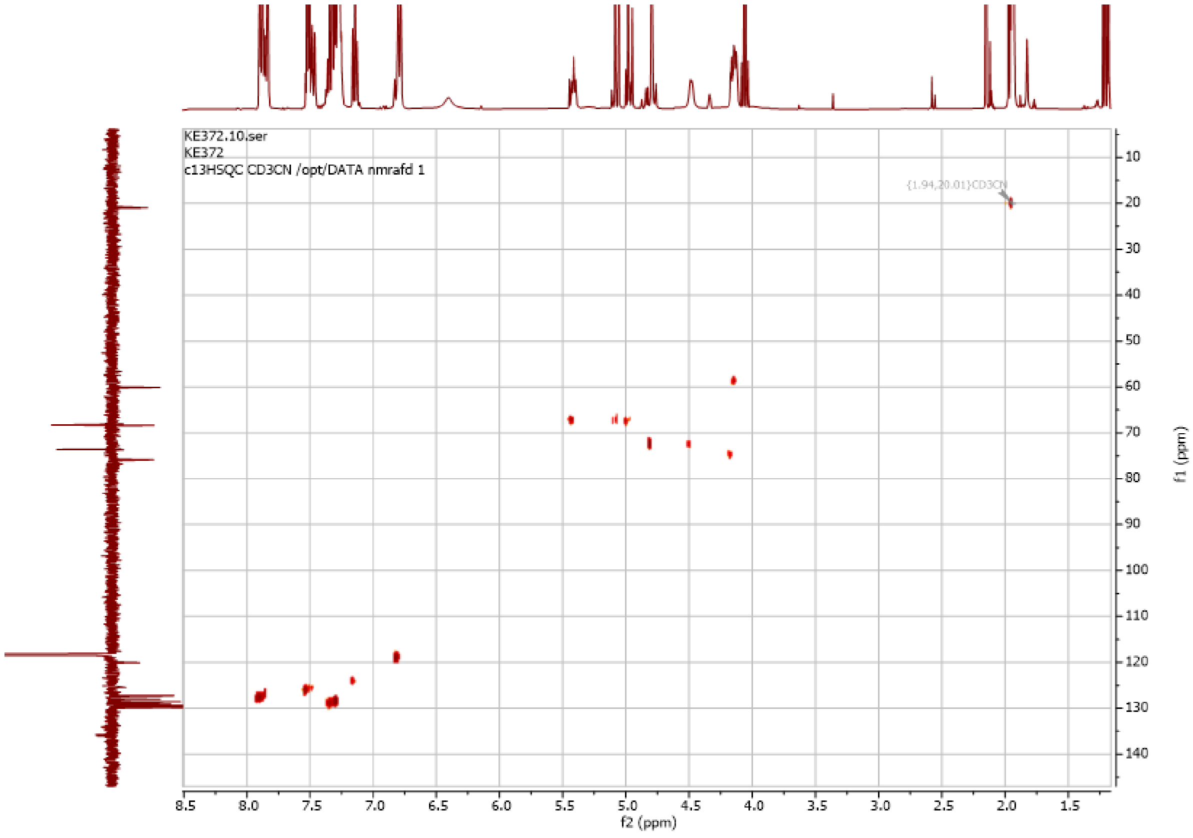
KE372.10.ser
KE372
c13HSQC CD3CN /opt/DATA nmrafd 1
f2 (ppm)
f1 (ppm)

***Tert*-butyldiphenylsilyl 2-azido-4-*O*-benzyl-2-deoxy-3-*O*-(2-naphthylmethyl)-*α*-L-fucopyranosyl-(1→3)-2-azido-4-*O*-benzyl-2-deoxy-*β*-D-fucopyranoside (13)**

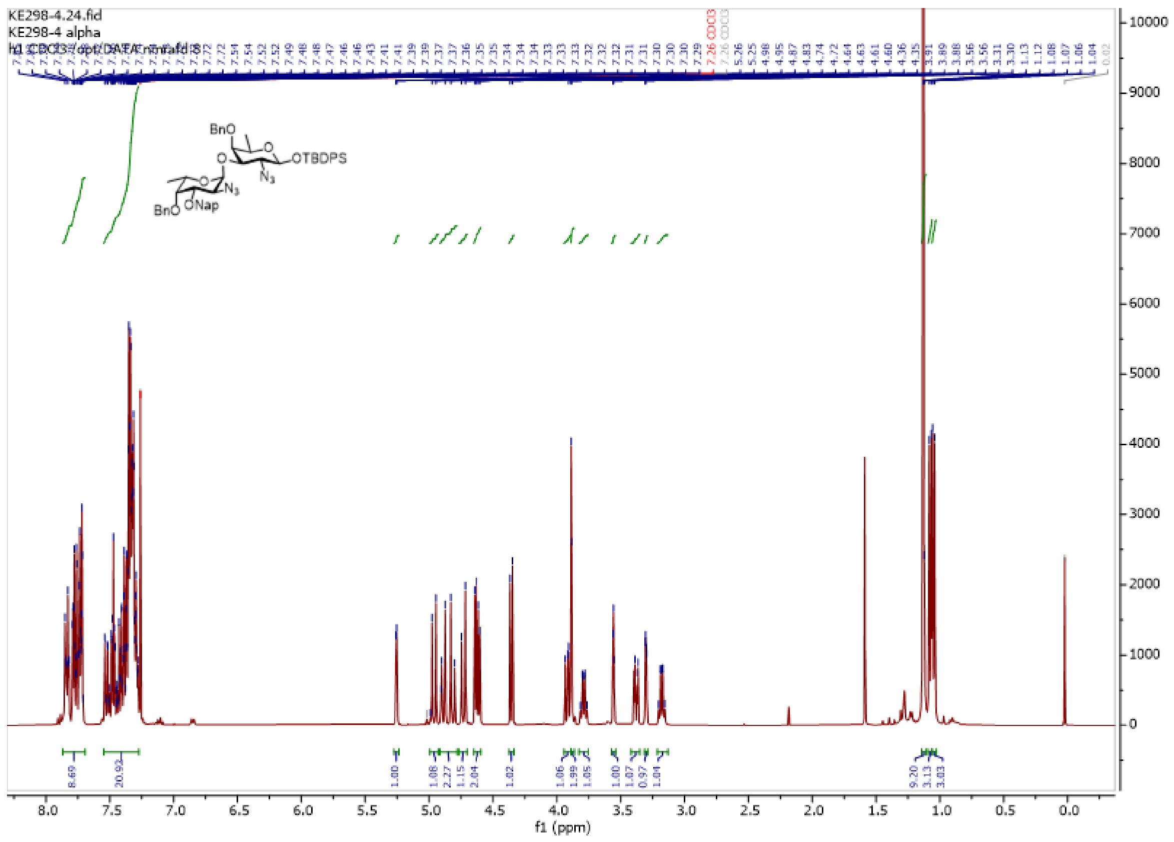

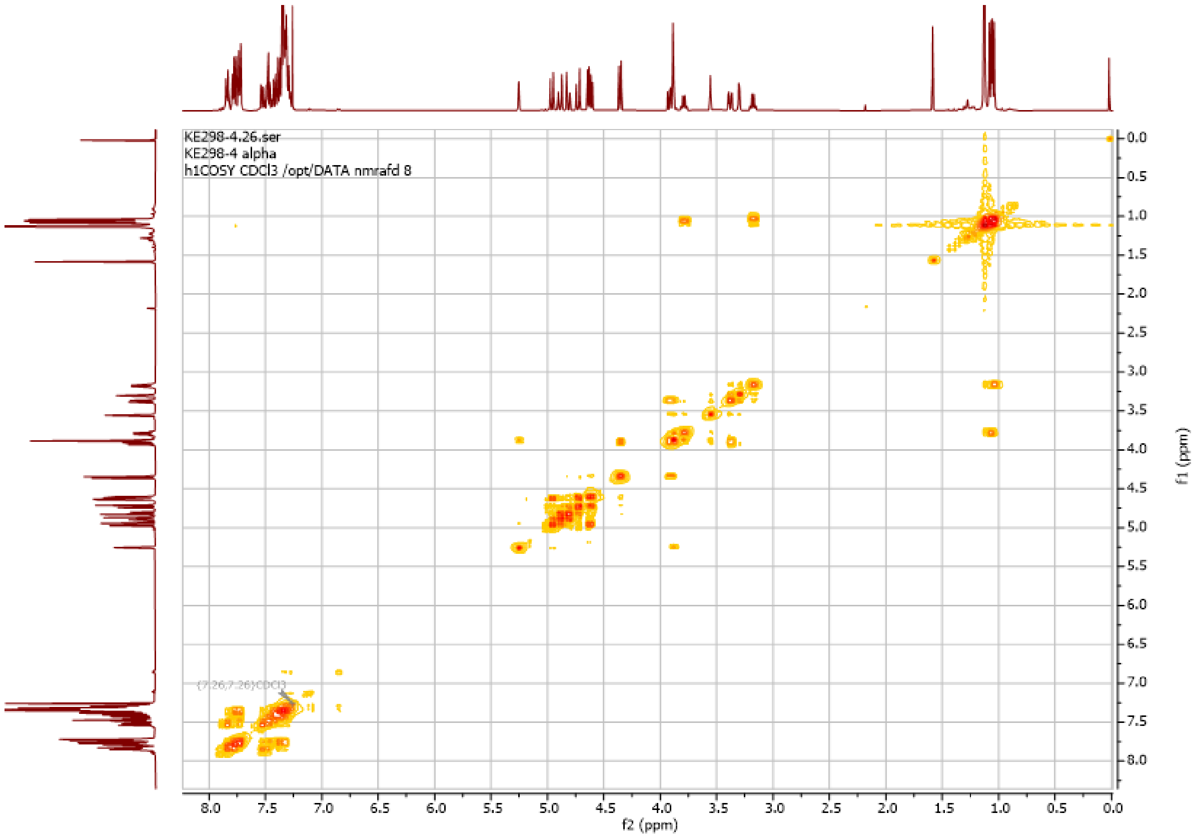

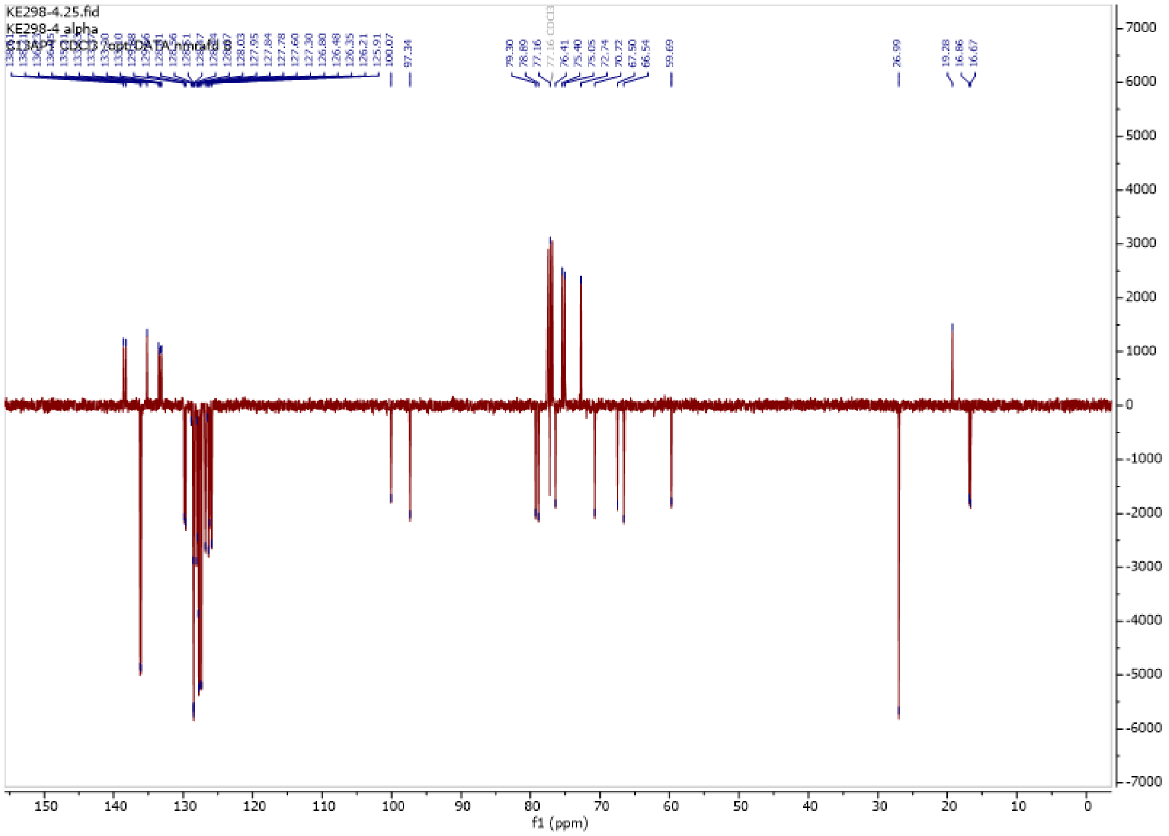
KE298-4.25.fid
KE298-4 alpha
C13APT CDCl3 /opt/DATA nmrafd 8
f1 (ppm)

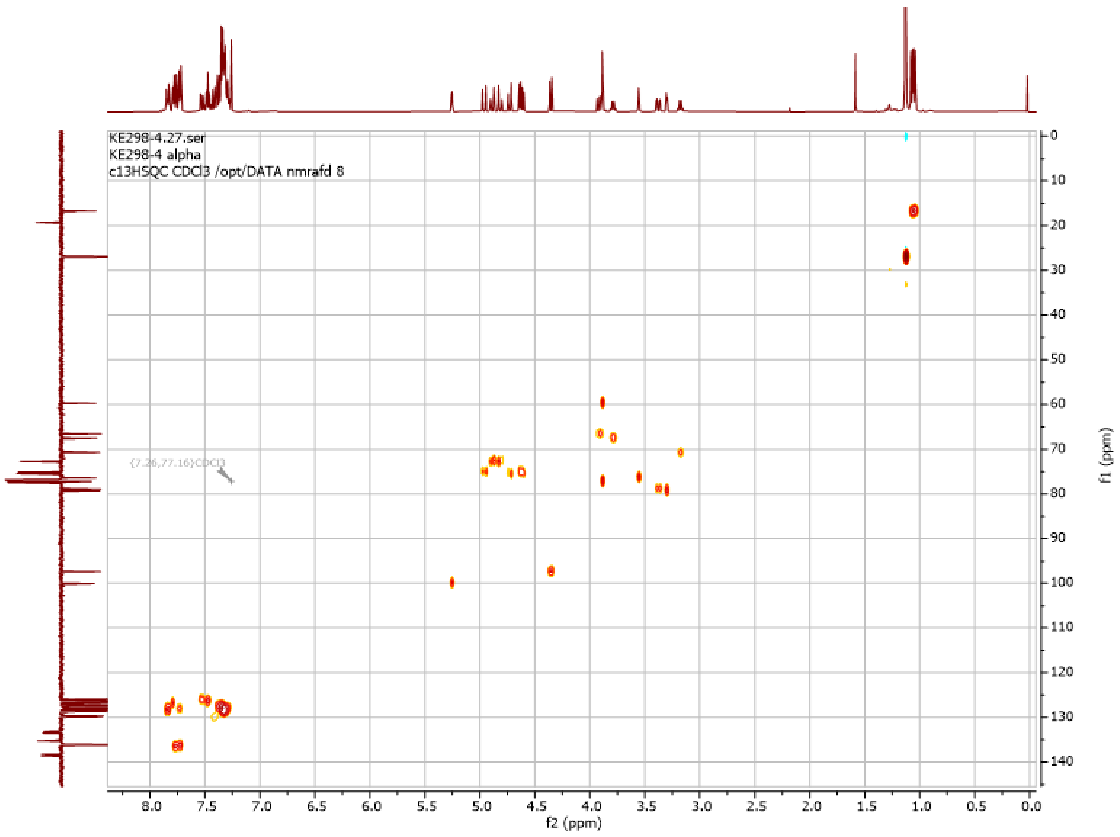
KE298-4.27.ser
KE298-4 alpha
c13HSQC CDCl3 /opt/DATA nmrafd 8
f2 (ppm)
f1 (ppm)

Tert-butyldiphenylsilyl 2-azido-4-_O_-benzyl-2-deoxy-_α_-L-fucopyranosyl-(1→3)-2-azido-4-_O_-benzyl-2-deoxy-_β_-D-fucopyranoside (14)

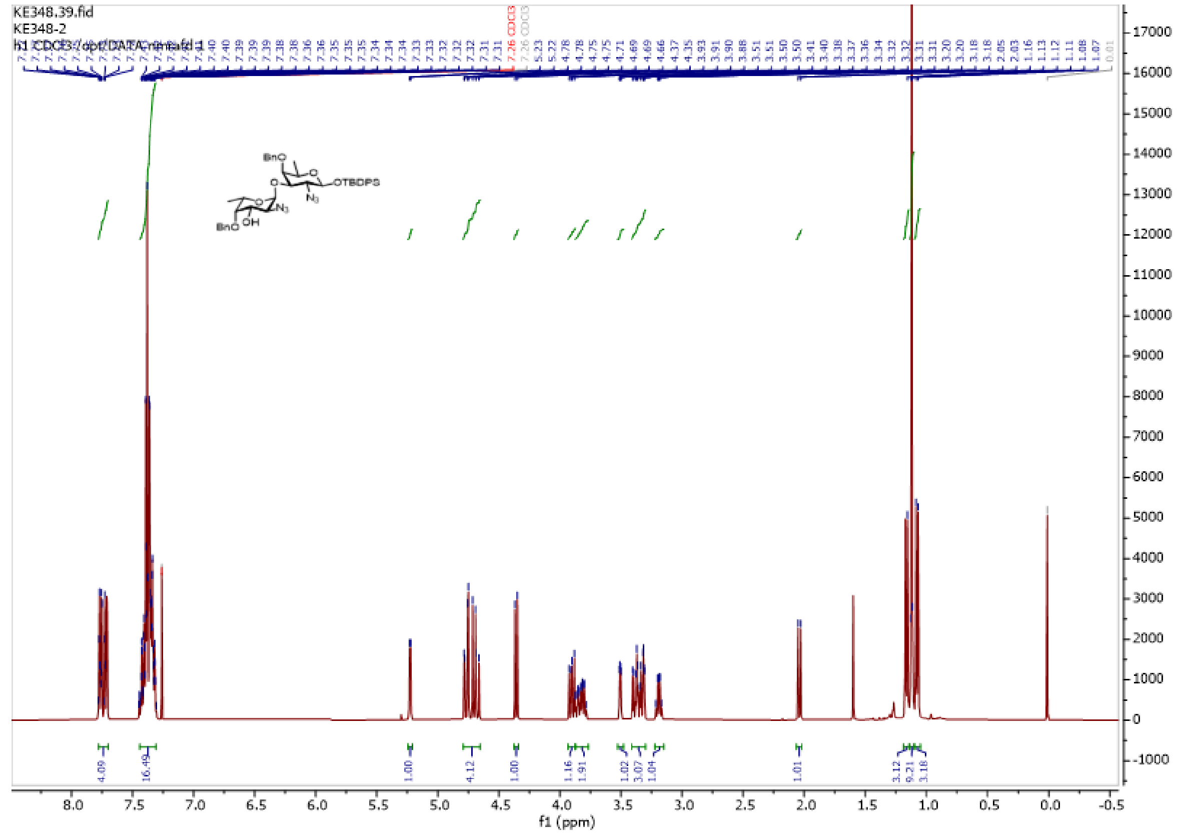

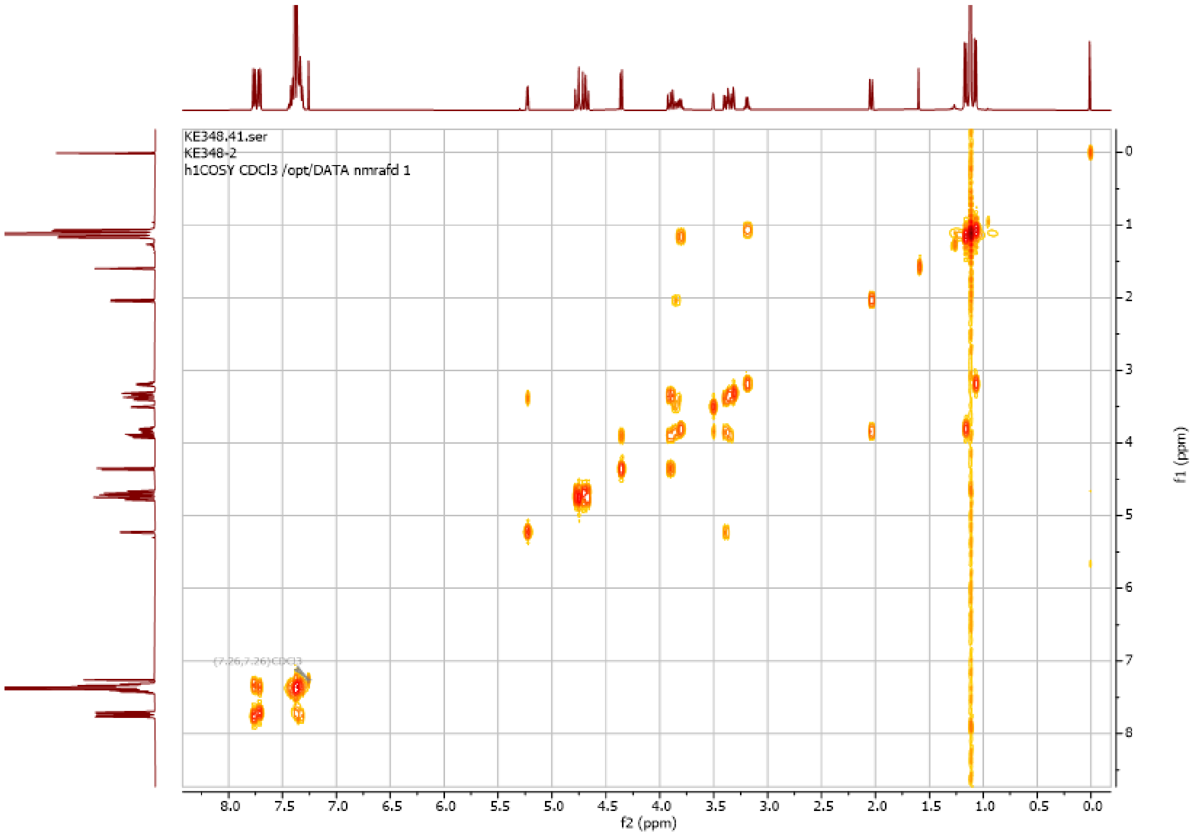

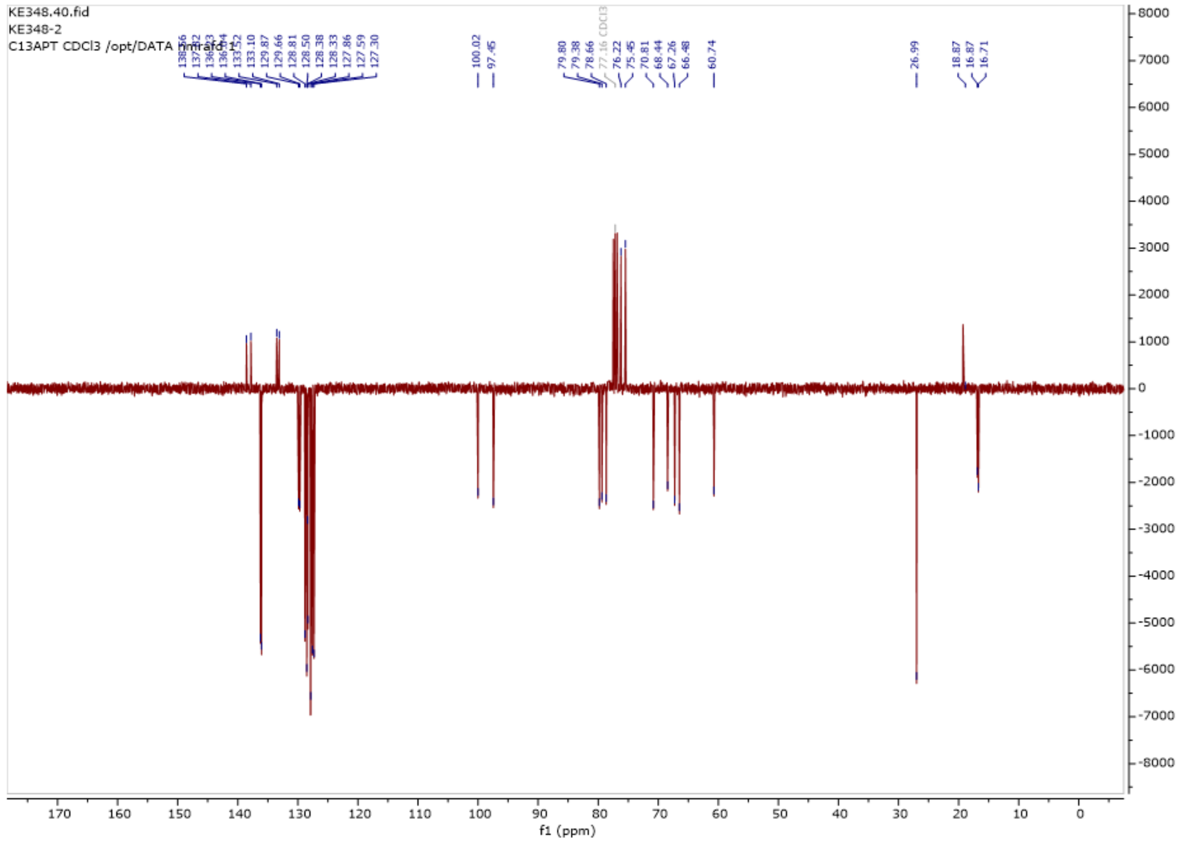
KE348.40.fid
KE348-2
C13APT CDCl3 /opt/DATA nmrafd 1
100.02
97.45
79.80
79.38
78.66
77.16 CDCl3
76.22
75.45
70.81
68.44
67.26
66.48
60.74
26.99
18.87
16.87
16.71
f1 (ppm)

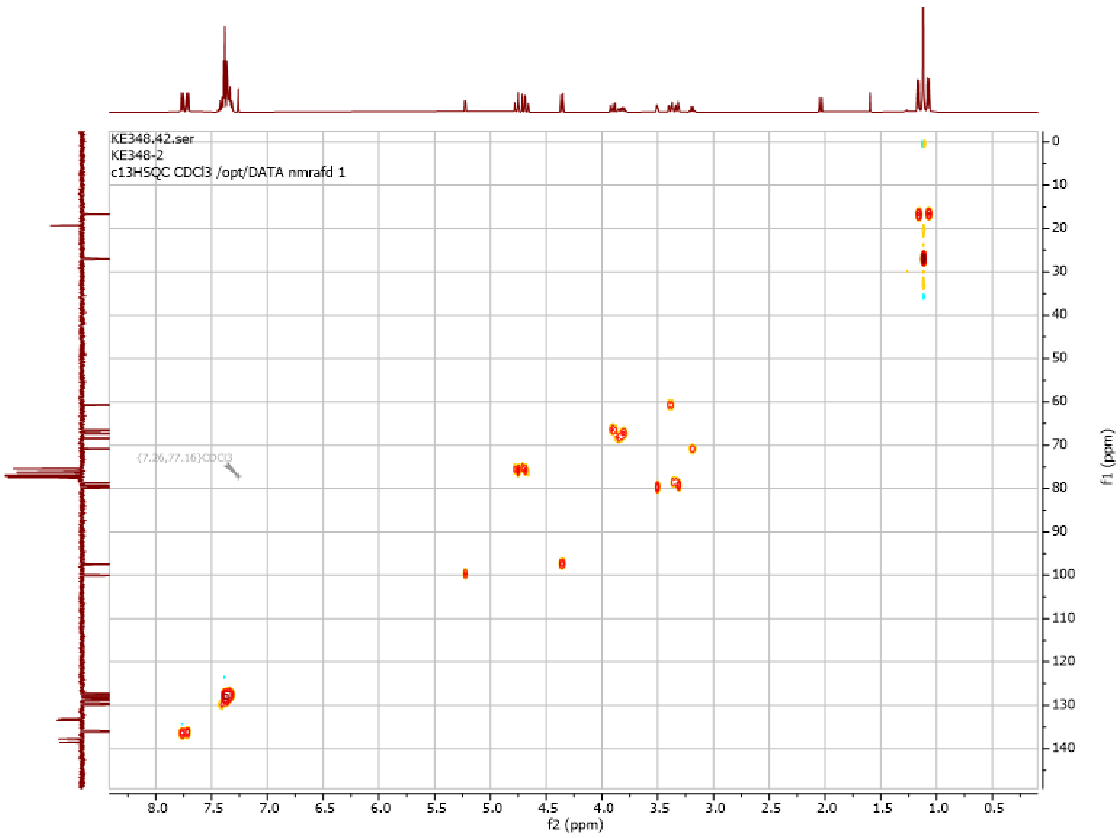
KE348.42.ser
KE348-2
c13HSQC CDCl3 /opt/DATA nmrafd 1
f2 (ppm)
f1 (ppm)

***Tert*-butyldiphenylsilyl (Benzyl (4-*O*-acetyl-2-azido-2-deoxy-3-*O*-(2-naphthylmethyl)-*β*-D-mannopyranosiduronsyl)-(1→3)-2-azido-4-*O*-benzyl-2-deoxy-*α*-L-fucopyranosyl-(1→3)-2-azido-4-*O*-benzyl-2-deoxy-*β*-D-fucopyranoside (9)**

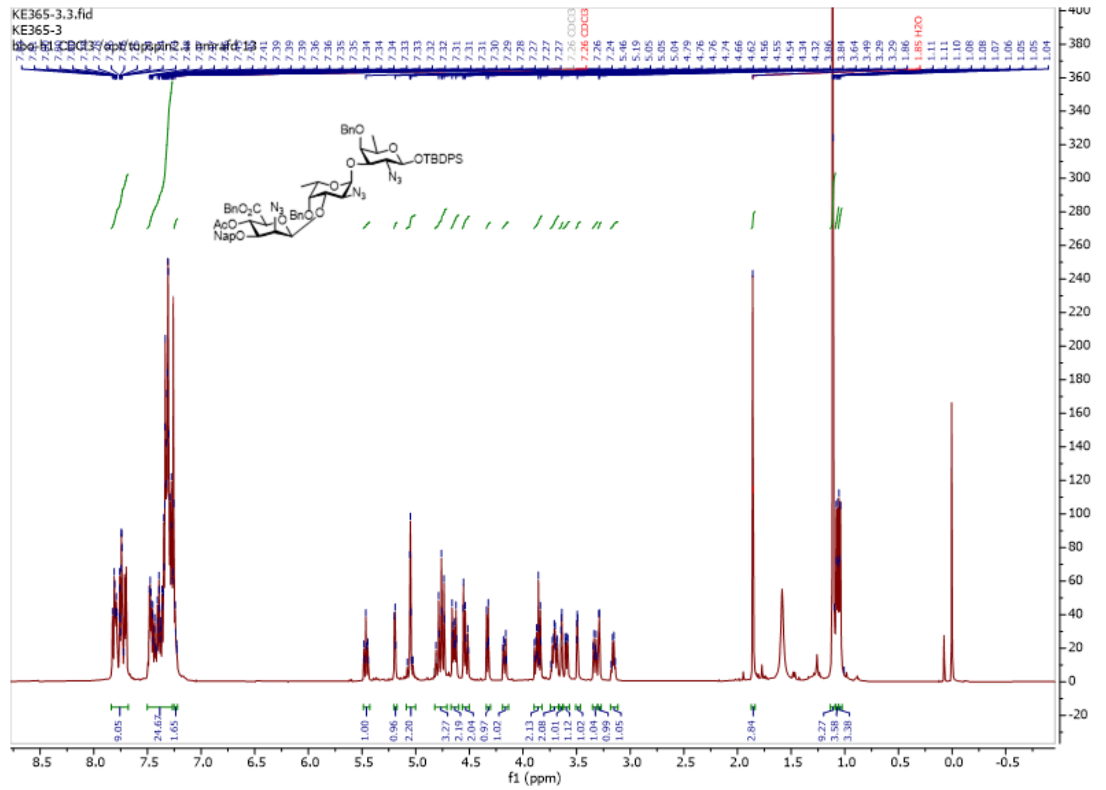

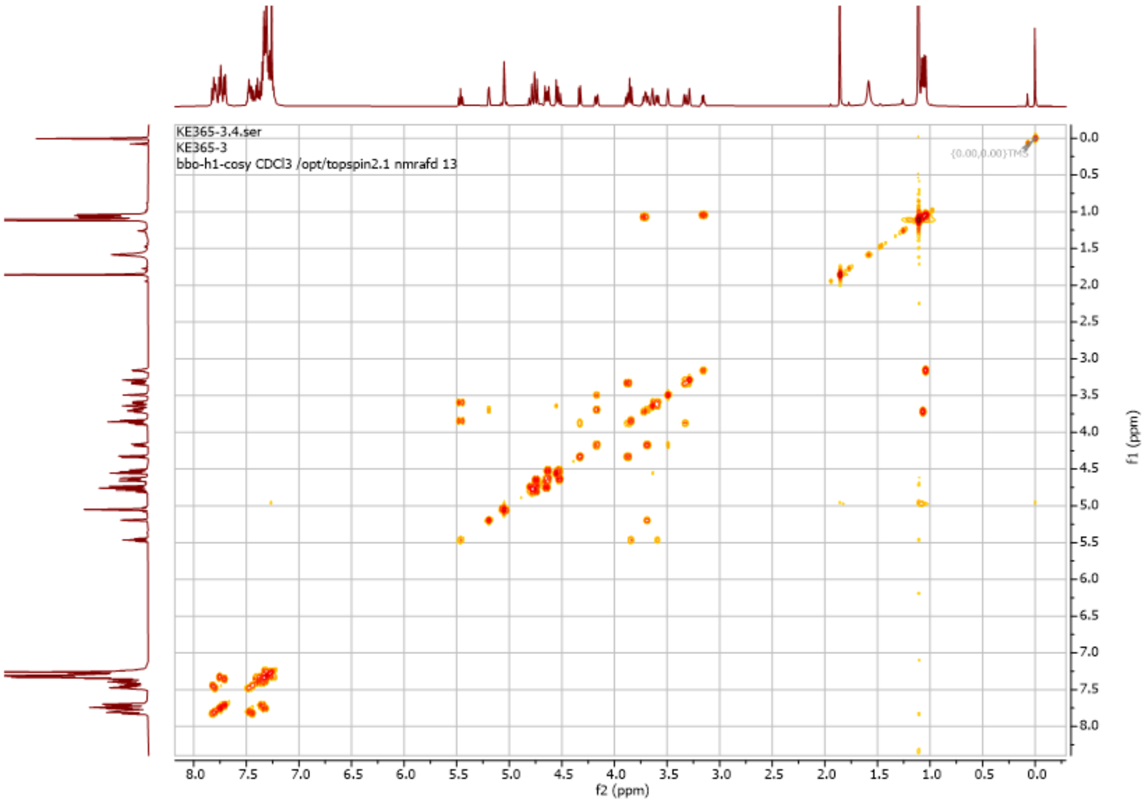

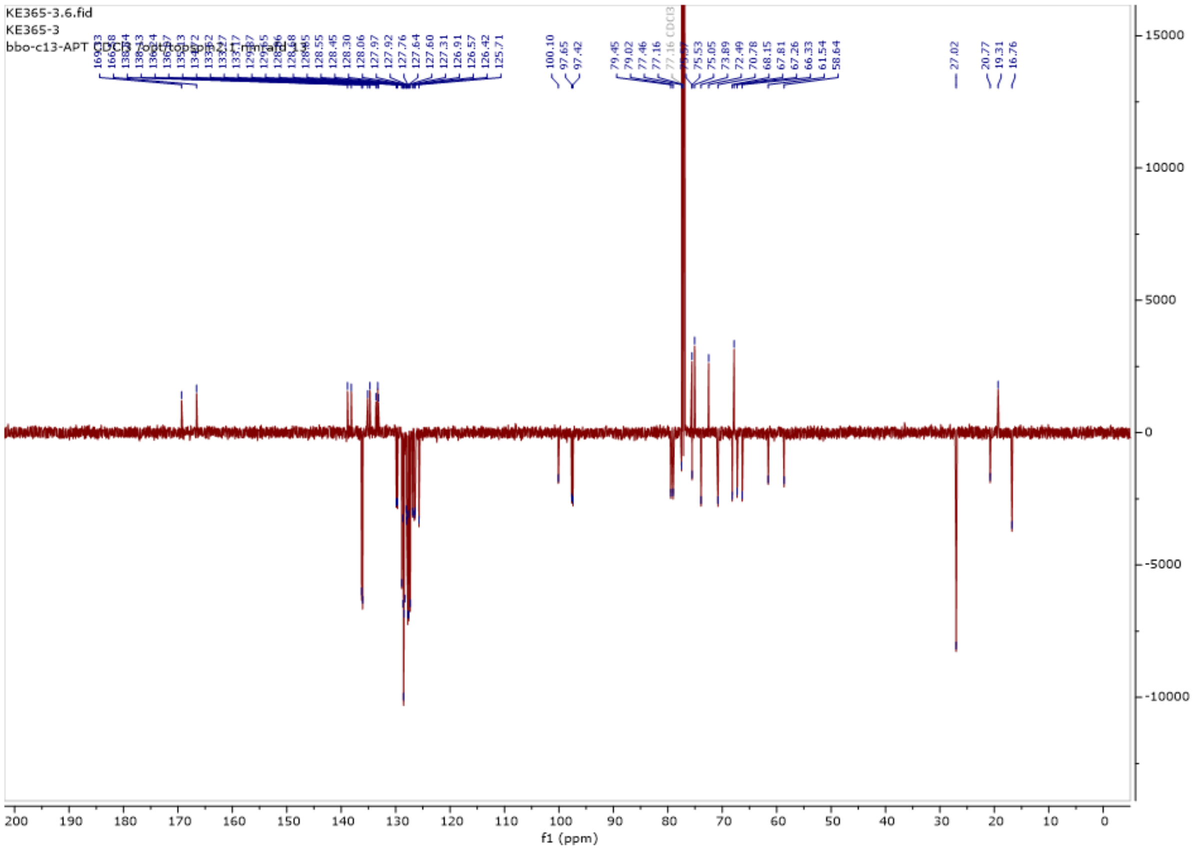
KE365-3.6.fid
KE365-3
bbo-c13-APT CDCl3 /opt/topspin2.1 nmrafd 13
f1 (ppm)

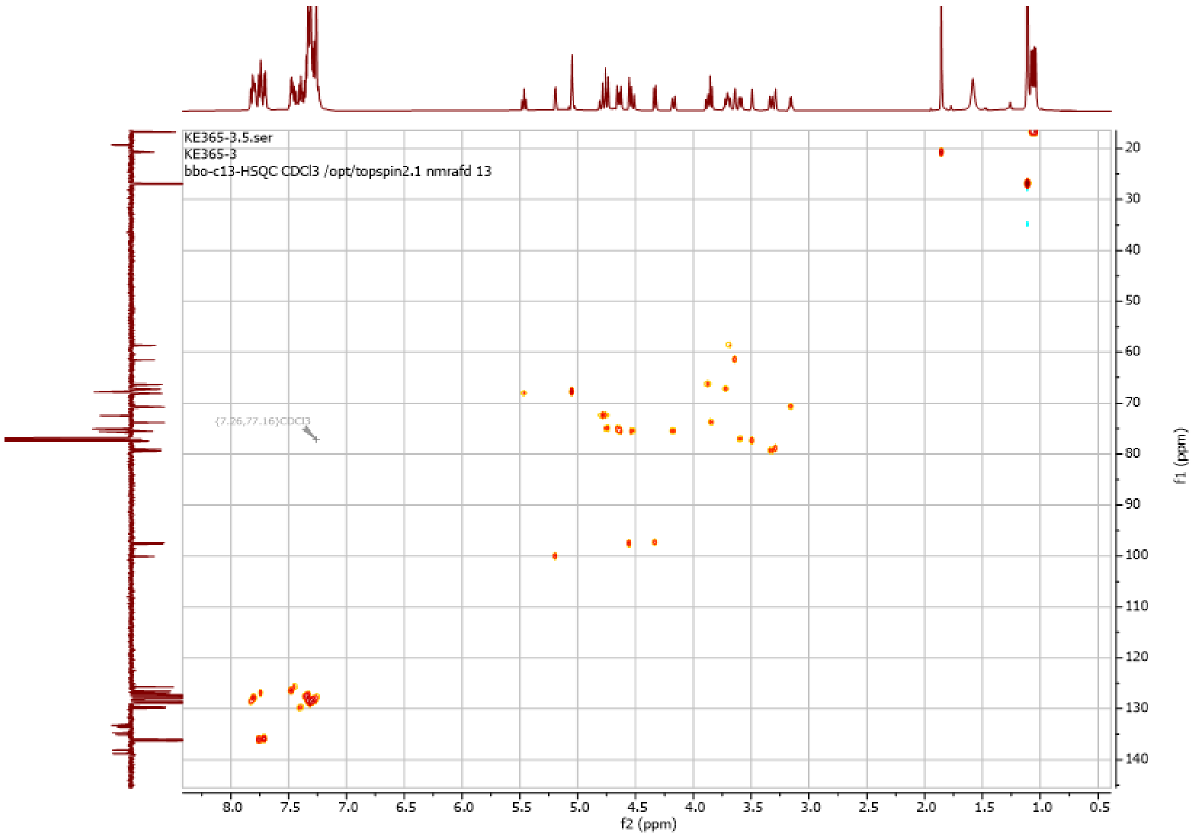
KE365-3.5.ser
KE365-3
bbo-c13-HSQC CDCl3 /opt/topspin2.1 nmrafd 13
f2 (ppm)
f1 (ppm)

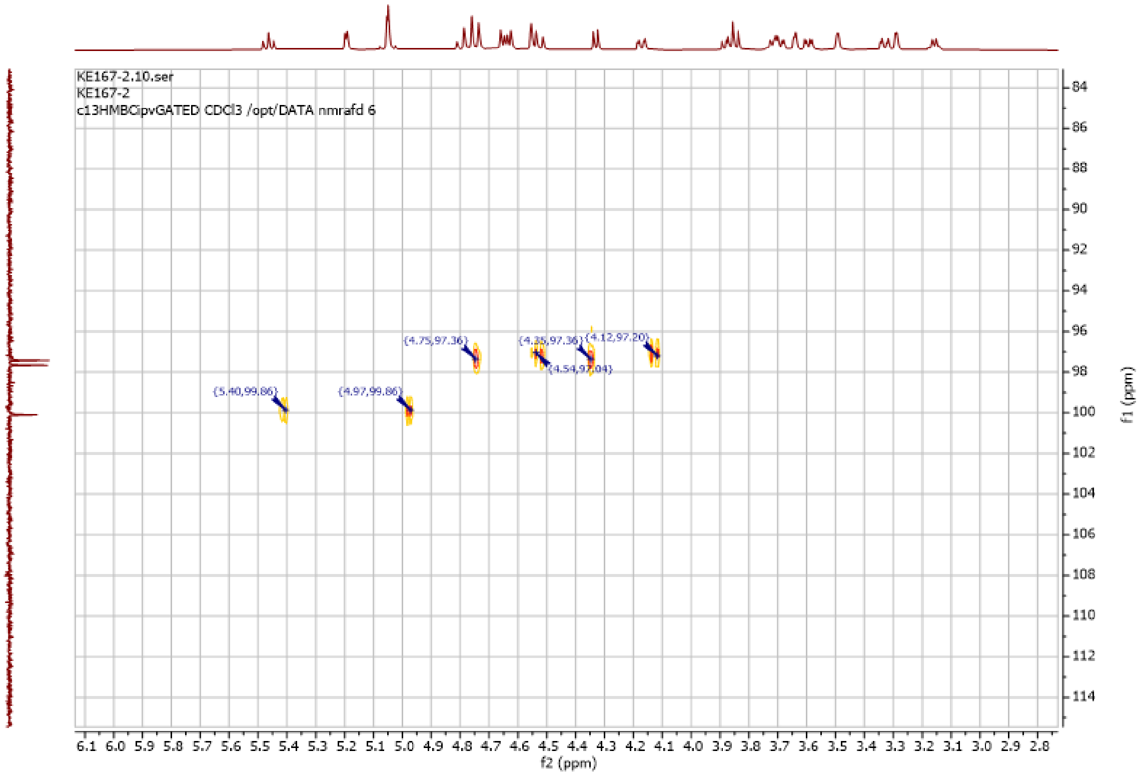

***Tert*-butyldiphenylsilyl (Benzyl (4-*O*-acetyl-2-azido-2-deoxy-3-*O*-(2-naphthylmethyl)-*α*-D-mannopyranosiduronsyl)-(1→3)-2-azido-4-*O*-benzyl-2-deoxy-*α*-L-fucopyranosyl-(1→3)-2-azido-4-*O*-benzyl-2-deoxy-*β*-D-fucopyranoside (9)**

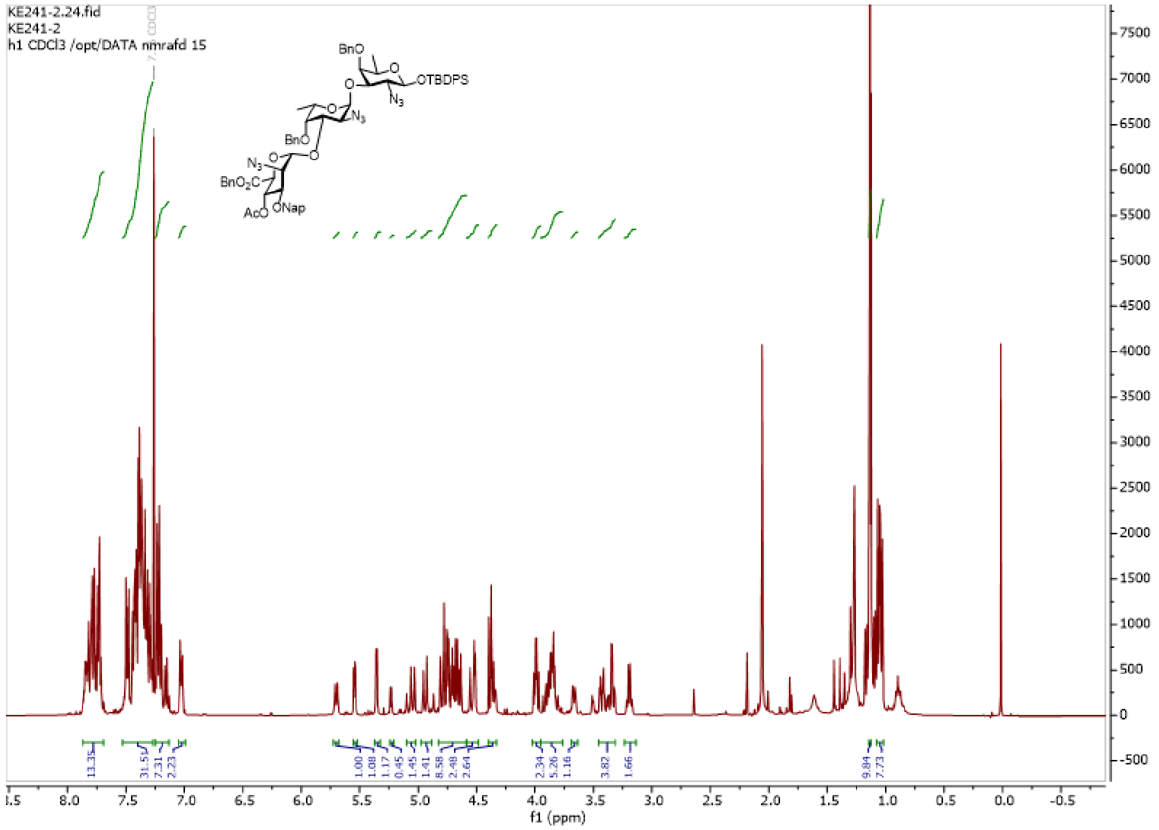

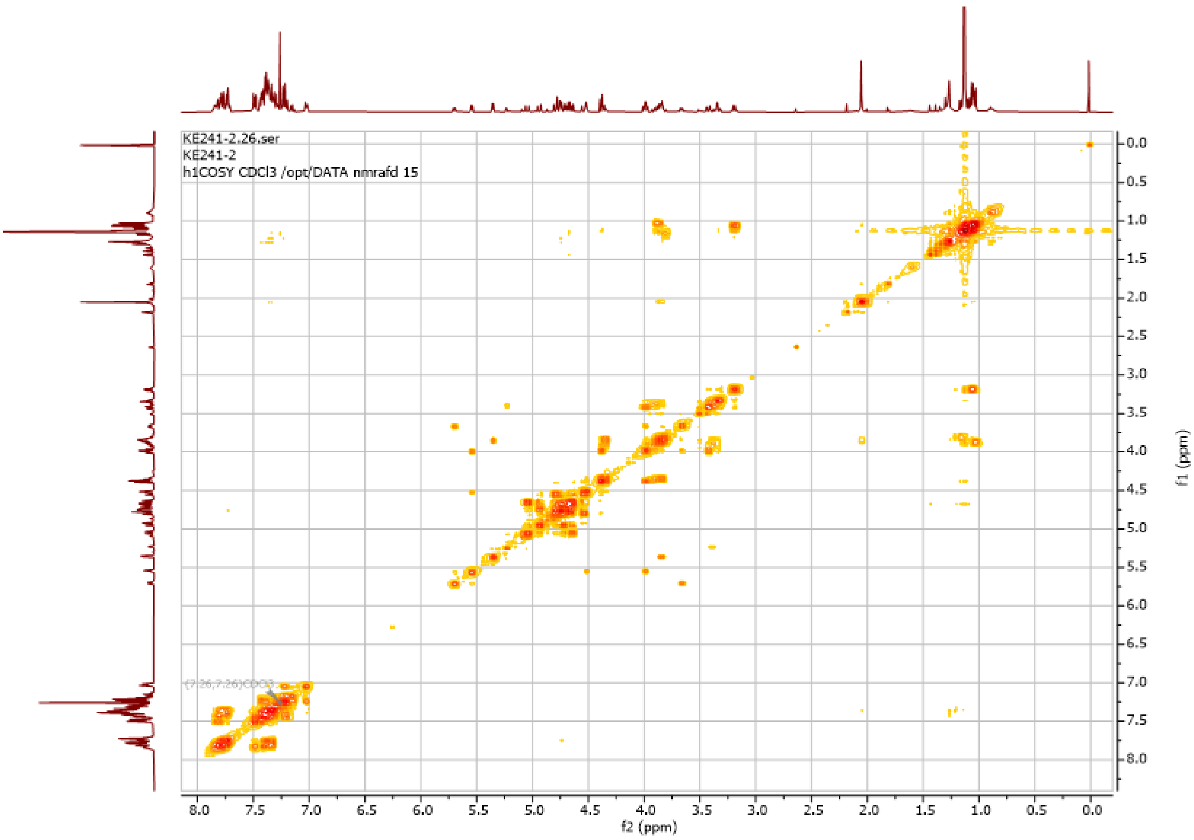
KE241-2.26.ser
KE241-2
h1COSY CDCl3 /opt/DATA nmrafd 15
f2 (ppm)
f1 (ppm)

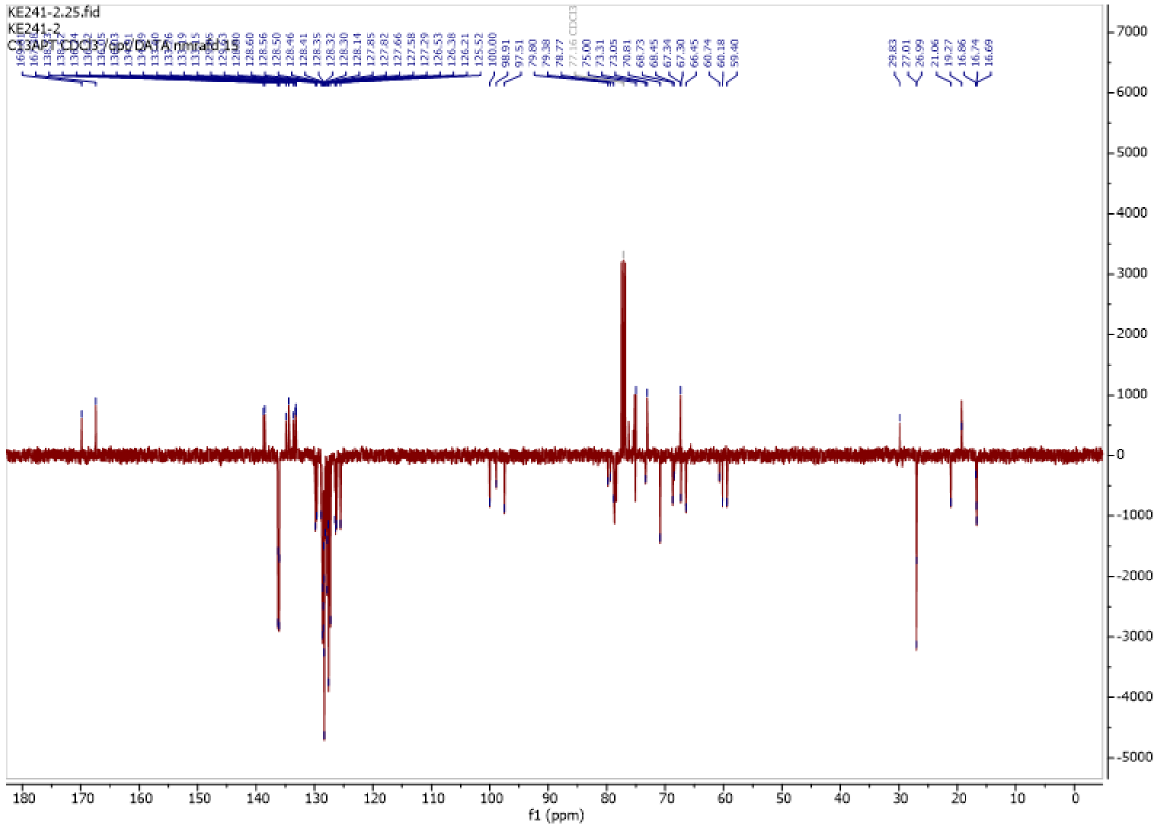
KE241-2.25.fid
KE241-2
C13APT CDCl3 /opt/DATA nmrafd 15
128.60
128.56
128.50
128.46
128.41
128.35
128.32
128.30
128.14
127.85
127.82
127.66
127.58
127.29
126.53
126.38
126.21
125.52
100.00
98.91
97.51
79.80
79.38
78.77
77.16 CDCl3
75.00
73.31
73.05
70.81
68.73
68.45
67.34
67.30
66.45
60.74
60.18
59.40
29.83
27.01
26.99
21.06
19.27
16.86
16.74
16.69
f1 (ppm)

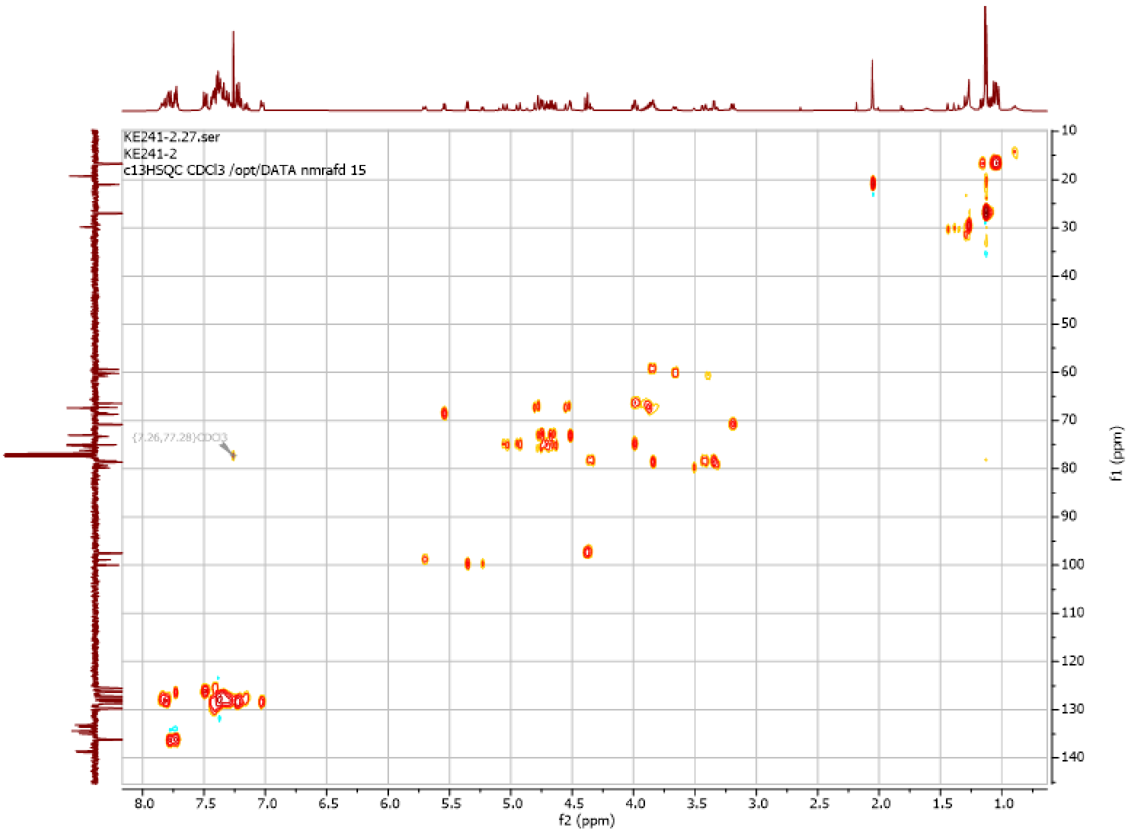

(Benzyl (4-*O*-acetyl-2-azido-2-deoxy-3-*O*-(2-naphthylmethyl)-*β*-D-mannopyranosiduronsyl))-(1→3)-2-azido-4-*O*-benzyl-2-deoxy-*α*-L-fucopyranosyl-(1→3)-2-azido-4-*O*-benzyl-2-deoxy-*α*/*β*-D-fucopyranose (15)

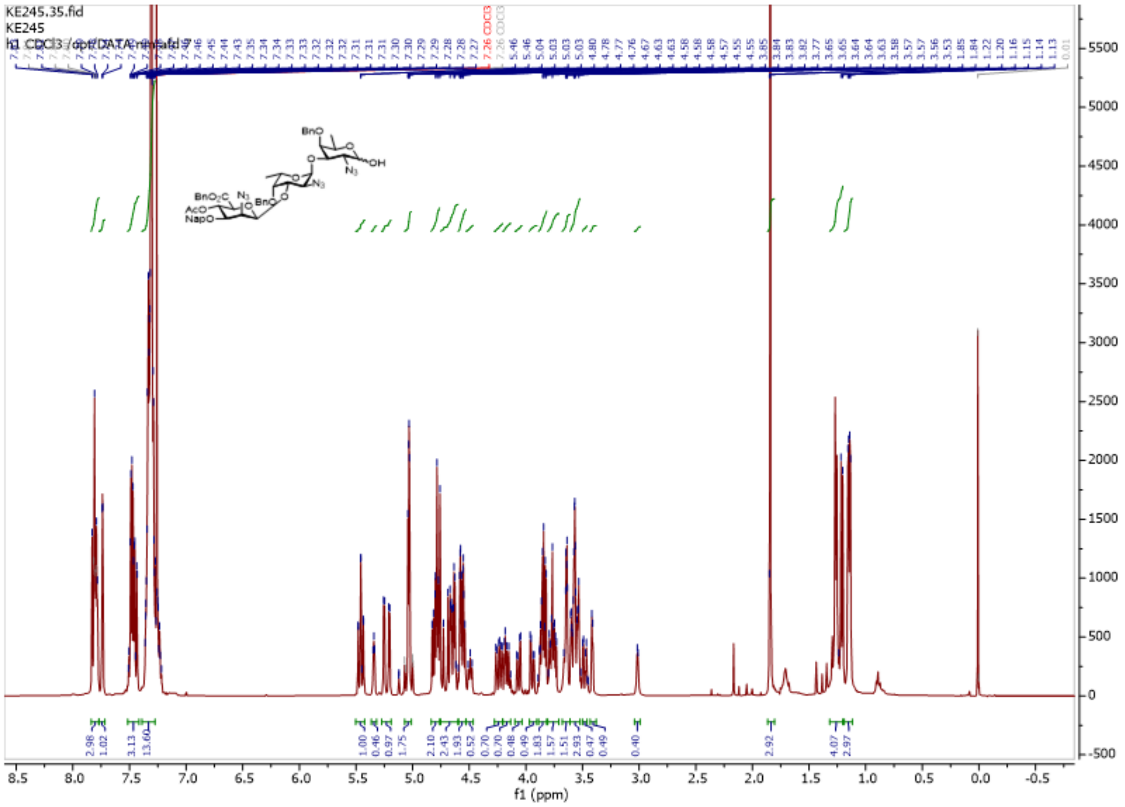

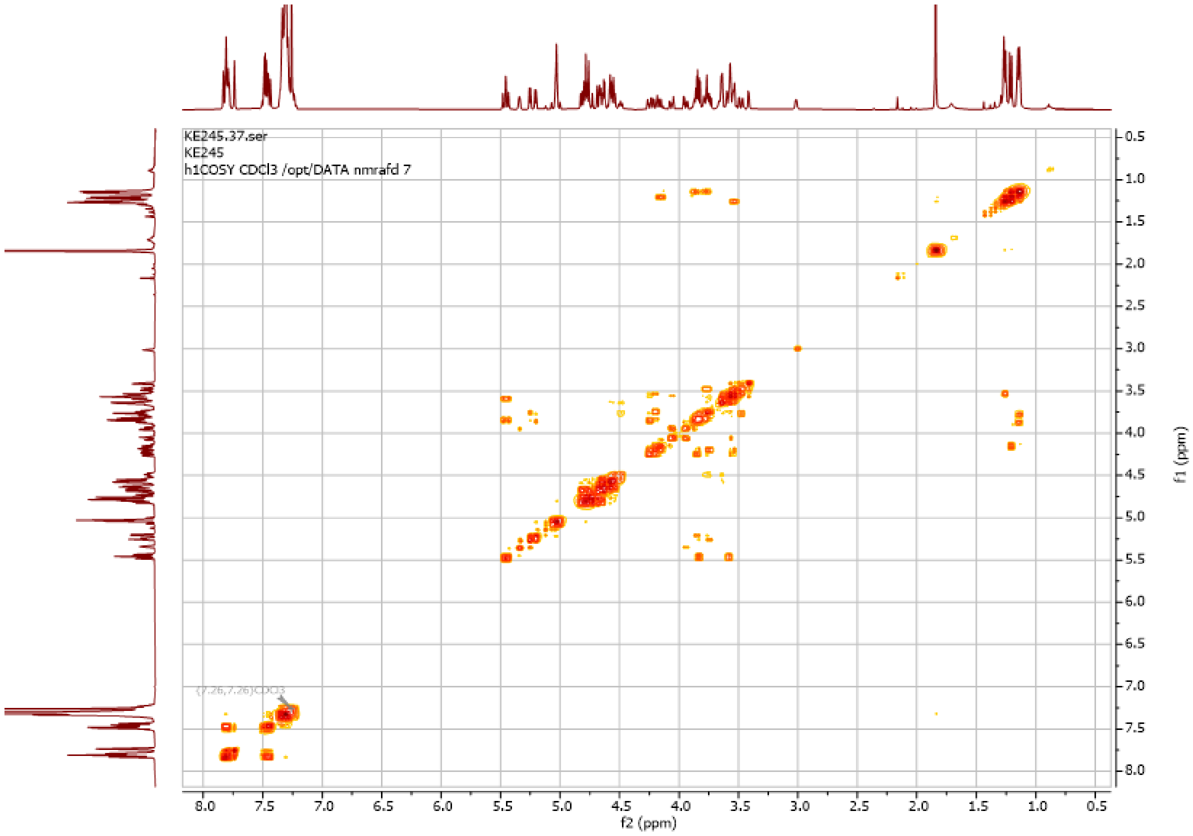
KE245.37.ser
KE245
h1COSY CDCl3 /opt/DATA nmrafd 7
f2 (ppm)
f1 (ppm)

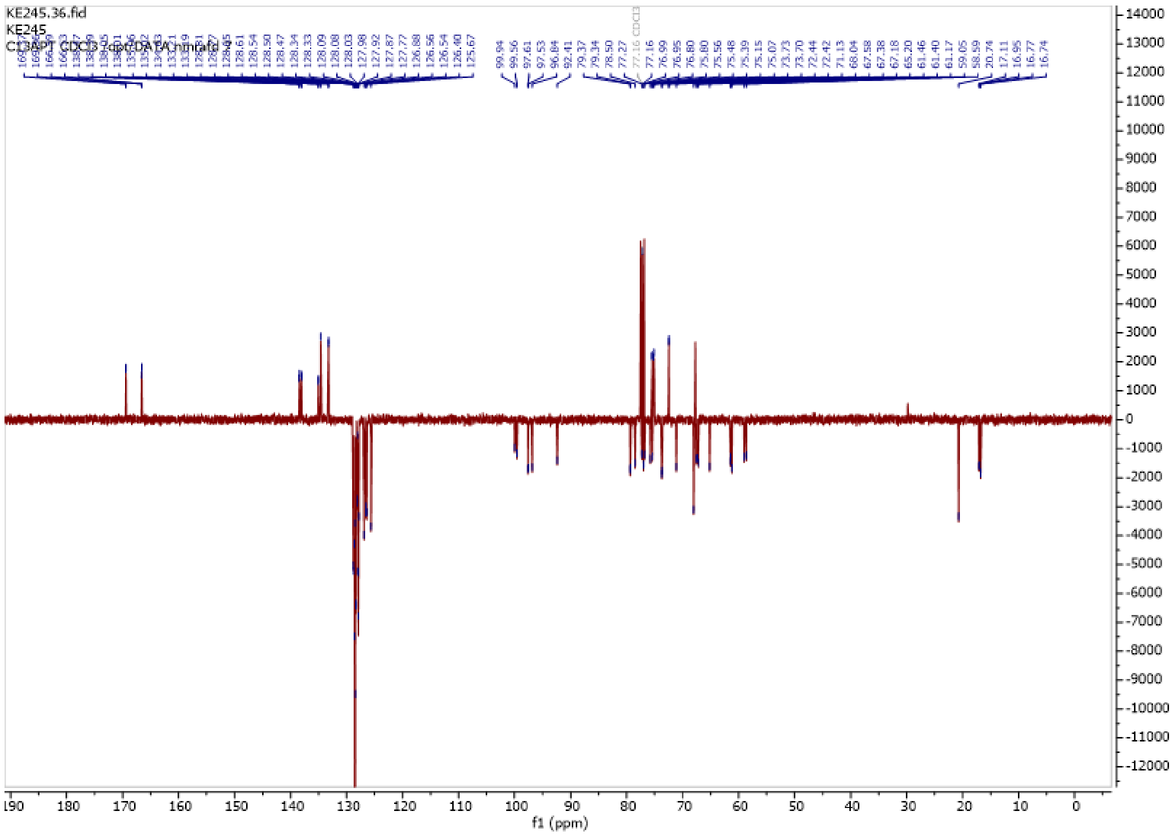
KE245.36.fid
KE245
C13APT CDCl3 /opt/DATA nmrafd
f1 (ppm)

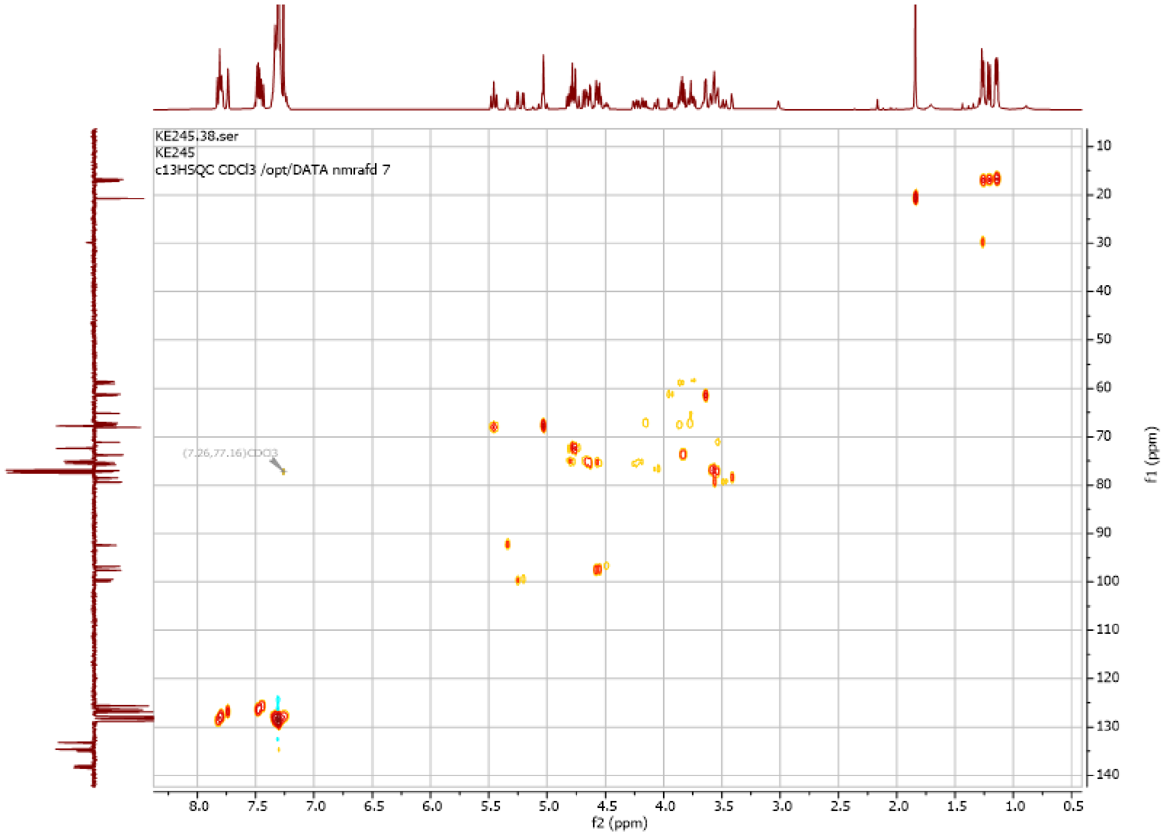

(Benzyl (4-*O*-acetyl-2-azido-2-deoxy-3-*O*-(2-naphthylmethyl)-*β*-D-mannopyranosiduronsyl))-(1→3)-2-azido-4-*O*-benzyl-2-deoxy-*α*-L-fucopyranosyl-(1→3)-2-azido-4-*O*-benzyl-2-deoxy-1-*O*-(*N*-phenyl-2,2,2-trifluoroacetimidoyl)-*α*/*β*-D-fucopyranose (16)

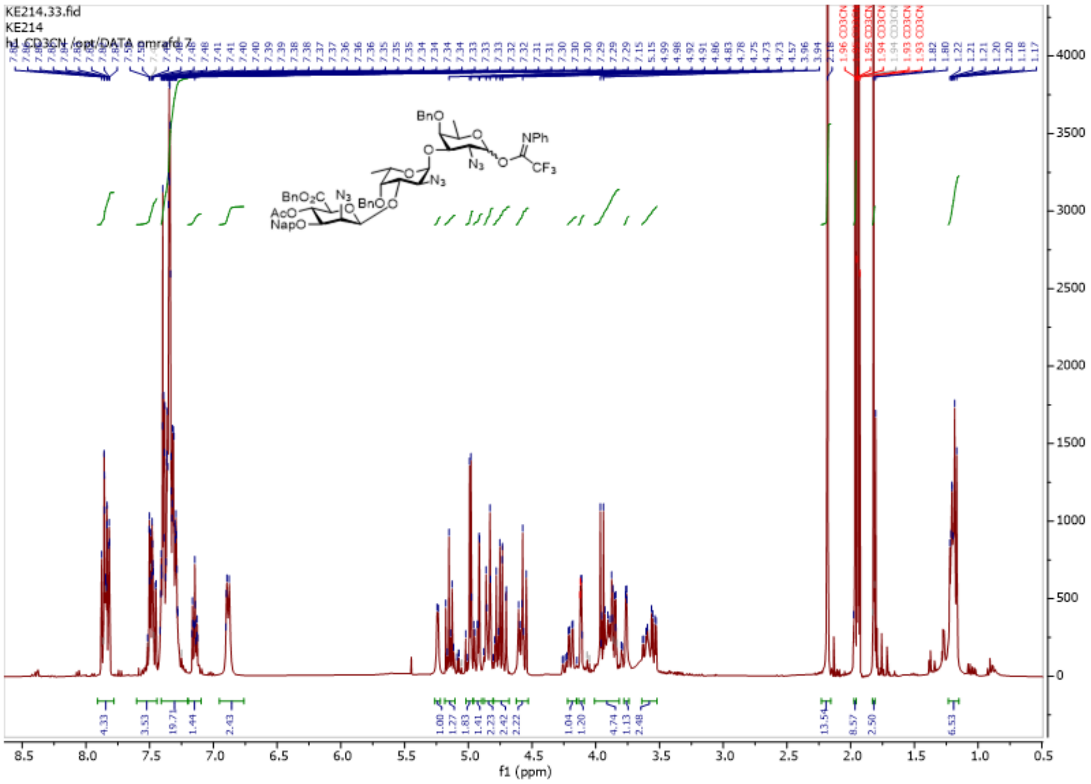

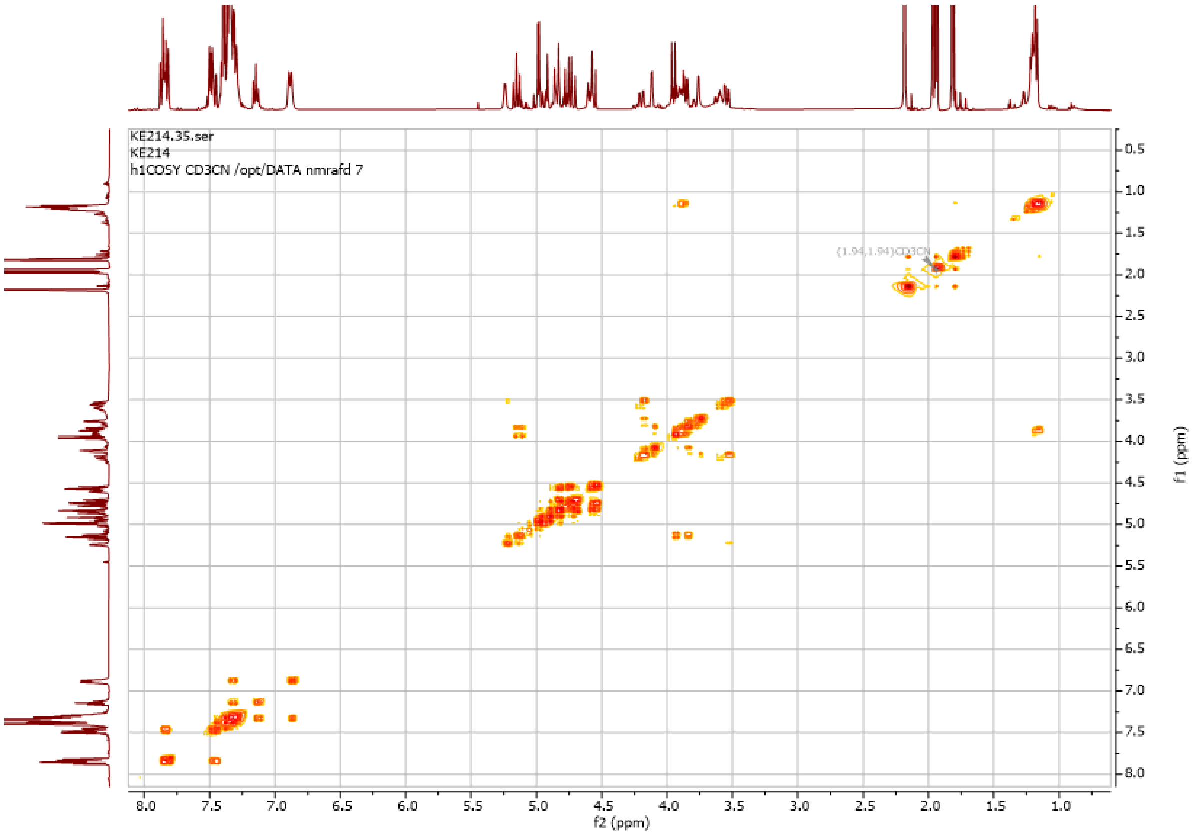
KE214.35.ser
KE214
h1COSY CD3CN /opt/DATA nmrafd 7
(1.94,1.94)CD3CN
f2 (ppm)
f1 (ppm)

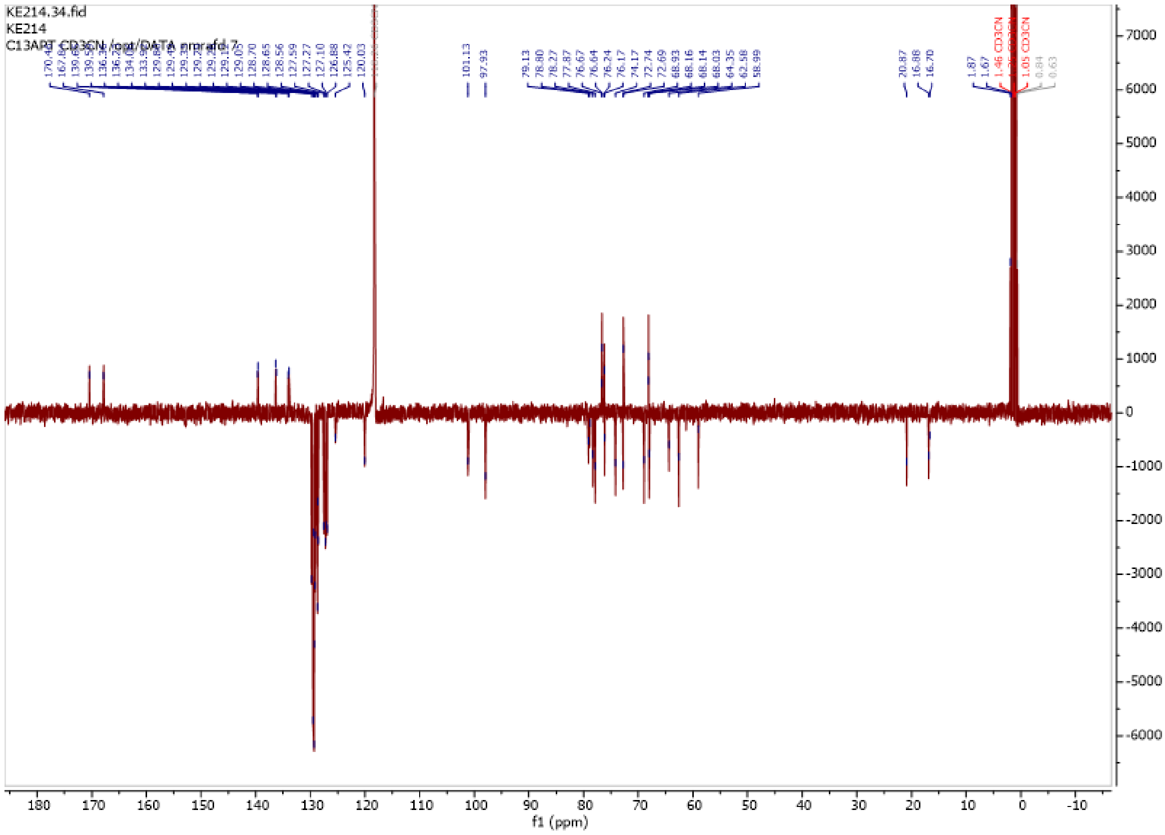
KE214.34.fid
KE214
C13APT CD3CN /opt/DATA nmrafd 7
120.03
101.13
97.93
79.13
78.80
78.27
77.87
76.67
76.64
76.24
76.17
74.17
72.74
72.69
68.93
68.16
68.14
68.03
64.35
62.58
58.99
20.87
16.88
16.70
1.87
1.67
1.46 CD3CN
1.05 CD3CN
0.84
0.63
f1 (ppm)

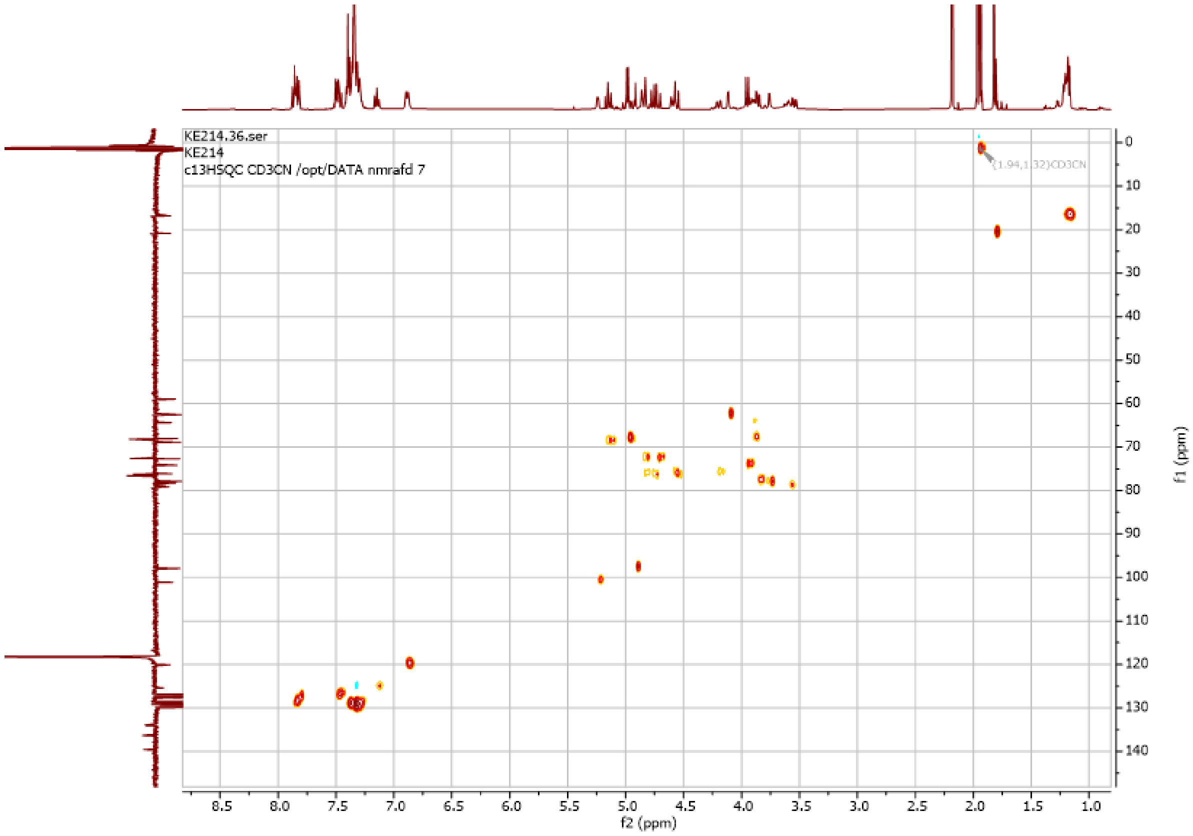

5-(Benzyl(benzyloxycarbonyl)amino)pentyl (Benzyl (4-*O*-acetyl-2-azido-2-deoxy-3-*O*-(2-naphthylmethyl)-*β*-D-mannopyranosiduronsyl)-(1→3)-2-azido-4-*O*-benzyl-2-deoxy-*α*-L-fucopyranosyl-(1→3)-2-azido-4-*O*-benzyl-2-deoxy-*α*-D-fucopyranoside (5)

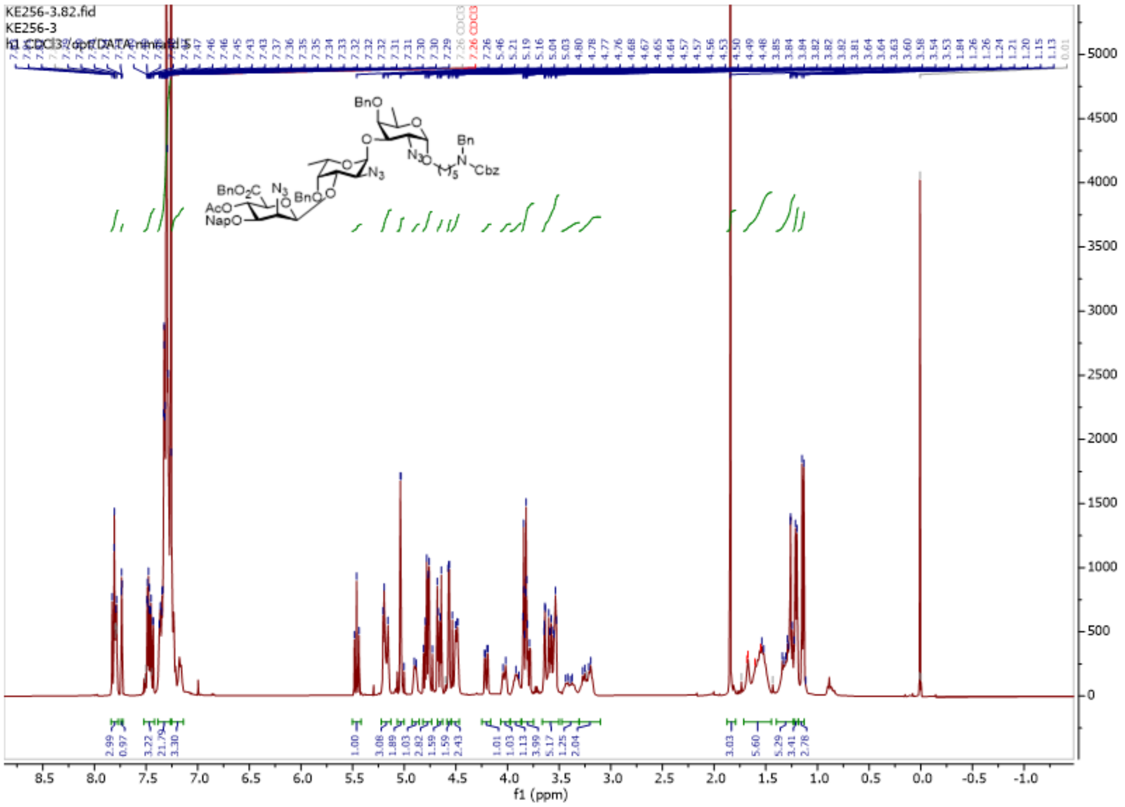

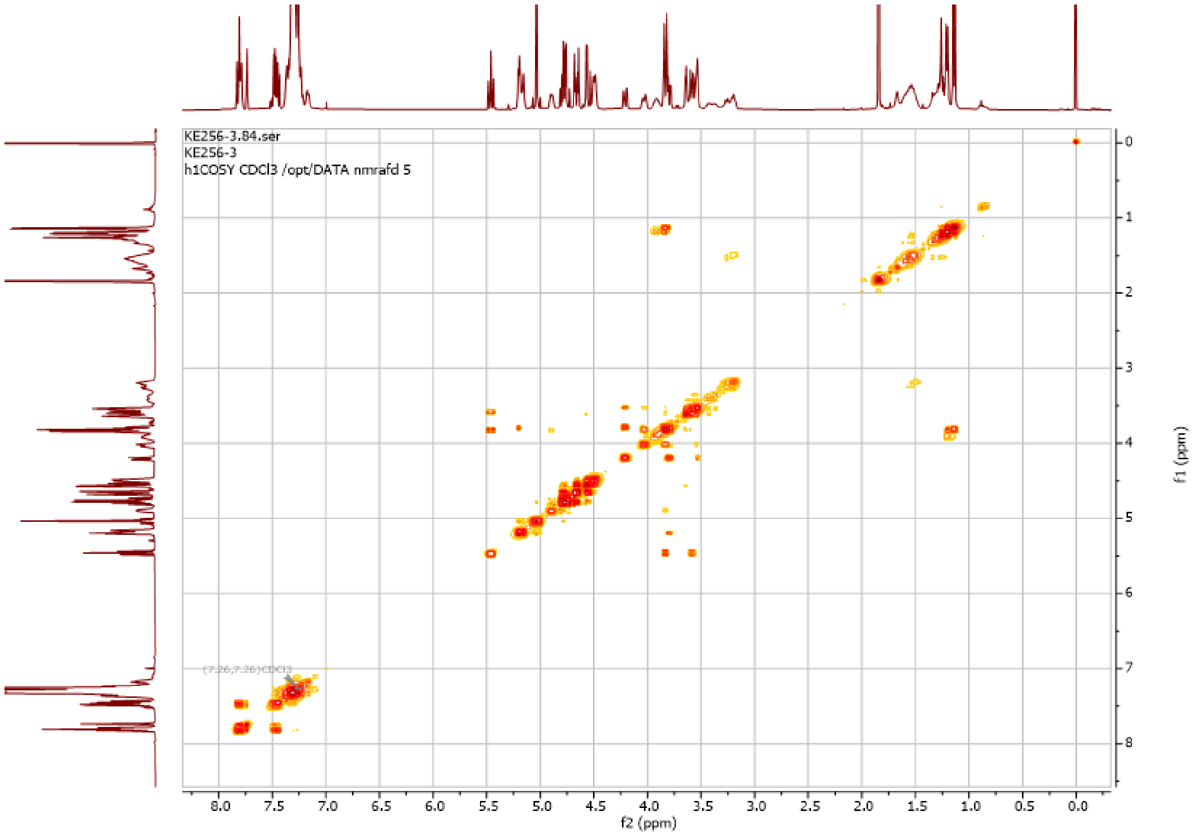
KE256-3.84.ser
KE256-3
h1COSY CDCl3 /opt/DATA nmrafd 5
f2 (ppm)
f1 (ppm)

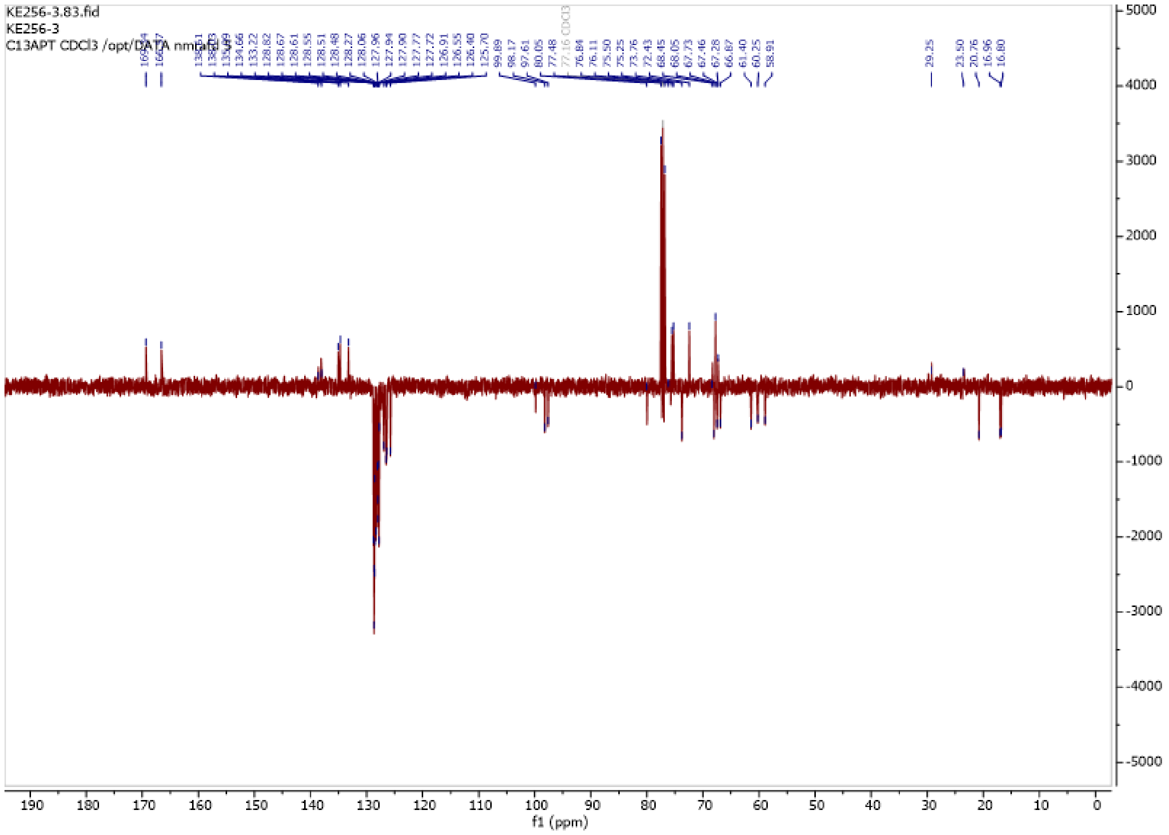
KE256-3.83.fid
KE256-3
C13APT CDCl3 /opt/DATA nmrafd 5
134.66
133.22
128.82
128.67
128.61
128.55
128.51
128.48
128.27
128.06
127.96
127.94
127.90
127.77
127.72
126.91
126.55
126.40
125.70
99.89
98.17
97.61
80.05
77.48
77.16 CDCl3
76.84
76.11
75.50
75.25
73.76
72.43
68.45
68.05
67.73
67.46
67.28
66.87
61.40
60.25
58.91
29.25
23.50
20.76
16.96
16.80
f1 (ppm)

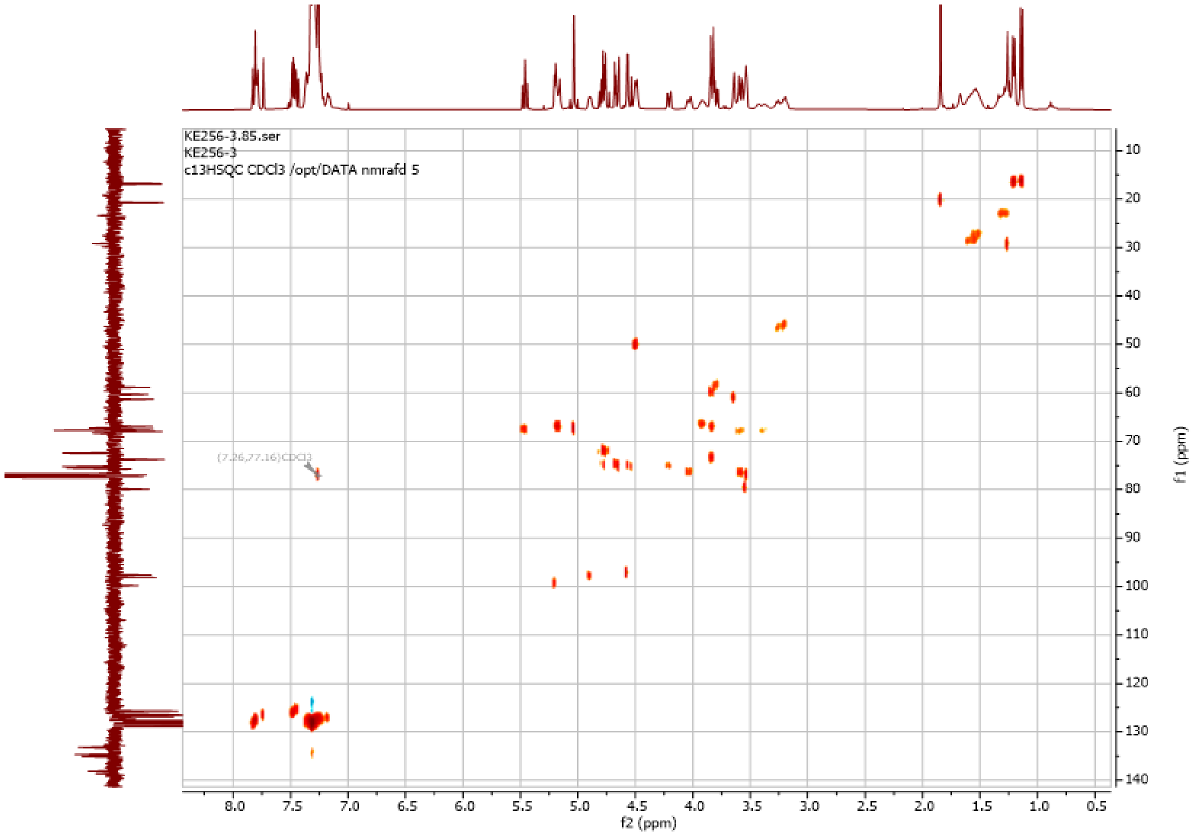
KE256-3.85.ser
KE256-3
c13HSQC CDCl3 /opt/DATA nmrafd 5
{7.26,77.16}CDCl3
f2 (ppm)
f1 (ppm)

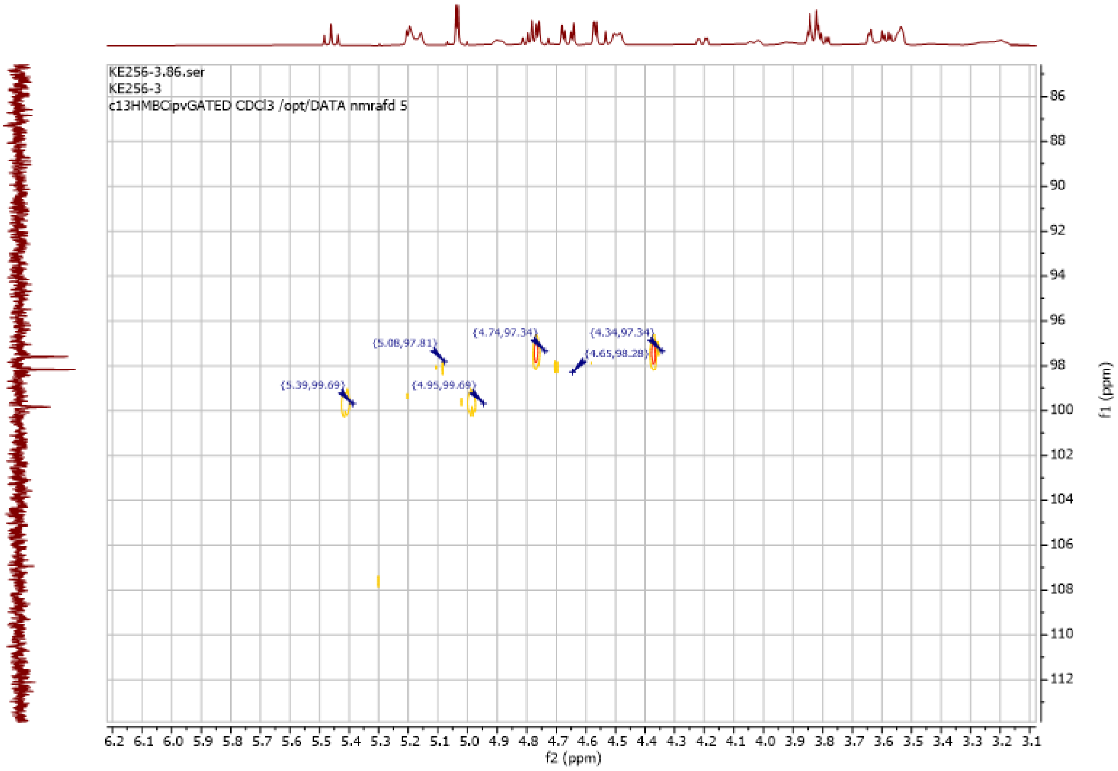
KE256-3.86.ser
KE256-3
c13HMBCipvGATED CDCl3 /opt/DATA nmrafd 5
{5.39,99.69}
{5.08,97.81}
{4.95,99.69}
{4.74,97.34}
{4.65,98.28}
{4.34,97.34}
f2 (ppm)
f1 (ppm)

5-(Benzyl(benzyloxycarbonyl)amino)pentyl (Benzyl (4-*O*-acetyl-2-azido-2-deoxy-3-*O*-(2-naphthylmethyl)-*β*-D-mannopyranosiduronsyl)-(1→3)-2-azido-4-*O*-benzyl-2-deoxy-*β*-L-fucopyranosyl-(1→3)-2-azido-4-*O*-benzyl-2-deoxy-*α*-D-fucopyranoside (5)

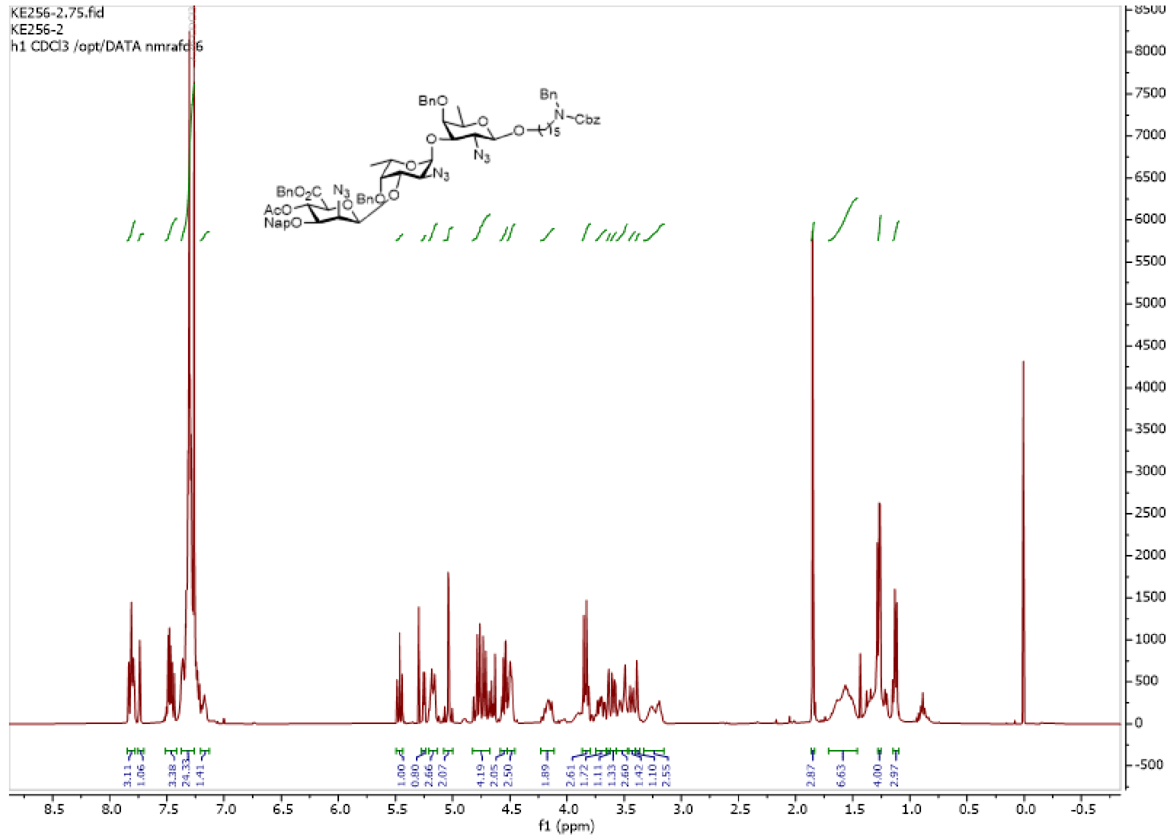

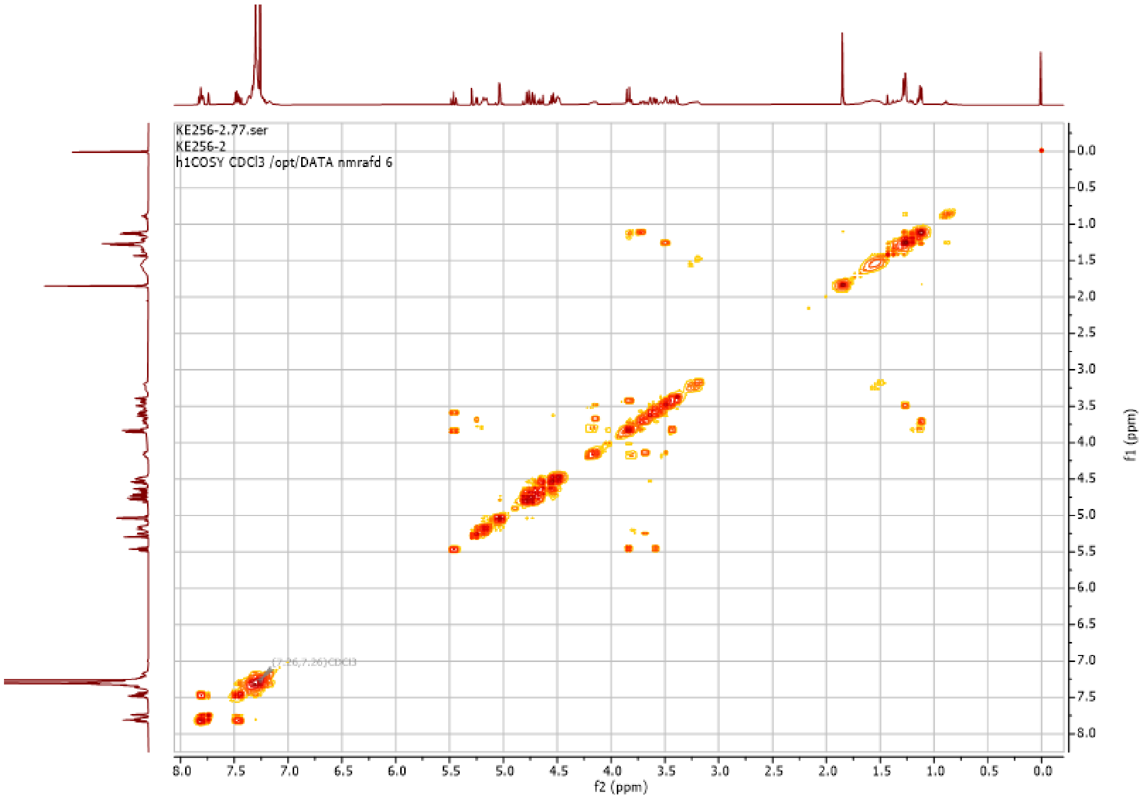

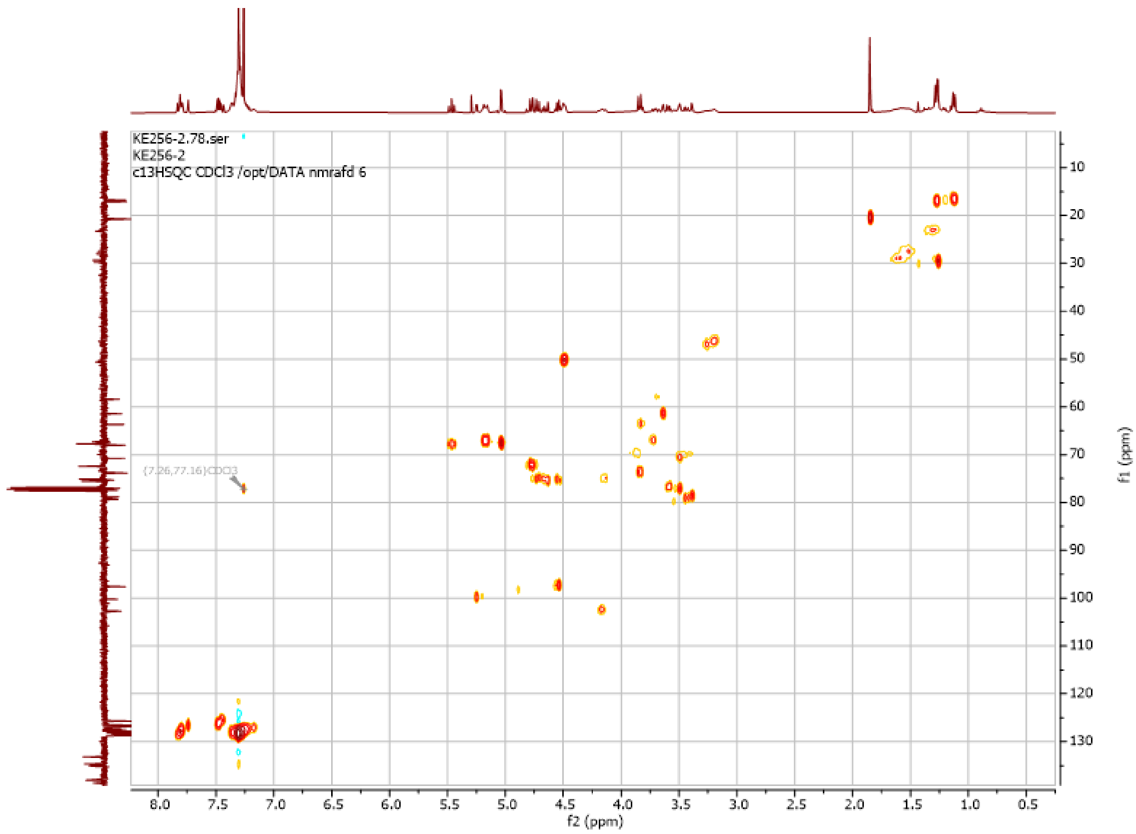
KE256-2.78.ser
KE256-2
c13HSQC CDCl3 /opt/DATA nmrafd 6
f2 (ppm)
f1 (ppm)

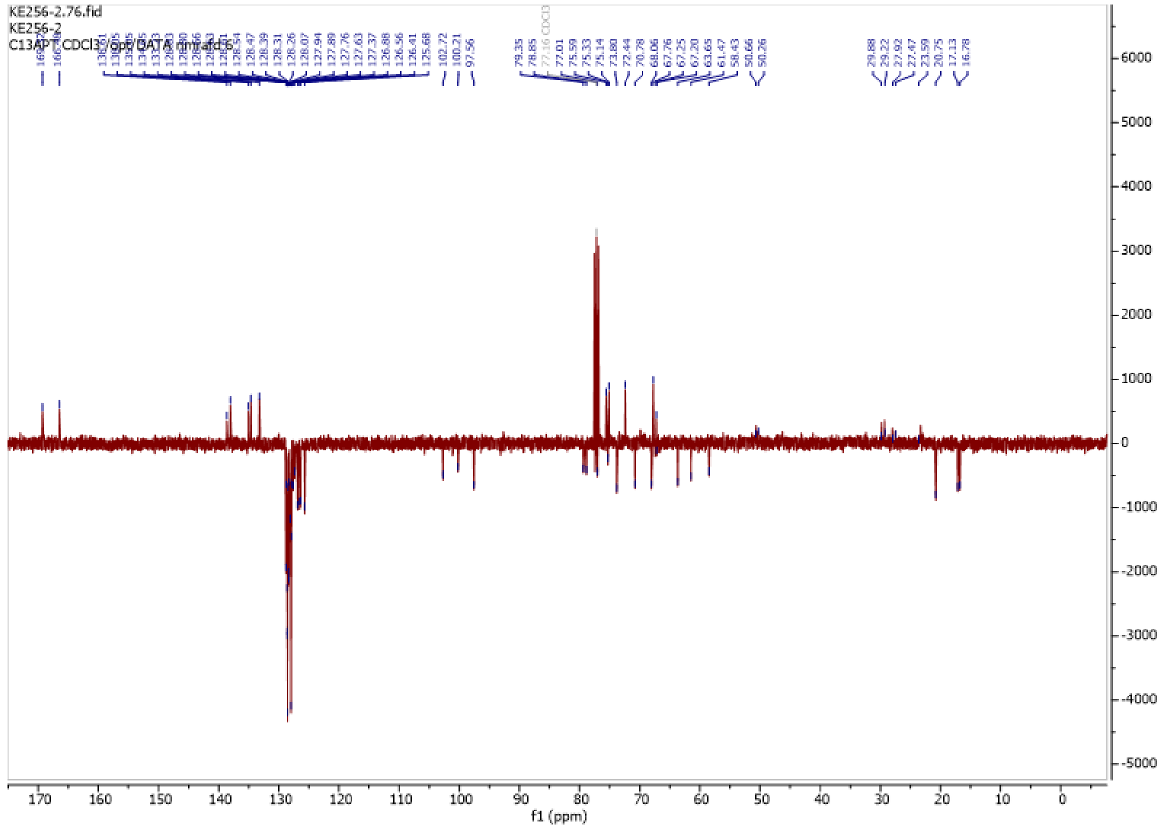
KE256-2.76.fid
KE256-2
C13APT CDCl3 /opt/DATA nmrafd 6
f1 (ppm)

5-aminopentyl 2-acetamide-4-*O*-acetyl-2-deoxy-*β*-D-mannopyranosiduronsyl-(1→3)-2-acetamide-2-deoxy-*α*-L-fucopyranosyl-(1→3)-2-acetamide-2-deoxy-*α*-D-fucopyranoside (1)

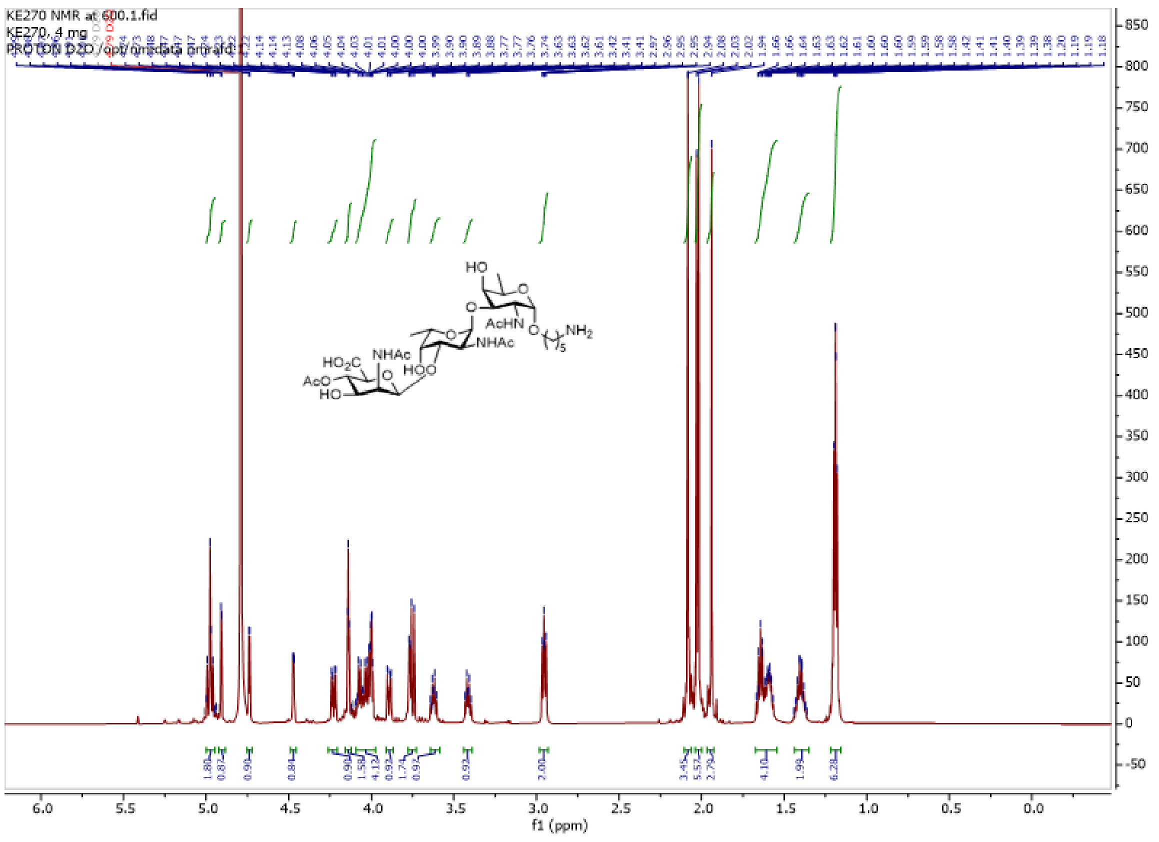

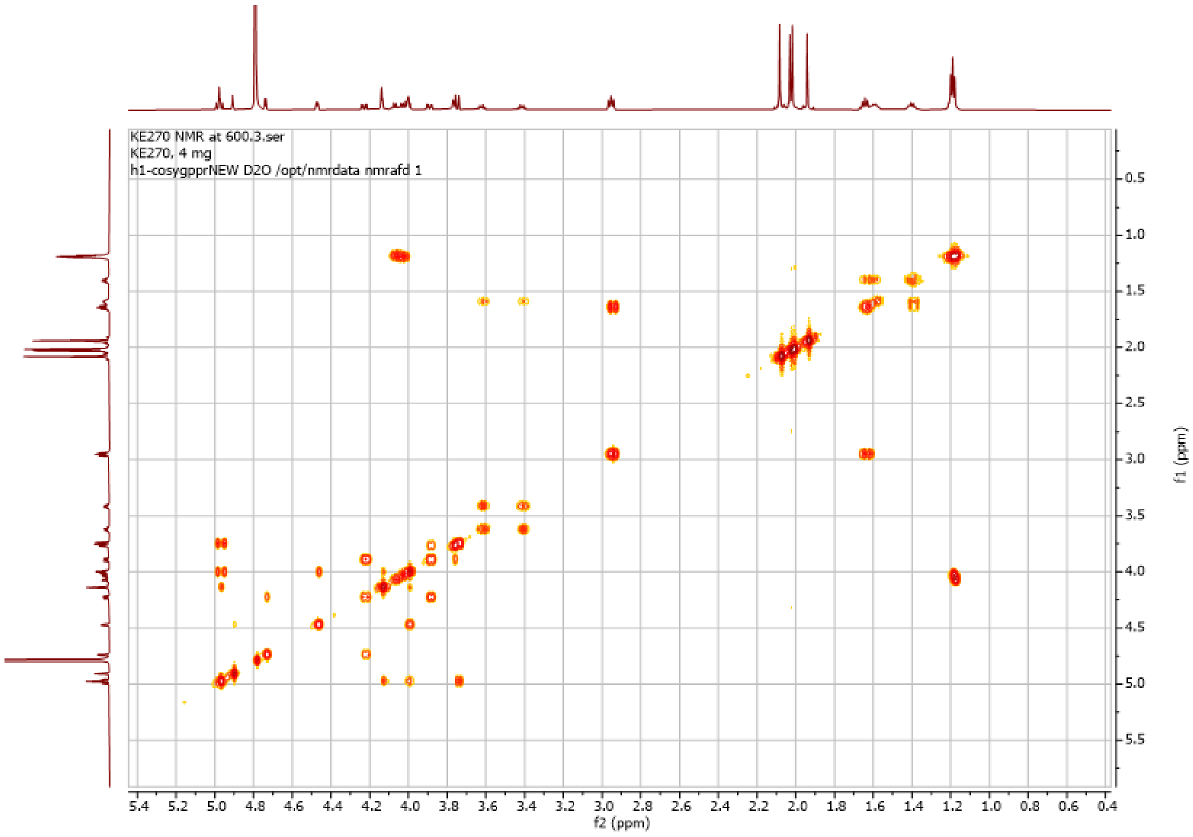

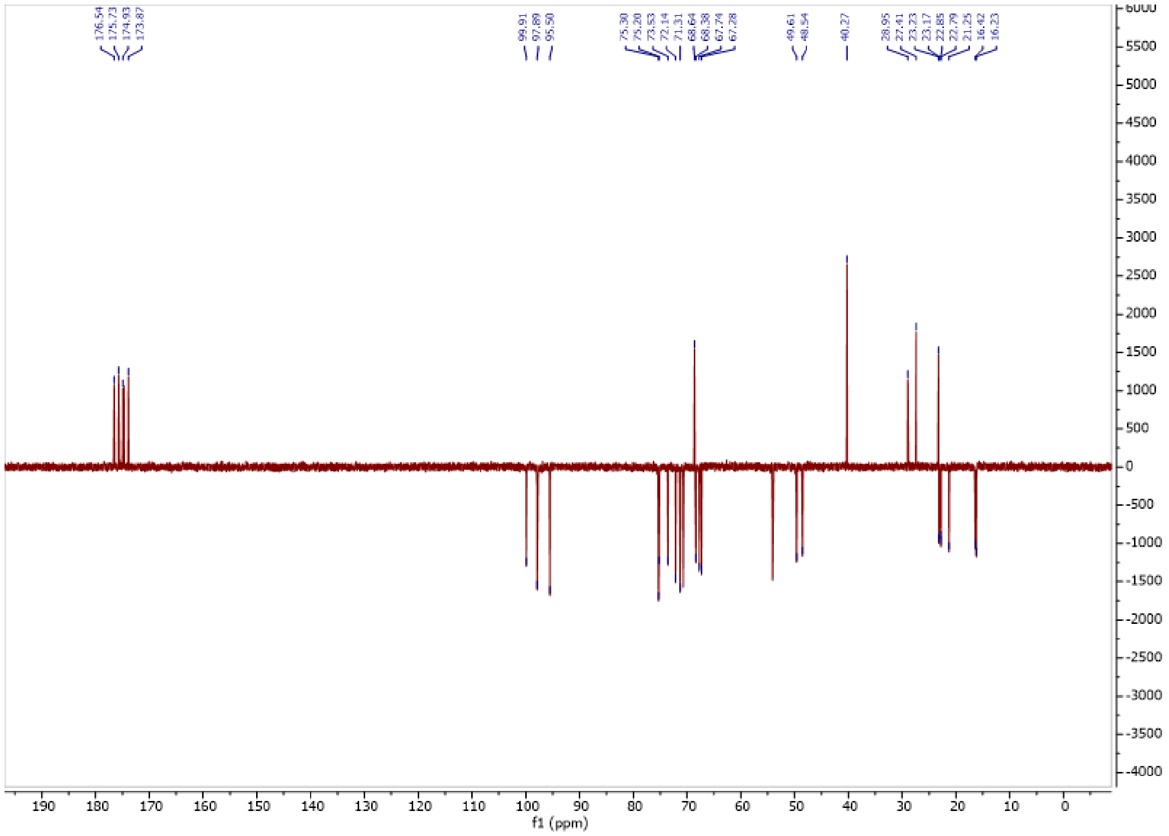
176.54
175.73
174.93
173.87
99.91
97.89
95.50
75.30
75.20
73.53
72.14
71.31
68.64
68.38
67.74
67.28
49.61
48.54
40.27
28.95
27.41
23.23
23.17
22.85
22.79
21.25
16.42
16.23
f1 (ppm)

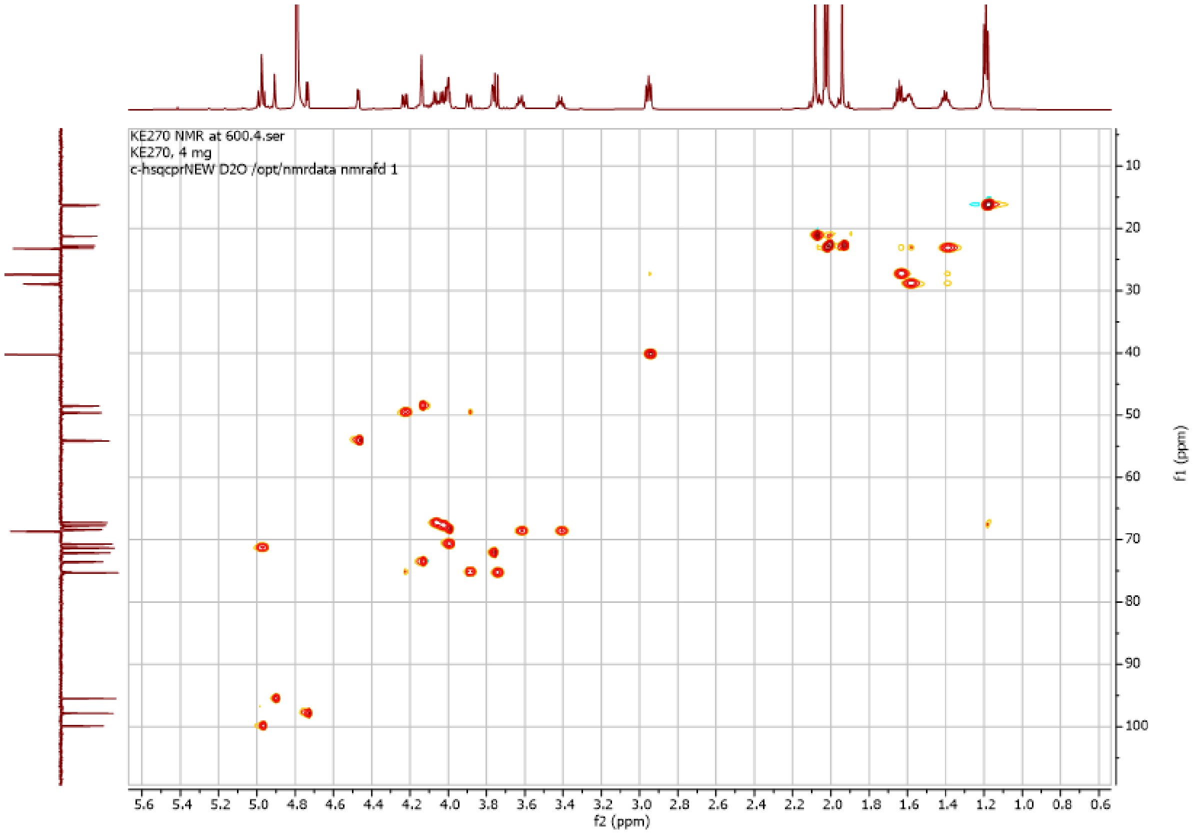
KE270 NMR at 600.4.ser
KE270, 4 mg
c-hsqcprNEW D2O /opt/nmrdata nmrafd 1
f2 (ppm)
f1 (ppm)

5-(Benzyl(benzyloxycarbonyl)amino)pentyl (Benzyl (4-*O*-acetyl-2-azido-2-deoxy-*β*-D-mannopyranosiduronsyl)-(1→3)-2-azido-4-*O*-benzyl-2-deoxy-*α*-L-fucopyranosyl-(1→3)-2-azido-4-*O*-benzyl-2-deoxy-3-*O*-(2-naphthylmethyl)-*α*-D-fucopyranoside (18)

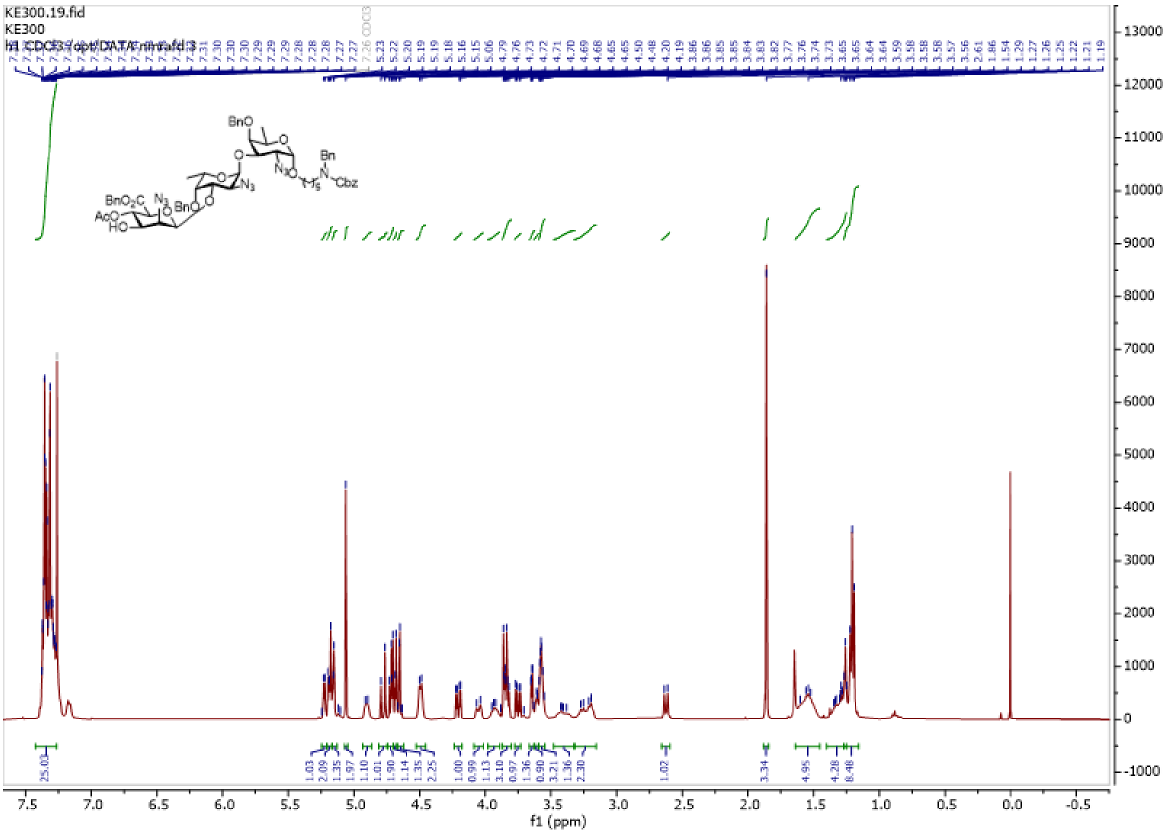

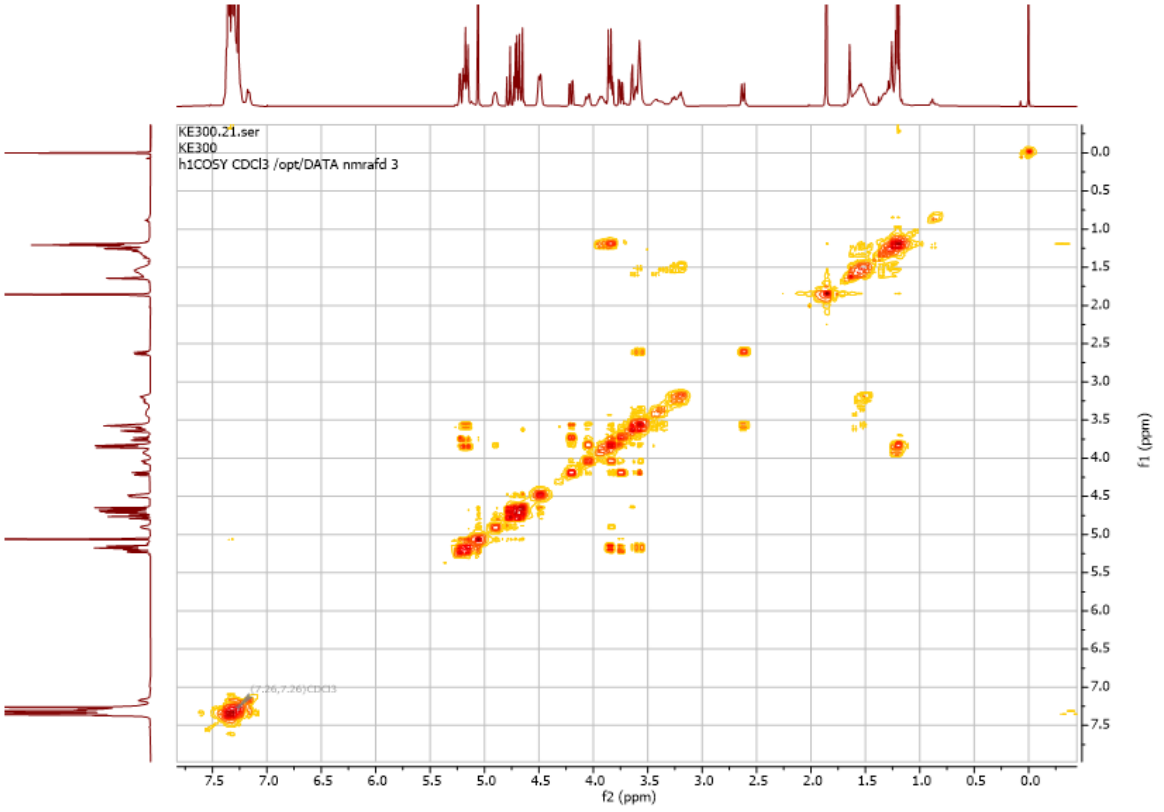

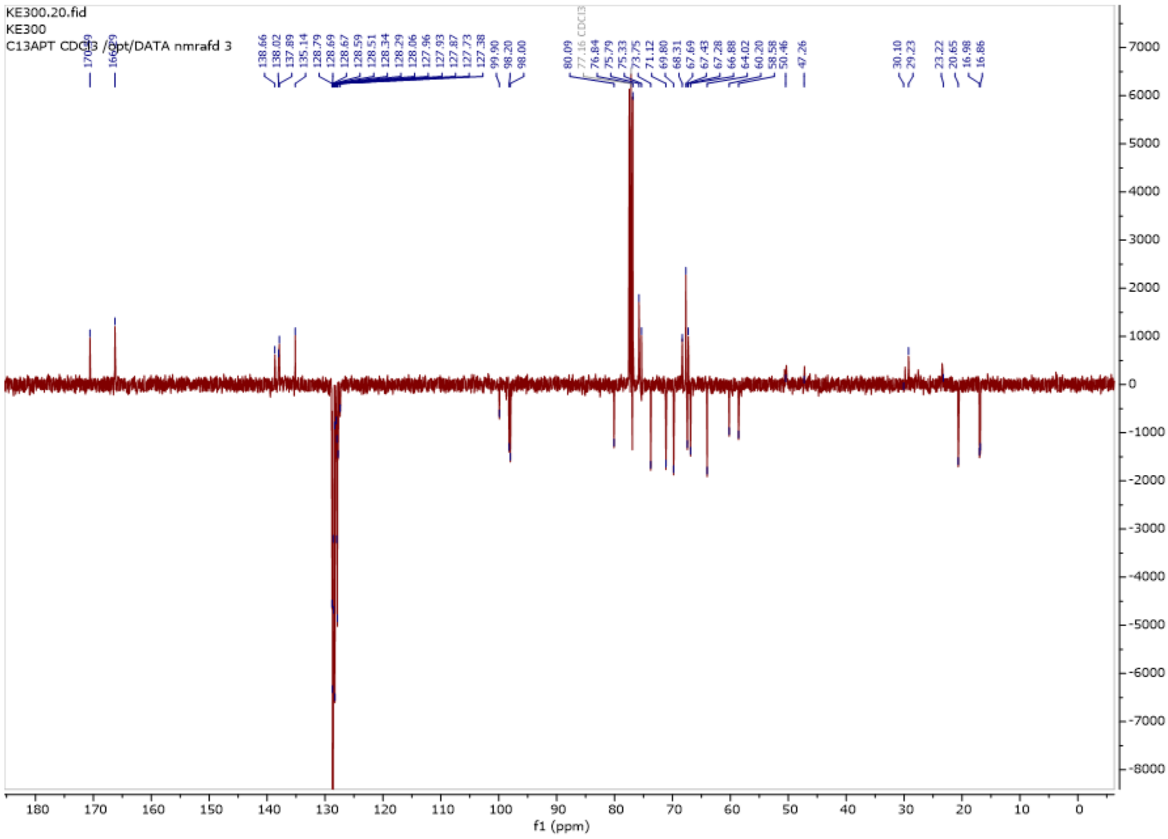
KE300.20.fid
KE300
C13APT CDCl3 /opt/DATA nmrafd 3
138.66
138.02
137.89
135.14
128.79
128.69
128.67
128.59
128.51
128.34
128.29
128.06
127.96
127.93
127.87
127.73
127.38
99.90
98.20
98.00
80.09
77.16 CDCl3
76.84
75.79
75.33
73.75
71.12
69.80
68.31
67.69
67.43
67.28
66.88
64.02
60.20
58.58
50.46
47.26
30.10
29.23
23.22
20.65
16.98
16.86
f1 (ppm)

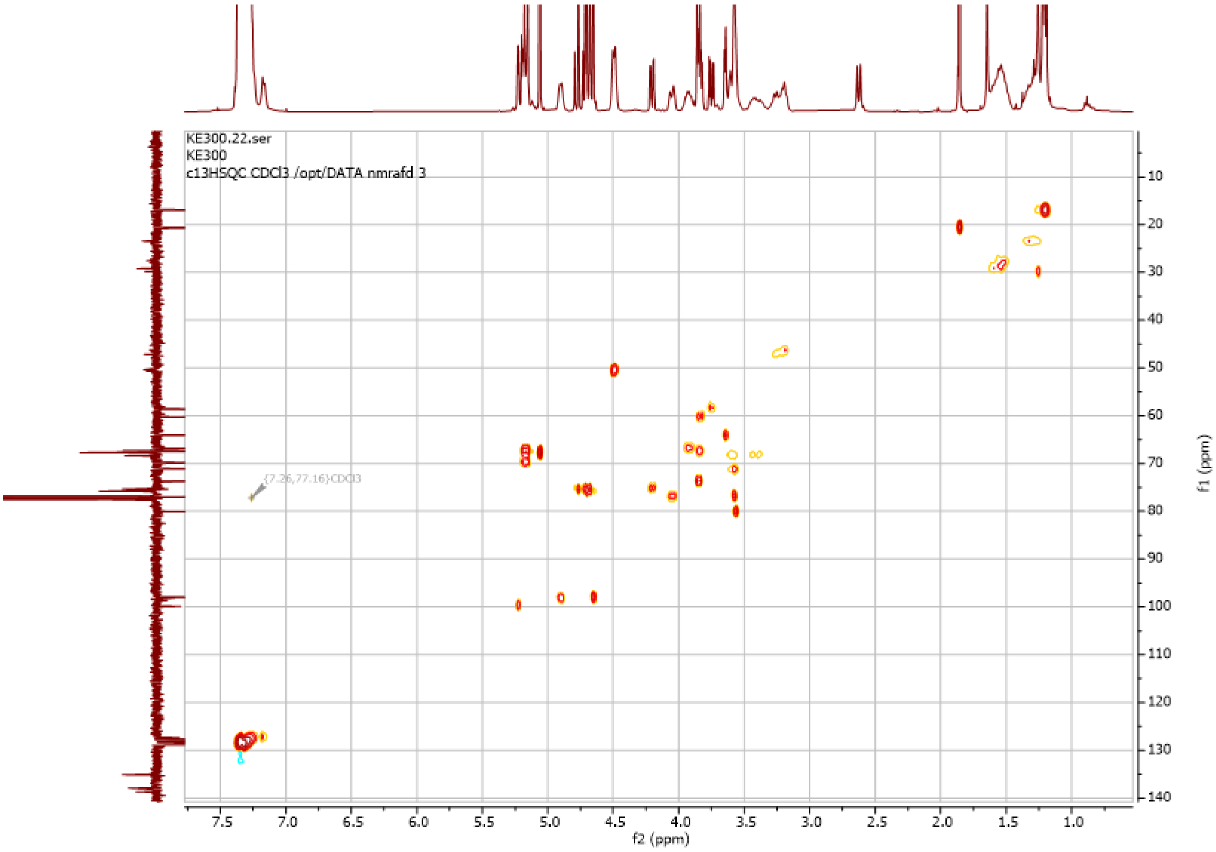
KE300.22.ser
KE300
c13HSQC CDCl3 /opt/DATA nmrafd 3
{7.26,77.16}CDCl3
f2 (ppm)
f1 (ppm)

Hexasaccharide protected (6)

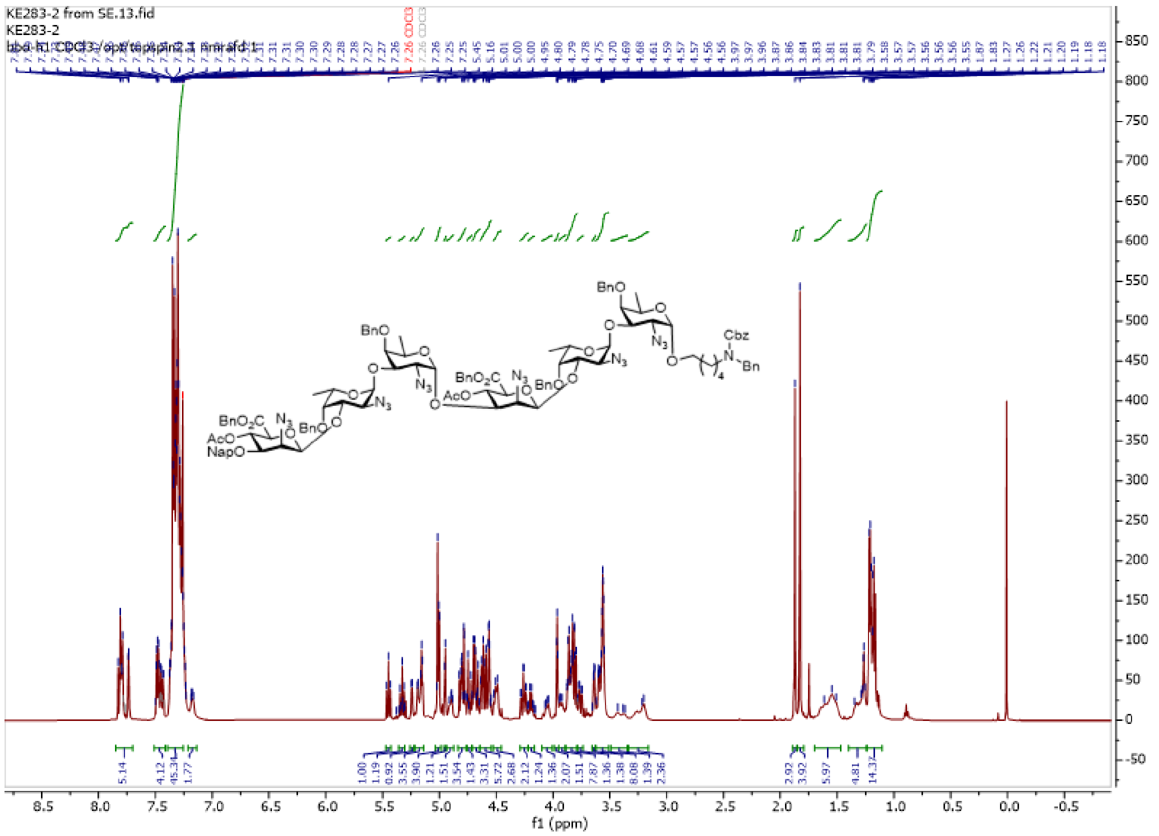

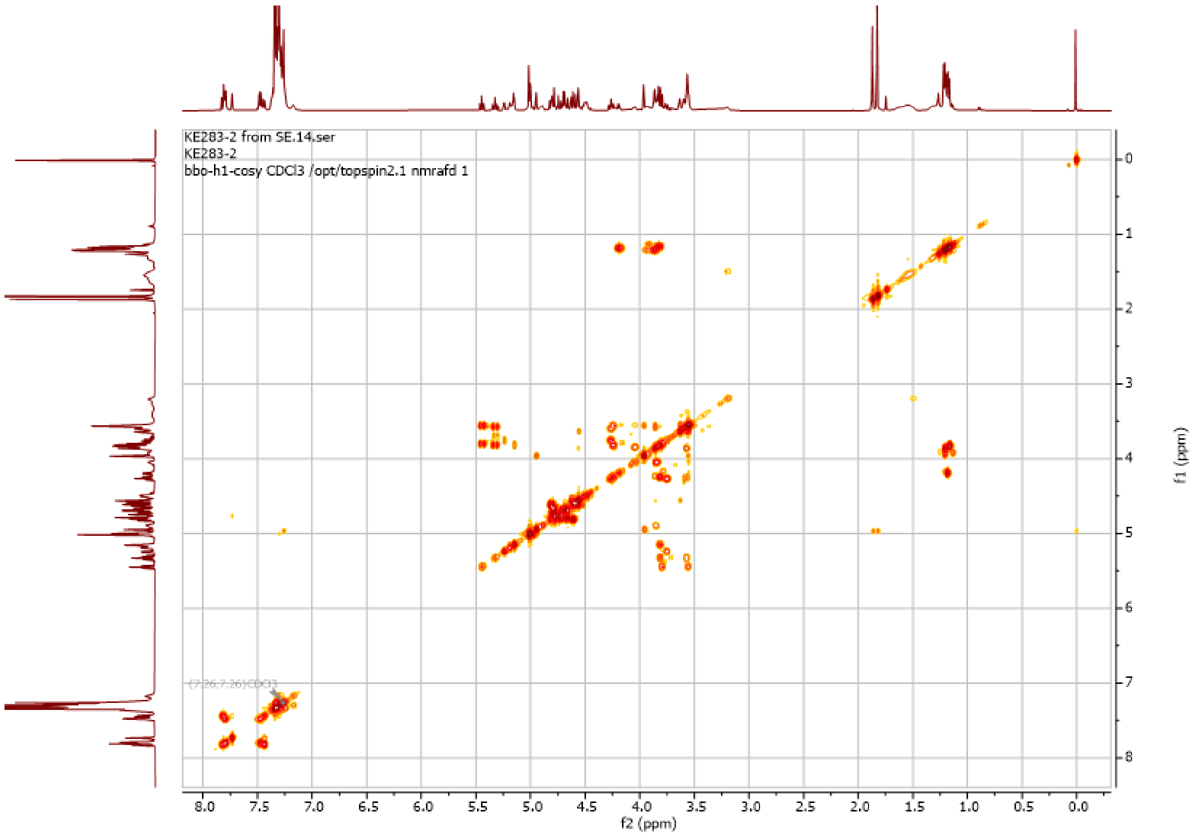

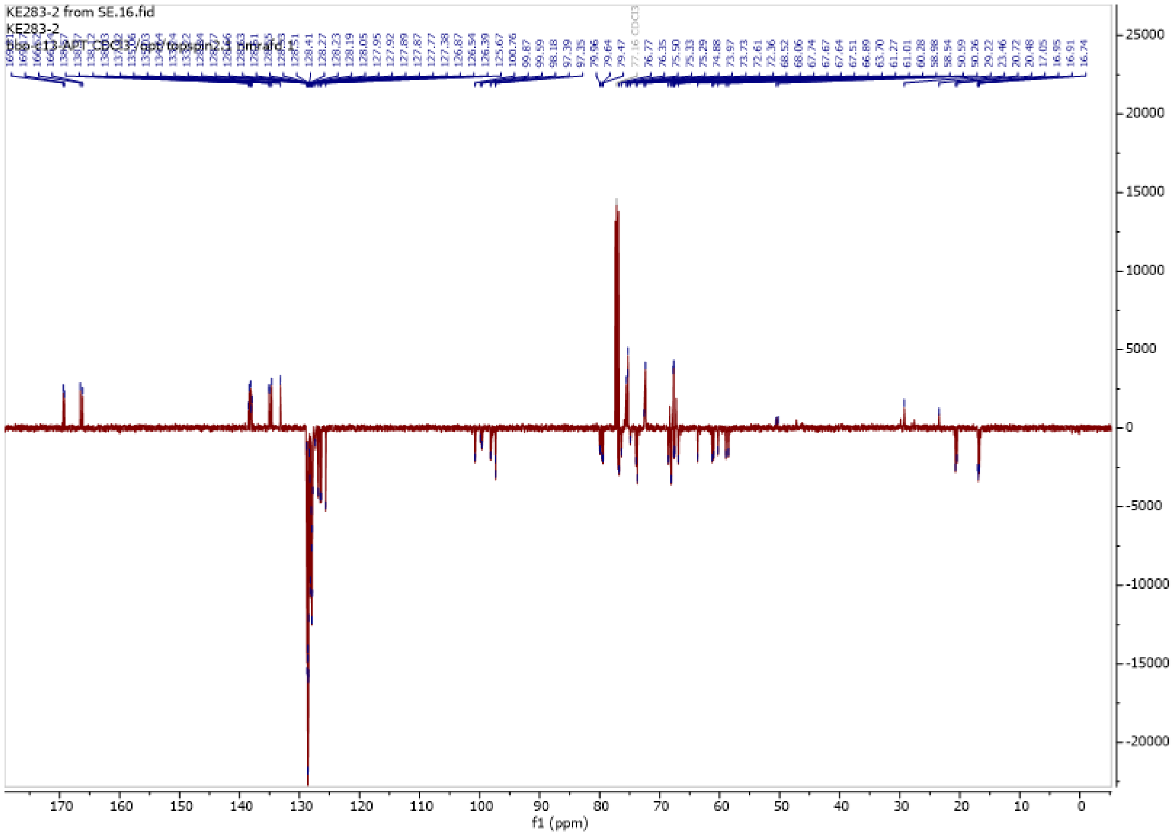
KE283-2 from SE.16.fid
KE283-2
bbo-c13-APT CDCl3 /opt/topspin2.1 nmrafd 1
f1 (ppm)

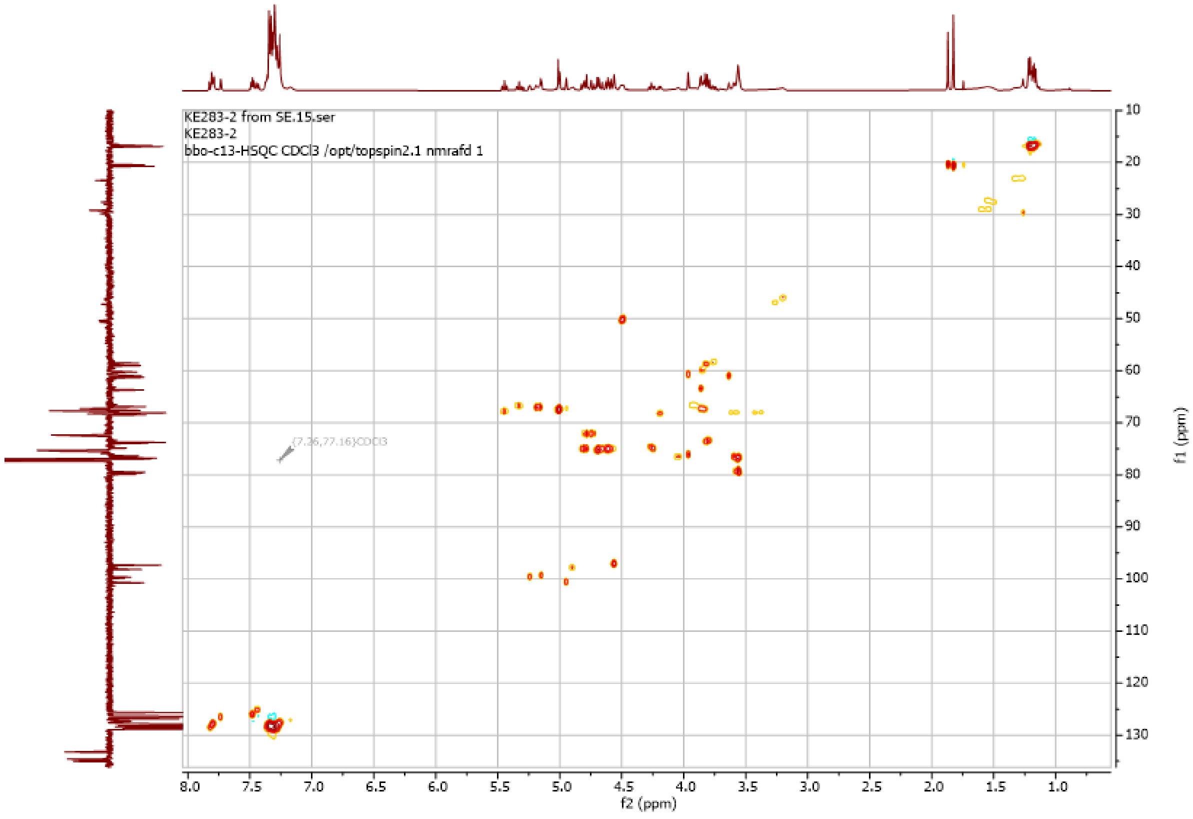
KE283-2 from SE.15.ser
KE283-2
bbo-c13-HSQC CDCl3 /opt/topspin2.1 nmrafd 1
f2 (ppm)
f1 (ppm)

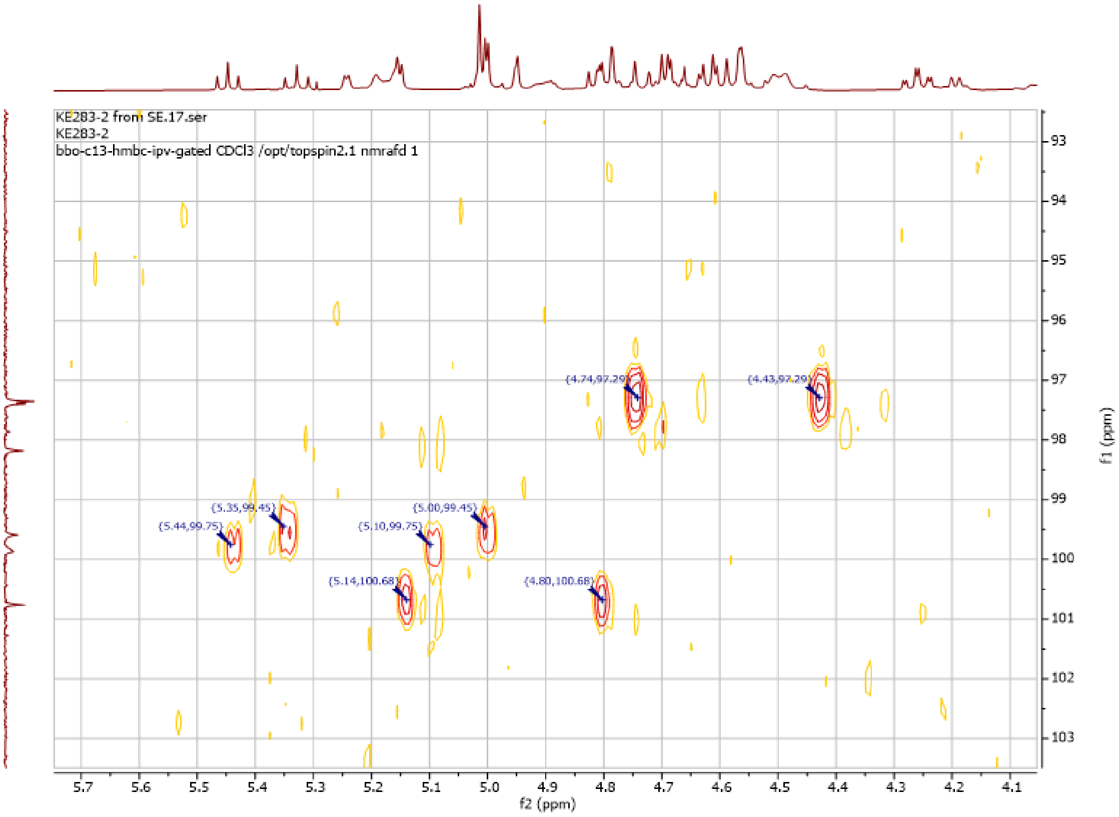

CP8-hexasaccahride (2)

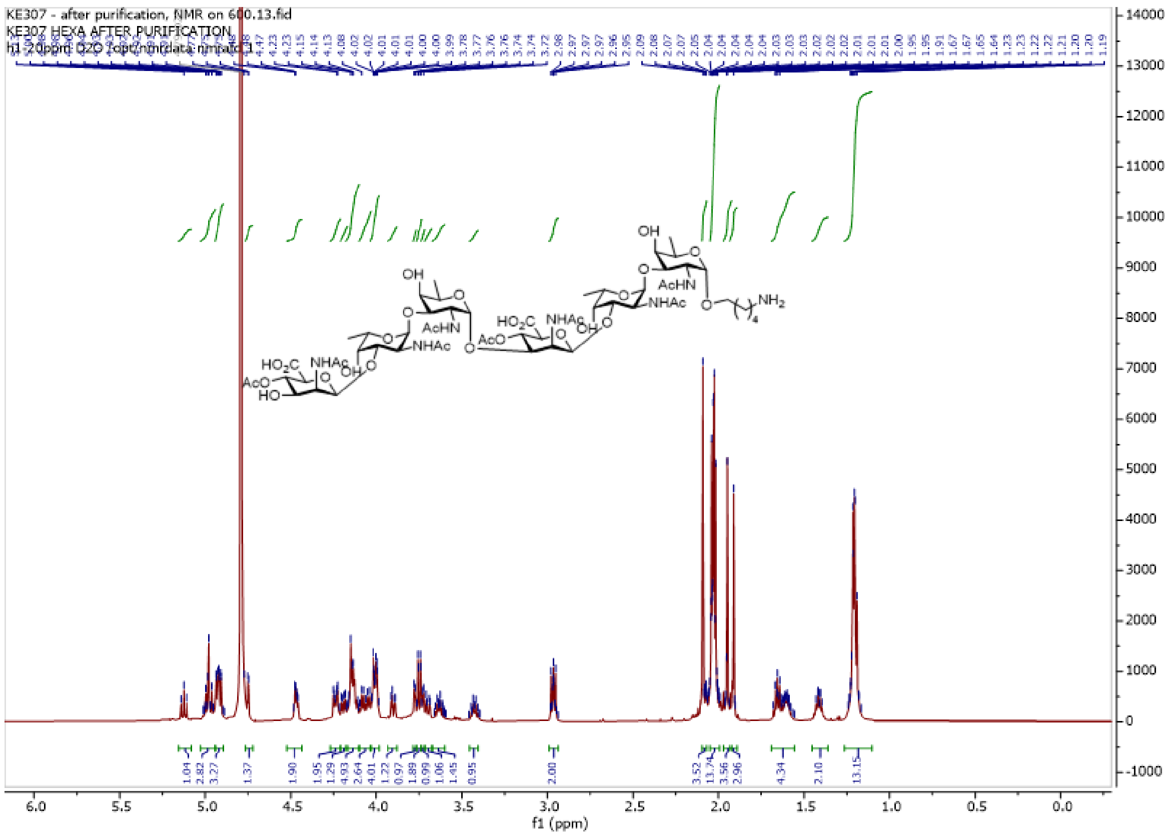

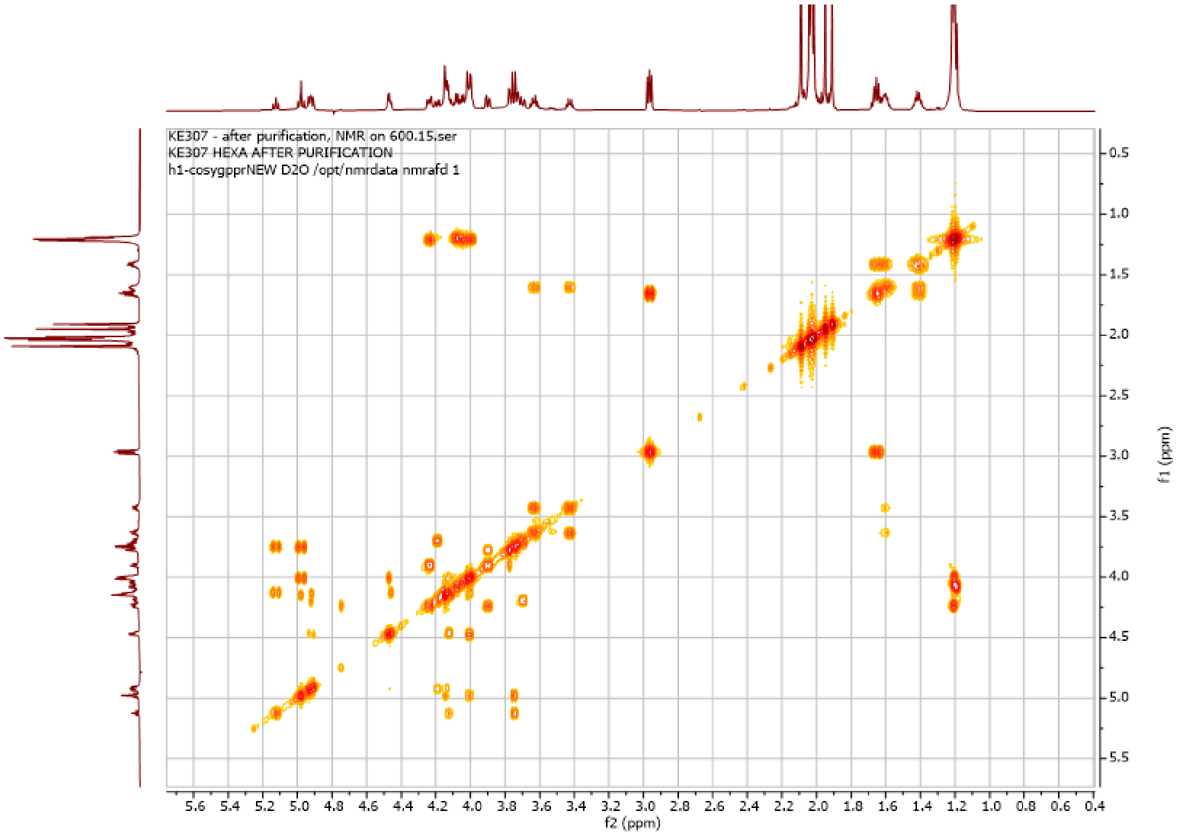
KE307 - after purification, NMR on 600.15.ser
KE307 HEXA AFTER PURIFICATION
h1-cosygpprNEW D2O /opt/nmrdata nmrafd 1
f2 (ppm)
f1 (ppm)

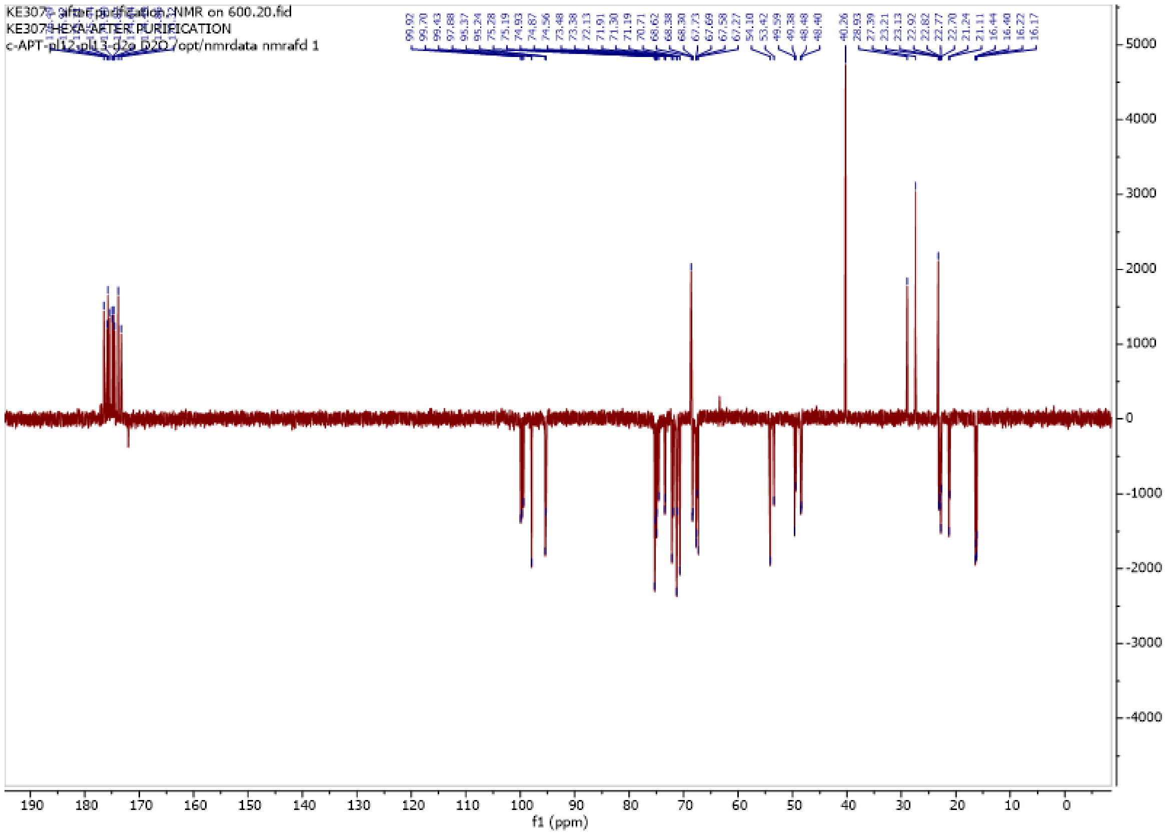
KE307 - after purification, NMR on 600.20.fid
KE307 HEXA AFTER PURIFICATION
c-APT-pl12.pl13-d2o D2O /opt/nmrdata nmrafd 1
99.92
99.70
99.43
97.88
95.37
95.24
75.28
75.19
74.93
74.87
74.56
73.48
73.38
72.13
71.91
71.30
71.19
70.71
68.62
68.38
68.30
67.73
67.69
67.58
67.27
54.10
53.42
49.59
49.38
48.48
48.40
40.26
28.93
27.39
23.21
23.13
22.92
22.82
22.77
22.70
21.24
21.11
16.44
16.40
16.22
16.17
f1 (ppm)

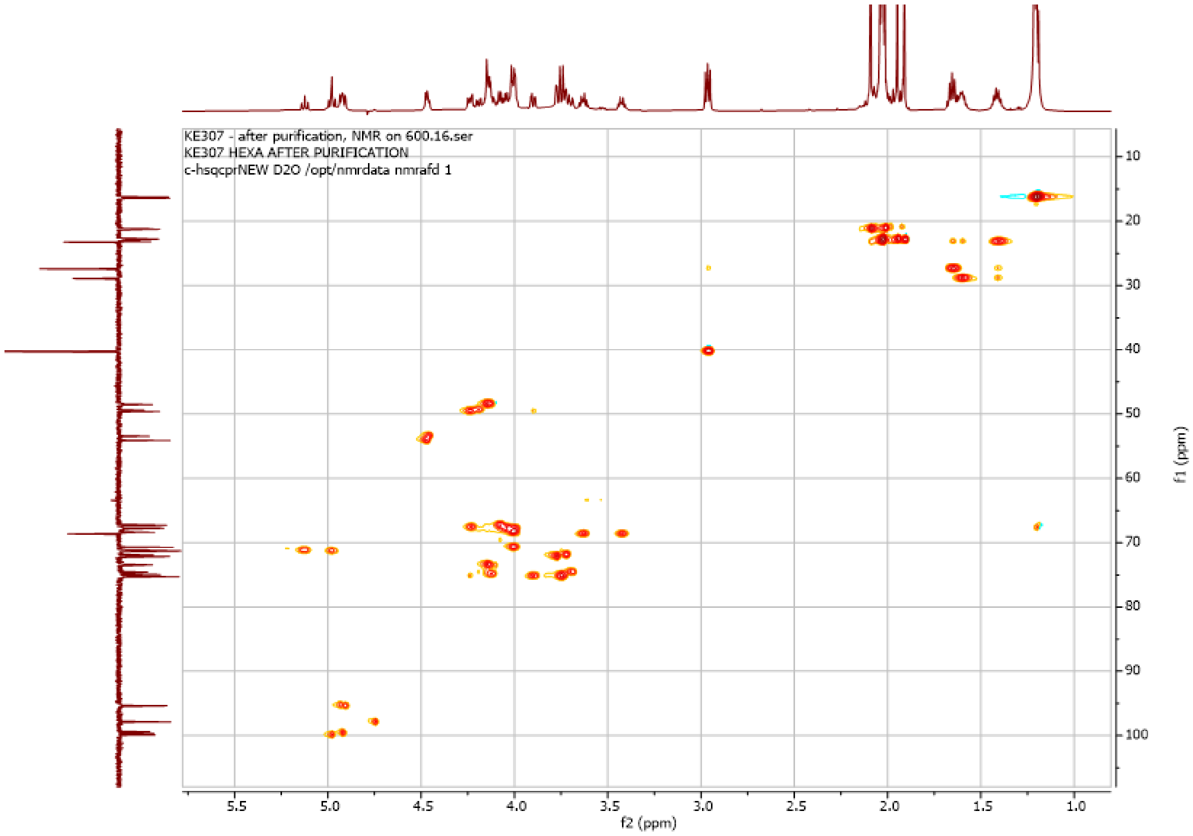
KE307 - after purification, NMR on 600.16.ser
KE307 HEXA AFTER PURIFICATION
c-hsqcprNEW D2O /opt/nmrdata nmrafd 1
f2 (ppm)
f1 (ppm)

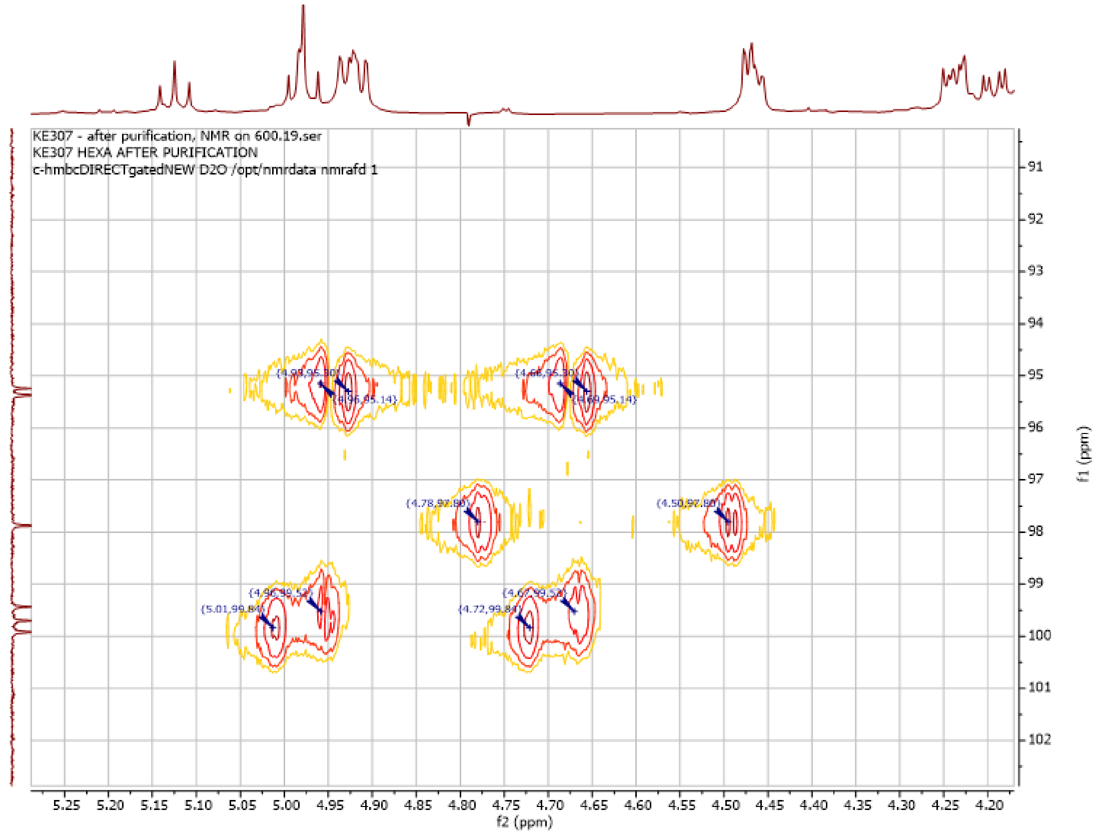
KE307 - after purification, NMR on 600.19.ser
KE307 HEXA AFTER PURIFICATION
c-hmbcDIRECTgatedNEW D2O /opt/nmrdata nmrafd 1
(4.78,97.80)
(4.50,97.80)
(5.01,99.84)
(4.72,99.84)
f2 (ppm)
f1 (ppm)

Hexasaccharide-acceptor (19)

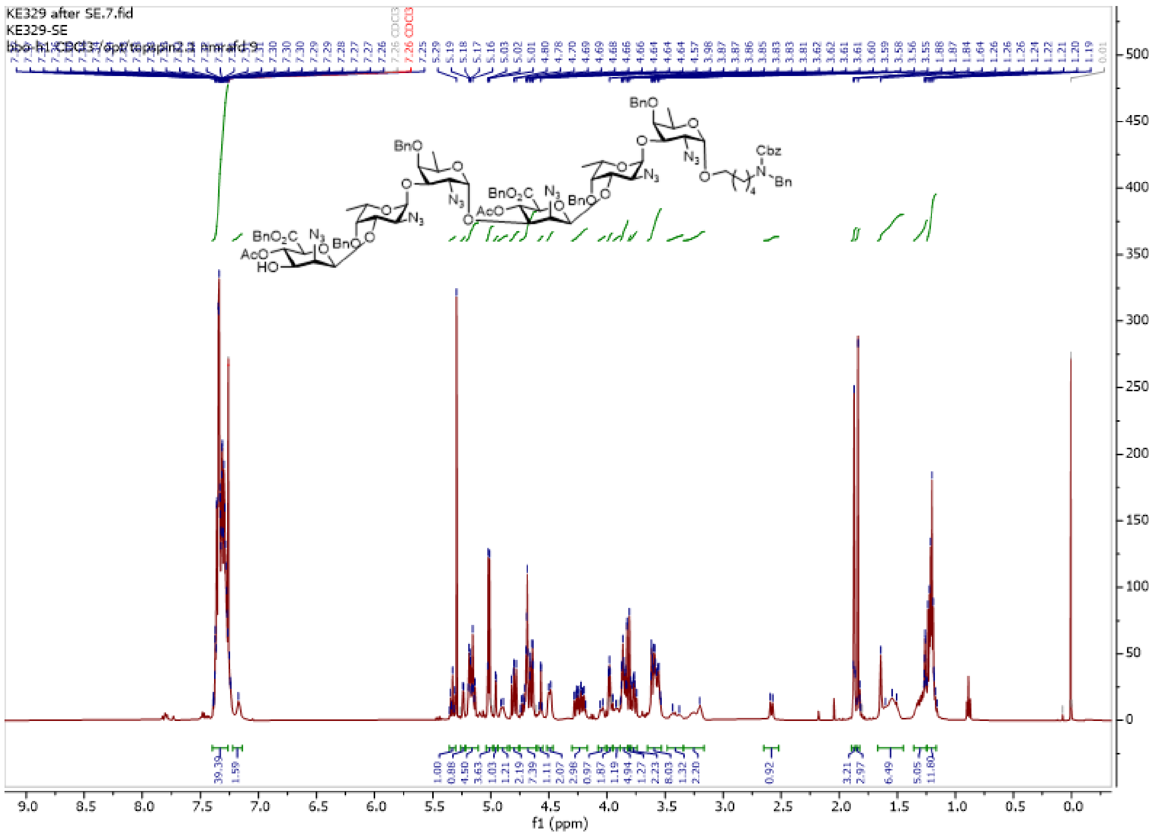

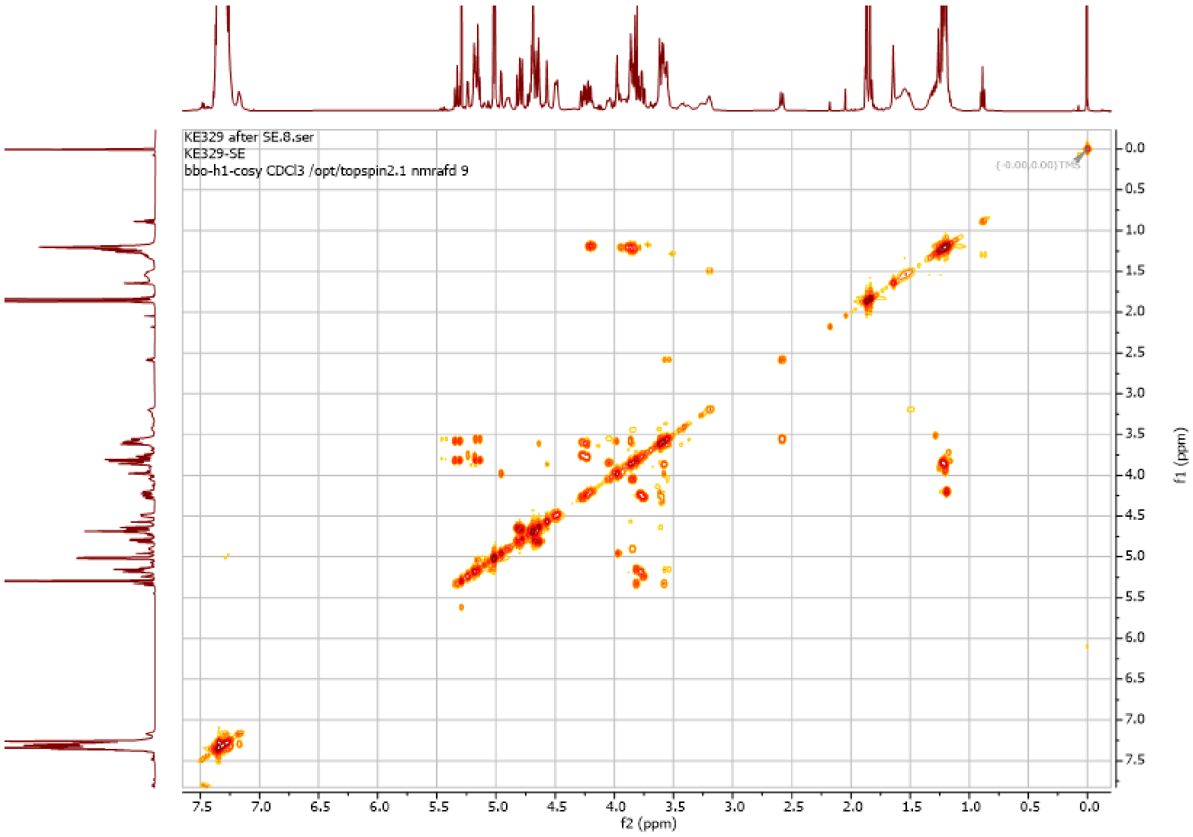

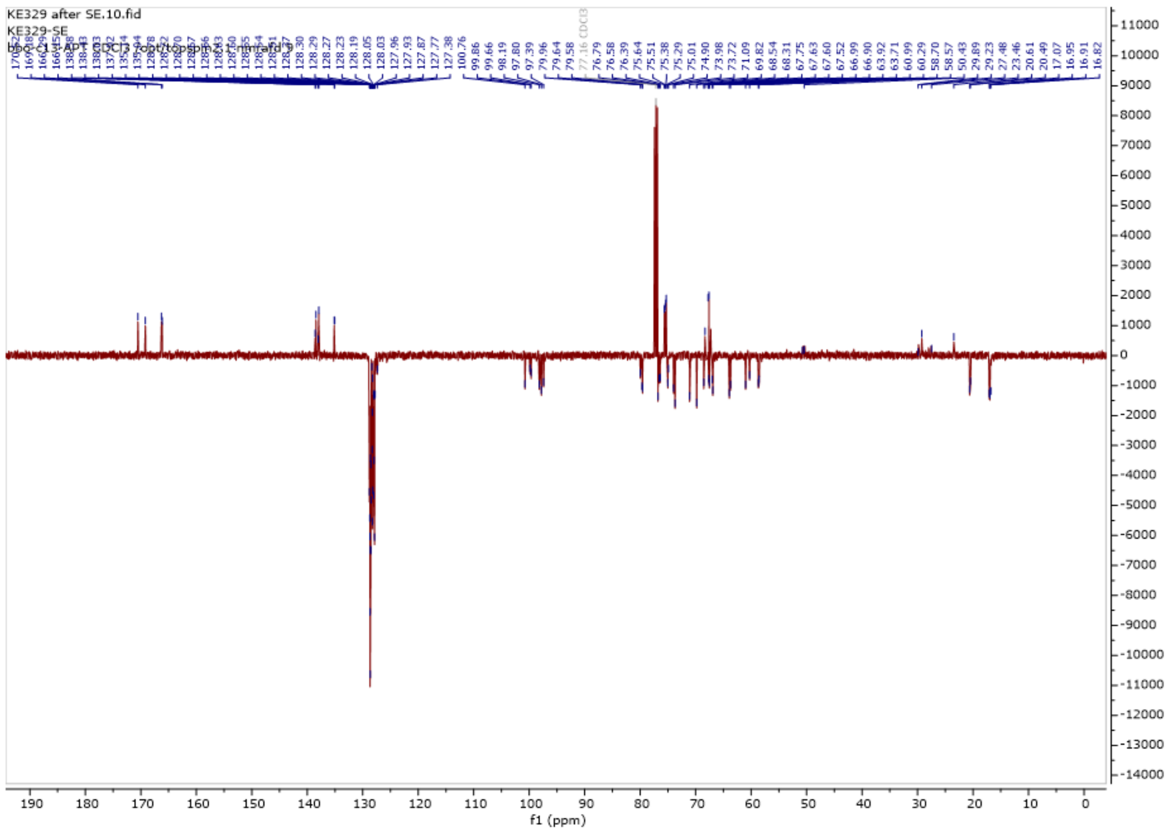
KE329 after SE.10.fid
KE329-SE
f1 (ppm)

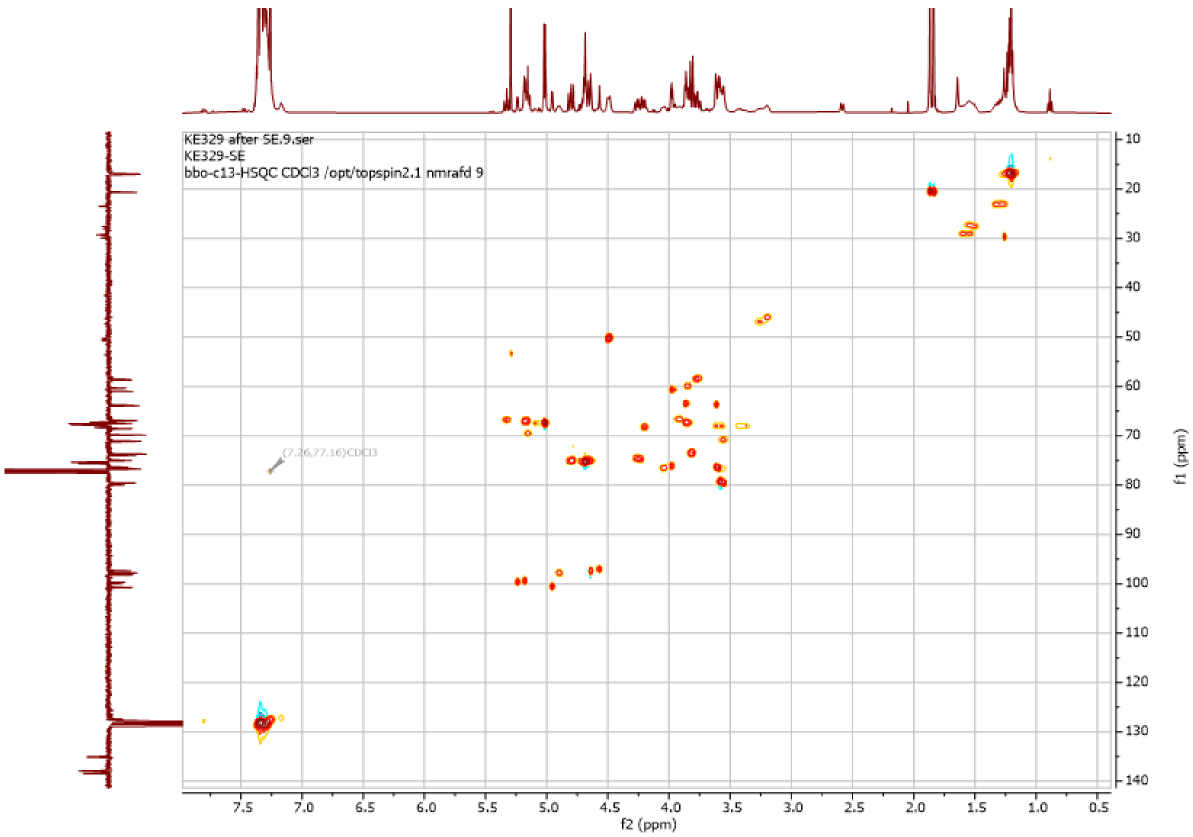
KE329 after SE.9.ser
KE329-SE
bbo-c13-HSQC CDCl3 /opt/topspin2.1 nmrafd 9
f2 (ppm)
f1 (ppm)

Nonasaccharide protected (7)

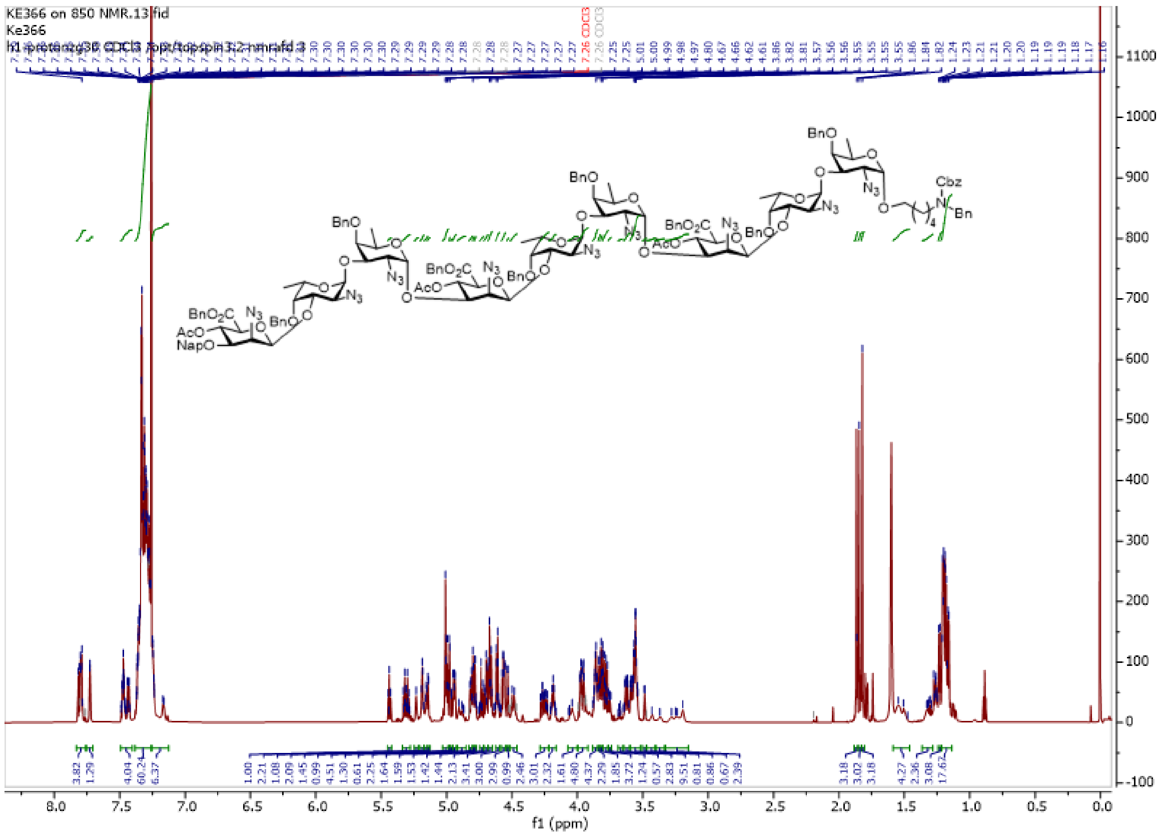

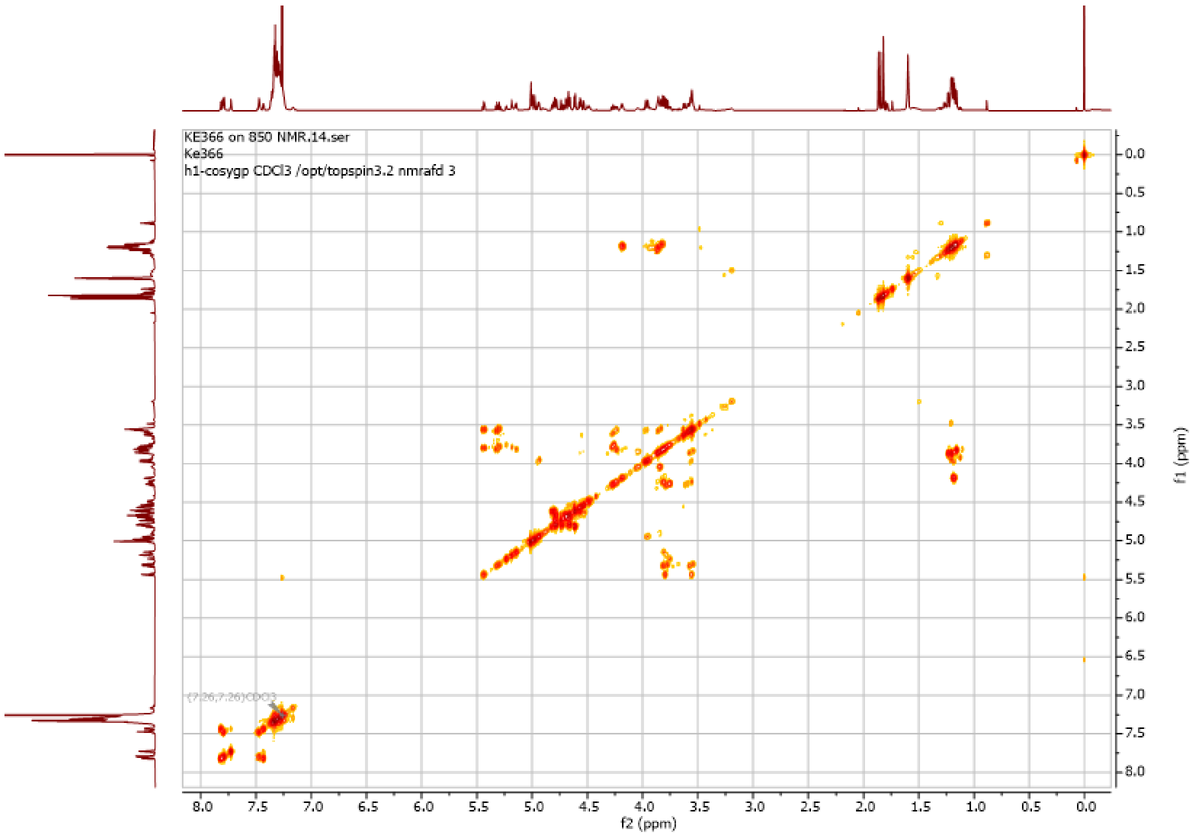

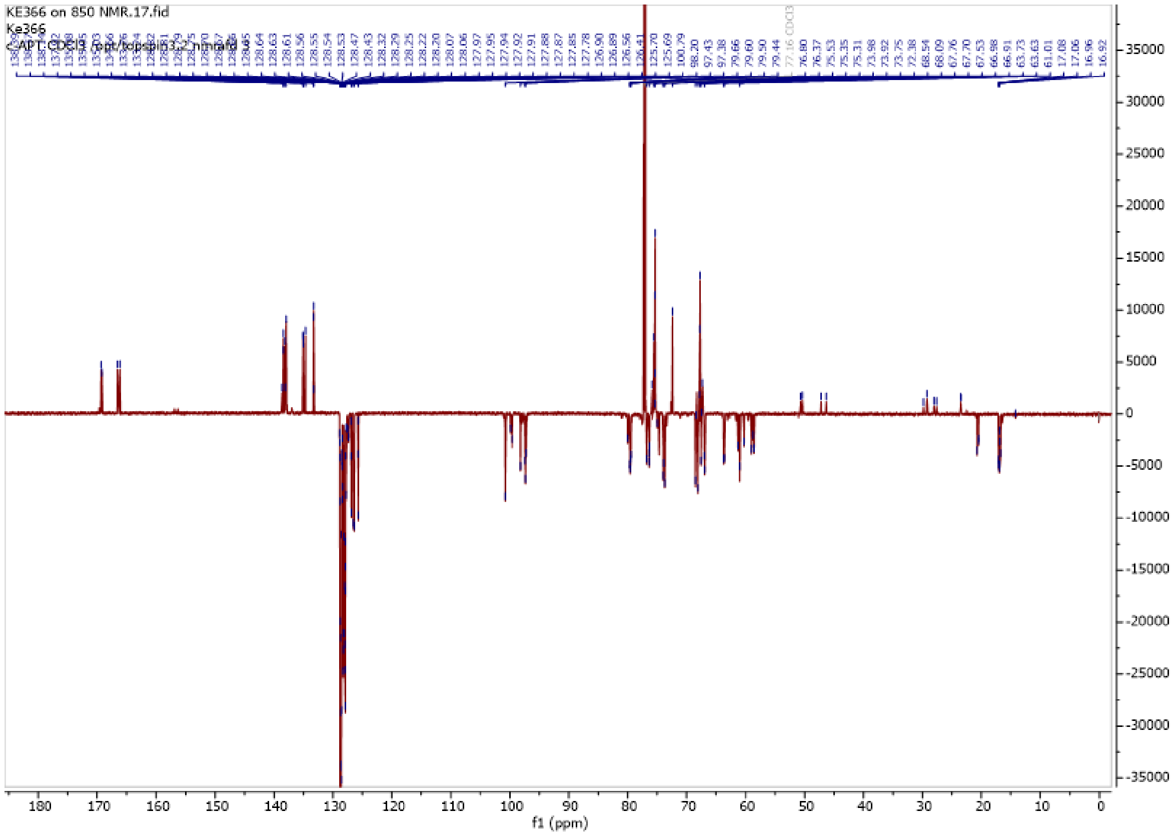
KE366 on 850 NMR.17.fid
Ke366
c-APT CDCl3 /opt/topspin3.2 nmrafd 8
77.16 CDCl3
f1 (ppm)

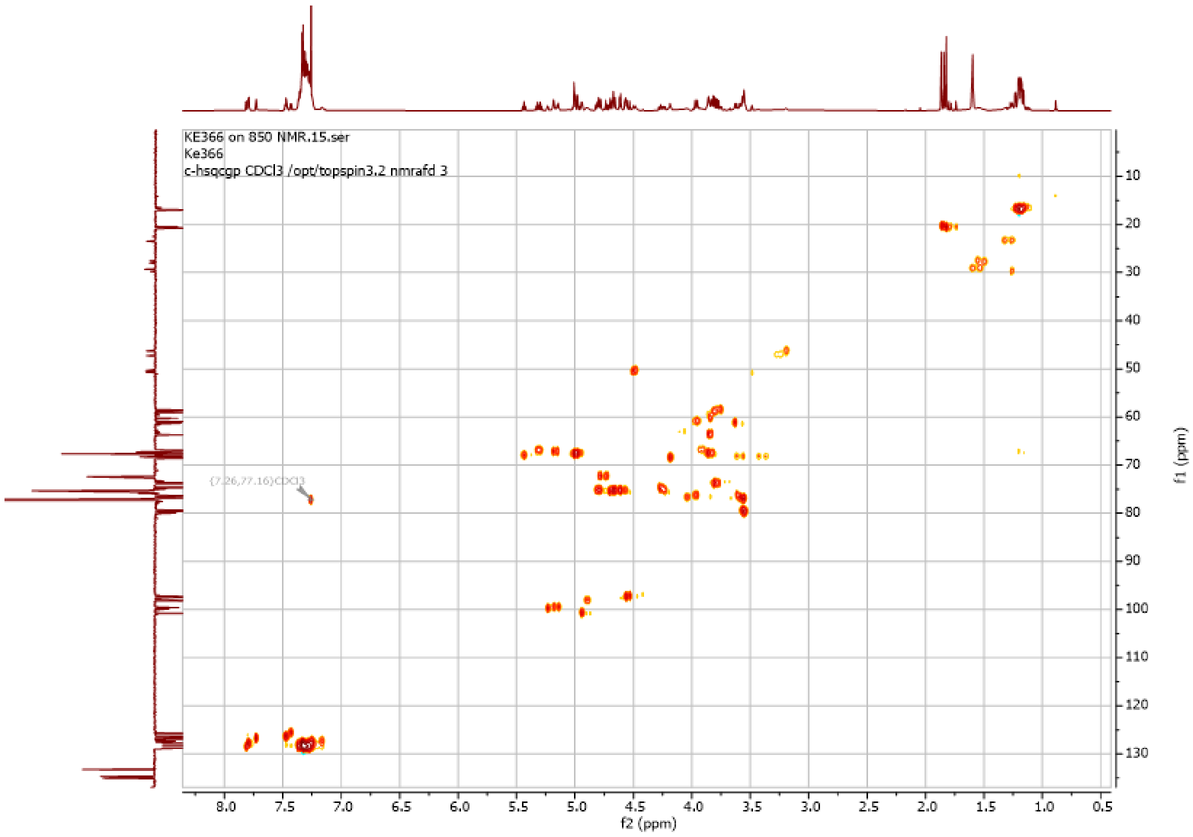
KE366 on 850 NMR.15.ser
Ke366
c-hsqcgp CDCl3 /opt/topspin3.2 nmrafd 3
{7.26,77.16}CDCl3
f2 (ppm)
f1 (ppm)

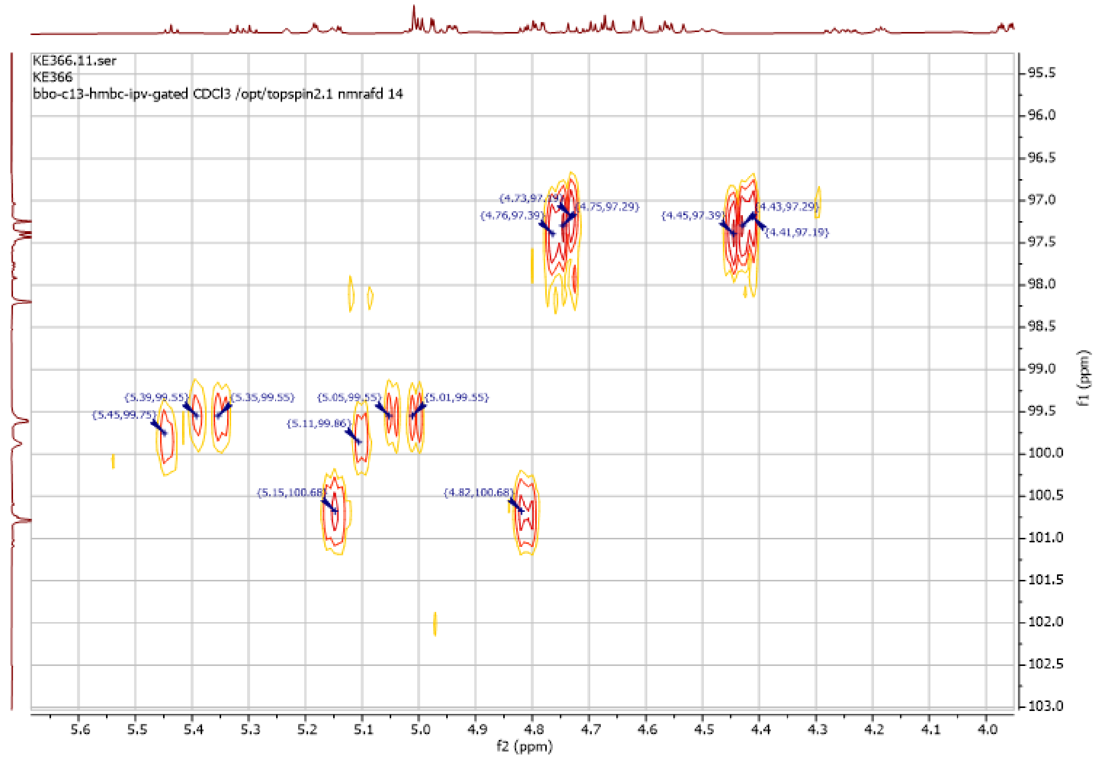

CP8-Nonasaccharide (3)

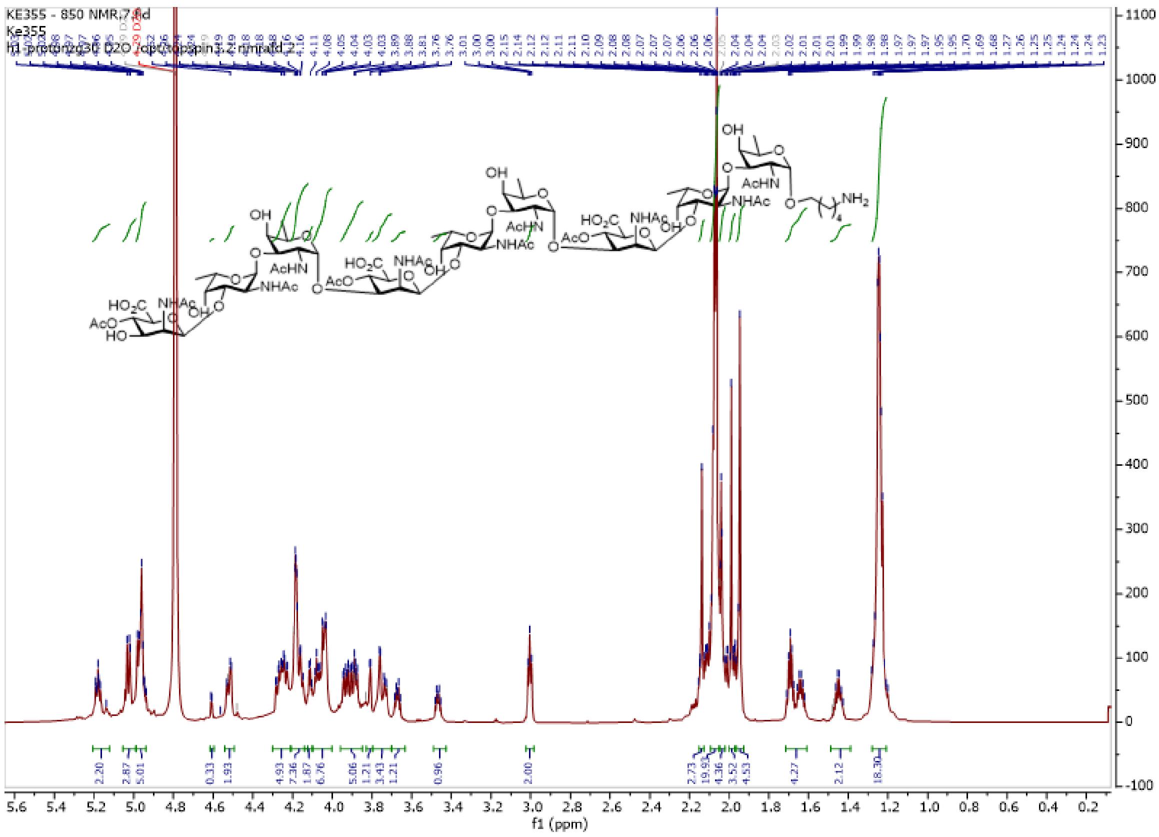

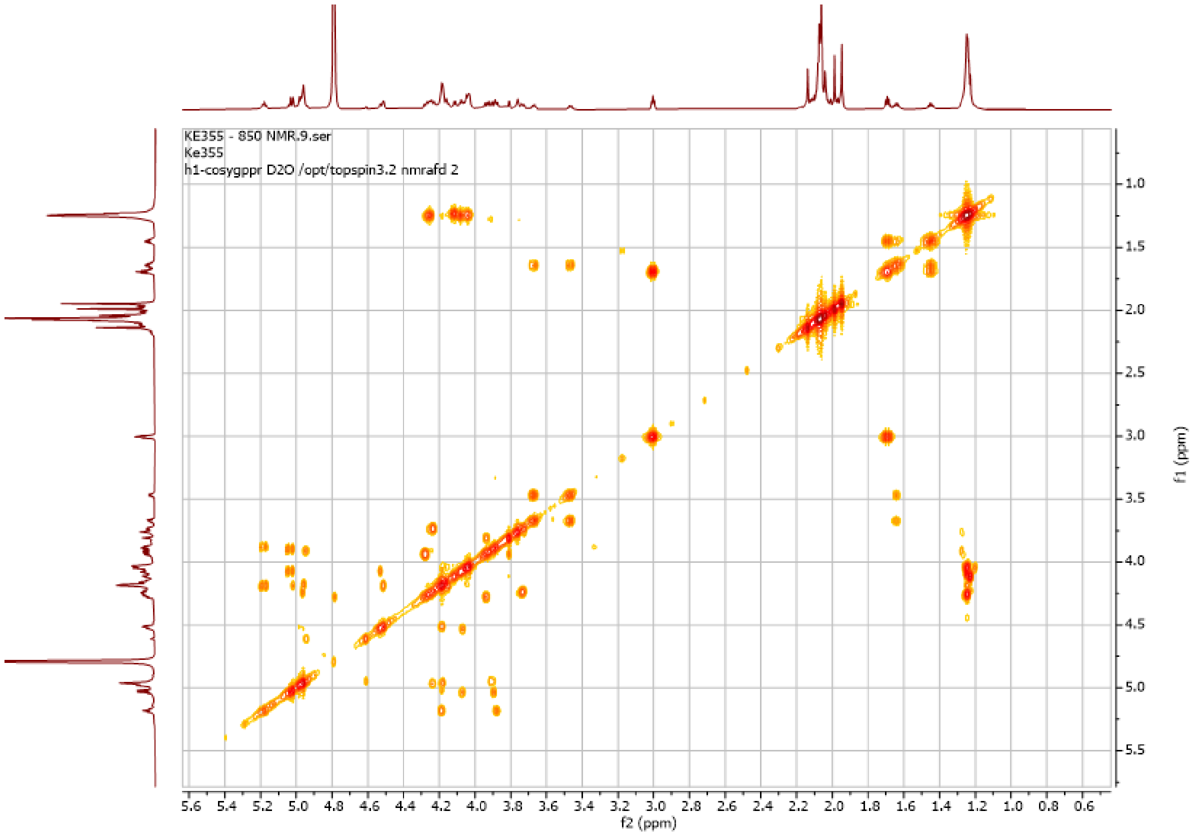
KE355 - 850 NMR.9.ser
Ke355
h1-cosygppr D2O /opt/topspin3.2 nmrafd 2
f2 (ppm)
f1 (ppm)

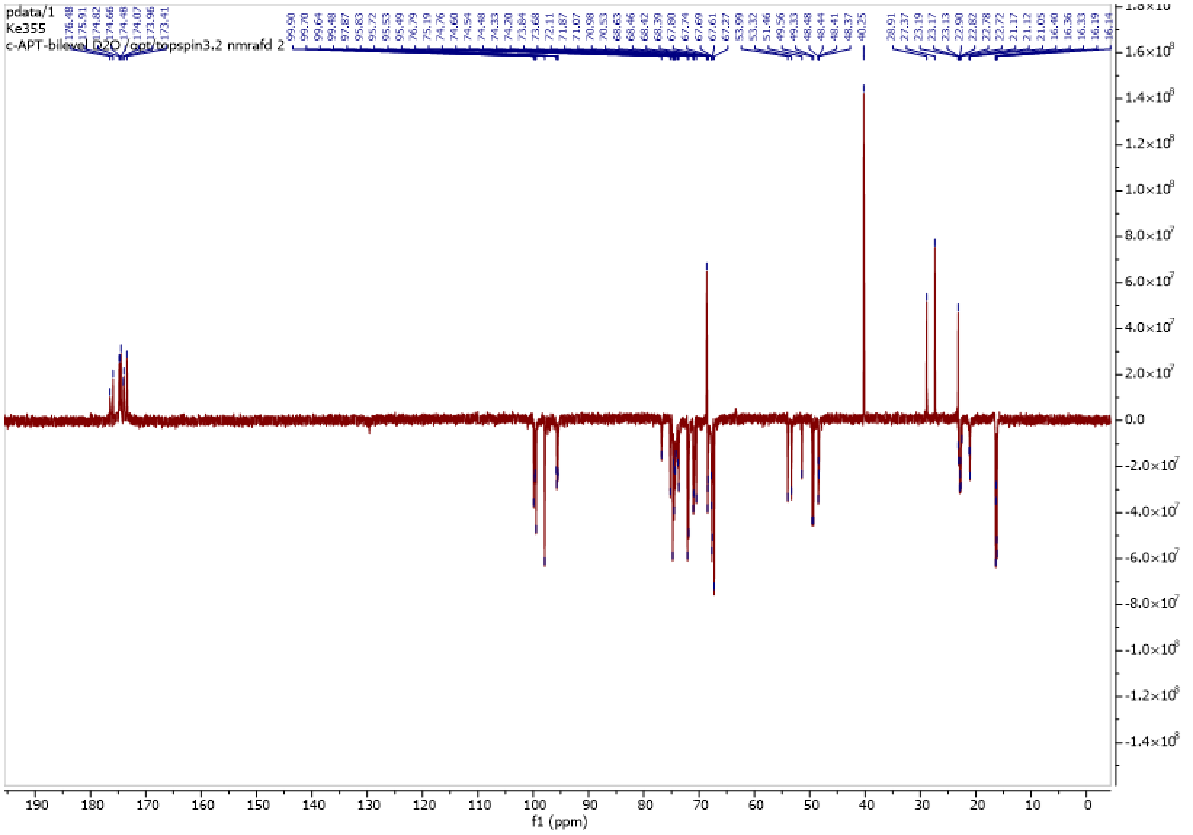
pdata/1
Ke355
c-APT-bilevel D2O /opt/topspin3.2 nmrafd 2
f1 (ppm)

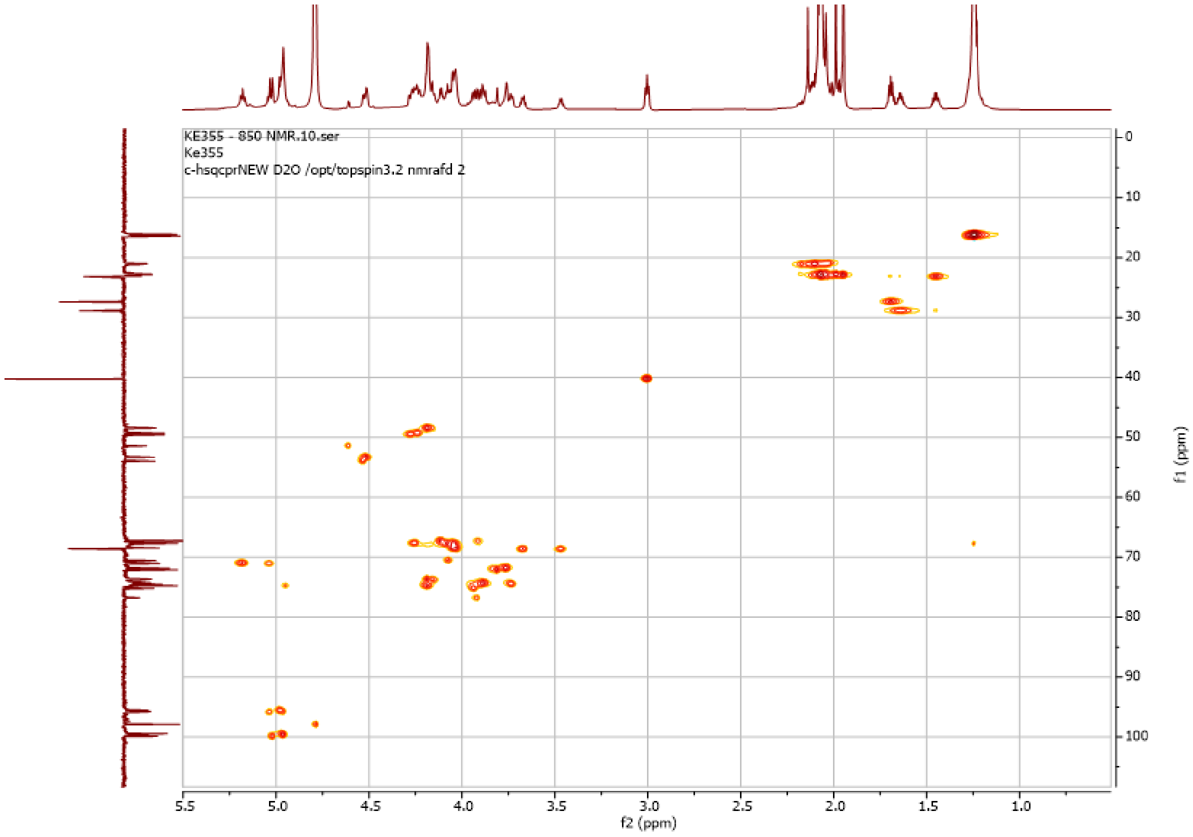

Nona-acceptor (20)

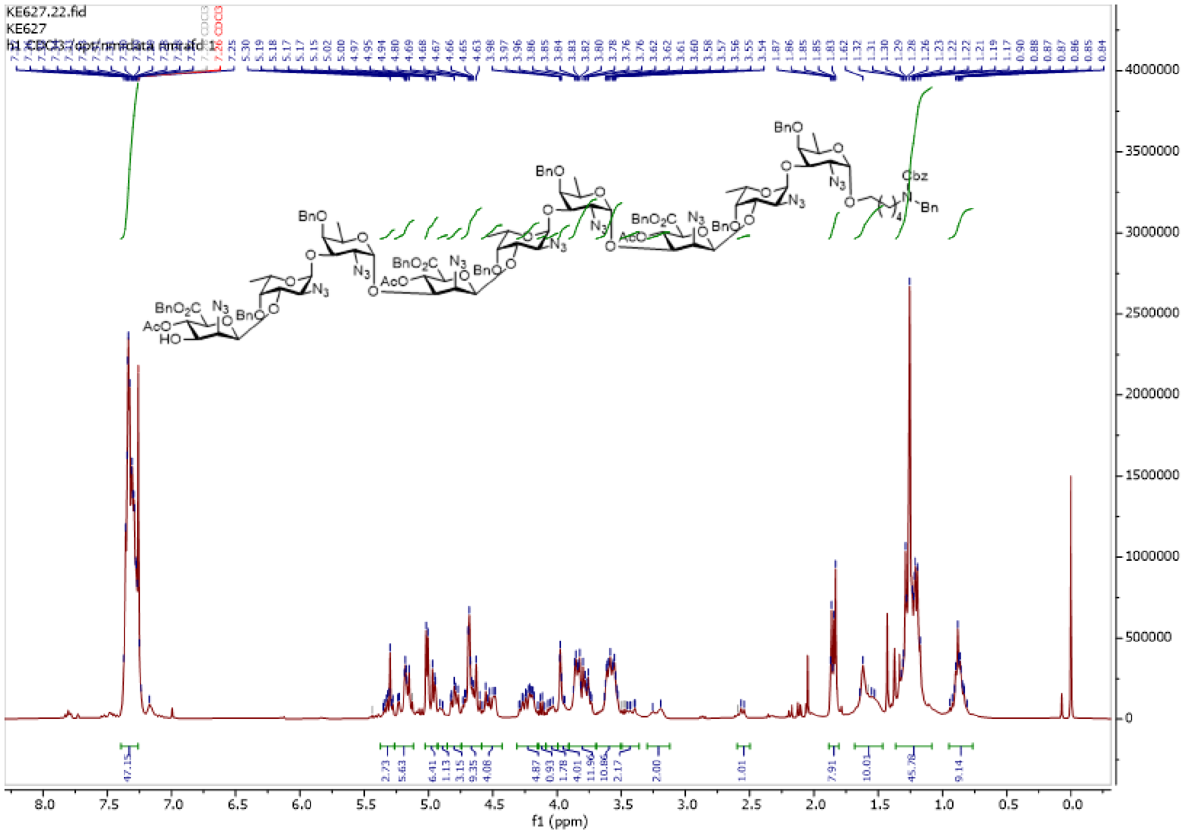

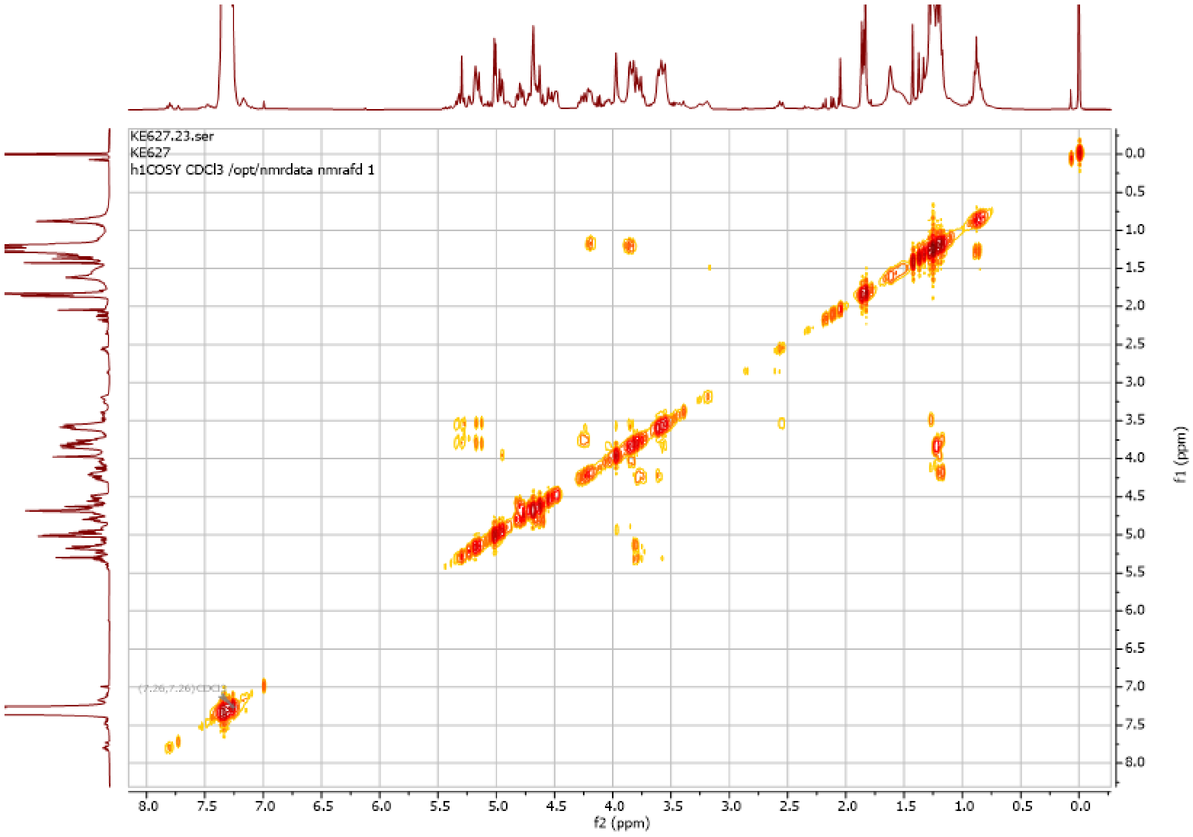
KE627.23.ser
KE627
h1COSY CDCl3 /opt/nmrdata nmrafd 1
f2 (ppm)
f1 (ppm)

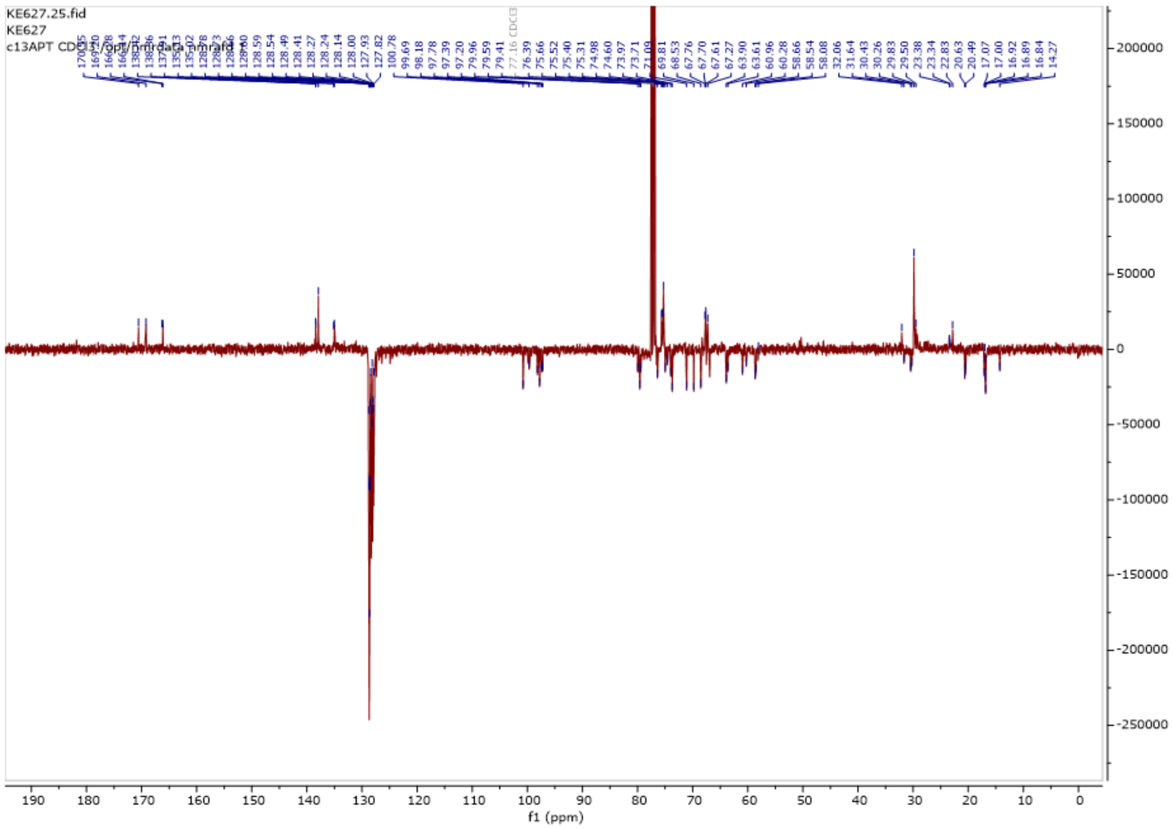
KE627.25.fid
KE627
c13APT CDCl3 /opt/nmrdata nmrafd 1
128.59
128.54
128.49
128.41
128.27
128.24
128.14
128.00
127.93
127.82
100.78
99.69
98.18
97.78
97.39
97.20
79.96
79.59
79.41
77.16 CDCl3
76.39
75.66
75.52
75.40
75.31
74.98
74.60
73.97
73.71
71.09
69.81
68.53
67.76
67.70
67.61
67.27
63.90
63.61
60.96
60.28
58.66
58.54
58.08
32.06
31.64
30.43
30.26
29.83
29.50
23.38
23.34
22.83
20.63
20.49
17.07
17.00
16.92
16.89
16.84
14.27
f1 (ppm)

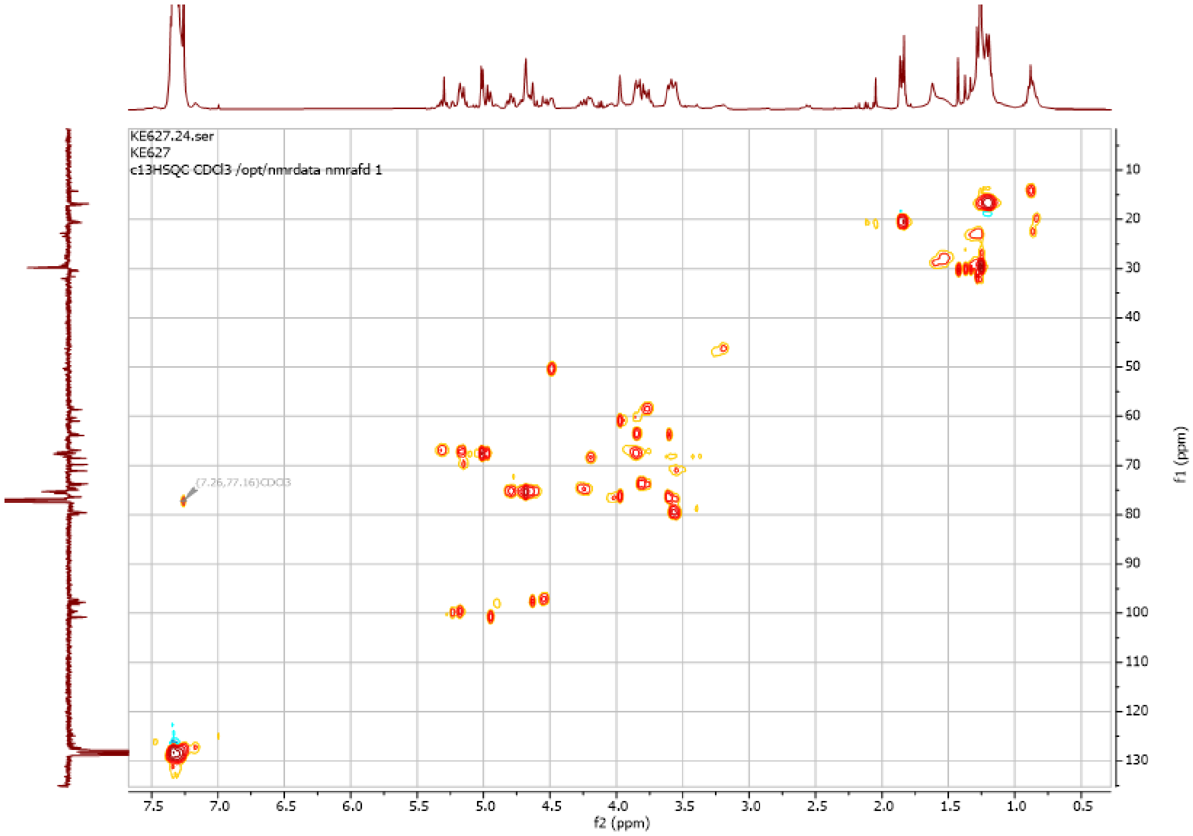

Dodeca-saccharide (8)

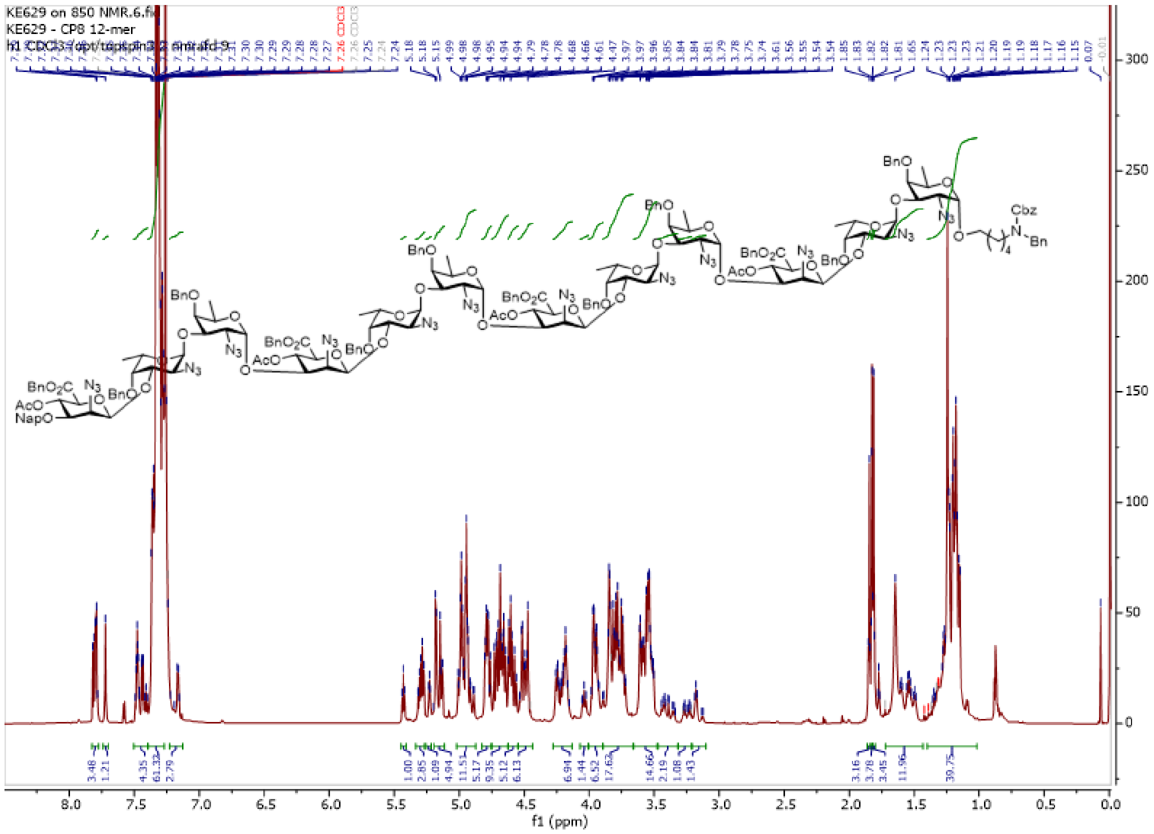

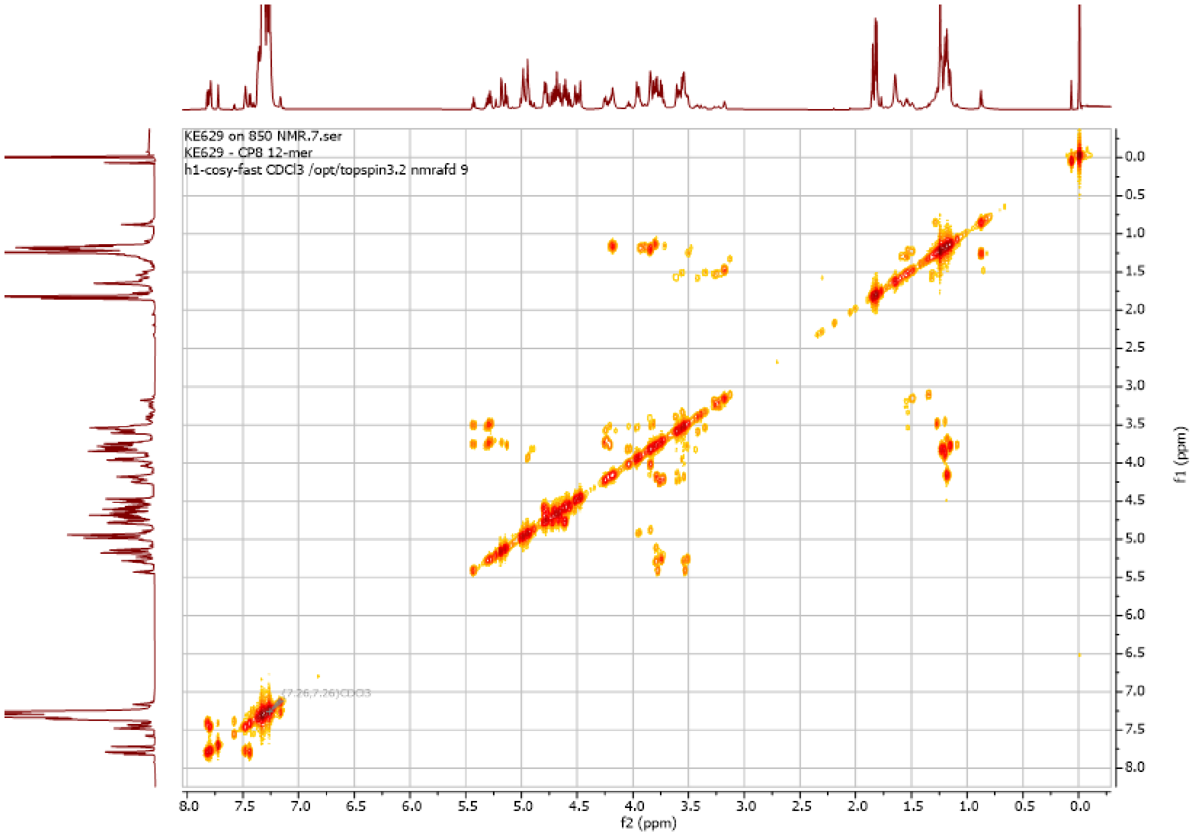
KE629 on 850 NMR.7.ser
KE629 - CP8 12-mer
h1-cosy-fast CDCl3 /opt/topspin3.2 nmrafd 9
f2 (ppm)
f1 (ppm)

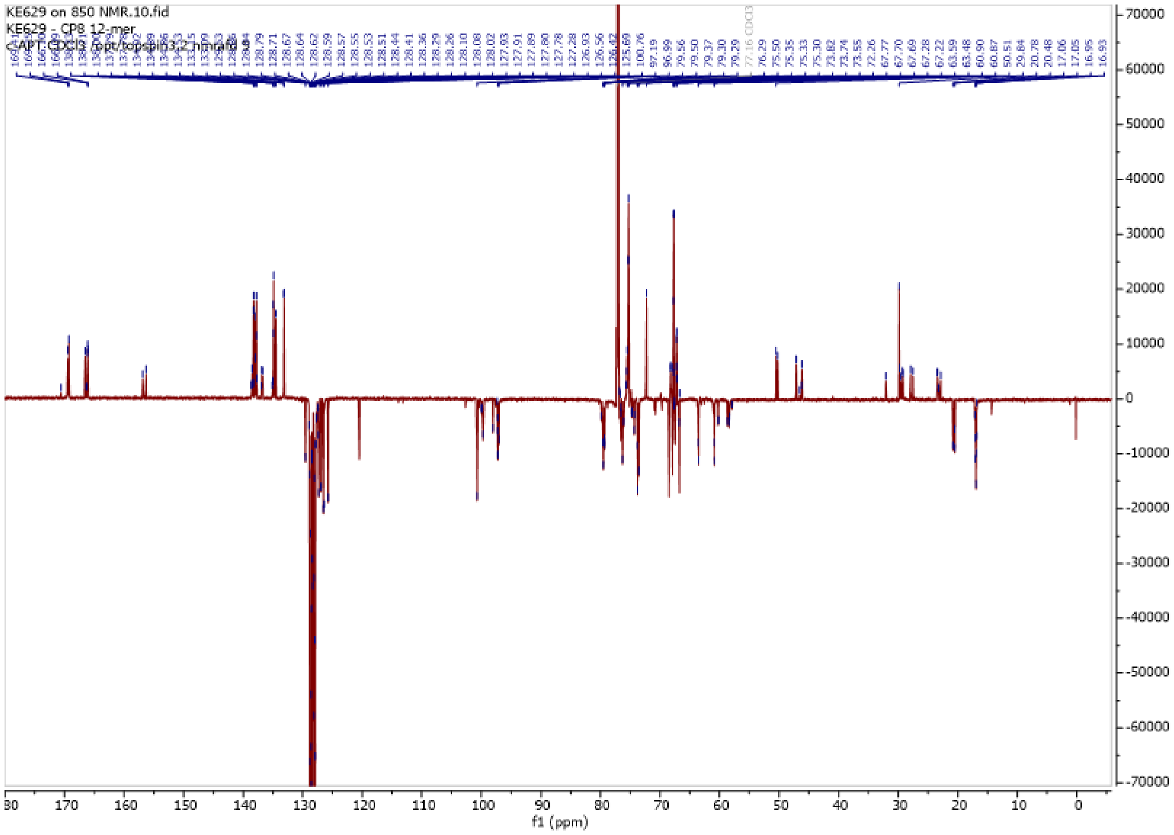
KE629 on 850 NMR.10.fid
KE629 - CP8 12-mer
APT CDCl3 /opt/topspin3.2 nmrafd 9
f1 (ppm)

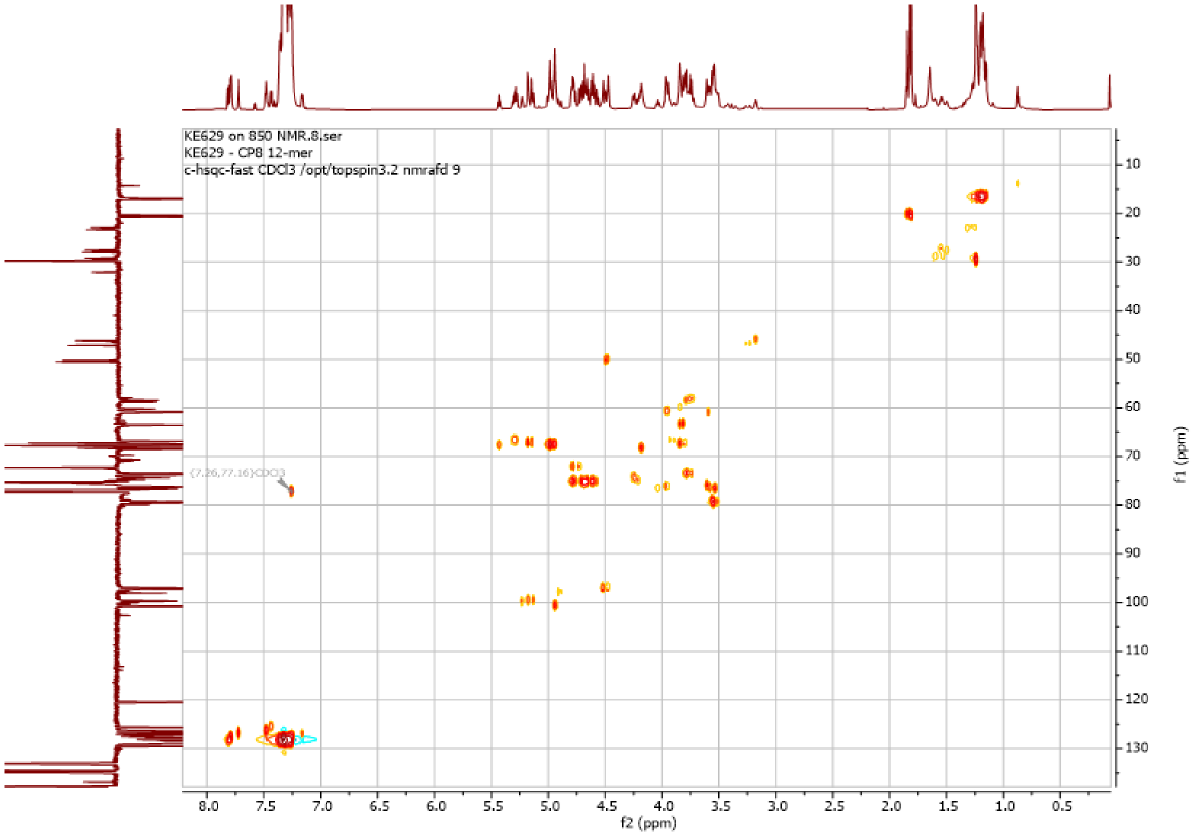

KE629 on 850 NMR.8.ser
KE629 - CP8 12-mer
c-hsqc-fast CDCl3 /opt/topspin3.2 nmrafd 9
f2 (ppm)
f1 (ppm)

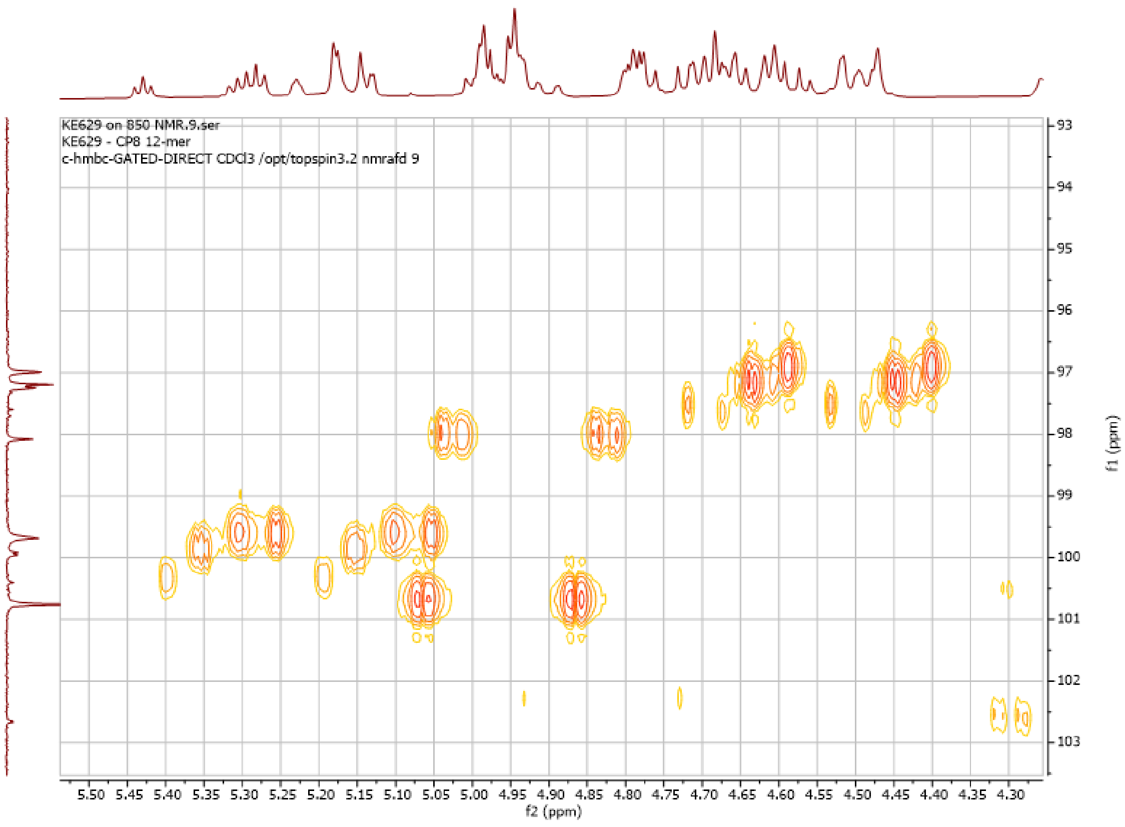

KE629 on 850 NMR.9.ser
KE629 - CP8 12-mer
c-hmbc-GATED-DIRECT CDCl3 /opt/topspin3.2 nmrafd 9
f2 (ppm)
f1 (ppm)

CP8-Dodeca (4)

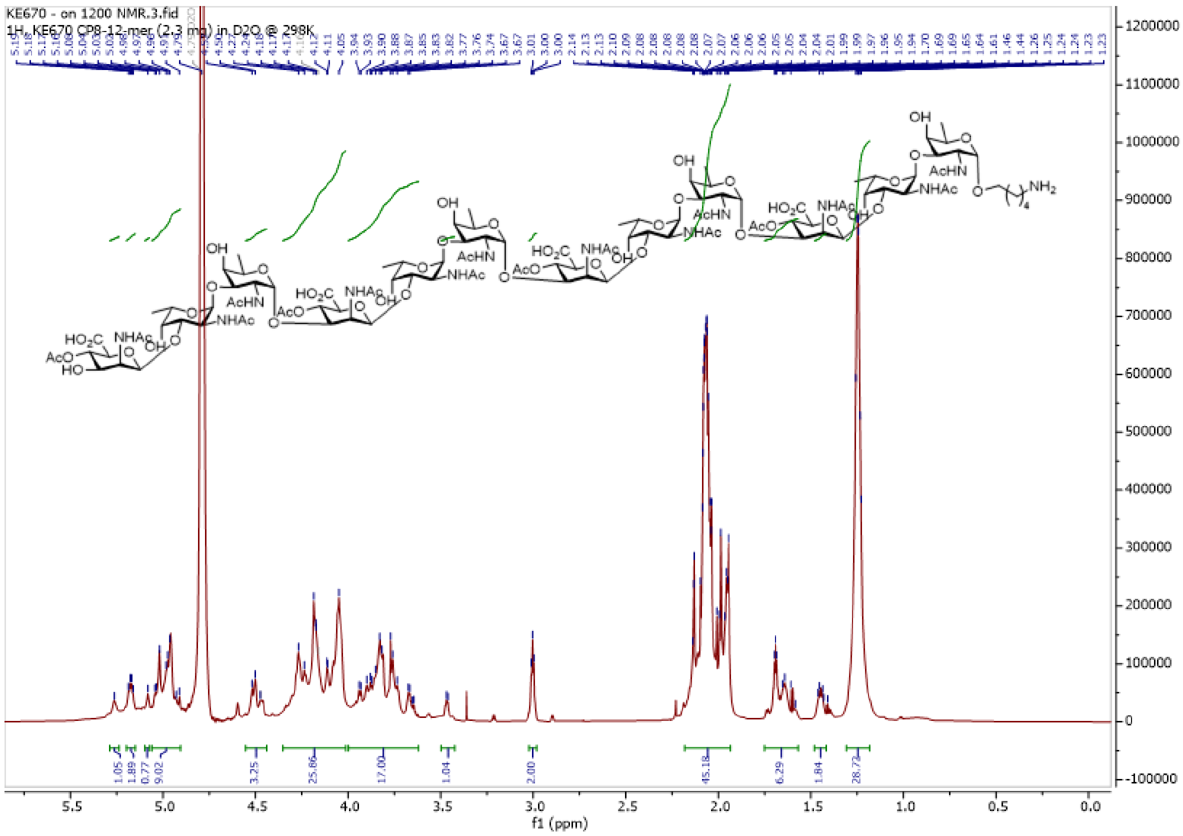

KE670 - on 1200 NMR.3.fid
1H, KE670 CP8-12-mer (2.3 mg) in D2O @ 298K
f1 (ppm)

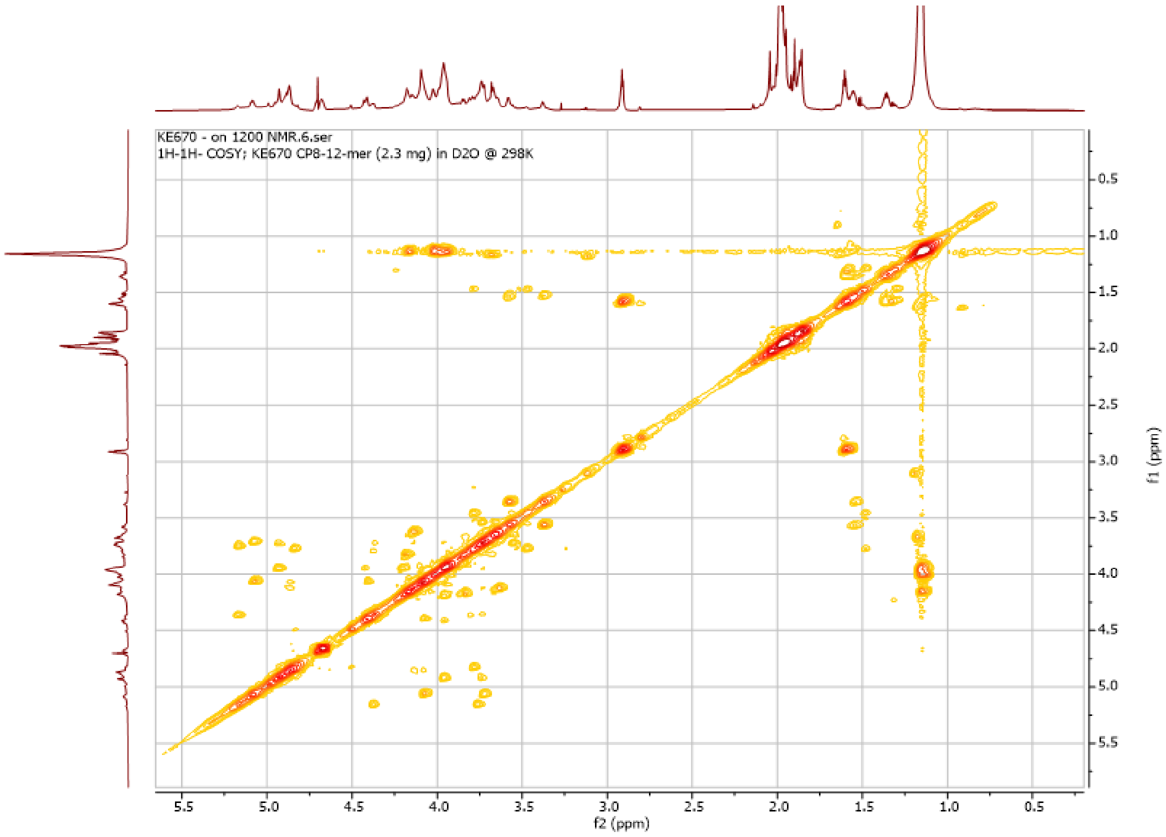

KE670 - on 1200 NMR.6.ser
1H-1H- COSY; KE670 CP8-12-mer (2.3 mg) in D2O @ 298K
f2 (ppm)
f1 (ppm)

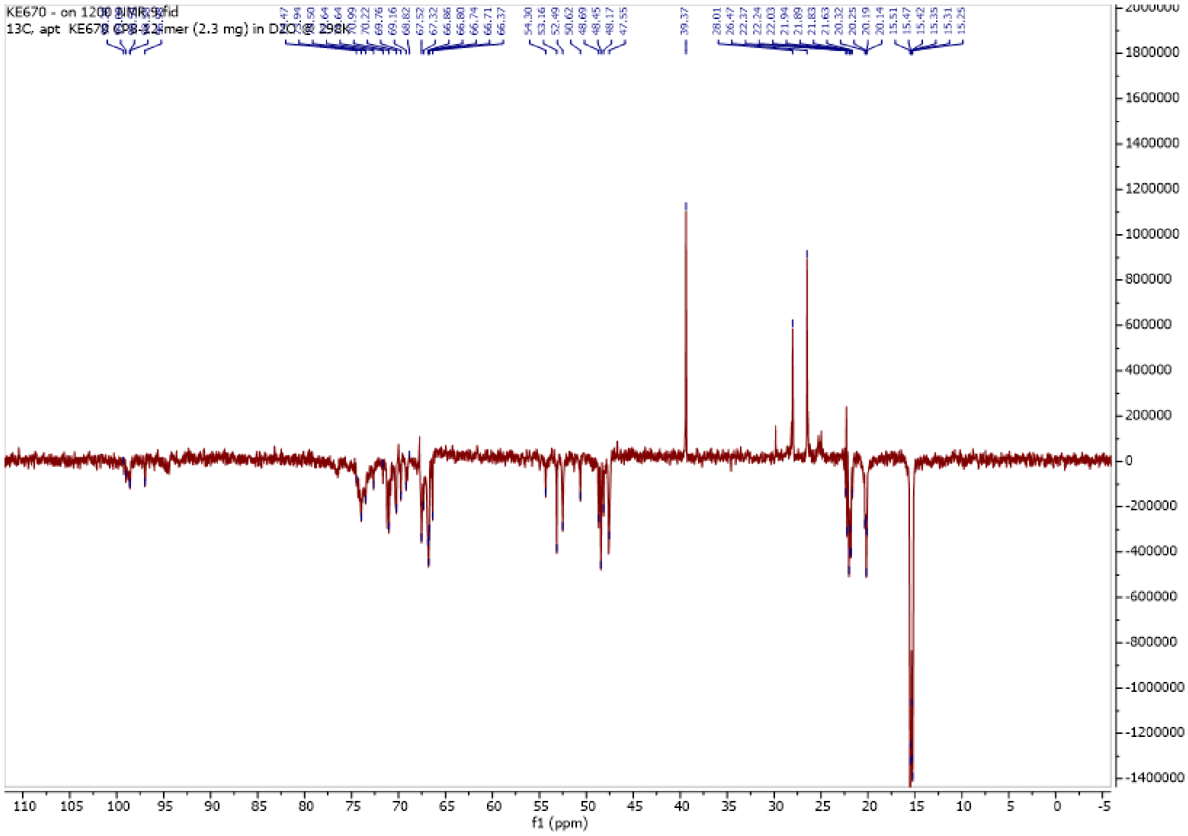
13C, apt KE670 CP8-12-mer (2.3 mg) in D2O @ 298K
70.99
70.22
69.76
69.16
68.82
67.52
67.32
66.86
66.80
66.74
66.71
66.37
54.30
53.16
52.49
50.62
48.69
48.45
48.17
47.55
39.37
28.01
26.47
22.37
22.24
22.03
21.94
21.89
21.83
21.63
20.32
20.25
20.19
20.14
15.51
15.47
15.42
15.35
15.31
15.25
f1 (ppm)

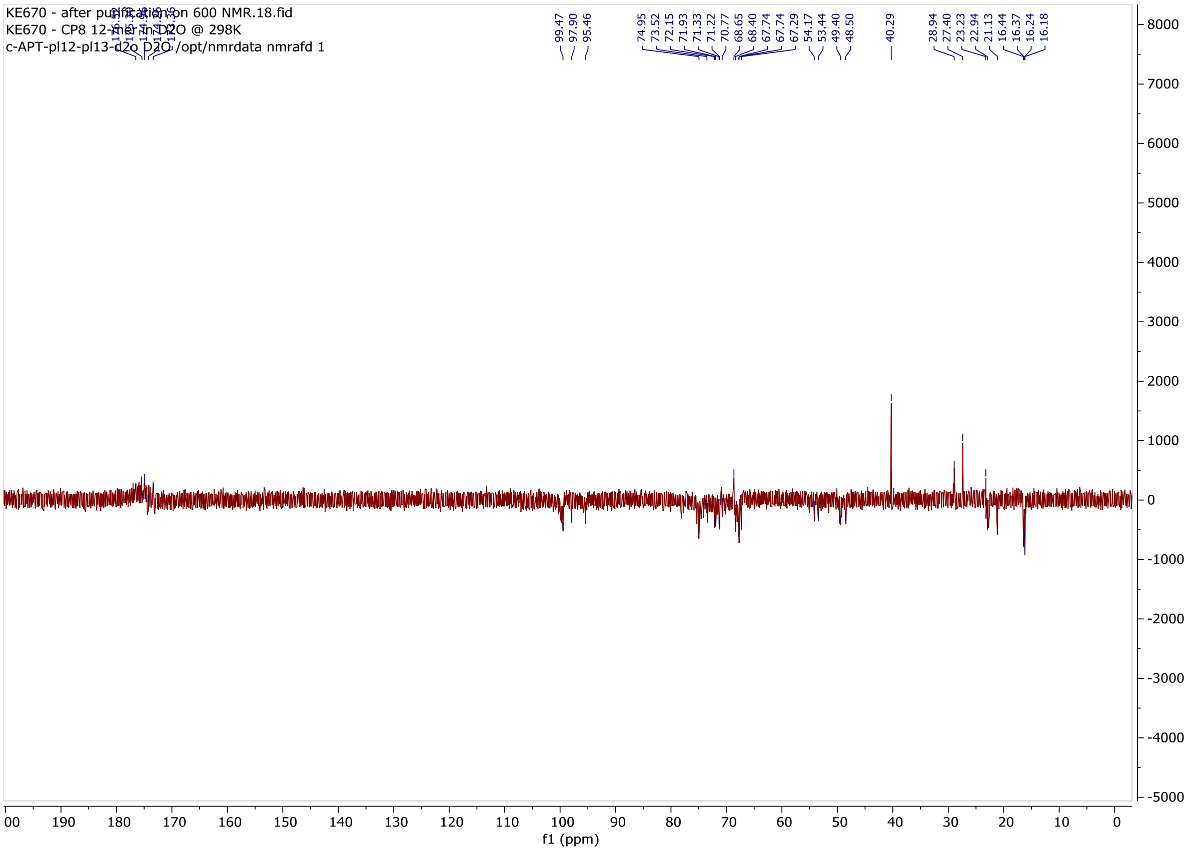
KE670 - after purification on 600 NMR.18.fid
KE670 - CP8 12-mer in D2O @ 298K
c-APT-pl12-pl13-d2o D2O /opt/nmrdata nmrafd 1
99.47
97.90
95.46
74.95
73.52
72.15
71.93
71.33
71.22
70.77
68.65
68.40
67.74
67.29
54.17
53.44
49.40
48.50
40.29
28.94
27.40
23.23
22.94
21.13
16.44
16.37
16.24
16.18
f1 (ppm)

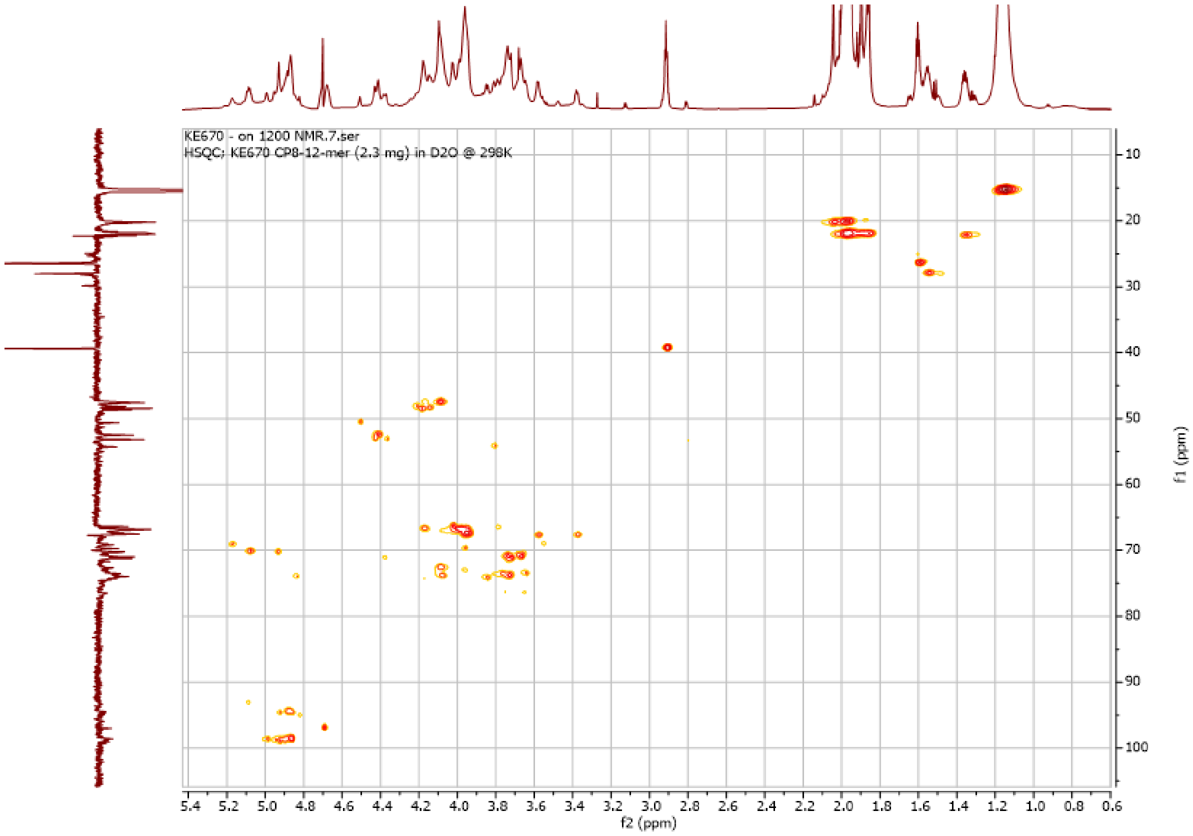

CP8-deAc-Hexasaccharide (2-deAc)

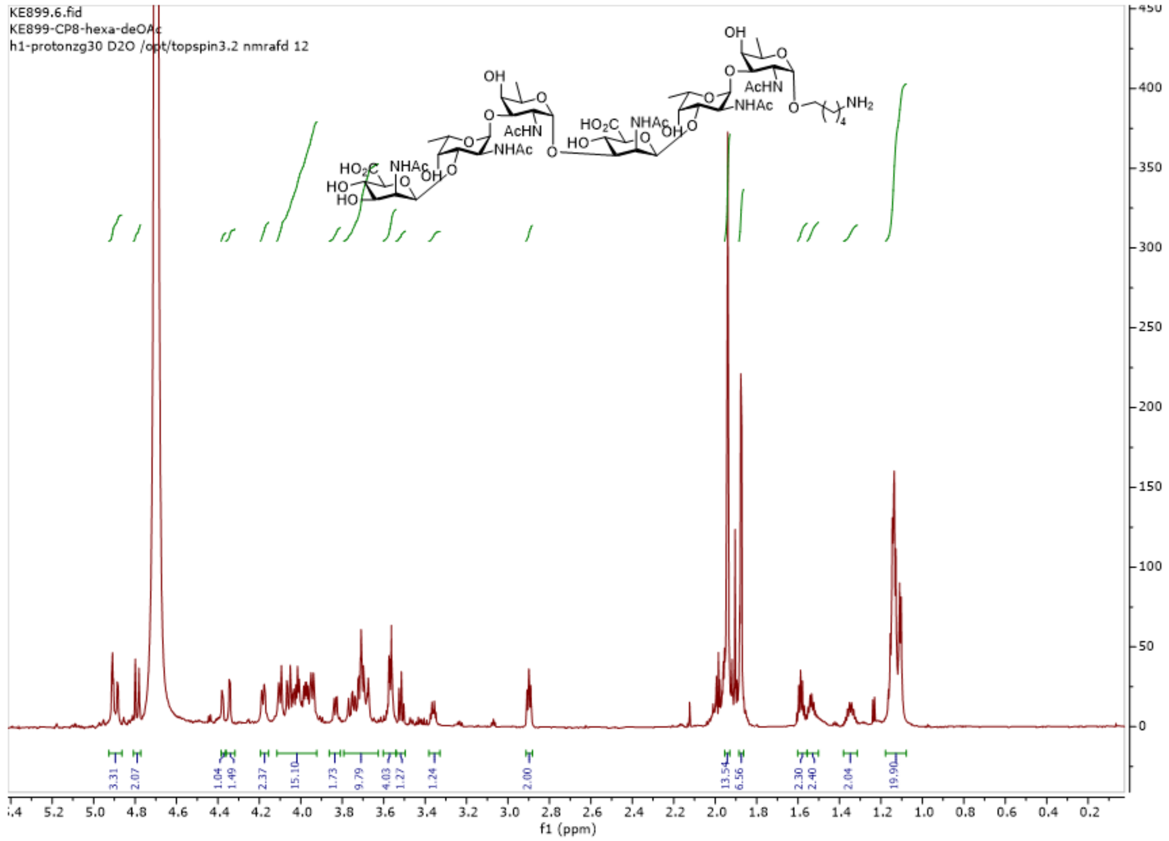

CP8-deAc-Nonasaccharide (3-deAc)

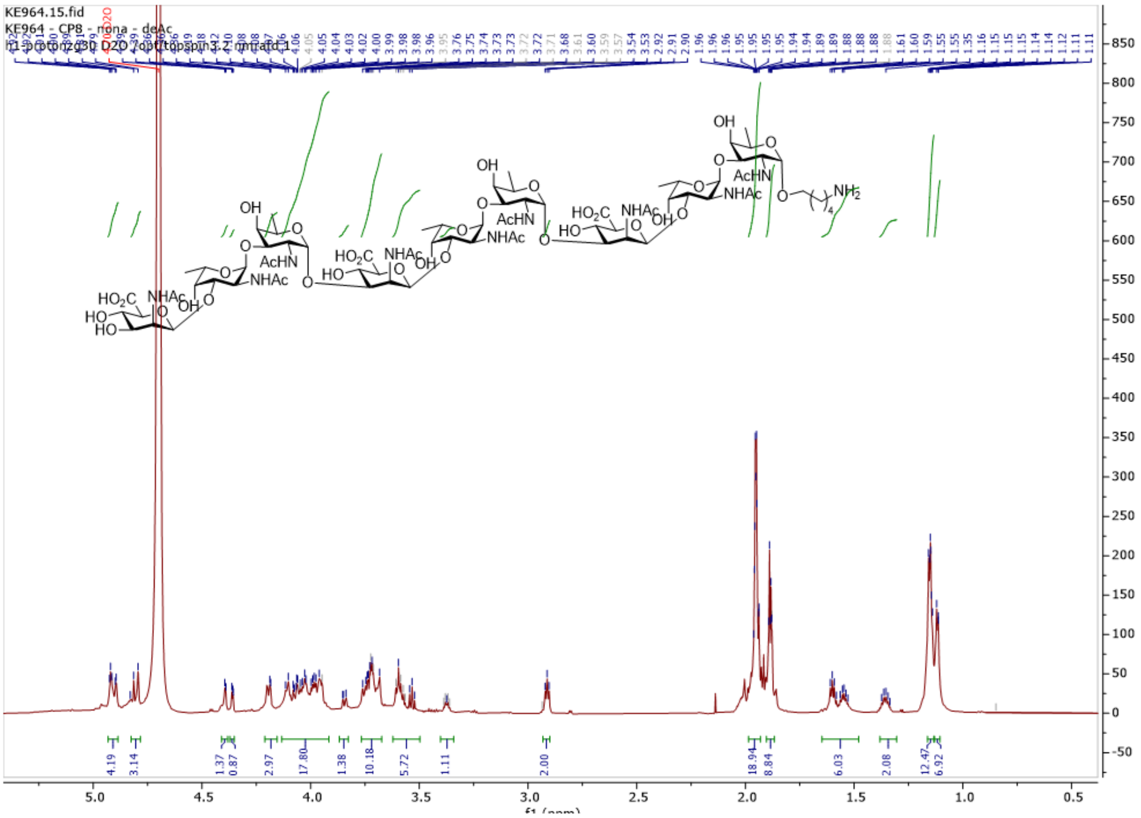

Supplement: Supplementary file 1 — ja4c16118_si_001.pdf [file ja4c16118_si_001.pdf]
